# Catalytic Asymmetric 1,2-Migration/Allylation of Alkynyl Boronate Complexes: A Modular Route to Enantioenriched Skipped 1,4-Dienes

**DOI:** 10.1021/jacs.5c19143

**Published:** 2025-12-23

**Authors:** Liang Wei, Zhuowen Guo, Jasper L. Tyler, Varinder K. Aggarwal

**Affiliations:** School of Chemistry, 1980 University of Bristol, Cantock’s Close, Bristol, BS8 1TS, U.K.

## Abstract

Stereodefined skipped 1,4-dienes are key structural motifs found in a wide range of natural products and bioactive molecules. However, their stereo- and enantioselective syntheses remain a significant challenge. While electrophilic-allylation-triggered 1,2-migration reactions of alkynyl boronate complexes are in principle a straightforward method to produce skipped dienes, such reactions suffer from poor stereoselectivity or undesired regioselectivity. We now report that, through judicious selection of the alkynyl boronate complex, we can achieve a highly regio-, diastereo-, and enantioselective construction of polysubstituted skipped 1,4-dienes via an Ir-catalyzed 1,2-migration/allylation strategy. Furthermore, the resulting alkenylboron species could be intercepted, which provides a gateway to even more challenging tetrasubstituted alkene-containing skipped dienes.

## Graphical abstract

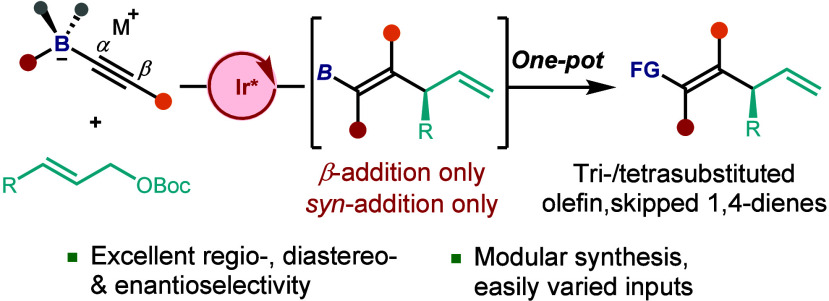

Alkenes are ubiquitous structural motifs in natural products, pharmaceuticals, and functional materials, and their stereoselective synthesis continues to be a central focus of modern organic chemistry.^1,2^ Among them, skipped 1,4-dienes represent particularly valuable targets due to their prevalence in bioactive molecules and their versatility as synthetic building blocks (Scheme 1a).^3^ However, general and efficient catalytic methods to access these motifs with high regio-, diastereo-, and enantioselectivity remain limited.^4^ Transition-metal-catalyzed asymmetric alkenylation of allylic electrophiles^5–8^ and hydroalkenylation of 1,3-dienes^8,9^ have been developed to construct this core structure. While successful, these methods are not able to construct fully substituted alkenes and lack the flexibility of a modular approach.

**Scheme 1:**
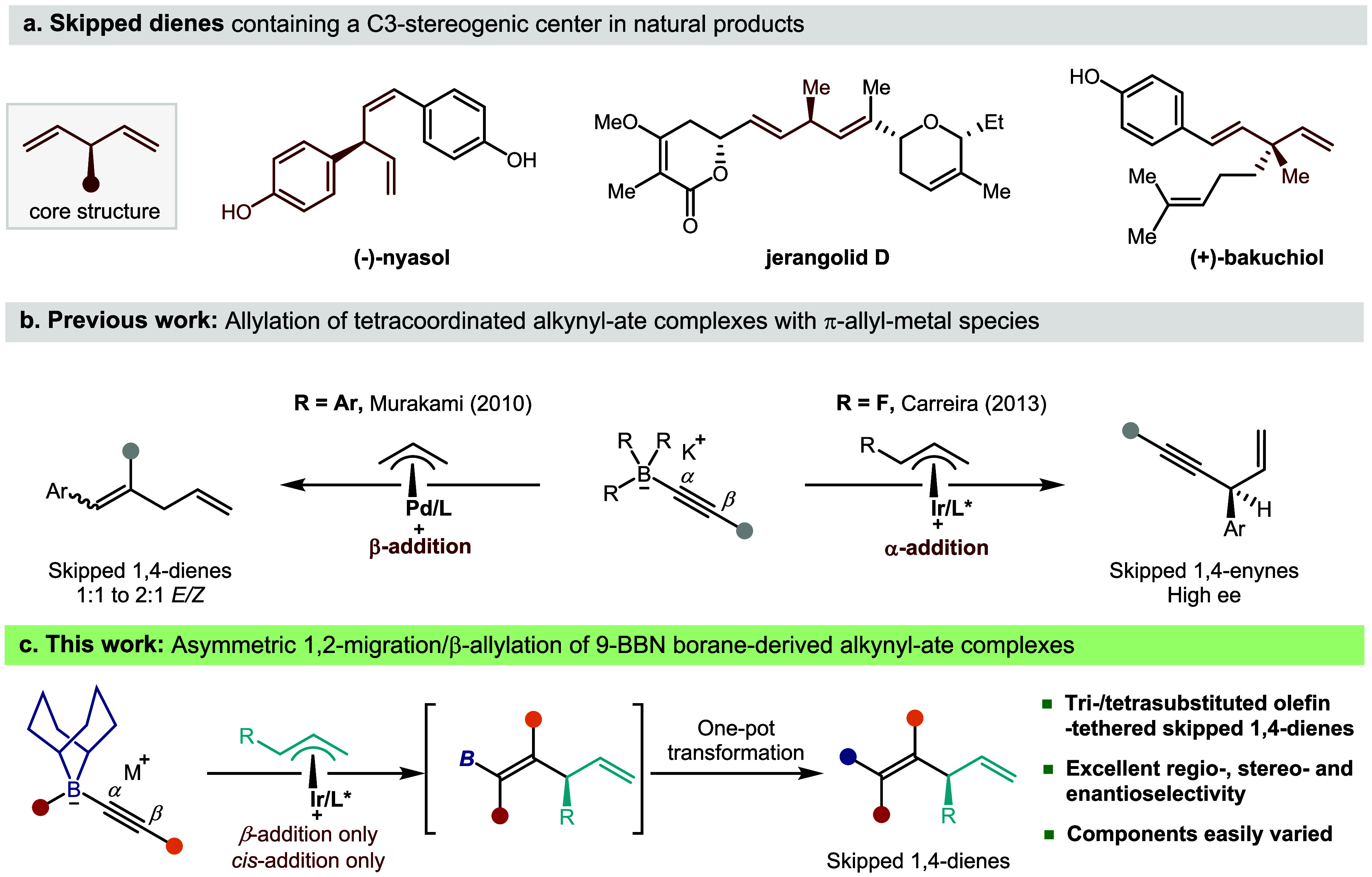
Reactivity of Tetracoordinated Alkynyl Boronate Complexes with π-Allylmetal Species, Previous Work and Our Design

We envisioned that skipped 1,4-dienes could be accessed by the reaction of an alkynyl boronate complex with an electrophilic π-allyl metal species.^10–13^ Indeed, Murakami demonstrated that π-allylpalladium complexes were capable of triggering 1,2-metalate rearrangements of triarylborane-derived alkynyl boronates to generate skipped 1,4-dienes.^14^ However, the migration was nonselective, giving an ∼1:1 mixture of *E*/*Z*-isomers. Carreira later showed that potassium alkynyl trifluoroborate salts reacted with π-allyliridium complexes to give skipped 1,4-enynes; the alkynyl trifluoroborate salt reacted exclusively at the C–B bond (Scheme 1b).^15^ These examples show that the inherent linear geometry and distinct electronic properties of alkynes pose unique challenges in 1,2-metalate rearrangements, including controlling regioselectivity and achieving high levels of diastereocontrol. Nonetheless, a catalytic asymmetric alkynyl 1,2-migration would offer a direct, modular route to skipped dienes bearing a stereodefined, fully substituted chiral alkene. Furthermore, if the resulting alkenylboron species could be intercepted, it would offer a gateway to an even broader class of tetrasubstituted alkenes with an allylic stereocenter, a structural motif notoriously difficult to access enantioselectively.

We recognized that judicious selection of the alkynyl boronate complex would be critical to modulate the regio- and stereoselectivity of the process. We previously reported that, in contrast to pinacol boronic esters, 9-borabicyclo[3.3.1]­nonane (9-BBN)-derived alkynyl boronate complexes could react with a broad range of electrophiles to give trisubstituted alkenylboranes with high selectivity.^16^ In these reactions, the electrophile and boron substituent exclusively add *syn* to the alkyne. We now report that these intermediates react with π-allyliridium complexes^17,18^ to give highly valuable skipped 1,4-dienes with excellent control of regio-, diastereo- and enantioselectivity. Mechanistic studies, including ^11^B NMR experiments, revealed the identity of the key intermediates and provided insight into the reaction pathway. These findings guided the development of conditions for the further elaboration of the trisubstituted alkenylborane products to previously inaccessible tetrasubstituted skipped 1,4-dienes, again with very high selectivity.

We began our investigation with alkynyl boronate complex **3a** generated *in situ* from phenyl 9-BBN (formed from phenyl magnesium chloride and B-methoxy-9-BBN) and 1-pentynyllithium (see Supporting Information Tables S1 and S2 for details). For the electrophile, we selected the π-allyliridium complex derived from linear allyl carbonate **4a**, [Ir­(cod)­Cl]_2_ and **L1**.^19^ After optimization of the reaction parameters, we were pleased to find conditions that furnished skipped diene **5** as a single regio- and diastereoisomer in 86% yield with >99:1 e.r. (Table 1, entry 1). For ease of isolation, workup with AcOH was conducted to facilitate the protodeboronation of the initial trisubstituted alkenylborane product. Performing the reaction at room temperature versus 50 °C led to lower yields (entry 2). The use of branched allyl carbonate **4a′** and [Ir­(cod)­Cl]_2_/Carreira’s (phosphoramidite olefin) ligand **L2** ^20^ was not effective in THF (entry 3) but provided comparable levels of regio- and stereoselectivity in CH_2_Cl_2_, albeit in slightly lower yield (entry 4). The isolated iridium complex (*S*,*S*,*S*)-Ir­(III) gave a slightly higher yield and was therefore chosen for the substrate scope examination (entry 5). Switching from Ir to Pd gave the linear rather than the branched β-allylation product in a moderate yield (entry 6). Pinacol boronic esters, or boronic acids, in place of 9-BBN boranes, were ineffective presumably because the C­(sp)–B boronate complex easily fragments to form a metalated-alkyne, which is subsequently trapped by the electrophile (α-trapping, see Supporting Information).^16^

**Table 1:**
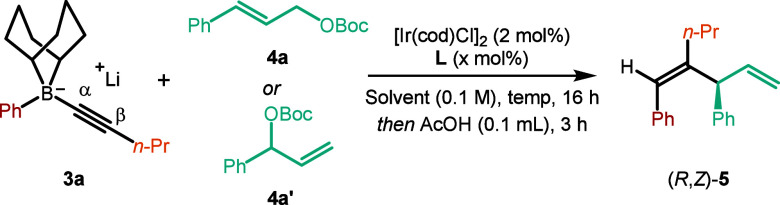
Screening of π-Allyl Metal Species for the Asymmetric 1,2-Migration/Allylation of **3a** ^a^

Entry	Conditions	Yield (%)^c^	α/β[Table-fn t1fn4]	*Z*/*E* [Table-fn t1fn4]	*b*/*l* [Table-fn t1fn4]	e.r.
1	**L1** (4 mol %), **4a**, THF, 50 °C	86	>20:1	>20:1	>20:1	>99:1
2	**L1** (4 mol %), **4a**, THF, 25 °C	65	>20:1	>20:1	>20:1	>99:1
3	**L2** (8 mol %), **4a′**, THF, 50 °C	<10	n.d	n.d	n.d	n.d
4	**L2** (8 mol %), **4a′**, DCM, 25 °C	69	>20:1	>20:1	>20:1	>99:1
5	(*S*,*S*,*S*)-**Ir(III)** (4 mol %), **4a**, THF, 50 °C	**88**	****>**20:1**	****>**20:1**	****>**20:1**	****>**99:1**
6	Pd-**L1** (5 mol %), **4a**, THF, 25 °C	21	>20:1	10:1	1:11	n.d

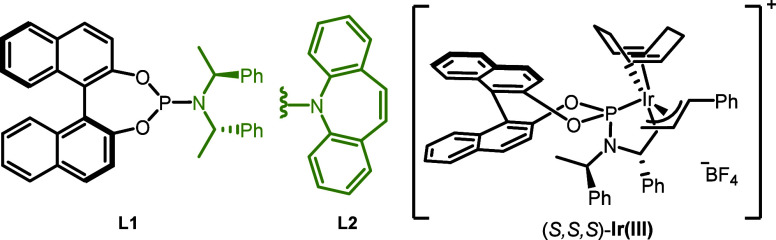

a All reactions were conducted with 0.2 mmol of **3a** and 0.24 mmol of **4a** or 0.44 mmol of **4a′** in 2 mL of solvent.

b Isolated yields given.

c Determined by crude ^1^H NMR.

With the optimized reaction conditions in hand, we then set out exploring the generality of this methodology toward the synthesis of trisubstituted skipped 1,4-dienes (Table 2). Alkynyl boronate complexes (**3**) derived from alkynes bearing primary and secondary alkyl substituents all reacted smoothly with **4a**, giving dienes **5**–**10** in 52%–88% yield, with complete regio- and diastereocontrol and excellent enantioselectivities. 1-Propyne could also be employed, leading to methyl-substituted 1,4-diene **11** in good yield and high selectivity. However, tertiary-alkyl-substituted alkynes only gave traces of the product. Arylalkynes are more challenging substrates since the negative charge of the boronate complex can be delocalized onto the aromatic ring and thus decrease its nucleophilicity. Indeed, under standard conditions, the reaction of the phenylethynyl boronate complex and **4a** afforded the desired product in low yield and a large amount of **4a** was recovered. Fortunately, increasing the concentration led to higher reactivity, giving **12** in 62% yield with essentially complete regio-, diastereo-, and enantioselectivity. In addition to simple alkynes, terminal alkynes bearing olefins, halides, ethers, alcohols, amines, sulfides, and heterocycles (**13**–**19**) proved to be viable reaction partners. TMS-alkyne, like Carreira’s alkynyl trifluoroborate salts,^15^ reacted with π-allyliridium complexes at the α- rather than the β-position, to give a skipped 1,4-enyne (see Supporting Information Scheme S1).

**Table 2:**
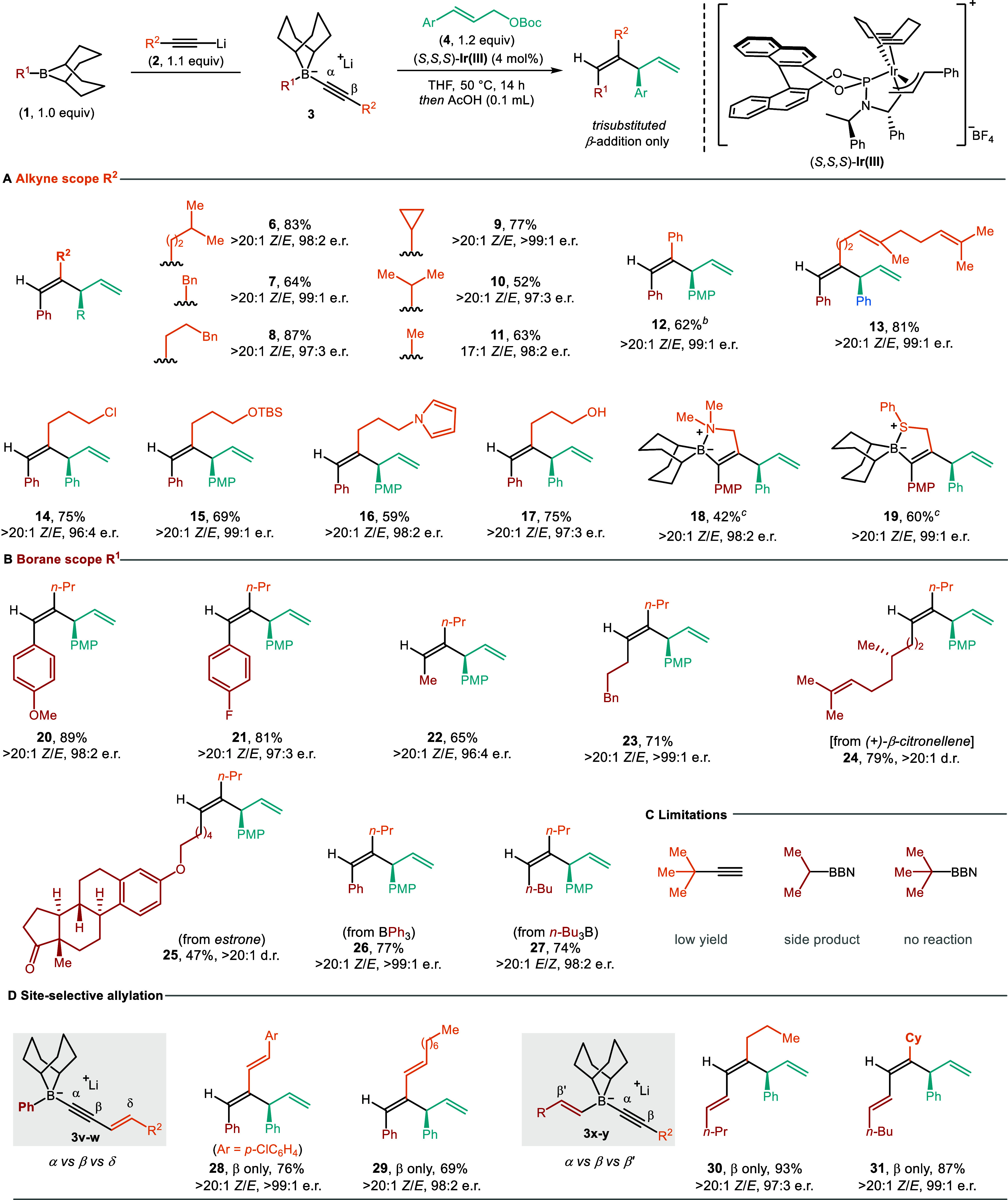
Scope and Limitations of the Alkynyl Boronate Complex^a^

a All reactions were conducted on a 0.2 mmol scale. E.r. was determined by chiral HPLC, either directly or after hydroboration/oxidation.

b THF (0.3 M) was used.

c Without AcOH work-up.

We then explored an array of boranes, which were generated *in situ* from the hydroboration of terminal alkenes or by substitution of B-methoxy-9-BBN with organometallic reagents and used directly without isolation (Table 2b). Both electron-rich and -deficient aryl boranes underwent the desired process, giving **20**–**21** in high yield and excellent enantioselectivity. Methyl- and 3-phenylpropyl boranes were also found to be suitable reactants (**22**, **23**). Notably, boranes derived from the hydroboration of (+)-β-citronellene and an estrone derivative afforded **24** and **25**, respectively, in moderate to high yield and >20:1 d.r. In addition to substituted 9-BBN boranes, we then showed that commercially available triphenylborane and tri-*n*-butylborane-derived boronate complexes exhibited comparable reactivity and selectivity under our standard conditions, giving dienes **26** and **27**. Remarkably, when conjugated alkynyl boronate complexes **3v**–**3w** and **3x**–**3y** that contain multiple reactive sites were subjected to this reaction, the nucleophilic addition to π-allyliridium species occurred exclusively at the β-C­(sp) position, providing a variety of structurally diverse polyenes (**28**–**31**) in high yield with almost exclusive chemo-, regio-, diastereo-, and enantioselectivities. These results are striking because (i) related Pd-catalyzed 1,2-migration/arylation reactions of enyne boronate complexes,^21^ which are structurally similar to **3v** and **3w**, resulted in exclusive reaction at the remote δ-position; (ii) alkenes are usually more reactive toward electrophiles than alkynes, whereas in these alkenyl–alkynyl boronate complexes **3x** and **3y**, the alkyne reacted exclusively. Secondary and tertiary alkyl boranes were less effective, giving either a small amount of BBN ring-migration product or unreacted starting materials, indicating that the current 1,2-migration/allylation process is sensitive to steric hindrance, particularly at the R^1^ position.

We then turned our attention to the scope of the electrophilic component. To simplify the procedure, the tetramethylammonium salt **3a**-**NMe**_**4**_ was used, since it could be easily isolated on scale, upon cation exchange of **3a** with tetramethylammonium chloride. We first explored a series of cinnamyl carbonates bearing different functionalities (Table 3). Electron-rich, electron-deficient, and heterocyclic carbonates were well tolerated, giving 1,4-dienes **32**–**38** in high yield and high selectivity. *Ortho*-methyl cinnamyl carbonate provided **33** in 60% yield and 97:3 e.r. albeit with a reduced 7:1 *Z*/*E* ratio, likely caused by its increased steric hindrance.^17^ Moreover, alkenyl- and alkynyl-substituted branched allyl carbonates, which are easier to prepare than their linear analogues, were all successfully applied to our protocol using [Ir­(cod)­Cl]_2_/**L2** as the catalyst, affording the corresponding enantiomerically pure triene and dienynes **39**–**41** in moderate yields. Allyl carbonates bearing an alkyl group often fail in iridium-catalyzed allylations due to reduced reactivity of the π-allyliridium intermediate.^6^ However, we were pleased to find that simple methyl and *n*-propyl substituted carbonates worked effectively, giving **42** and **43** in high yield and high diastereoselectivity, enantioselectivity, and branch/linear selectivity. Furthermore, allyl carbonate derived from (−)-citronellal gave **44** in 81% yield with >20:1 d.r. Success with these alkyl substrates is of particular interest as skipped 1,4-dienes bearing C3-methyl groups are widespread in natural products.^3^

**Table 3:**
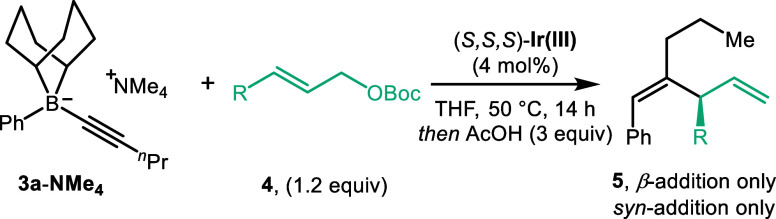

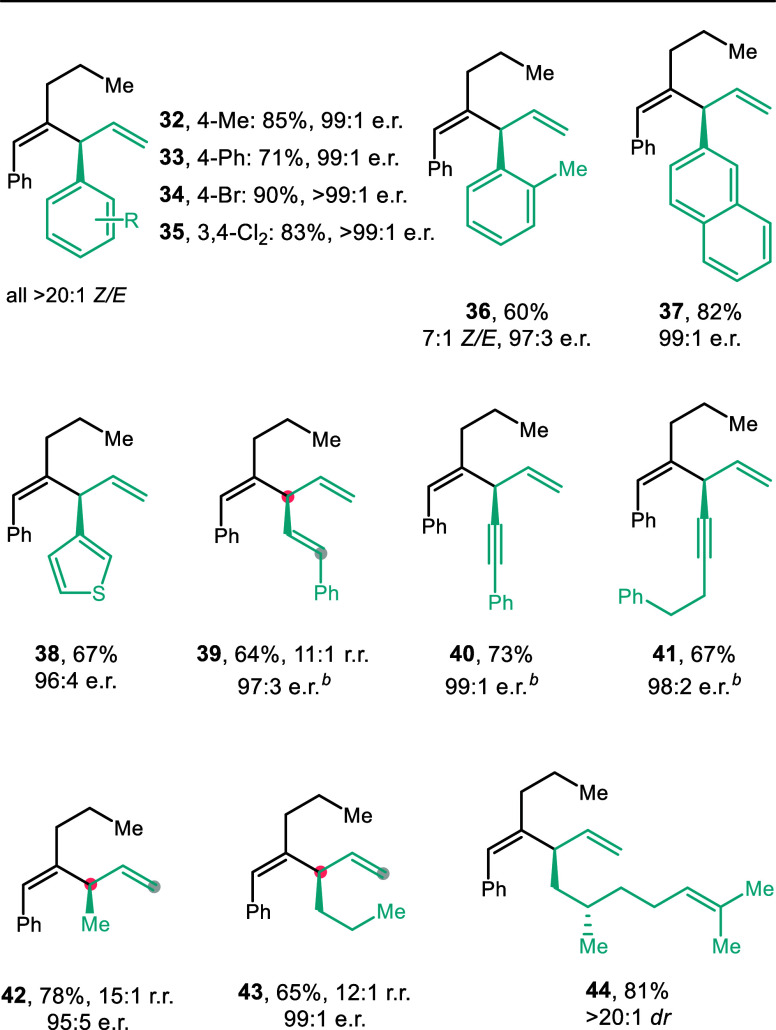
Scope of Allyl Carbonates^a^

a All reactions were conducted on a 0.2 mmol scale.

b [Ir­(cod)­Cl]_2_ (2 mol %), **L2** (8 mol %) and the corresponding branched allyl carbonate (2.2 equiv) were used. R.r. was determined by crude ^1^H NMR.

We subsequently explored alternative procedures other than protodeboronation that could exploit the synthetic utility of the alkenylborane intermediate through further transformations. Unfortunately, attempts to form the corresponding borinic ester using TMANO,^22^ or to engage the intermediate borane in either cross-coupling or Zweifel olefination,^16^ all failed, and the protodeboronated trisubstituted alkene **5** was isolated as the major product in all cases. Indeed, analysis of the reaction mixture by ^11^B NMR prior to workup indicated the absence of the expected alkenylborane intermediate. Instead, three signals at −19.0, 11.1, and 56.3 ppm were observed, which were assigned to **3a**-**NMe**_**4**_, tetracoordinated boron species **Int I**, and B*-* ^*t*^BuO-BBN, respectively (Figure 1b). Based on this NMR analysis, we propose the following mechanism for the reaction (Figure 1c). The decarboxylative oxidative addition of the iridium catalyst to *t*-butyl cinnamyl carbonate **4a** generates the chiral π-allyliridium species, carbon dioxide and *t*-butoxide. The π-allyliridium species induces a concerted 1,2-migration/allylation reaction giving tetrasubstituted alkenyl-BBN **45′**, which, being electrophilic, is attacked by *t*-butoxide to form tetracoordinated boronate **Int I**. Subsequent protonation from adventitious water or on workup gives trisubstituted alkene **5**.

**Figure 1:**
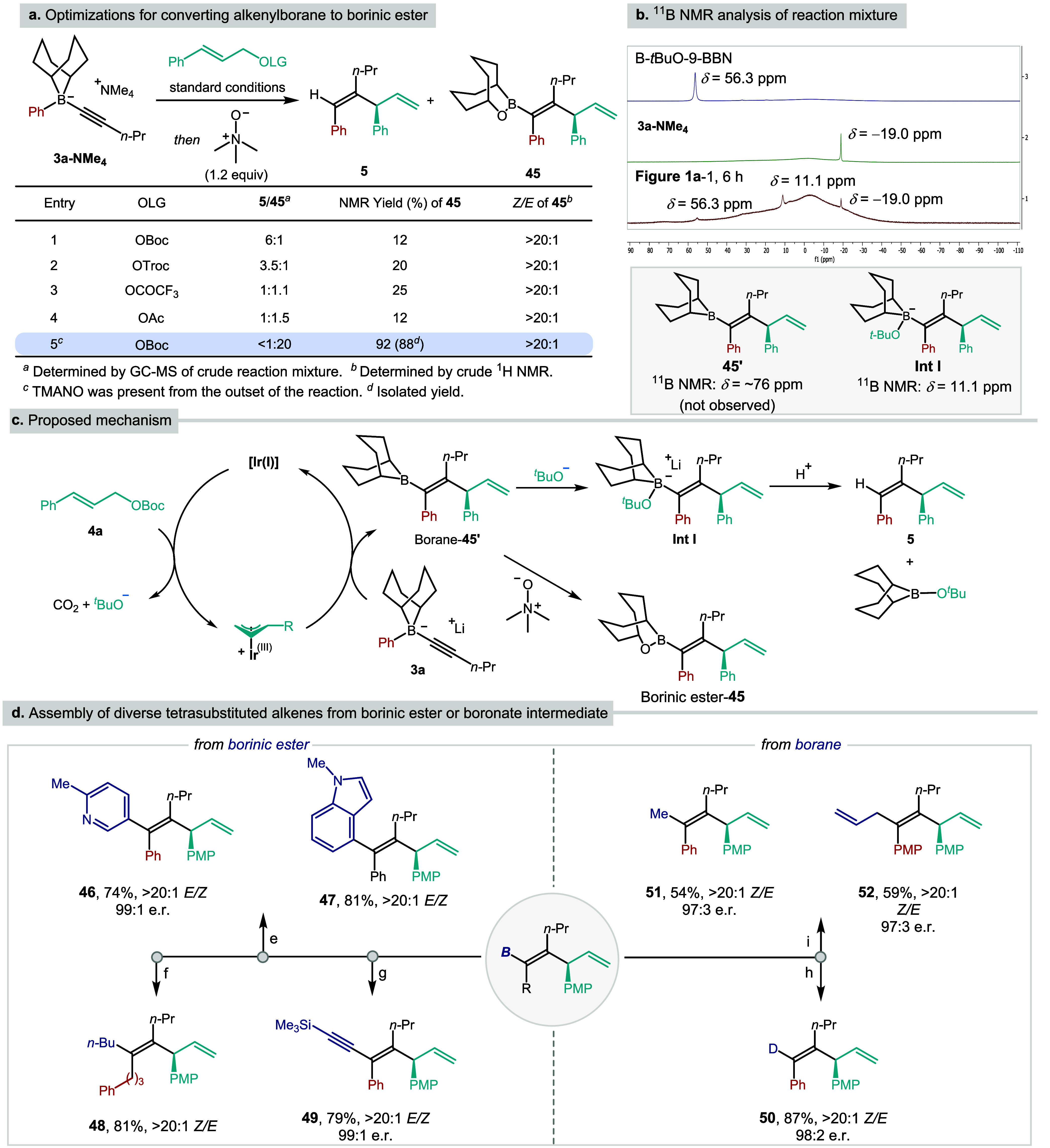
Mechanistic studies and synthesis of chiral tetrasubstituted alkenes. Conditions for diversifications: (e) Pd­(OAc)_2_/SPhos, arylbromide, K_3_PO_4_; (f) Pd­(dba)_2_/*t*-Bu_2_PMe·HBF_4_, *n*-BuBr, KOH; (g) lithium TMS-acetylide and then I_2_/MeOH; (h) acetic acid-*d*_4_; (i) *n*-BuLi, then CuI, MeI or allylbromide.

To enable manipulation of the borane intermediate, it was clearly essential to prevent nucleophilic attack by *t*-butoxide, which would otherwise lead to the formation of **Int I**. We first attempted to do this using alternative carbonate-based leaving groups that would display lower nucleophilicity upon decarboxylation. However, this strategy was unsuccessful, giving a mixture of **5** and **45** in all cases (Figure 1a, entries 1–4). Although we had found that we were unable to oxidize the borane to the less electrophilic borinic ester after the reaction, we wondered if we could effect this transformation before the borane had been captured by *t*-butoxide. This would require addition of TMANO at the outset of the reaction, but we were concerned about its compatibility with the iridium catalyst and its potential effect on enantioselectivity. In the event, our fears proved groundless, and to our delight, simply by adding TMANO simultaneously with iridium catalyst and carbonates gave the desired borinic ester **45** in 88% isolated yield (Figure 1a, entry 5), with the same enantioselectivity, as determined following cross-coupling (see below).

The ability to access these trisubstituted alkenyl borinic ester products allowed us to explore the synthesis of tetrasubstituted skipped 1,4-dienes. Since the 1,2-migration/allylation/oxidation sequence was highly efficient, we decided to use the crude reaction mixture for the further transformations directly rather than isolating the intermediate borinic esters. Thus, the Ir-catalyzed 1,2-migration/allylation could be telescoped in one pot with Pd-catalyzed Suzuki–Miyaura cross-coupling with different aryl halides. This provided tetrasubstituted alkenes **46** and **47** in high yield and high stereoselectivity.^12c^ Alkyl bromides could also be engaged using Pd­(dba)_2_/P^*t*^Bu_2_Me·HBF_4_,^23^ thereby effecting an sp^2^–sp^3^ cross-coupling to give **48**. In addition to cross-coupling, the borinic ester was readily converted to an alkynyl group (**49**) through a Zweifel-type alkynylation in a stereoretentive manner.^24^ Stereoretentive deuterodeboration of **Int I** could be effected by treatment with 3 equiv of acetic acid-*d*_4_ giving **50** in 87% yield and >99:1 e.r. Transmetalation of the alkenyl borane to copper was also feasible, and trapping the resulting nucleophilic alkenyl-copper species with methyl iodide and allyl bromide, gave 1,4-dienes **51** and **52** in moderate yield and high selectivity.^25^

In summary, we have discovered that 1,2-metalate rearrangements of alkynyl boronates can be triggered by π-allyl-Ir species, giving a diverse range of polysubstituted skipped 1,4-dienes, with high regio-, diastereo-, and enantioselectivities. Most notably, we uncovered that the reaction could be conducted in the presence of TMANO which allowed boron to be retained through the formation of the corresponding borinic ester. This discovery enabled the interception of the boron-appended intermediate, which in turn enabled its transformation into a diverse range of functional groups.

## Supplementary Material



## Notes

Experimental procedures and characterization data for new compounds (PDF)
