# Supplementary material for: Catalytic Asymmetric 1,2-Migration/Allylation of Alkynyl Boronate Complexes: A Modular Route to Enantioenriched Skipped 1,4-Dienes
Source: J Am Chem Soc. 2025 Dec 23;148(1):106–13. doi: 10.1021/jacs.5c19143 (PMC12814179; doi:10.1021/jacs.5c19143)

Supporting Information

Catalytic Asymmetric 1,2-Migration/Allylation of Alkynyl-Boronate Complexes: A Modular Route to Enantioenriched Skipped 1,4-Dienes

Liang Wei, Zhuowen Guo, Jasper L. Tyler, Varinder K. Aggarwal*

School of Chemistry, University of Bristol, Cantock's Close, Bristol BS8 1TS, UK.

**email: v.aggarwal@bristol.ac.uk*

Table of Contents

1. MATERIALS AND GENERAL METHODS 3

2. EXPERIMENTAL DATA 5

2.1 Starting Materials and Catalysts 5

2.2 Optimization of Reaction Conditions 5

2.2.1 Catalyst preparation 5

2.2.2 General procedure for reaction optimization 6

2.2.3 Unsuccessful Examples 7

2.3. General Procedures 9

2.3.1 General Procedure A for products 5-22 and 26-29 9

2.3.2 General Procedure B for products 23-25, 30 and 31 10

2.3.3 General Procedure C for products 32-38 and 42-44 10

2.3.4 General Procedure D for products 39-41 11

2.3.5 General Procedure E for for ee determination 11

2.4. Procedures for Transformation of Products 12

2.4.1 Procedures for *in-situ* TMANO oxidation of borane to borinic ester 12

2.4.2 sp^2-sp^2 and sp^2-sp^3 cross coupling of 45 13

2.4.3 Zweifel-type alkynylation 14

2.4.4 Transmetallation/electrophilic trapping of boronate intermediate 14

2.5. Determination of Stereochemistry 15

2.5.1 Stereochemistry of 5 15

2.5.2 Stereochemistry of 49 16

2.5.3 Stereochemistry of 52 18

2.6. Proposed Reaction Mechanism 18

2.7. Characterization Data for Products. 19

3. REFERENCES 64

4. NMR SPECTRA 66

1. MATERIALS AND GENERAL METHODS

Solvents, Reagents, Glassware and Reaction Setup. Unless otherwise stated, all reactions were conducted under an inert atmosphere of nitrogen in flame dried glassware using standard Schlenk techniques. Air- and moisture-sensitive liquids and solutions were transferred via syringe into the reaction vessels through a rubber septum. Unless otherwise specified, all reagents were purchased at highest commercial quality and used as received. Non-anhydrous solvents were purchased (unless specified) at the highest commercial quality and used as received. CH_2Cl_2, Et_2O and THF were dried on an Anhydrous Engineering alumina column drying system. Temperatures described below −10 °C were achieved using Thermo Scientific EK-90 or Huber TC100E cryostats or appropriate solvent/dry ice baths.

Thin layer chromatography (TLC) was performed using Merck Kieselgel 60 F254 fluorescent treated silica, which was visualized under UV light, or by staining with aqueous basic potassium permanganate followed by heating, or Hanessian's stain (CAM stain) followed by heating, or *p*-anisaldehyde solution followed by heating, as stated.

Preparative Thin layer chromatography (PLC) was performed using Merck Z513032-1PAK TLC plates, Silica gel, which was visualized under UV light.

Chromatography was carried out using Sigma-Aldrich silica gel (60 Å, 230-400 mesh, 40-63 μm) or a Biotage Isolera One automated flash purification system, as indicated. Reactions were followed by thin-layer chromatography (TLC) where practical, using aluminium-backed Merck Kieselgel 60 F254 fluorescent treated silica gel plates, which were visualised under UV light or by staining with aqueous basic $KMnO_4$, acidic *p*-anisaldehyde solution in ethanol, or phosphomolybdic acid solution in ethanol.

NMR were recorded at various field strengths, as indicated, using Bruker 400 MHz, Varian VNMR 400 MHz, or Bruker Cryo 500 MHz for ^{1}H, ^{11}B, and ^{13}C acquisitions. All NMR spectra were recorded at 25 °C unless otherwise stated. Chemical shifts (δ) are reported in parts per million (ppm) and referenced to $CDCl_3$ (^{1}H: 7.26 ppm; ^{13}C: 77.16 ppm) or CD_2Cl_2 (^{1}H: 5.32 ppm; ^{13}C: 53.84 ppm). Coupling constants (J) are given in Hertz (Hz) and refer to apparent multiplicities (s = singlet, d = doublet, t = triplet, q = quartet, quin = quintet, hex = hextet, h = heptet, m = multiplet, brs = broad signal, dd = doublet of doublets, etc.). The ^{1}H NMR spectra are reported as follows: chemical shift (multiplicity, coupling constants, number of protons).

HRMS (high resolution mass spectra) were recorded on a Bruker Daltonics MicrOTOF II by Electrospray Ionisation (ESI); a Thermo Scientific QExactive by Electron Ionisation (EI); or a Bruker UltrafleXtreme by Matrix-assisted Laser Desorption/Ionisation (MALDI).

HPLC analyses were performed on Agilent 1100 system with Daicel Chiralpak columns.

Naming of compounds. Compound names are generated by ChemDraw Professional 20.0 software (PerkinElmer), following the IUPAC nomenclature.

2. EXPERIMENTAL DATA

2.1 Starting Materials and Catalysts

Linear allylic carbonates were synthesized according to a relevant literature[1] from allylic alcohols or aldehydes.

Branched allylic carbonates were synthesized according to a relevant literature[2] from aldehydes and vinyl magnesium bromide.

Substituted boranes were synthesized according to a relevant literature[3] from substitution of *B*-MeO 9-BBN with organometallic reagents or hydroboration of alkene/alkyne with 9-*H*-BBN. Triphenylborane and tributylborane were commercially available and used as received.

All alkynes were commercially available and used as received.

$[Ir(cod)Cl]_2$, **L1** and **L2** were commercially available and used as received.

2.2 Optimization of Reaction Conditions

2.2.1 Catalyst preparation

In situ preparation of iridium catalyst Ir(I)/**L1**: A flame dried 7 mL vial equipped with a magnetic stir bar was charged with $[Ir(cod)Cl]_2$ (2 mol%) and **L1** (4 mol%) and was purged with nitrogen (N_2) three times. To this vial was added 0.5 mL of THF (anhydrous and degassed) and 0.5 mL of *n*-propylamine (anhydrous and degassed) under nitrogen. The mixture was stirred at 50 °C for 30 minutes before cooling down to room temperature. All volatile materials were carefully removed under reduced pressure to afford desired Ir/**L1** complex as a red-brown solid.

In situ preparation of iridium catalyst Ir(I)/**L2**: A flame dried 7 mL vial equipped with a magnetic stir bar was charged with $[Ir(cod)Cl]_2$ (2 mol%) and **L2** (8 mol%) and was purged with nitrogen (N_2) three times. 1.0 mL of indicated solvent (anhydrous and degassed) was added under nitrogen and the mixture was stirred at 25 °C for 30 minutes.

Preparation of iridium catalyst (*S,S,S*)-**Ir(III)**: prepared following literature[4] with modifications: A flame-dried Schlenk tube under an argon atmosphere, a solution of $[Ir(cod)Cl]_2$ (167 mg, 0.25 mmol) and **L1** (270 mg, 0.50 mmol) in 5 mL of dry THF was stirred at room temperature for 30 minutes. The methyl cinnamyl carbonate (1.0 mmol) and $AgBF_4$ (0.60 mmol) were added to the orange solution causing the formation of a white precipitate. The mixture was stirred for 30 minutes and kept at room temperature for 12 hours. The mixture

was filtered through celite, giving a clean orange-red solution which was concentrated under reduced pressure. The crude iridium catalyst was purified by flash chromatography (silica gel, pentane/EtOAc = 20:1 then DCM/IPA = 98:2) to afford the Ir(III) complex as a yellow solid (88-95% yield).

2.2.2 General procedure for reaction optimization

A solution of Ir/**L** complex (1.0 mL) was transferred to a separated vial containing **3a** (0.20 mmol, 1.0 equiv), cinnamyl carbonate (0.24 mmol, 1.2 equiv) and 1.0 mL of solvent (as indicated) under nitrogen. After stirring at indicated temperature for 16 hours, the reaction was cooled down to 25 °C and acetic acid (0.1 mL) was added. After stirring the mixture for 3 hours, the crude reaction mixture was concentrated under reduced pressure and purified by flash chromatography.

Table S1: Screening of solvents and leaving groups

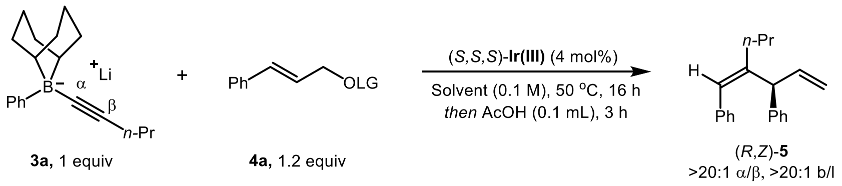

Entry	Solvent	OLG	Yield/%[a]	Z/E[b]	e.r.
1	THF	OBoc	88	>20:1	>99:1
2	DCM	OBoc	77	>20:1	>99:1
3	2-Me-THF	OBoc	69	16:1	98:2
4	Toluene	OBoc	59	19:1	98:2
5	MeCN	OBoc	n.r	n.d	n.d
6	THF	OTroc	86	>20:1	>99:1
7	THF	OAc	21	n.d	n.d
8	THF	$OCOCF_3$	27	>20:1	98:2
9	THF	OH	<10	n.d	n.d

All reactions were conducted with 0.2 mmol of **3a**, 0.24 mmol of **3** in 2 mL of solvent. [a] Reported as isolated yield. [b] Determined by crude ^{1}H NMR.

Conclusion: This reaction worked well in many commonly used solvents with high selectivity, and THF was the best. In addition, a good leaving group such as carbonate is necessary.

Table S2: Screening of boron sources and counter cation

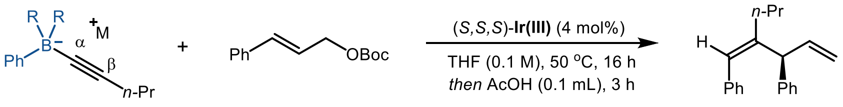

3, 1 equiv | **4a**, 1.2 equiv | (*R*,*Z*)-**5**

Entry	Boron sources	M⁺	Yield of **5**/%[a]	Z/E[b]	e.r.
1	Ph-BBN	Li	88	>20:1	>99:1
2	Ph_3B	Li	89	>20:1	>99:1
3	Ph-Bpin[c]	Li	n.d	-	-
4	$Ph\text{-}B(OH)_2$[c]	Li	n.d	-	-
5	Ph-BBN	NMe_4	89	>20:1	>99:1

All reactions were conducted with 0.2 mmol of **3a**, 0.24 mmol of **3** in 2 mL of solvent. [a] Reported as isolated yield. [b] Determined by crude ^{1}H NMR. [c] A small amount (<10%) of α-addition product was detected.

Conclusion: The use of borane as the boron source is key to achieve the desired transformation. Boronic acid and boronic ester were not suitable for this transformation, probably due to instability of the boronate complex, which was confirmed by ^{11}B NMR experiment (see below):

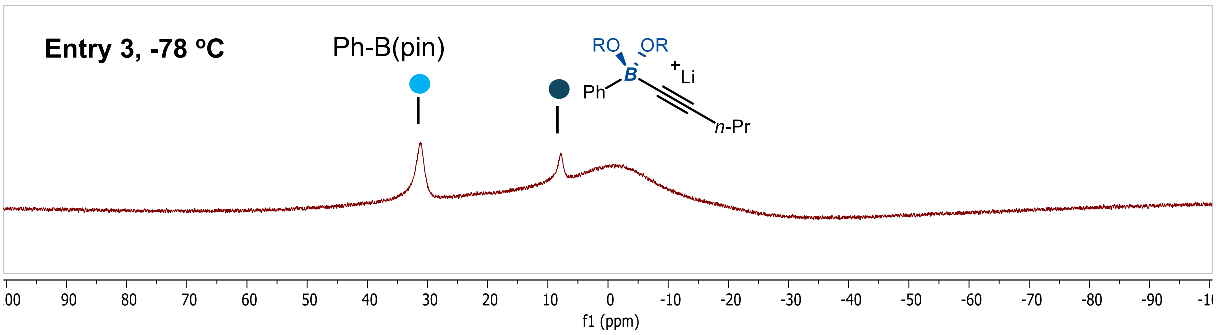

2.2.3 Unsuccessful examples

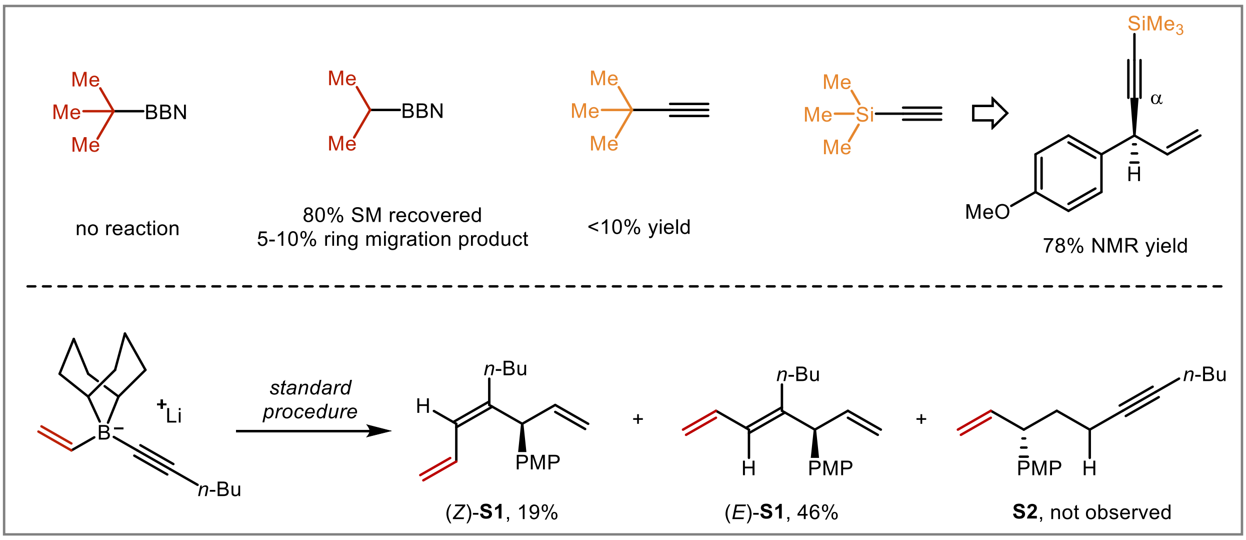

Figure S1: Summarized unsuccessful substrates

^{1}H NMR (400 MHz, CDCl$_3$) of the mixture of (*E*)-**S1** and (*Z*)-**S1**

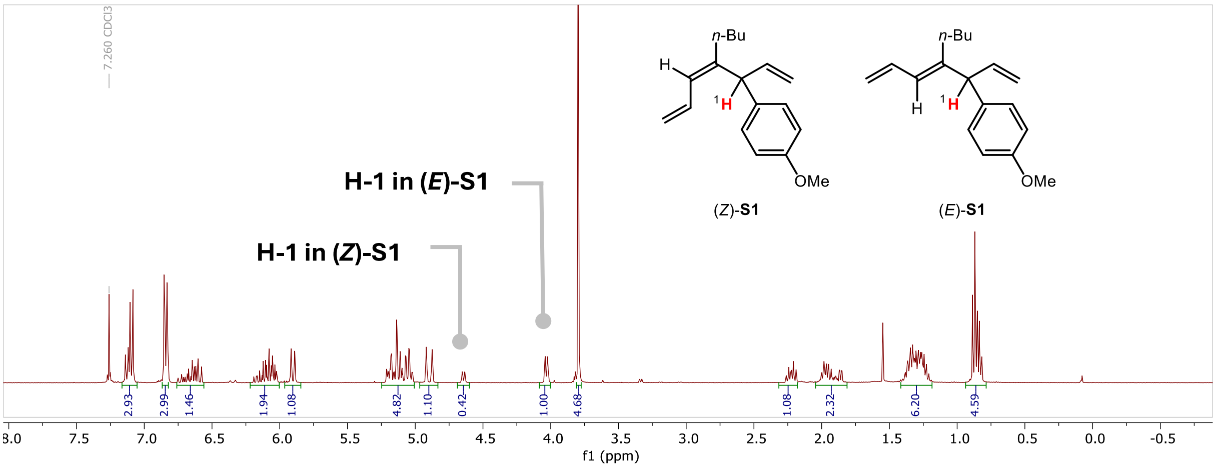

1D selective NOESY (500 MHz, CDCl$_3$) of the major isomer

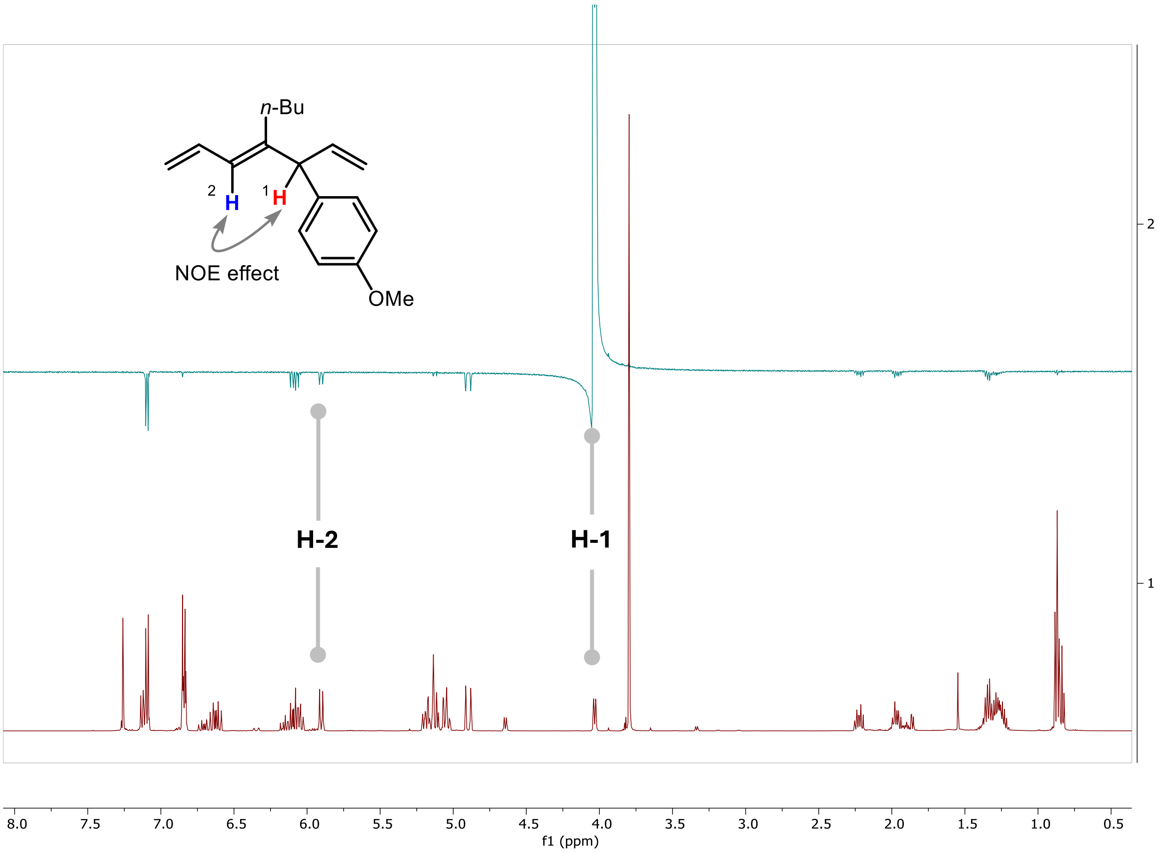

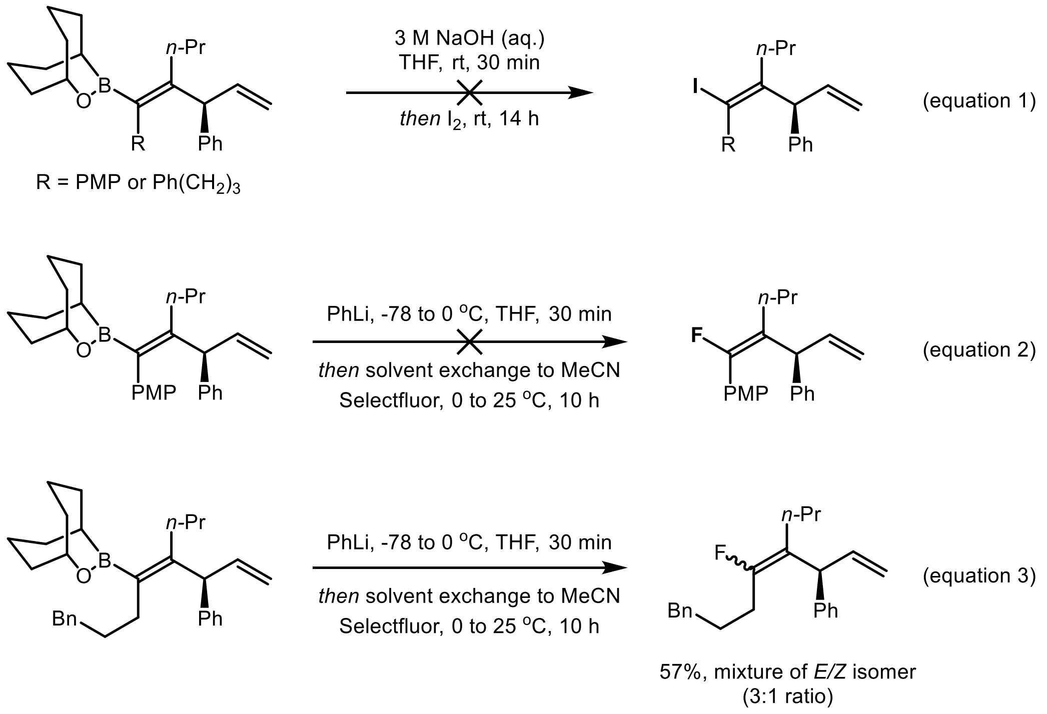

Figure S2: Attempts for other diversifications

2.3. General Procedures

2.3.1 General Procedure A for products 5-22 and 26-29

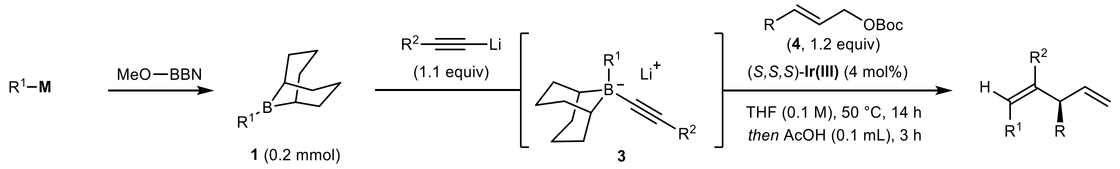

Step 1. Formation of alkynyl-ate complex: In a flame dried 8 mL Biotage® microwave reaction vial equipped with a magnetic stir bar was purged with nitrogen (N_2) three times. To this vial was added alkyne (0.22 mmol, 1.1 equiv) and anhydrous THF (1.0 mL) under nitrogen. The solution was cooled to -78 °C using a dry ice/acetone bath and *n*-BuLi (1.6 M in hexane, 0.22 mmol, 1.1 equiv; for **17**, 2.0 equiv of *n*-BuLi was used) was added dropwise. The reaction mixture was stirred for 30 minutes at this temperature, then borane (0.20 mmol, prepared from organometal reagent and *B*-methoxyl-BBN and purified via distillation) in dry THF (0.5 mL) was added dropwise via syringe. The mixture was allowed to warm to 25 °C and stirred for 1 hour at this temperature to ensure complete formation of alkynyl-ate complex. **Step 2. 1,2-Migration/allylation:** All solvents were carefully evaporated under reduce pressure, then 2.0 mL of anhydrous THF (c = 0.1 M), cinnamyl carbonate (0.24 mmol, 1.2 equiv) and Ir(III)

complex (8.0 mg, 0.008 mmol, 4 mol%) were sequentially added. After stirring at 50 °C for 14 hours, the reaction was cooled down to 25 °C and acetic acid (0.1 mL) was added. After stirring the mixture for 3 hours, the crude reaction mixture was concentrated under reduced pressure and purified by flash chromatography to give the desired product.

2.3.2 General Procedure B for products 23-25, 30 and 31

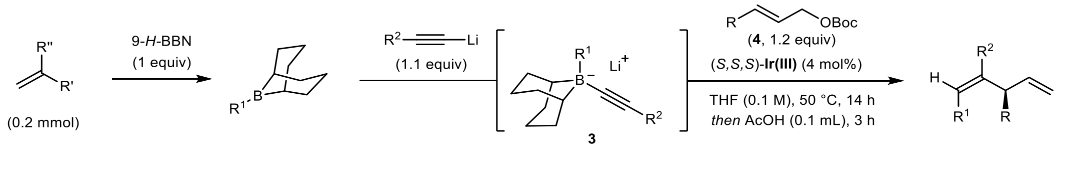

Step 1. Hydroboration: A flame dried 8 mL Biotage® microwave reaction **vial-1** equipped with a magnetic stir bar was purged with nitrogen (N_2) three times. To this vial was added alkene or alkyne (0.20 mmol, 1.0 equiv) under nitrogen. The solution was cooled to 0 °C using an ice bath and 9-*H*-BBN solution (0.5 M in THF, 0.2 mmol, 1.0 equiv) was slowly added. The mixture was allowed to warm to 25 °C and stirred for 3 hours at this temperature to ensure complete hydroboration. **Step 2. Formation of alkynyl-ate complex:** In a separated, flame dried 8 mL Biotage® microwave reaction **vial-2** equipped with a magnetic stir bar was purged with nitrogen (N_2) three times. To this vial was added alkyne (0.22 mmol, 1.1 equiv) and anhydrous THF (1.0 mL) under nitrogen. The solution was cooled to -78 °C using a dry ice/acetone bath and *n*-BuLi (1.6 M in hexane, 0.22 mmol, 1.1 equiv) was added dropwise. The reaction mixture was stirred for 30 minutes at this temperature, then borane in **vial-1** was carefully transferred to **vial-2** via syringe. The mixture was allowed to warm to 25 °C and stirred for 1 hour at this temperature to ensure complete formation of alkynyl ate complex. **Step 3. 1,2-Migration/allylation:** All solvents were carefully evaporated under reduce pressure, then 2.0 mL of anhydrous THF (c = 0.1 M), cinnamyl carbonate (0.24 mmol, 1.2 equiv) and Ir(III) complex (8.0 mg, 0.008 mmol, 4 mol%) were sequentially added. After stirring at 50 °C for 14 hours, the reaction was cooled down to 25 °C and acetic acid (0.1 mL) was added. After stirring the mixture for 3 hours, the crude reaction mixture was concentrated under reduced pressure and purified by flash chromatography to give the desired product.

2.3.3 General Procedure C for products 32-38 and 42-44

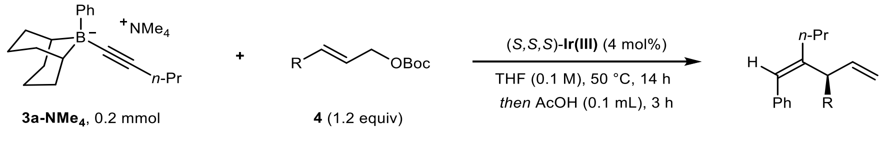

A flame dried 7 mL vial equipped with a magnetic stir bar was charged with **3a-NMe4** (68 mg, 0.20 mmol, 1.0 equiv) under N_2 atmosphere. To this vial was transferred into an anhydrous, argon-filled glovebox where 2.0 mL of anhydrous THF (c = 0.1 M), cinnamyl carbonate (0.24 mmol, 1.2 equiv) and Ir(III) complex (8.0 mg, 0.008 mmol, 4 mol%) were sequentially added. The vial was sealed with a cap with septum, removed from the glovebox. After stirring at 50 °C for 14 hours, the reaction was cooled down to 25 °C and acetic acid (0.1 mL) was added. After stirring the mixture for 3 hours, the crude reaction mixture was concentrated under reduced pressure and purified by flash chromatography to give the desired product.

2.3.4 General Procedure D for products 39-41

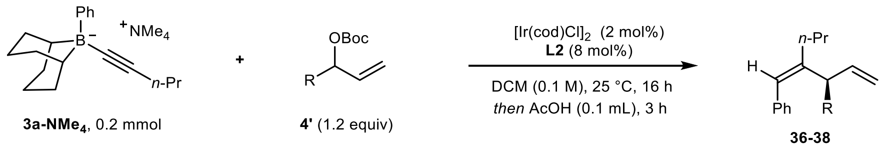

A flame dried 7 mL vial equipped with a magnetic stir bar was charged with $[Ir(cod)Cl]_2$ (2 mol%) and **L2** (8 mol%) and was purged with nitrogen (N_2) three times. 1.0 mL of DCM (anhydrous and degassed) was added under nitrogen and the mixture was stirred at 25 °C for 30 minutes. To this solution was added **3a-NMe4** (68 mg, 0.20 mmol, 1.0 equiv), cinnamyl carbonate (0.24 mmol, 1.2 equiv) and DCM (1.0 mL) under N_2 atmosphere. After stirring at 25 °C for 16 hours, the reaction quenched by adding acetic acid (0.1 mL) and stirring for further 3 hours. The crude reaction mixture was concentrated under reduced pressure and purified by flash chromatography to give the desired product.

2.3.5 General Procedure E for hydroboration/oxidation of products for ee determination

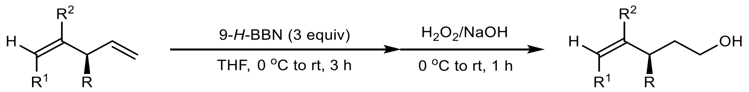

In some cases, the obtained racemic skipped diene was unable to separate well by chiral HPLC column. Therefore hydroboration/oxidation was conducted to convert diene to more polarized alcohol.

A flame dried 8 mL Biotages® microwave reaction vial equipped with a magnetic stir bar was charged with skipped diene (0.10 mmol, 1.0 equiv). 9-*H*-BBN (0.5 M in THF, 0.6 mL, 0.30 mmol, 3 equiv) was added slowly to this vial at 0 °C in 5 minutes. The reaction was then allowed to warm up to room temperature and stirred for a further 3 hours before cooling back to 0 °C. 3 M NaOH (0.3 mL) and 30% H_2O_2 were sequentially added. The reaction mixture was stirred for 30 minutes at 0 °C and 30 minutes at room temperature to complete the oxidation.

After quenching the excess hydroperoxide with saturated $Na_2S_2O_3$, the mixture was diluted with brine and extracted with Et_2O (3 x 5 mL). The organic phase was dried over anhydrous $MgSO_4$ and concentrated in vacuo. Purification of the colorless oil residue by silica gel chromatography (pentane/EtOAc, 10:1) affords alcohol as a colorless oil.

2.4. Procedures for Transformation of Products

2.4.1 Procedures for *in-situ* TMANO oxidation of borane to borinic ester

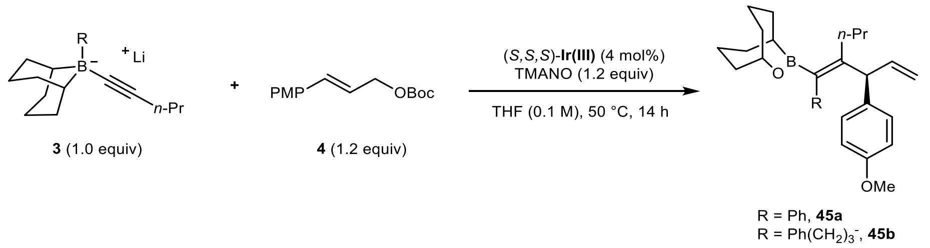

A flame dried 8 mL Biotage® microwave reaction vial equipped with a magnetic stir bar was transferred into an anhydrous, argon-filled glovebox where **3** (0.20 mmol), 2.0 mL of anhydrous THF (c = 0.1 M), cinnamyl carbonate (1.2 equiv), trimethylammonium *N*-oxide (TMANO, 1.2 equiv) and Ir(III) complex (4 mol%) were sequentially added. The vial was sealed with a cap with septum, removed from the glovebox. After stirring at 50 °C for 14 hours, the reaction was cooled down to 25 °C. The reaction mixture was concentrated under reduced pressure to give crude **45**, which were used directly for downstream transformations. Borinic ester **45a** could be purified by flash chromatography to give pure product.

Note: 1**)** The borinic esters were unstable and would decompose using silica gel for flash column chromatography, giving a mixture of borinic ester and protodeboronation product which are difficult to separate. Fast flash column chromatography (less than 10 min) using a short column (aluminum oxide neutral, 1.5 cm diameter and 7 cm length, for 0.5 mmol scale) is recommended.

2) We found that trisubstituted alkenyl borinic esters that derived from aryl-BBN are more stable than that from alkyl-BBN. Attempts to isolate borinic ester **45b** resulted in considerably loss of material.

3) Borinic esters were also found to be slightly unstable in $CDCl_3$, probably due to the presence of a trace amount of HCl in $CDCl_3$. To avoid decomposition that leads to impure NMR spectra, K_2CO_3-neutralized $CDCl_3$ or other non-acidic deuterated solvents (e.g. CD_2Cl_2, *d8*-toluene) are recommended.

2.4.2 *sp*^{2}-*sp*^{2} and *sp*^{2}-*sp*^{3} cross coupling of 45[5,6]

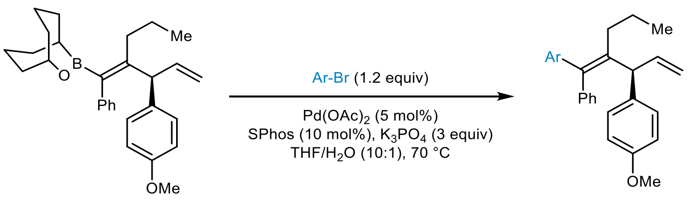

45a **46, 47**

A 25 mL Schlenk flask equipped with a magnetic stir bar was charged with $Pd(OAc)_2$ (0.01 mmol, 5 mol%), SPhos (0.02 mmol, 10 mol%) and K_3PO_4 (0.60 mmol, 3.0 equiv) then purged with nitrogen (N_2) three times. To this flask was sequentially added crude **45a** (0.20 mmol scale from **2.4.1**, 1.0 equiv) in THF (degassed, 2.0 mL), H_2O (degassed, 0.2 mL) and arylbromide (0.24 mmol, 1.2 equiv). The mixture was stirred for overnight at 70 °C before cooling to ambient temperature. H_2O (5 mL) and Et_2O (5 mL) were added to the mixture. The organic layer was separated, and the aqueous layer was extracted with Et_2O (3 x 5 mL). The combined organic layers were dried over anhydrous $MgSO_4$, filtered and concentrated under reduced pressure. Purification of the yellow oil residue by silica gel chromatography afforded desired *sp*^{2}-*sp*^{2} coupling products **46** and **47**.

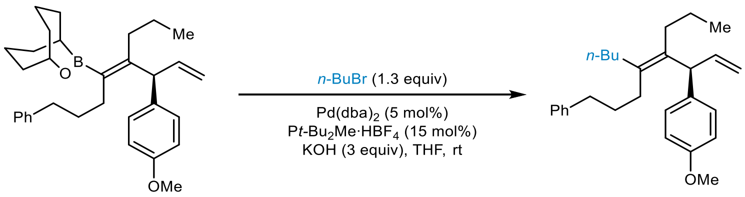

45b **48**

A 25 mL Schlenk flask equipped with a magnetic stir bar was charged with $Pd(dba)_2$ (0.01 mmol, 5 mol%), *t*-$Bu_2MeP{\cdot}HBF_4$ (0.03 mmol, 15 mol%) and KOH (0.60 mmol, 3.0 equiv) then purged with nitrogen (N_2) three times. To this flask was sequentially added **45b** (0.20 mmol scale from **2.4.1**, 1.0 equiv) in anhydrous THF (degassed, 1.0 mL) and *n*-butylbromide (0.26 mmol, 1.3 equiv). The mixture was then stirred for 24 hours at ambient temperature. After evaporating all volatile materials, the residue was directly purified by silica gel chromatography to afford desired *sp*^{2}-*sp*^{3} coupling product **48**.

2.4.3 Zweifel-type alkynylation[7]

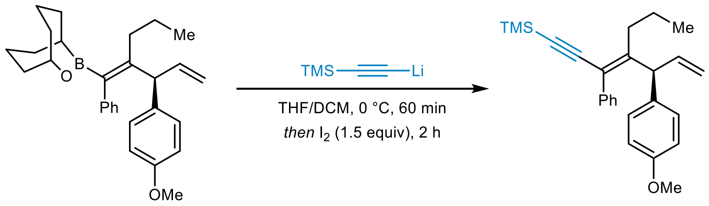

45a **49**

Prepared following a literature procedure[7] with modifications. A solution of trimethylsilylacetylene (0.30 mmol, 1.5 equiv) in anhydrous THF (1.0 mL) was treated with *n*-BuLi (1.6 M in hexane, 0.30 mmol, 1.5 equiv) at -78 °C and stirred at this temperature for 45 minutes. Then a solution of **45a** (0.20 mmol scale from **2.4.1**, 1.0 equiv) in THF (1.0 mL) was added dropwise to the reaction mixture, and the mixture was allowed to warm to ambient temperature. After stirring for 1 hour, the reaction was cooled to -78 °C, and a solution of I_2 (0.30 mmol) in THF/DCM (1:1, 2.0 mL) was added dropwise. The solution was stirred at -78 °C for an additional 20 minutes and aqueous NaOH (1 M, 1.0 mL) was added followed by 30% H_2O_2 (0.3 mL). The cold bath was removed, and the mixture was warmed to ambient temperature then diluted with H_2O/DCM (10 mL 1:1). The organic layer was washed with brine, dried over $MgSO_4$ and concentrated in vacuo. Purification of the oil residue by silica gel chromatography affords **49** as a colorless oil.

2.4.4 Transmetallation/electrophilic trapping of boronate intermediate[8]

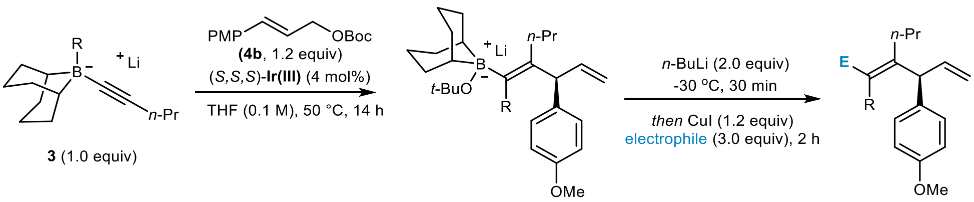

3 (1.0 equiv)

Int I, R = Ph
Int II, R = PMP

51, 52

The reaction mixture from **General Procedure A** was cooled down to -30 °C before dropwise addition of *n*-BuLi (2.5 M in hexane, 0.4 mmol, 2.0 equiv). After stirring at the same temperature for 30 minutes, copper(I) iodide (0.24 mmol, 1.2 equiv) was added to the red/orange solution in one portion, quickly resulting in a brown to black solution. Then, methyl iodide or allyl bromide (0.6 mmol, 3.0 equiv) was added dropwise via syringe and the cold bath was removed. The reaction was stirred for 2 hours followed by sequential addition of aqueous NaOH (3 M, 0.3 mL) and 30% H_2O_2 (0.2 mL) at 0 °C. After stirring for 30 minutes, a saturated aqueous solution of $Na_2S_2O_3$ (1.0 mL) was added to destroy excess H_2O_2. The

aqueous layer was washed with Et_2O (3 × 5 mL), and the combined organic layers were dried over $MgSO_4$ and concentrated in vacuo. Purification of the yellow oil residue by silica gel chromatography affords **51** and **52** as a colorless oil.

2.5. Determination of Stereochemistry

2.5.1 Stereochemistry of 5

1D selective NOESY (500 MHz, $CDCl_3$) of **5**

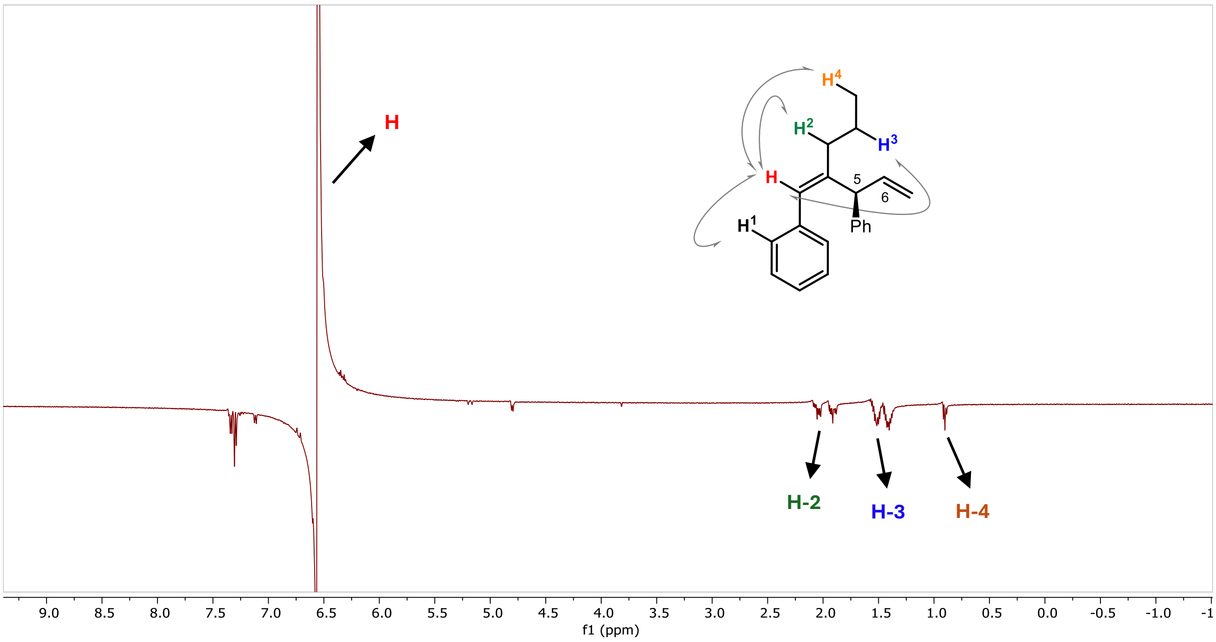

Conclusion: The stereochemistry of compound **5** was determined to be (*Z*)-geometry through 1D selective NOESY NMR experiment. In the NMR spectra that shown above, the noe effect of **H-H2, H-H3** and **H-H4** are clearly observed. Meanwhile, no noe effect of **H-H5** or **H1-H6** are observed. These results indicates that (*Z*)-**5** was obtained from our methodology.

2.5.2 Stereochemistry of 49

^{1}H NMR (400 MHz, $CDCl_3$) of **46**

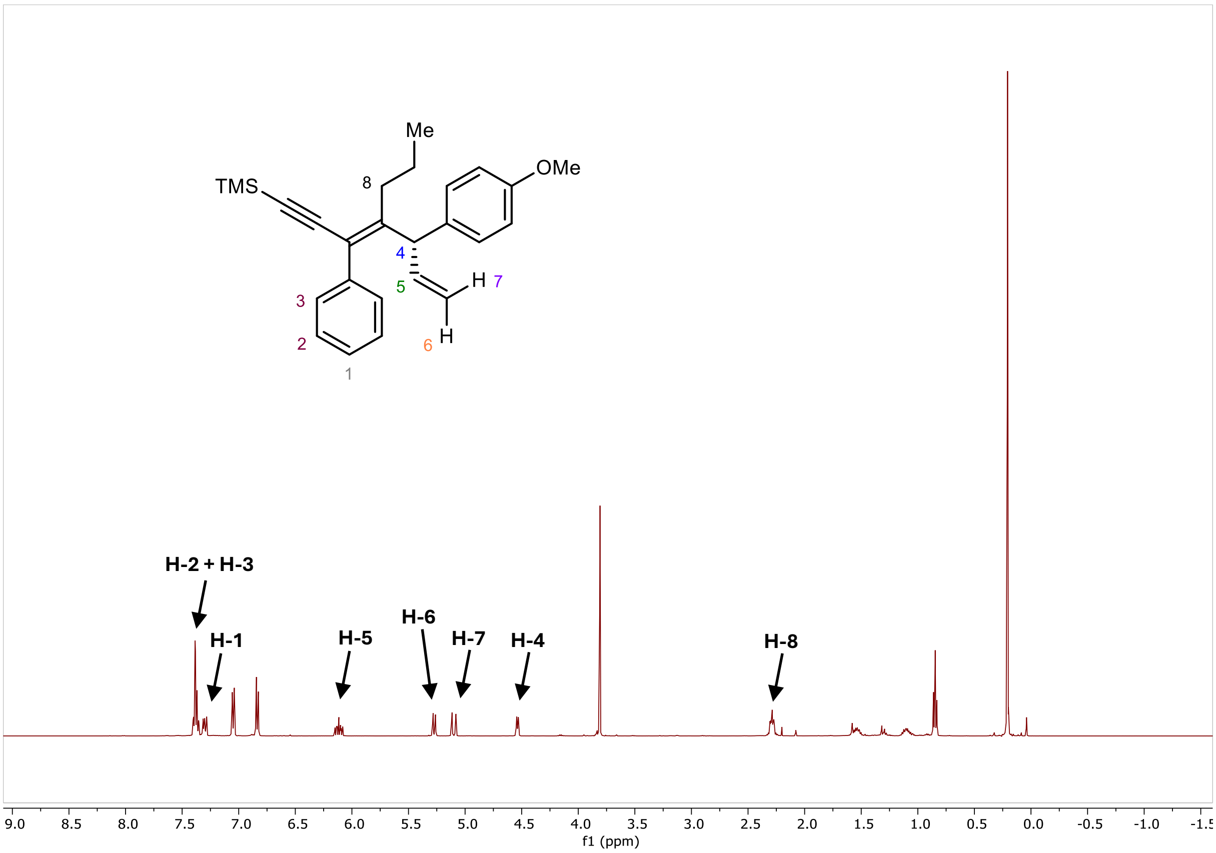

2D NOESY (400 MHz, $CDCl_3$) of **49**

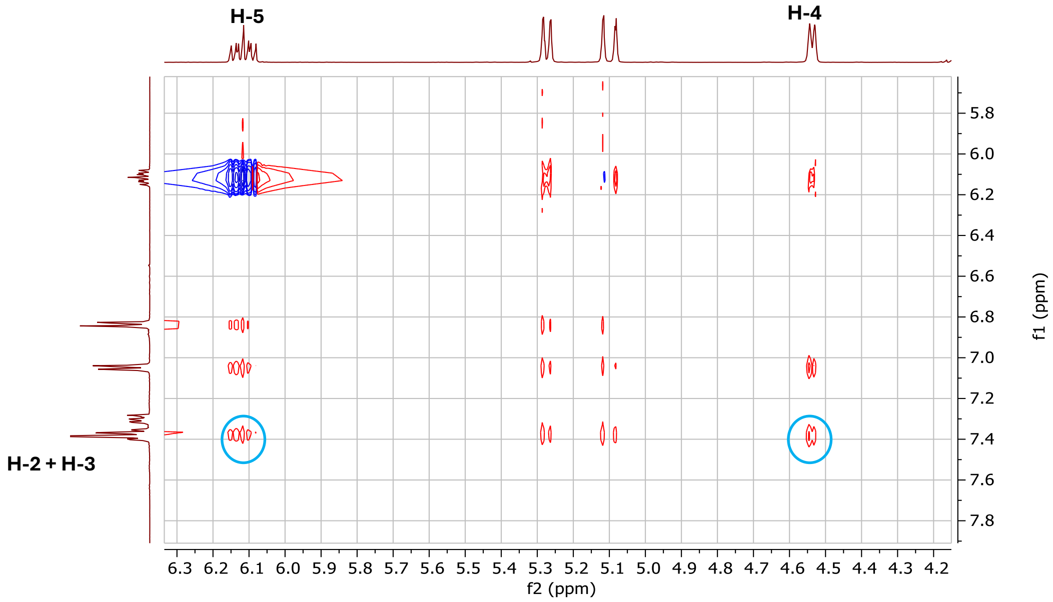

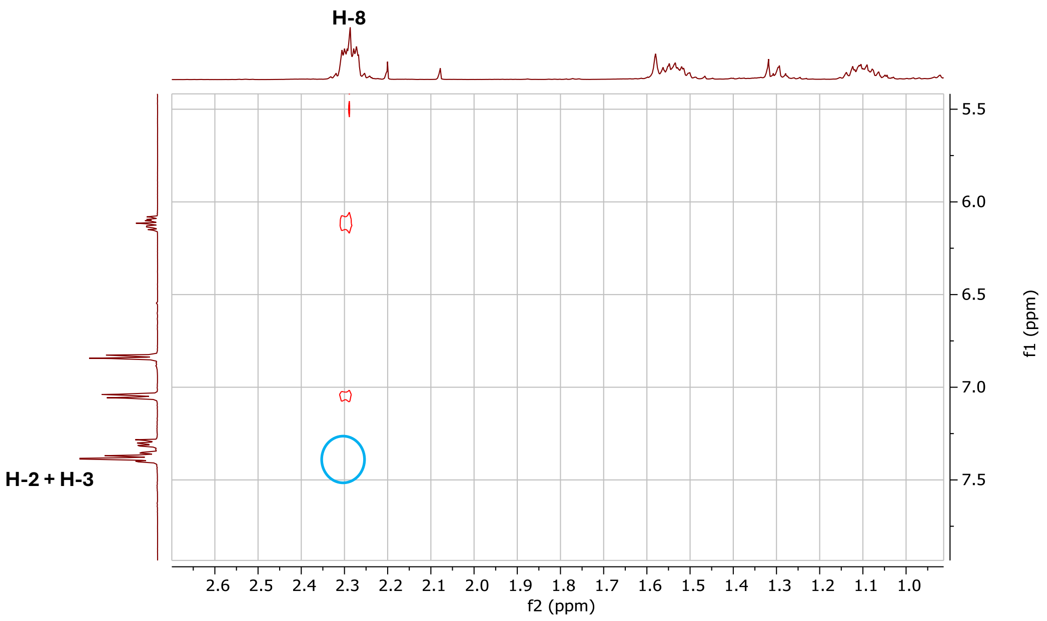

<u>Conclusion:</u> The stereochemistry of compound **49** was determined to be (*E*)-geometry through NOESY NMR analysis. In the NMR spectra that shown above, the noe effect of **H5-H(2+3)** and **H4-H(2+3)** are clearly observed. Meanwhile, no noe effect of **H8-H(2+3)** are observed. These results indicate that phenyl group and allyl group locate *syn* across the alkene, therefore the Zweifel-type alkynylation occurred in a stereoretentive manner.

2.5.3 Stereochemistry of 52

1D selective NOESY (500 MHz, $CDCl_3$) of **52**

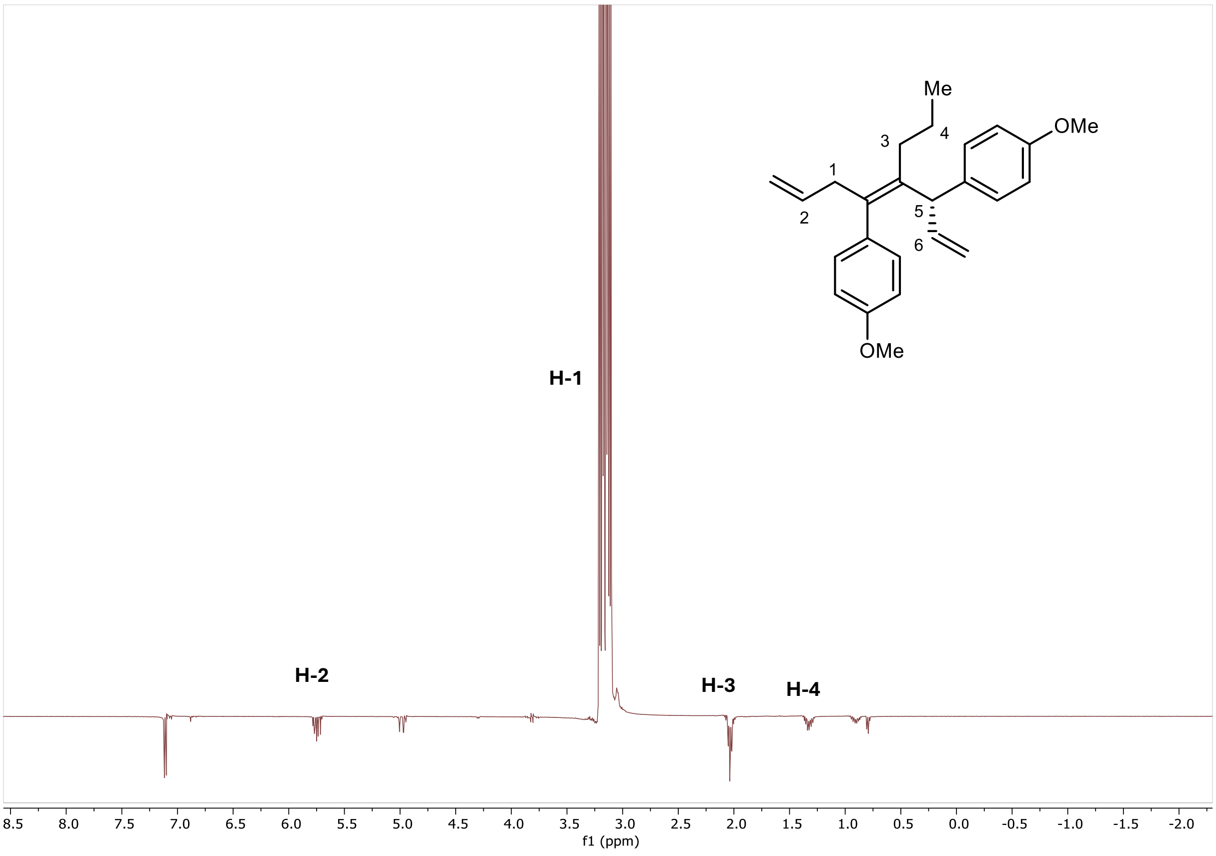

Conclusion: The stereochemistry of compound **52** was determined to be (*Z*)-geometry through 1D selective NOESY NMR analysis. In the NMR spectra that shown above, the noe effect of **H1-H3** and **H1-H4** are clearly observed. Meanwhile, no noe effect of **H1-H5** or **H1-H6** are observed. These results indicate that allyl group and *n*-propyl group locate *syn* across the alkene, therefore the transmetallation/electrophilic trapping occurred in a stereoretentive manner.

2.6. Proposed Reaction Mechanism

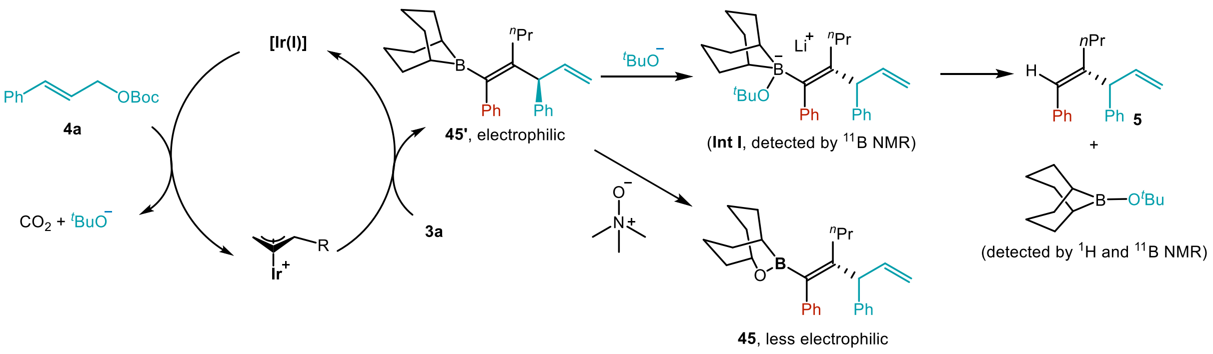

2.7. Characterization Data for Products.

(*R*,*Z*)-(2-propylpenta-1,4-diene-1,3-diyl)dibenzene (5)

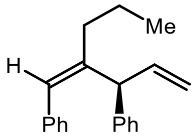

Prepared following **General Procedure A**. Purification by flash column chromatography (silica gel, 5-10% DCM in pentane) gave the title compound (46.1 mg, 88%, >20:1 *Z*/*E*, >99:1 e.r.) as a colorless oil.

R_f = 0.60 (20:1 pentane: EtOAc, UV)

^{1}H NMR (400 MHz, $CDCl_3$) δ 7.35 – 7.26 (m, 6H), 7.24 – 7.18 (m, 4H), 6.55 (s, 1H), 6.21 (ddd, J = 17.2, 10.4, 6.8 Hz, 1H), 5.30 (ddd, J = 10.4, 1.6 Hz, 1H), 5.18 (ddd, J = 17.2, 1.6 Hz, 1H), 4.84 (d, J = 6.8 Hz, 1H), 2.04 (dddd, J = 16.0, 10.4, 5.6, 1.6 Hz, 1H), 1.89 (dddd, J = 16.0, 10.4, 5.6, 1.6 Hz, 1H), 1.53 – 1.33 (m, 2H), 0.87 (t, J = 7.2 Hz, 3H).

^{13}C NMR (101 MHz, $CDCl_3$) δ 143.05, 142.52, 138.75, 138.46, 128.69, 128.31, 128.27, 126.51, 126.42, 126.29, 117.11, 50.25, 34.65, 21.91, 14.28.

HRMS (EI) m/z calculated for $C_{20}H_{22}$ [M], 262.1722, found: 262.1715.

Specific rotation $[\alpha]_D^{24}$ = -33.1 (c = 0.65, CH_2Cl_2).

HPLC conditions: Chiral column IC, hexane: isopropanol = 99:1, flow rate = 1.0 mL/min, wavelength = 254 nm, t_R = 5.3 min for major isomer, t_R = 5.7 min for major isomer.

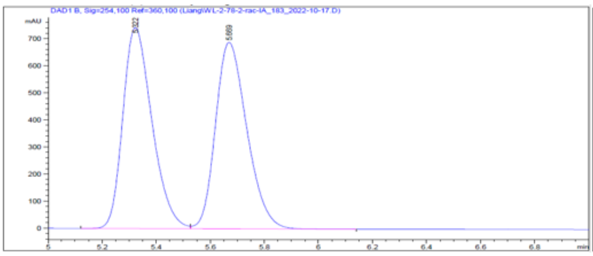

Peak #	RetTime [min]	Sig	Type	Area [mAU*s]	Height [mAU]	Area %
1	5.322	1	BV	5567.73633	742.89410	50.0398
2	5.669	1	VB	5558.87744	689.46625	49.9602

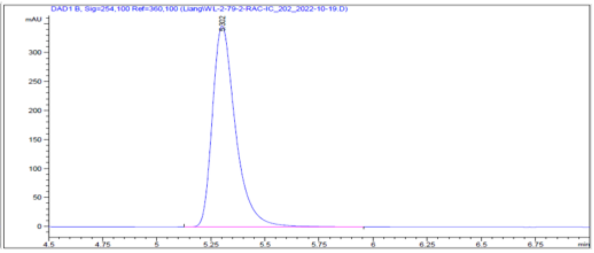

Peak #	RetTime [min]	Sig	Type	Area [mAU*s]	Height [mAU]	Area %
1	5.302	1	BB	2571.46582	346.87204	100.0000

<u>(*R*,*Z*)-(2-isobutylpenta-1,4-diene-1,3-diyl)dibenzene (6)</u>

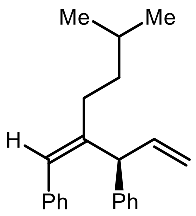

Prepared following **General Procedure A**. Purification by flash column chromatography (silica gel, 5-10% DCM in pentane) gave the title compound (45.8 mg, 83%, >20:1 *Z*/*E*, 98:2 e.r.) as a colorless oil.

R*f* = 0.60 (20:1 pentane: EtOAc, UV)

^{1}H NMR (400 MHz, $CDCl_3$) δ 7.31 – 7.06 (m, 10H), 6.46 (s, 1H), 6.13 (ddd, *J* = 17.2, 10.4, 6.4 Hz, 1H), 5.22 (ddd, *J* = 10.4, 1.6 Hz, 1H), 5.09 (ddd, *J* = 17.2, 1.6 Hz, 1H), 4.76 (d, *J* = 6.8 Hz, 1H), 1.98 (dddd, *J* = 15.6, 11.2, 5.2, 1.6 Hz, 1H), 1.82 (dddd, J = 15.6, 11.2, 5.2, 1.6 Hz, 1H), 1.42 – 1.21 (m, 2H), 1.20 – 1.03 (m, 1H), 0.80 – 0.63 (m, 6H).

^{13}C NMR (101 MHz, $CDCl_3$) δ 143.56, 142.50, 138.80, 138.49, 128.68, 128.31, 126.57, 126.41, 126.30, 117.10, 50.40, 38.34, 30.31, 28.23, 22.73, 22.72.

HRMS (EI) m/z calculated for $C_{22}H_{26}$ [M], 290.2035, found: 290.2025.

Specific rotation $[\alpha]_D^{24}$ = -22.5 (c = 0.6, CH_2Cl_2).

HPLC conditions: Chiral column AD-H, hexane: isopropanol = 99:1, flow rate = 1.0 mL/min, wavelength = 254 nm, t_R = 4.0 min for major isomer, t_R = 4.2 min for major isomer.

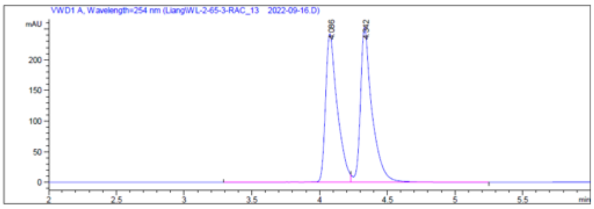

Peak #	RetTime [min]	Type	Width [min]	Area [mAU*s]	Height [mAU]	Area %
1	4.086	BV	0.1032	1459.35596	229.87448	49.1500
2	4.342	VB	0.1011	1509.83105	244.82147	50.8500

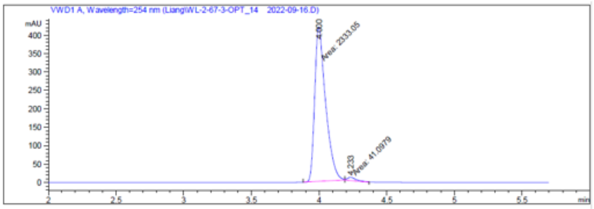

Peak #	RetTime [min]	Type	Width [min]	Area [mAU*s]	Height [mAU]	Area %
1	4.000	MM	0.0923	2333.04663	421.23218	98.2689
2	4.233	MM	0.0781	41.09789	8.77375	1.7311

<u>(*R*,*Z*)-(2-benzylpenta-1,4-diene-1,3-diyl)dibenzene (7)</u>

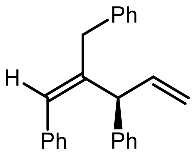

Prepared following **General Procedure A**. Purification by flash column chromatography (silica gel, 5-13% DCM in pentane) gave the title compound (39.7 mg, 64%, >20:1 *Z*/*E*, 99:1 e.r.) as a colorless oil.

R*f* = 0.50 (20:1 pentane: EtOAc, UV)

^{1}H NMR (400 MHz, $CDCl_3$) δ 7.35 – 7.16 (m, 13H), 7.15 – 7.09 (m, 2H), 6.30 – 6.16 (m, 2H), 5.34 (ddd, *J* = 10.4, 1.6 Hz, 1H), 5.26 (ddd, *J* = 17.2, 1.6 Hz, 1H), 4.93 (d, *J* = 6.8 Hz, 1H), 3.39 (dd, *J* = 16.4, 1.6 Hz, 1H), 3.17 (dd, *J* = 16.4, 1.6 Hz, 1H).

^{13}C NMR (101 MHz, $CDCl_3$) δ 143.07, 142.27, 139.80, 138.42, 138.14, 129.98, 129.45, 128.59, 128.48, 128.39, 128.33, 128.28, 126.60, 126.49, 126.14, 117.44, 50.16, 38.89.

HRMS (EI) m/z calculated for $C_{24}H_{22}$ [M], 310.1722, found: 310.1714.

Specific rotation $[\alpha]_D^{24}$ = -19.2 (c = 0.65, CH_2Cl_2).

HPLC conditions: Chiral column OJ, hexane: isopropanol = 99:1, flow rate = 0.7 mL/min, wavelength = 254 nm, t_R = 7.9 min for major isomer, t_R = 9.2 min for minor isomer.

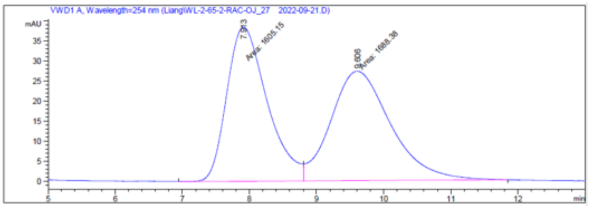

Peak #	RetTime [min]	Type	Width [min]	Area [mAU*s]	Height [mAU]	Area %
1	7.913	MF	0.6965	1605.14990	38.41098	48.7364
2	9.606	FM	1.0321	1688.38110	27.26450	51.2636

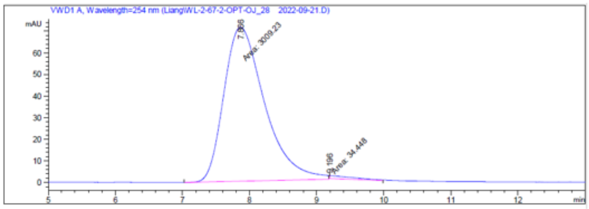

Peak #	RetTime [min]	Type	Width [min]	Area [mAU*s]	Height [mAU]	Area %
1	7.866	MM	0.7038	3009.23364	71.26051	98.8682
2	9.196	MM	0.3127	34.44802	1.31563	1.1318

<u>(*R*,*Z*)-(2-(1-phenylallyl)pent-1-ene-1,5-diyl)dibenzene (8)</u>

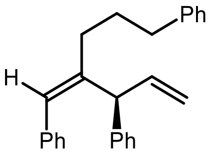

Prepared following **General Procedure A**. Purification by flash column chromatography (silica gel, 5-12% DCM in pentane) gave the title compound (58.8 mg, 87%, >20:1 *Z*/*E*, 97:3 e.r.) as a colorless oil.

R*f* = 0.55 (20:1 pentane: EtOAc, UV)

^{1}H NMR (400 MHz, $CDCl_3$) δ 7.30 – 6.98 (m, 15H), 6.48 (s, 1H), 6.11 (ddd, *J* = 17.2, 10.0, 6.8 Hz, 1H), 5.21 (ddd, *J* = 10.0, 1.6 Hz, 1H), 5.09 (ddd, *J* = 17.2, 1.6 Hz, 1H), 4.76 (d, *J* = 6.8 Hz, 1H), 2.46 (t, *J* = 7.6 Hz, 2H), 2.06 (dddd, *J* = 16.0, 10.4, 5.2, 1.6 Hz, 1H), 1.90 (dddd, *J* = 15.6, 10.4, 5.2, 1.6 Hz, 1H), 1.80 – 1.62 (m, 1H), 1.62 – 1.49 (m, 1H).

^{13}C NMR (101 MHz, $CDCl_3$) δ 142.80, 142.59, 142.40, 138.65, 138.30, 128.67, 128.49, 128.39, 128.37, 128.34, 128.30, 126.88, 126.50, 126.37, 125.81, 117.19, 50.25, 36.05, 32.32, 30.90.

HRMS (EI) m/z calculated for $C_{26}H_{26}$ [M], 338.2035, found: 338.2026.

Specific rotation $[\alpha]_D^{24}$ = -33.0 (c = 0.95, CH_2Cl_2).

HPLC conditions: Chiral column IA, hexane: isopropanol = 98:2, flow rate = 1.0 mL/min, wavelength = 254 nm, t_R = 5.6 min for major isomer, t_R = 5.3 min for minor isomer.

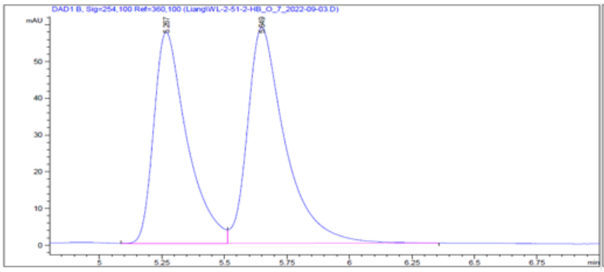

Peak #	RetTime [min]	Sig	Type	Area [mAU*s]	Height [mAU]	Area %
1	5.267	1	BV	531.01801	57.57842	46.3761
2	5.649	1	VB	614.00824	58.74664	53.6239

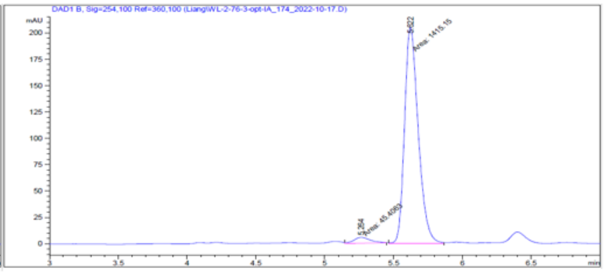

Peak #	RetTime [min]	Sig	Type	Area [mAU*s]	Height [mAU]	Area %
1	5.264	1	MM	45.40633	5.49481	3.1088
2	5.622	1	MM	1415.14502	204.71243	96.8912

<u>(*S*,*Z*)-(2-cyclopropylpenta-1,4-diene-1,3-diyl)dibenzene (9)</u>

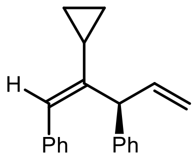

Prepared following **General Procedure A**. Purification by flash column chromatography (silica gel, 5-10% DCM in pentane) gave the title compound (44.0 mg, 77%, >20:1 *Z*/*E*, >99:1 e.r.) as a colorless oil.

R$_f$ = 0.60 (20:1 pentane: EtOAc, UV)

^{1}H NMR (400 MHz, $CDCl_3$) δ 7.29 – 7.07 (m, 10H), 6.30 (ddd, J = 17.2, 10.4, 7.2 Hz, 1H), 6.19 (s, 1H), 5.22 (ddd, J = 10.4, 1.6, 1.2 Hz, 1H), 5.13 (ddd, J = 17.2, 1.6 Hz, 1H), 4.83 (d, J = 7.2 Hz, 1H), 1.27 – 1.10 (m, 1H), 0.75 – 0.58 (m, 1H), 0.53 – 0.37 (m, 3H).

^{13}C NMR (101 MHz, $CDCl_3$) δ 144.95, 142.72, 139.09, 138.14, 128.84, 128.35, 128.32, 128.28, 126.43, 126.25, 122.36, 116.93, 50.88, 13.96, 9.14, 7.03.

HRMS (EI) m/z calculated for $C_{20}H_{20}$ [M], 260.1565, found: 260.1559.

Specific rotation $[\alpha]_D^{24}$ = -10.8 (c = 0.70, CH_2Cl_2).

HPLC conditions: Chiral column IA, hexane: isopropanol = 98:2, flow rate = 1.0 mL/min, wavelength = 254 nm, t_R = 5.2 min for major isomer.

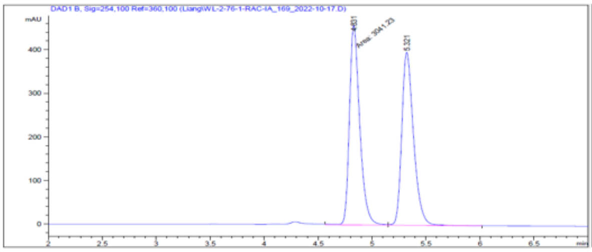

Peak #	RetTime [min]	Sig	Type	Area [mAU*s]	Height [mAU]	Area %
1	4.831	1	FM	3041.22534	457.08478	50.2816
2	5.321	1	VB	3007.15967	396.75787	49.7184

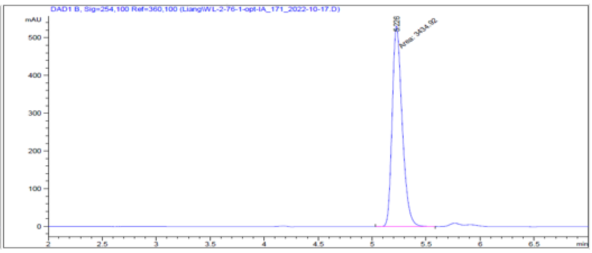

Peak #	RetTime [min]	Sig	Type	Area [mAU*s]	Height [mAU]	Area %
1	5.226	1	MM	3434.91577	531.60345	100.0000

<u>**(*S*,*Z*)-1-(4-benzylidene-5-methylhex-1-en-3-yl)-4-methoxybenzene (10)**</u>

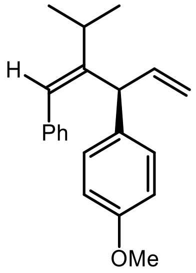

Prepared following **General Procedure A**. Purification by flash column chromatography (silica gel, 5-15% DCM in pentane) gave the title compound (30.4 mg, 52%, >20:1 *Z*/*E*, 97:3 e.r.) as a colorless oil.

R$_f$ = 0.50 (20:1 pentane: EtOAc, UV)

^{1}H NMR (400 MHz, $CDCl_3$) δ 7.36 – 7.26 (m, 4H), 7.23 – 7.18 (m, 1H), 7.10 (d, J = 8.8 Hz, 2H), 6.81 (d, J = 8.8 Hz, 2H), 6.57 (s, 1H), 6.19 (ddd, J = 17.2, 10.4, 6.8 Hz, 1H), 5.28 (ddd, J = 10.4, 1.6 Hz, 1H), 5.18 (ddd, J = 17.2, 1.6 Hz, 1H), 4.82 (d, J = 6.8 Hz, 1H), 3.78 (s, 3H), 2.33 – 2.18 (m, 1H), 1.16 (d, J = 6.8 Hz, 3H), 0.81 (d, J = 6.8 Hz, 3H).

^{13}C NMR (101 MHz, $CDCl_3$) δ 158.19, 150.52, 139.15, 138.52, 134.52, 129.39, 128.70, 128.27, 126.34, 125.10, 116.90, 113.63, 55.37, 50.12, 30.17, 25.23, 24.90.

HRMS (EI) m/z calculated for $C_{21}H_{24}O$ [M], 292.1827, found: 292.1818.

Specific rotation $[\alpha]_D^{24}$ = -21.8 (c = 0.40, CH_2Cl_2).

HPLC conditions: Chiral column IA, hexane: isopropanol = 98:2, flow rate = 1.0 mL/min, wavelength = 254 nm, t_R = 4.7 min for major isomer, t_R = 4.9 min for minor isomer.

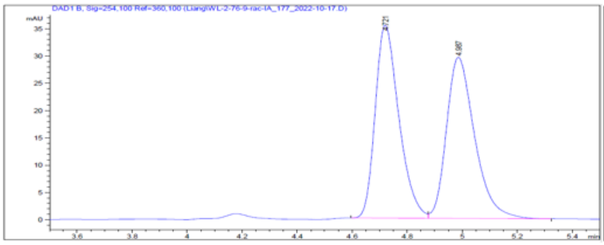

Peak #	RetTime [min]	Sig	Type	Area [mAU*s]	Height [mAU]	Area %
1	4.721	1	BV	211.66829	35.35848	50.1593
2	4.987	1	VB	210.32358	29.52109	49.8407

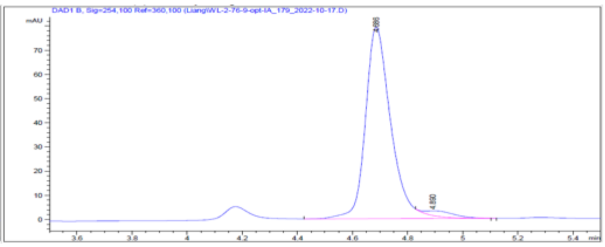

Peak #	RetTime [min]	Sig	Type	Area [mAU*s]	Height [mAU]	Area %
1	4.686	1	BV R	508.22058	79.24549	97.0068
2	4.890	1	VB E	15.68165	1.88457	2.9932

<u>**(*R*,*Z*)-1-methoxy-4-(2-methyl-1-phenylpenta-1,4-dien-3-yl)benzene (11)**</u>

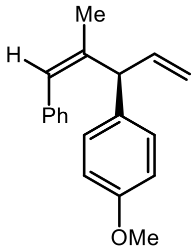

Prepared following **General Procedure A**. Purification by flash column chromatography (silica gel, 5-13% DCM in pentane) gave the title compound (33.4 mg, 63%, 17:1 *Z*/*E*, 98:2 e.r.) as a colorless oil.

R_f = 0.50 (20:1 pentane: EtOAc, UV)

^{1}H NMR (400 MHz, $CDCl_3$) δ 7.26 – 7.18 (m, 4H), 7.16 – 7.12 (m, 1H), 7.01 (d, J = 8.4 Hz, 2H), 6.75 (d, J = 8.4 Hz, 2H), 6.43 (s, 1H), 6.08 (ddd, J = 17.2, 10.4, 6.4 Hz, 1H), 5.21 (ddd, J

= 10.4, 1.6 Hz, 1H), 5.10 (ddd, J = 17.2, 1.6 Hz, 1H), 4.66 (d, J = 6.4 Hz, 1H), 3.71 (s, 3H), 1.62 (d, J = 1.6 Hz, 3H).

^{13}C NMR (101 MHz, $CDCl_3$) δ 158.19, 139.27, 138.71, 138.15, 134.35, 129.17, 128.62, 128.32, 127.78, 126.49, 116.87, 113.78, 55.39, 49.14, 20.35.

HRMS (EI) m/z calculated for $C_{19}H_{20}O$ [M], 264.1514, found: 264.1510.

Specific rotation $[\alpha]_D^{24}$ = -28.4 (c = 0.60, CH_2Cl_2).

HPLC conditions: Chiral column IA, hexane: isopropanol = 98:2, flow rate = 1.0 mL/min, wavelength = 254 nm, t_R = 9.1 min for major isomer, t_R = 9.8 min for minor isomer.

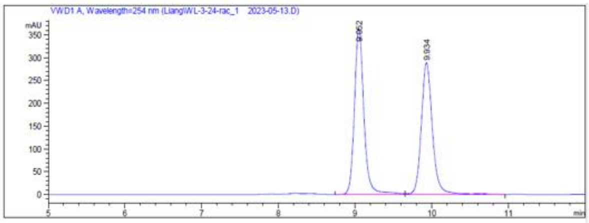

Peak #	RetTime [min]	Type	Width [min]	Area [mAU*s]	Height [mAU]	Area %
1	9.052	BV	0.1369	3141.82373	359.57248	51.7991
2	9.934	VV R	0.1588	2923.57788	288.07278	48.2009

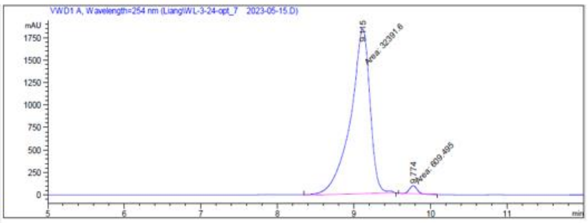

Peak #	RetTime [min]	Type	Width [min]	Area [mAU*s]	Height [mAU]	Area %
1	9.115	MM	0.2943	3.23916e4	1834.67395	98.1531
2	9.774	MF	0.1130	609.49451	89.90153	1.8469

<u>(*S*,*E*)-(3-(4-methoxyphenyl)penta-1,4-diene-1,2-diyl)dibenzene (12)</u>

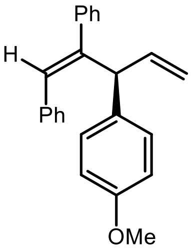

Prepared following **General Procedure A**. Purification by flash column chromatography (silica gel, 5-18% DCM in pentane) gave the title compound (40.5 mg, 62%, >20:1 *E*/*Z*, 99:1 e.r.) as a colorless oil.

R_f = 0.40 (20:1 pentane: EtOAc, UV)

^{1}H NMR (500 MHz, $CDCl_3$) δ 7.40 – 7.38 (m, 2H), 7.37 – 7.34 (m, 2H), 7.28 – 7.25 (m, 1H), 7.22 – 7.20 (m, 7H), 6.90 (s, 1H), 6.83 (d, J = 8.8 Hz, 2H), 6.09 (ddd, J = 17.4, 9.6, 8.0 Hz, 1H), 5.22 – 5.18 (m, 2H), 5.10 (d, J = 8.0 Hz, 1H), 3.80 (s, 3H).

^{13}C NMR (126 MHz, $CDCl_3$) δ 158.21, 143.31, 141.83, 139.00, 137.72, 134.06, 130.98, 129.12, 128.91, 128.63, 128.51, 127.92, 127.12, 127.07, 117.44, 113.85, 55.38, 49.31.

HRMS (EI) m/z calculated for $C_{24}H_{22}O$ [M], 326.1671, found: 326.1658.

Specific rotation $[\alpha]_D^{24}$ = -12.5 (c = 0.30, CH_2Cl_2).

HPLC conditions: Chiral column AD-H, hexane: isopropanol = 99:1, flow rate = 1.0 mL/min, wavelength = 254 nm, t_R = 7.3 min for major isomer, t_R = 9.0 min for minor isomer.

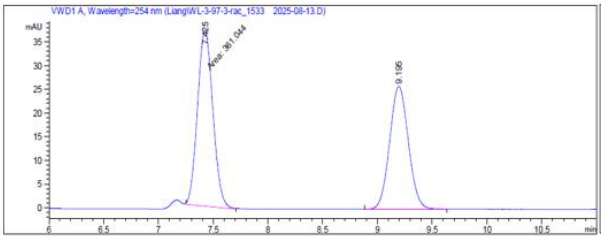

Peak #	RetTime [min]	Type	Width [min]	Area [mAU*s]	Height [mAU]	Area %
1	7.425	MM	0.1625	361.04388	37.02297	53.9749
2	9.195	BV R	0.1835	307.86713	25.89028	46.0251

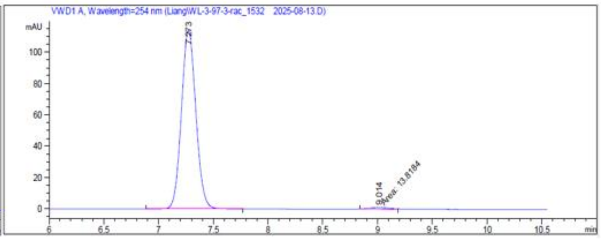

Peak #	RetTime [min]	Type	Width [min]	Area [mAU*s]	Height [mAU]	Area %
1	7.273	BV R	0.1439	1045.33289	113.82639	98.6953
2	9.014	MM	0.1979	13.81838	1.16382	1.3047

<u>(*R*,*Z*)-2-((E)-4,8-dimethylnona-3,7-dien-1-yl)penta-1,4-diene-1,3-diyl)dibenzene (13)</u>

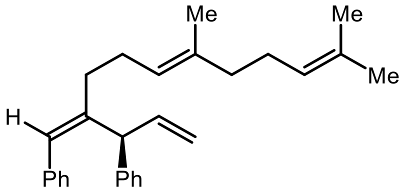

Prepared following **General Procedure A**. Purification by flash column chromatography (silica gel, 5-8% DCM in pentane) gave the title compound (57.7 mg, 81%, >20:1 *Z*/*E*, 99:1 e.r.) as a colorless oil.

R_f = 0.70 (20:1 pentane: EtOAc, UV)

^{1}H NMR (400 MHz, $CDCl_3$) δ 7.25 – 7.17 (m, 6H), 7.15 – 7.08 (m, 4H), 6.49 (s, 1H), 6.12 (ddd, *J* = 17.2, 10.4, 6.8 Hz, 1H), 5.21 (ddd, *J* = 10.4, 1.6 Hz, 1H), 5.09 (ddd, *J* = 17.2, 1.6 Hz, 1H), 5.05 – 4.92 (m, 2H), 4.75 (d, *J* = 6.8 Hz, 1H), 2.11 – 1.90 (m, 5H), 1.89 – 1.79 (m, 3H), 1.58 (s, 3H), 1.50 (s, 3H), 1.45 (s, 3H).

^{13}C NMR (101 MHz, $CDCl_3$) δ 142.74, 142.44, 138.71, 138.40, 135.39, 131.47, 128.69, 128.34, 128.32, 126.85, 126.46, 126.33, 124.48, 124.17, 117.15, 50.25, 39.82, 32.48, 27.41, 26.85, 25.82, 17.82, 16.16.

HRMS (EI) m/z calculated for $C_{28}H_{34}$ [M], 370.2661, found: 370.2653.

Specific rotation $[\alpha]_D^{24}$ = -19.0 (c = 0.15, CH_2Cl_2).

HPLC conditions: Chiral column AD-H, hexane: isopropanol = 99.5:0.5, flow rate = 1.0 mL/min, wavelength = 254 nm, t_R = 3.7 min for major isomer, t_R = 4.0 min for minor isomer.

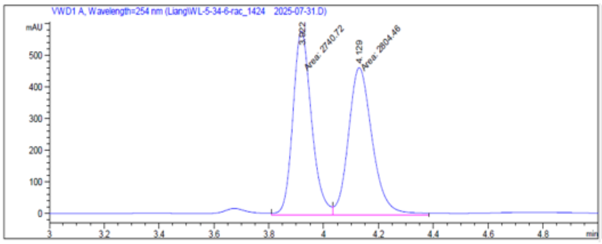

Peak #	RetTime [min]	Type	Width [min]	Area [mAU*s]	Height [mAU]	Area %
1	3.922	MF	0.0787	2740.72046	580.07623	49.4252
2	4.129	FM	0.0996	2804.46313	469.50684	50.5748

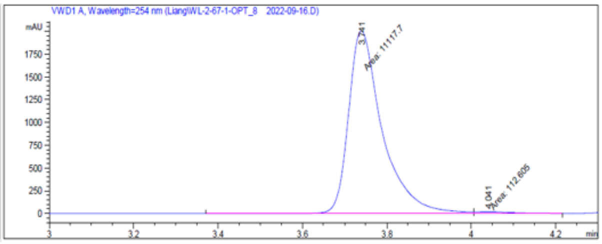

Peak #	RetTime [min]	Type	Width [min]	Area [mAU*s]	Height [mAU]	Area %
1	3.741	MF	0.0910	1.11177e4	2036.64294	98.9973
2	4.041	FM	0.0962	112.60458	19.50890	1.0027

(*R*,*Z*)-2-(3-chloropropyl)penta-1,4-diene-1,3-diyl)dibenzene (14)

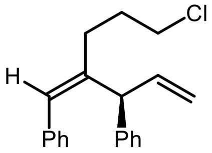

Prepared following **General Procedure A**. Purification by flash column chromatography (silica gel, 5-12% DCM in pentane) gave the title compound (44.5 mg, 75%, >20:1 *Z*/*E*, 96:4 e.r.) as a colorless oil.

R$_f$ = 0.60 (20:1 pentane: EtOAc, UV)

^{1}H NMR (400 MHz, $CDCl_3$) δ 7.30 – 7.06 (m, 10H), 6.49 (s, 1H), 6.13 (ddd, *J* = 17.2, 10.4, 6.4 Hz, 1H), 5.25 (ddd, *J* = 10.4, 1.6 Hz, 1H), 5.11 (ddd, *J* = 17.2, 1.6 Hz, 1H), 4.78 (d, *J* = 6.4 Hz, 1H), 3.46 – 3.28 (m, 2H), 2.16 (dddd, *J* = 15.6, 10.0, 5.6, 1.6 Hz, 1H), 1.98 (dddd, *J* = 15.6, 10.0, 5.6, 1.6 Hz, 1H), 1.87 – 1.74 (m, 1H), 1.76 – 1.60 (m, 1H).

^{13}C NMR (101 MHz, $CDCl_3$) δ 142.09, 141.58, 138.37, 137.94, 128.64, 128.46, 128.41, 128.26, 127.55, 126.72, 126.54, 117.43, 50.14, 44.89, 32.10, 29.89.

HRMS (EI) m/z calculated for $C_{20}H_{21}Cl$ [M], 296.1332, found: 296.1323.

Specific rotation $[\alpha]_D^{24}$ = -13.1 (c = 0.65, CH_2Cl_2).

HPLC conditions: Chiral column IB, hexane: isopropanol = 99:1, flow rate = 1.0 mL/min, wavelength = 254 nm, t_R = 27.0 min for major isomer, t_R = 29.0 min for major isomer.

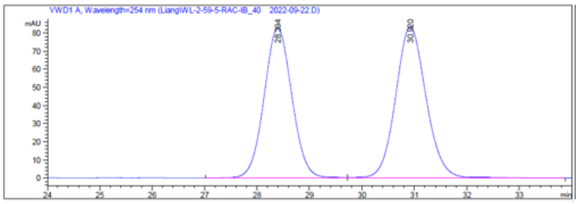

Peak #	RetTime [min]	Type	Width [min]	Area [mAU*s]	Height [mAU]	Area %
1	28.394	BV	0.5749	3052.12573	82.93591	47.5047
2	30.920	VV	0.6224	3372.76172	83.54865	52.4953

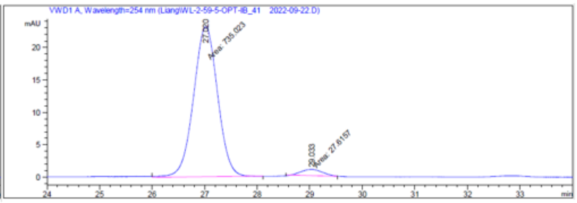

Peak #	RetTime [min]	Type	Width [min]	Area [mAU*s]	Height [mAU]	Area %
1	27.020	MM	0.5295	735.02264	23.13743	96.3789
2	29.033	MM	0.4752	27.61570	9.68477e-1	3.6211

(*R*,*Z*)-((4-benzylidene-5-(4-methoxyphenyl)hept-6-en-1-yl)oxy)(tert-butyl)dimethylsilane (15)

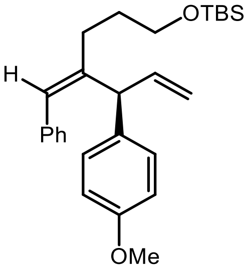

Prepared following **General Procedure A**. Purification by flash column chromatography (silica gel, 5-15% DCM in pentane) gave the title compound (58.3 mg, 69%, >20:1 *Z*/*E*, 99:1 e.r.) as a colorless oil.

R_f = 0.45 (20:1 pentane: EtOAc, UV)

^{1}H NMR (400 MHz, $CDCl_3$) δ 7.33 – 7.25 (m, 4H), 7.23 – 7.18 (m, 1H), 7.06 (d, *J* = 8.4 Hz, 2H), 6.80 (d, *J* = 8.4 Hz, 2H), 6.52 (s, 1H), 6.16 (ddd, *J* = 17.2, 10.4, 6.8 Hz, 1H), 5.26 (ddd, *J* = 10.4, 1.6 Hz, 1H), 5.14 (ddd, *J* = 17.2, 1.6 Hz, 1H), 4.77 (d, *J* = 6.8 Hz, 1H), 3.78 (s, 3H), 3.60 – 3.45 (m, 2H), 2.10 (dddd, *J* = 16.0, 10.8, 5.2, 1.6 Hz, 1H), 1.92 (dddd, *J* = 16.0, 10.4, 5.2, 1.6 Hz, 1H), 1.72 – 1.62 (m, 1H), 1.60 – 1.53 (m, 1H), 0.86 (s, 9H), 0.01 (s, 6H).

^{13}C NMR (101 MHz, $CDCl_3$) δ 158.18, 143.08, 139.00, 138.39, 134.47, 129.24, 128.69, 128.31, 126.44, 126.38, 116.88, 113.76, 63.11, 55.36, 49.54, 32.14, 28.70, 26.10, 18.45, -5.14.

HRMS (ESI) m/z calculated for $C_{27}H_{39}O_2Si$ $[M+H]^+$, 423.2714, found: 423.2707.

Specific rotation $[\alpha]_D^{24}$ = -8.4 (c = 0.30, CH_2Cl_2).

HPLC conditions: Chiral column AD-H, hexane: isopropanol = 99:1, flow rate = 1.0 mL/min, wavelength = 254 nm, t_R = 3.5 min for major isomer, t_R = 3.7 min for minor isomer.

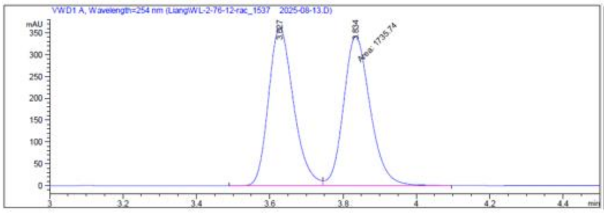

Peak #	RetTime [min]	Type	Width [min]	Area [mAU*s]	Height [mAU]	Area %
1	3.627	BV	0.0731	1717.31482	362.42783	49.7332
2	3.834	MF	0.0844	1735.73755	342.90909	50.2668

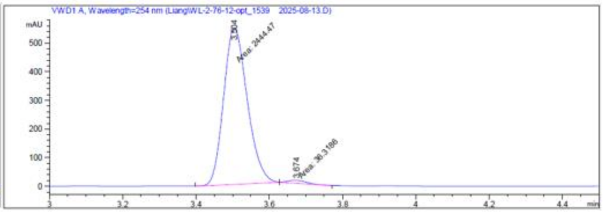

Peak #	RetTime [min]	Type	Width [min]	Area [mAU*s]	Height [mAU]	Area %
1	3.504	MM	0.0745	2444.46802	546.60919	98.5360
2	3.674	MM	0.0572	36.31858	10.58294	1.4640

<u>(*R*,*Z*)-1-(4-benzylidene-5-(4-methoxyphenyl)hept-6-en-1-yl)-1H-pyrrole (16)</u>

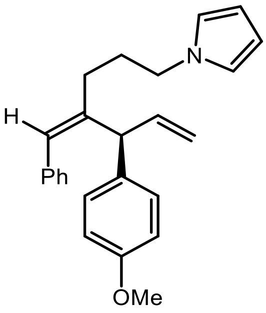

Prepared following **General Procedure A**. Purification by flash column chromatography (silica gel, 5-15% DCM in pentane) gave the title compound (42.2 mg, 59%, >20:1 *Z*/*E*, 98:2 e.r.) as a colorless oil.

$\mathbf{R}_f$ = 0.30 (40:1 pentane: EtOAc, UV)

^{1}H NMR (400 MHz, $CDCl_3$) δ 7.26 – 7.21 (m, 2H), 7.19 – 7.13 (m, 3H), 6.97 (d, *J* = 8.0 Hz, 2H), 6.74 (d, *J* = 8.8 Hz, 2H), 6.50 – 6.49 (m, 2H), 6.43 (s, 1H), 6.10 – 6.01 (m, 3H), 5.20 (ddd, *J* = 10.4, 1.6 Hz, 1H), 5.07 (ddd, *J* = 17.2, 1.6 Hz, 1H), 4.71 (d, *J* = 6.4 Hz, 1H), 3.75 – 3.65 (m, 5H), 2.03 – 1.42 (m, 1H), 1.90 – 1.78 (m, 2H), 1.73 – 1.64 (m, 1H).

^{13}C NMR (101 MHz, $CDCl_3$) δ 158.30, 142.11, 138.71, 137.97, 134.15, 129.21, 128.63, 128.38, 127.08, 126.65, 120.54, 117.11, 113.86, 108.02, 55.40, 49.56, 49.32, 31.09, 29.73.

HRMS (ESI) m/z calculated for $C_{25}H_{28}NO$ $[M+H]^+$, 358.2165, found: 358.2165.

Specific rotation $[\alpha]_D^{24}$ = -26.4 (c = 0.70, CH_2Cl_2).

HPLC conditions: Chiral column AD-H, hexane: isopropanol = 99:1, flow rate = 1.0 mL/min, wavelength = 254 nm, t_R = 6.2 min for major isomer, t_R = 7.3 min for minor isomer.

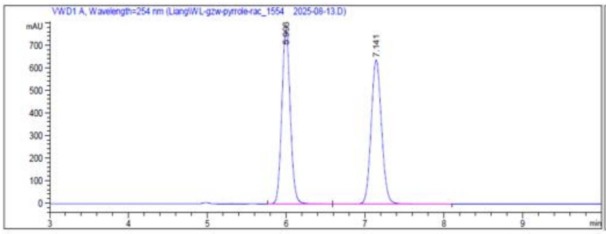

Peak #	RetTime [min]	Type	Width [min]	Area [mAU*s]	Height [mAU]	Area %
1	5.996	BB	0.1167	5778.66016	769.86853	49.7396
2	7.141	VV R	0.1416	5839.17432	639.01599	50.2604

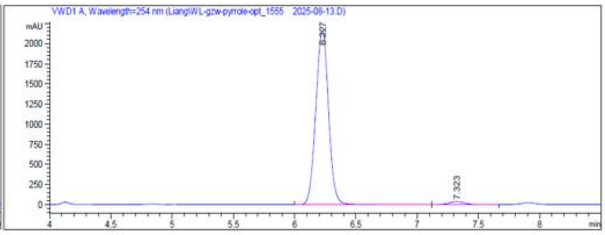

Peak #	RetTime [min]	Type	Width [min]	Area [mAU*s]	Height [mAU]	Area %
1	6.227	BV R	0.1127	1.56394e4	2164.42725	97.9520
2	7.323	VB	0.1390	326.98843	36.52874	2.0480

(*R*,*Z*)-4-(4-methoxybenzylidene)-5-phenylhept-6-en-1-ol (17)

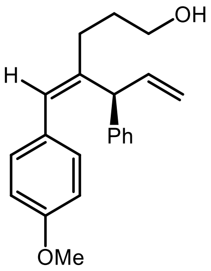

Prepared following **General Procedure A**. Purification by flash column chromatography (silica gel, 5-20% EtOAc in pentane) gave the title compound (45.5 mg, 75%, >20:1 *Z*/*E*, 97:3 e.r.) as a colorless oil.

$\mathbf{R}_f$ = 0.25 (4:1 pentane: EtOAc, UV)

^{1}H NMR (500 MHz, $CDCl_3$) δ 7.31 – 7.25 (m, 3H), 7.24 – 7.14 (m, 5H), 6.87 (d, *J* = 9.0 Hz, 2H), 6.52 (s, 1H), 6.20 (ddd, *J* = 17.0, 10.5, 6.5 Hz, 1H), 5.31 (ddd, *J* = 10.3, 1.5 Hz, 1H), 5.19 (ddd, *J* = 17.0, 1.5 Hz, 1H), 4.86 (d, *J* = 6.5 Hz, 1H), 3.81 (s, 3H), 3.61 – 3.50 (m, 2H), 2.13 (dddd, *J* = 16.0, 10.5, 5.5, 1.5 Hz, 1H), 1.97 (dddd, *J* = 16.0, 10.5, 5.5, 1.5 Hz, 1H), 1.78 – 1.65 (m, 1H), 1.64 – 1.45 (m, 1H).

^{13}C NMR (126 MHz, $CDCl_3$) δ 158.33, 142.39, 141.17, 138.60, 130.59, 129.76, 128.39, 128.30, 126.50, 126.43, 117.24, 113.79, 62.85, 55.38, 50.22, 32.04, 28.55.

HRMS (ESI) m/z calculated for $C_{20}H_{22}$ $[M+H]^+$, 309.1849, found: 309.1847.

Specific rotation $[\alpha]_D^{24}$ = -20.0 (c = 0.50, CH_2Cl_2).

HPLC conditions: Chiral column AD-H, hexane: isopropanol = 95:5, flow rate = 1.0 mL/min, wavelength = 254 nm, t_R = 8.0 min for major isomer, t_R = 9.5 min for major isomer.

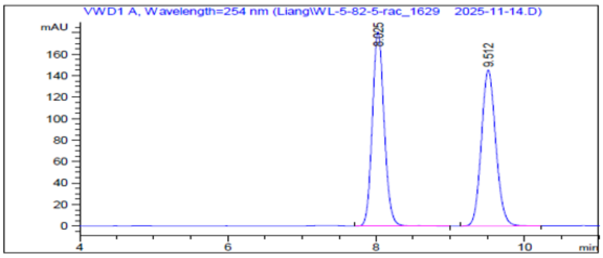

Peak #	RetTime [min]	Type	Width [min]	Area [mAU*s]	Height [mAU]	Area %
1	8.025	BV R	0.1718	2008.46594	180.82683	50.4641
2	9.512	BB	0.2104	1971.52576	145.24678	49.5359

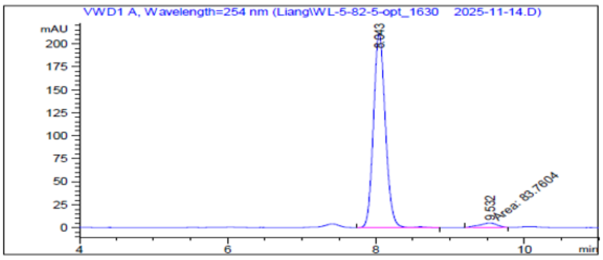

Peak #	RetTime [min]	Type	Width [min]	Area [mAU*s]	Height [mAU]	Area %
1	8.043	BV R	0.1730	2360.91772	211.24928	96.5738
2	9.532	MM	0.2798	83.76036	4.98929	3.4262

3-(4-methoxyphenyl)-1,1-dimethyl-4-((S)-1-phenylallyl)-1,5-dihydro-1λ^4-aza-2λ^4-boraspiro[[1,2]azaborole-2,9'-bicyclo[3.3.1]nonane] (18)

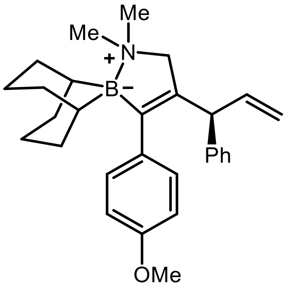

Prepared following **General Procedure A** without AcOH work up. Purification by flash column chromatography (silica gel, 5-15% DCM in pentane) gave the title compound (35.9 mg, 42%, >20:1 *Z*/*E*, 98:2 e.r.) as a colorless oil.

R*f* = 0.55 (20:1 pentane: EtOAc, UV)

^{1}H NMR (500 MHz, $CDCl_3$) δ 7.27 – 7.23 (m, 2H), 7.18 – 7.13 (m, 1H), 7.07 (d, *J* = 7.0 Hz, 2H), 7.05 – 6.99 (m, 2H), 6.86 – 6.79 (m, 2H), 6.02 (ddd, *J* = 17.0, 10.5, 6.0 Hz, 1H), 5.17 (ddd, *J* = 10.5, 2.0 Hz, 1H), 5.07 (ddd, *J* = 17.0, 2.0 Hz, 1H), 4.26 (d, *J* = 6.0 Hz, 1H), 3.81 (s, 3H), 3.49 (d, *J* = 13.5 Hz, 1H), 3.00 (d, *J* = 13.5 Hz, 1H), 2.69 (s, 3H), 2.46 (s, 3H), 1.99 – 1.89 (m, 2H), 1.87 – 1.74 (m, 3H), 1.62 – 1.53 (m, 5H), 1.43 – 1.33 (m, 2H), 1.06 – 0.99 (m, 2H).

^{13}C NMR (126 MHz, $CDCl_3$) δ 156.90, 142.77, 139.07, 138.40, 131.73, 128.64, 128.59, 128.35, 127.94, 126.07, 116.45, 113.02, 112.89, 67.39, 55.26, 48.85, 47.92, 47.54, 34.33, 33.54, 30.91, 30.78, 24.10, 23.62.

^{11}B NMR (400 MHz, $CDCl_3$) δ 7.2

HRMS (MALDI) m/z calculated for $C_{29}H_{38}BNNaO^+$ $[M+Na]^+$, 450.2975, found: 450.2979.

Specific rotation $[\alpha]_D^{24}$ = -27.5 (c = 0.20, CH_2Cl_2).

HPLC conditions: Chiral column IC, hexane: isopropanol = 98:2, flow rate = 1.0 mL/min, wavelength = 254 nm, t_R = 4.1 min for major isomer, t_R = 4.3 min for major isomer.

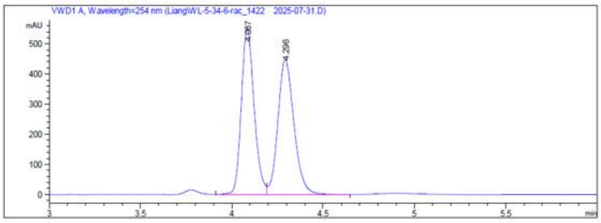

Peak #	RetTime [min]	Type	Width [min]	Area [mAU*s]	Height [mAU]	Area %
1	4.087	BV	0.0872	2685.75977	539.70862	49.4138
2	4.296	VB	0.1026	2749.48047	436.32602	50.5862

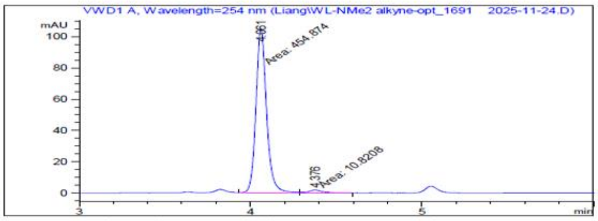

Peak #	RetTime [min]	Type	Width [min]	Area [mAU*s]	Height [mAU]	Area %
1	4.061	MF	0.0722	454.87357	104.98199	97.6764
2	4.376	FM	0.0959	10.82078	1.88111	2.3236

3'-(4-methoxyphenyl)-1'-phenyl-4'-((S)-1-phenylallyl)-1',5'-dihydro-1'λ⁴-thia-9λ⁴-boraspiro[bicyclo[3.3.1]nonane-9,2'-[1,2]thiaborole] (19)

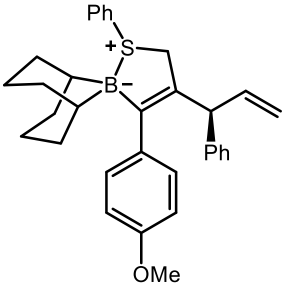

Prepared following **General Procedure A** without AcOH work up. Purification by flash column chromatography (silica gel, 5-10% DCM in pentane) gave the title compound (59.0 mg, 60%, >20:1 *Z*/*E*, >99:1 e.r.) as a colorless oil.

R*f* = 0.60 (20:1 pentane: EtOAc, UV)

^{1}H NMR (400 MHz, $CDCl_3$ δ 7.50 (d, *J* = 7.2 Hz, 2H), 7.41 – 7.33 (m, 1H), 7.29 – 7.23 (m, 4H), 7.21 – 7.02 (m, 5H), 6.95 – 6.87 (m, 2H), 6.09 (ddd, *J* = 16.8, 10.4, 6.0 Hz, 1H), 5.25 (ddd, *J* = 10.4, 1.8 Hz, 1H), 5.15 (ddd, *J* = 16.8, 1.8 Hz, 1H), 4.48 (d, *J* = 6.0 Hz, 1H), 3.86 (s, 3H), 3.83 (d, *J* = 16.8 Hz, 1H), 3.43 (d, *J* = 16.8 Hz, 1H), 1.87 – 1.71 (m, 5H), 1.65 – 1.54 (m, 4H), 1.39 – 1.32 (m, 2H), 1.14 – 1.05 (m, 2H), 0.99 – 0.88 (m, 1H).

^{13}C NMR (101 MHz, $CDCl_3$) δ 157.39, 142.17, 138.19, 132.86, 131.16, 130.46, 129.86, 129.06, 128.82, 128.68, 128.44, 128.17, 126.34, 116.82, 113.43, 113.25, 55.28, 49.54, 42.01, 33.50, 33.32, 31.74, 25.20, 24.24, 22.81, 14.27.

^{11}B NMR (400 MHz, $CDCl_3$) δ 18.9

HRMS (MALDI) m/z calculated for $C_{33}H_{37}BNaOS^+$ $[M+Na]^+$, 515.2556, found: 515.2559.

Specific rotation $[\alpha]_D^{24}$ = -24.3 (c = 1.40, CH_2Cl_2).

HPLC conditions: Chiral column IC, hexane: isopropanol = 98:2, flow rate = 1.0 mL/min, wavelength = 254 nm, t_R = 3.8 min for major isomer, t_R = 4.7 min for major isomer.

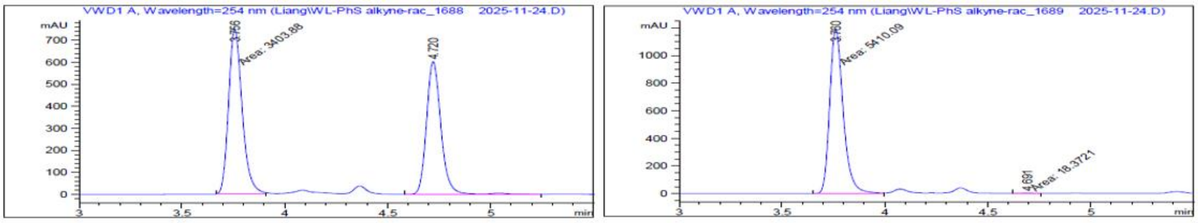

Peak #	RetTime [min]	Type	Width [min]	Area [mAU*s]	Height [mAU]	Area %
1	3.756	MM	0.0759	3403.88184	747.10944	53.7389
2	4.720	BV R	0.0738	2930.22437	601.63135	46.2611

Peak #	RetTime [min]	Type	Width [min]	Area [mAU*s]	Height [mAU]	Area %
1	3.760	MF	0.0758	5410.09277	1190.29712	99.6616
2	4.691	MM	0.0918	18.37212	3.33644	0.3384

(*R*,*Z*)-4,4'-(2-propylpenta-1,4-diene-1,3-diyl)bis(methoxybenzene) (20)

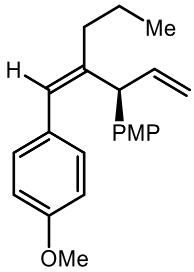

Prepared following **General Procedure A**. Purification by flash column chromatography (silica gel, 5-18% DCM in pentane) gave the title compound (57.3 mg, 89%, >20:1 *Z*/*E*, 98:2 e.r.) as a colorless oil.

R$_f$ = 0.35 (20:1 pentane: EtOAc, UV)

^{1}H NMR (400 MHz, $CDCl_3$) δ 7.21 (d, *J* = 8.0 Hz, 2H), 7.10 (d, *J* = 8.0 Hz, 2H), 6.88 – 6.82 (m, 4H), 6.47 (s, 1H), 6.18 (ddd, *J* = 17.2, 10.4, 6.8 Hz, 1H), 5.27 (ddd, *J* = 10.4, 1.6 Hz, 1H), 5.16 (ddd, *J* = 17.2, 1.6 Hz, 1H), 4.78 (d, *J* = 6.8 Hz, 1H), 3.81 (s, 3H), 3.80 (s, 3H), 2.02 (dddd,

J = 16.0, 10.4, 5.6, 1.6 Hz, 1H), 1.88 (dddd, J = 16.0, 10.4, 5.6, 1.6 Hz, 1H), 1.54 – 1.32 (m, 2H), 0.87 (t, J = 7.2 Hz, 3H).

^{13}C NMR (101 MHz, $CDCl_3$) δ 158.18, 158.10, 142.16, 139.21, 134.63, 130.93, 129.78, 129.21, 125.77, 116.74, 113.71, 113.68, 55.35, 49.49, 34.74, 22.00, 14.33.

HRMS (ESI) m/z calculated for $C_{22}H_{26}O_2$ [M], 322.1933, found: 322.1920.

Specific rotation $[\alpha]_D^{24}$ = -28.0 (c = 0.50, CH_2Cl_2).

HPLC conditions: Chiral column AD-H, hexane: isopropanol = 98:2, flow rate = 1.0 mL/min, wavelength = 254 nm, t_R = 5.3 min for major isomer, t_R = 6.7 min for minor isomer.

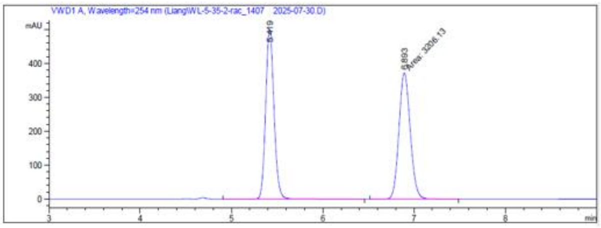

Peak #	RetTime [min]	Type	Width [min]	Area [mAU*s]	Height [mAU]	Area %
1	5.419	VB	0.1107	3178.69678	489.62878	49.7852
2	6.893	MF	0.1429	3206.13135	373.88461	50.2148

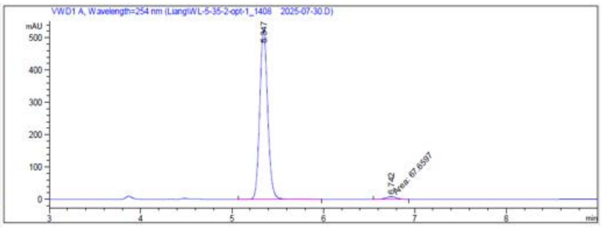

Peak #	RetTime [min]	Type	Width [min]	Area [mAU*s]	Height [mAU]	Area %
1	5.347	VB	0.1006	3176.85254	518.28491	97.9146
2	6.742	MM	0.1426	67.65974	7.90800	2.0854

<u>(*R*,*Z*)-1-fluoro-4-(3-(4-methoxyphenyl)-2-propylpenta-1,4-dien-1-yl)benzene (21)</u>

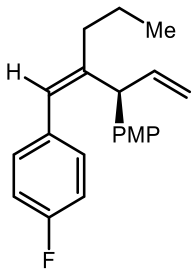

Prepared following **General Procedure A**. Purification by flash column chromatography (silica gel, 5-15% DCM in pentane) gave the title compound (50.2 mg, 81%, >20:1 *Z*/*E*, 97:3 e.r.) as a colorless oil.

R_f = 0.40 (30:1 pentane: EtOAc, UV)

^{1}H NMR (400 MHz, $CDCl_3$) δ 7.23 – 7.19 (m, 2H), 7.07 (d, J = 8.8 Hz, 2H), 7.02 – 6.98 (m, 2H), 6.85 (d, J = 8.8 Hz, 2H), 6.46 (s, 1H), 6.15 (ddd, J = 17.2, 10.4, 6.8 Hz, 1H), 5.26 (ddd, J = 10.4, 1.6 Hz, 1H), 5.13 (ddd, J = 17.2, 1.6 Hz, 1H), 4.70 (d, J = 6.8 Hz, 1H), 3.79 (s, 3H),

2.01 (dddd, J = 16.0, 10.4, 5.6, 1.6 Hz, 1H), 1.87 (dddd, J = 16.0, 10.4, 5.6, 1.6 Hz, 1H), 1.52 – 1.32 (m, 2H), 0.87 (t, J = 7.2 Hz, 3H).

^{13}C NMR (101 MHz, $CDCl_3$) δ 161.59 (J_{CF} = 245.9 Hz), 158.19, 143.51, 139.00, 134.38 (J_{CF} = 3.2 Hz), 134.34, 130.19 (J_{CF} = 7.8 Hz), 129.17, 125.16, 116.88, 115.13 (J_{CF} = 21.3 Hz), 113.75, 55.37, 49.50, 34.65, 21.91, 14.30.

^{19}F NMR (377 MHz, $CDCl_3$) δ -116.42.

HRMS (EI) m/z calculated for $C_{21}H_{23}FO$ [M], 310.1733, found: 310.1721.

Specific rotation $[\alpha]_D^{24}$ = -25.5 (c = 0.55, CH_2Cl_2).

HPLC conditions: Chiral column AD-H, hexane: isopropanol = 99:1, flow rate = 1.0 mL/min, wavelength = 254 nm, t_R = 4.5 min for major isomer, t_R = 5.6 min for minor isomer.

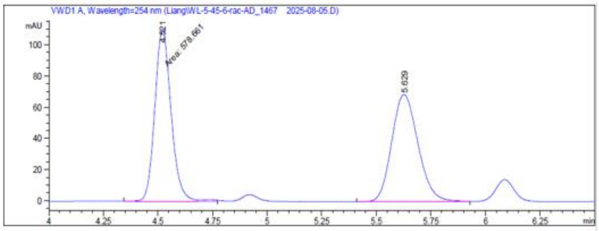

Peak #	RetTime [min]	Type	Width [min]	Area [mAU*s]	Height [mAU]	Area %
1	4.521	FM	0.0862	578.66052	111.89948	50.1045
2	5.629	BV	0.1332	576.24585	68.40592	49.8955

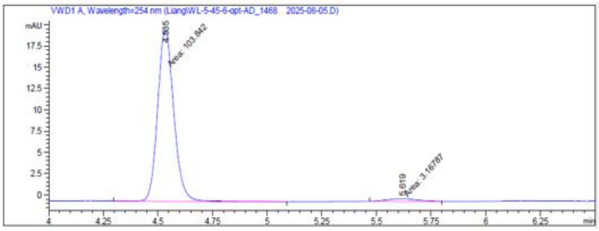

Peak #	RetTime [min]	Type	Width [min]	Area [mAU*s]	Height [mAU]	Area %
1	4.535	FM	0.0847	103.84212	20.44496	97.0396
2	5.619	MM	0.1612	3.16787	3.27566e-1	2.9604

<u>(*R*,*Z*)-1-(4-ethylidenehept-1-en-3-yl)-4-methoxybenzene (22)</u>

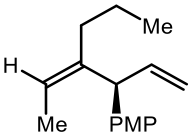

Prepared following **General Procedure A**. Purification by flash column chromatography (silica gel, 5-12% DCM in pentane) gave the title compound (29.9 mg, 65%, >20:1 *Z*/*E*, 96:4 e.r.) as a colorless oil.

R_f = 0.50 (30:1 pentane: EtOAc, $KMnO_4$)

^{1}H NMR (400 MHz, $CDCl_3$) δ 7.13 (d, J = 8.4 Hz, 2H), 6.83 (d, J = 8.4 Hz, 2H), 6.12 (ddd, J = 17.2, 10.4, 7.6 Hz, 1H), 5.42 (qt, J = 6.8, 1.6 Hz, 1H), 5.18 – 5.09 (m, 2H), 4.50 (d, J = 7.6 Hz, 1H), 3.79 (s, 3H), 1.91 – 1.74 (m, 2H), 1.67 (d, J = 6.8 Hz, 3H), 1.41 – 1.23 (m, 2H), 0.81 (t, J = 7.2 Hz, 3H).

^{13}C NMR (101 MHz, $CDCl_3$) δ 157.98, 140.91, 139.02, 134.81, 128.99, 119.91, 115.86, 113.67, 55.38, 49.28, 36.08, 21.82, 14.28, 13.61.

HRMS (EI) m/z calculated for $C_{16}H_{22}O$ [M], 230.1671, found: 230.1661.

Specific rotation $[\alpha]_D^{24}$ = -18.0 (c = 0.80, CH_2Cl_2).

HPLC conditions: Hydroboration/oxidation was conducted following **General Procedure E** for ee determination. Chiral column OD-H, hexane: isopropanol = 98:2, flow rate = 1.0 mL/min, wavelength = 220 nm, t_R = 6.6 min for major isomer, t_R = 8.8 min for minor isomer.

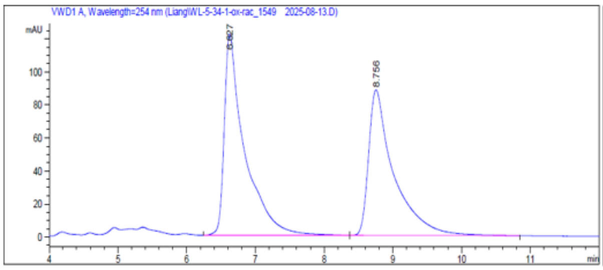

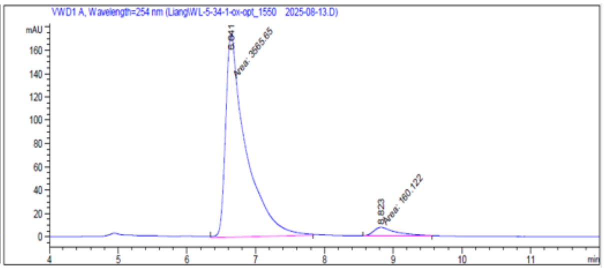

Peak #	RetTime [min]	Type	Width [min]	Area [mAU*s]	Height [mAU]	Area %
1	6.627	BB	0.2794	2509.05225	121.90238	52.8532
2	8.756	BB	0.3532	2238.15845	88.10056	47.1468

Peak #	RetTime [min]	Type	Width [min]	Area [mAU*s]	Height [mAU]	Area %
1	6.641	MM	0.3410	3565.65381	174.29205	95.7023
2	8.823	MM	0.3680	160.12163	7.25256	4.2977

(*R*,*Z*)-1-methoxy-4-(8-phenyl-4-propylocta-1,4-dien-3-yl)benzene (23)

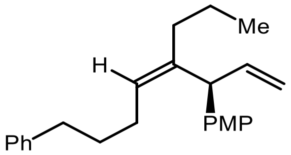

Prepared following **General Procedure B** from allylbenzene. Purification by flash column chromatography (silica gel, 5-12% DCM in pentane) gave the title compound (47.5 mg, 71%, >20:1 *Z*/*E*, >99:1 e.r.) as a colorless oil.

R*f* = 0.55 (30:1 pentane: EtOAc, $KMnO_4$)

^{1}H NMR (400 MHz, $CDCl_3$) δ 7.29 – 7.25 (m, 2H), 7.19 – 7.16 (m, 3H), 7.11 (d, *J* = 8.4 Hz, 2H), 6.83 (d, *J* = 8.4 Hz, 2H), 6.11 (ddd, *J* = 17.2, 10.0, 7.2 Hz, 1H), 5.35 (t, *J* = 7.2 Hz, 1H), 5.16 (ddd, *J* = 10.0, 2.0, 1.2 Hz, 1H), 5.08 (ddd, *J* = 17.2, 1.6 Hz, 1H), 4.46 (d, *J* = 7.2 Hz, 1H), 3.80 (s, 3H), 2.67 (t, *J* = 7.6 Hz, 2H), 2.17 – 2.12 (m, 2H), 1.92 – 1.76 (m, 2H), 1.75 – 1.66 (m, 2H), 1.42 – 1.24 (m, 2H), 0.82 (t, *J* = 7.2 Hz, 3H).

^{13}C NMR (101 MHz, $CDCl_3$) δ 158.02, 142.74, 140.36, 139.16, 134.86, 129.02, 128.56, 128.40, 125.92, 125.79, 115.95, 113.68, 55.39, 49.61, 35.90, 35.82, 31.90, 27.67, 21.90, 14.24.

HRMS (EI) m/z calculated for $C_{24}H_{30}O$ [M], 334.2297, found: 334.2289.

Specific rotation $[\alpha]_D^{24}$ = -15.6 (c = 0.45, CH_2Cl_2).

HPLC conditions: Chiral column IC, hexane: isopropanol = 99:1, flow rate = 1.0 mL/min, wavelength = 230 nm, t_R = 3.7 min for major isomer, t_R = 4.0 min for minor isomer.

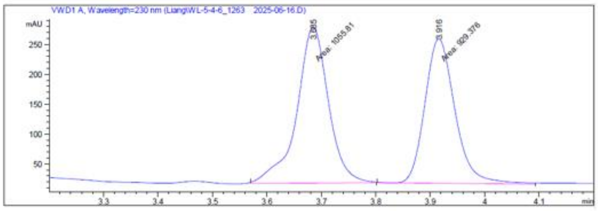

Peak #	RetTime [min]	Type	Width [min]	Area [mAU*s]	Height [mAU]	Area %
1	3.685	MM	0.0662	1055.80920	265.82941	53.1844
2	3.916	MM	0.0631	929.37573	245.52637	46.8156

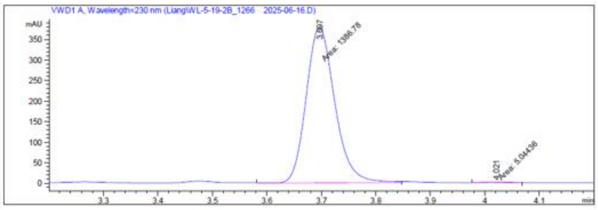

Peak #	RetTime [min]	Type	Width [min]	Area [mAU*s]	Height [mAU]	Area %
1	3.697	MM	0.0598	1386.78101	386.58392	99.6376
2	4.021	MM	0.0529	5.04436	1.58791	0.3624

1-((3*R*,8*S*,*Z*)-8,12-dimethyl-4-propyltrideca-1,4,11-trien-3-yl)-4-methoxybenzene (24)

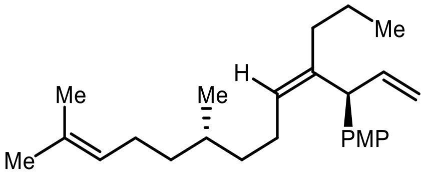

Prepared following **General Procedure B** from (+)-*β*-citronellene. Purification by flash column chromatography (silica gel, 5-12% DCM in pentane) gave the title compound (55.9 mg, 79%, >20:1 d.r.) as a colorless oil.

R_f = 0.55 (30:1 pentane: EtOAc, $KMnO_4$)

^{1}H NMR (400 MHz, $CDCl_3$) δ 7.12 (d, *J* = 8.4 Hz, 2H), 6.83 (d, *J* = 8.4 Hz, 2H), 6.17 – 6.08 (m, 1H), 5.31 (t, *J* = 7.2 Hz, 1H), 5.18 – 5.08 (m, 3H), 4.49 (d, *J* = 7.2 Hz, 1H), 3.79 (s, 3H), 2.17 – 1.90 (m, 4H), 1.88 – 1.72 (m, 2H), 1.68 (s, 3H), 1.60 (s, 3H), 1.47 – 1.23 (m, 6H), 1.20 – 1.08 (m, 2H), 0.86 (d, *J* = 6.4 Hz, 3H), 0.81 (t, *J* = 7.2 Hz, 3H).

^{13}C NMR (101 MHz, $CDCl_3$) δ 157.98, 139.60, 139.25, 134.92, 131.16, 129.01, 126.58, 125.15, 115.90, 113.66, 55.38, 49.56, 37.36, 37.17, 35.81, 32.31, 25.87, 25.69, 25.53, 21.88, 19.68, 17.79, 14.25.

HRMS (EI) m/z calculated for $C_{25}H_{38}O$[M], 354.2923, found: 354.2910.

Specific rotation $[\alpha]_D^{24}$ = -12.5 (c = 0.40, CH_2Cl_2).

(8*R*,9*S*,13*S*,14*S*)-3-(((*R*,*Z*)-8-(4-methoxyphenyl)-7-propyldeca-6,9-dien-1-yl)oxy)-13-methyl-6,7,8,9,11,12,13,14,15,16-decahydro-17H-cyclopenta[a]phenanthren-17-one (25)

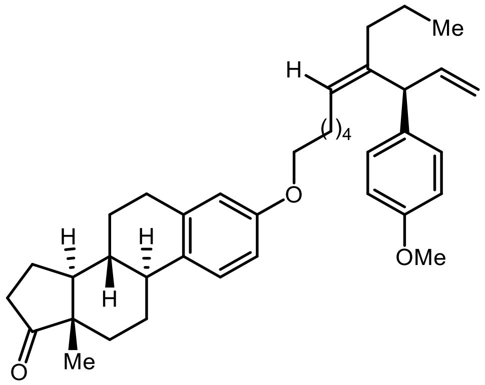

Prepared following **General Procedure B** from O-homoallyl estrone. Purification by flash column chromatography (silica gel, 5-10% EtOAc in pentane) gave the title compound (52.1 mg, 47%, >20:1 d.r.) as a colorless oil.

R*f* = 0.40 (10:1 pentane: EtOAc, $KMnO_4$)

^{1}H NMR (400 MHz, $CDCl_3$) δ 7.19 (d, *J* = 8.8 Hz, 1H), 7.11 (d, *J* = 8.4 Hz, 2H), 6.82 (d, *J* = 8.4 Hz, 2H), 6.71 (dd, *J* = 8.8, 2.8 Hz, 1H), 6.64 (d, *J* = 2.8 Hz, 1H), 6.11 (ddd, *J* = 17.2, 10.4, 7.2 Hz, 1H), 5.32 (t, *J* = 7.2 Hz, 1H), 5.16 (ddd, *J* = 10.4, 2.0, 1.2 Hz, 1H), 5.10 (ddd, *J* = 17.2, 1.6 Hz, 1H), 4.48 (d, *J* = 7.2 Hz, 1H), 3.92 (t, *J* = 6.4 Hz, 2H), 3.78 (s, 3H), 2.93 – 2.82 (m, 2H), 2.50 (dd, *J* = 18.8, 8.4 Hz, 1H), 2.43 – 2.34 (m, 1H), 2.30 – 2.21 (m, 1H), 2.20 – 2.07 (m, 3H), 2.05 – 1.94 (m, 2H), 1.86 – 1.73 (m, 3H), 1.65 – 1.42 (m, 10H), 1.37 – 1.22 (m, 3H), 0.91 (s, 3H), 0.81 (t, *J* = 7.2 Hz, 3H).

^{13}C NMR (101 MHz, $CDCl_3$) δ 221.14, 157.99, 157.30, 140.01, 139.19, 137.82, 134.83, 131.98, 129.00, 126.42, 126.14, 115.95, 114.71, 113.66, 112.26, 67.97, 55.37, 50.57, 49.55, 48.17, 44.14, 38.54, 36.03, 35.82, 31.74, 29.88, 29.80, 29.38, 27.91, 26.72, 26.07, 25.99, 21.88, 21.74, 14.26, 14.00.

HRMS (MALDI) m/z calculated for $C_{38}H_{50}O_3Na$ $[M+Na]^+$, 577.3652, found: 577.3661.

Specific rotation $[\alpha]_D^{24}$ = +11.0 (c = 0.50, CH_2Cl_2).

<u>(*R*,*Z*)-1-(4-benzylidenehept-1-en-3-yl)-4-methoxybenzene (26)</u>

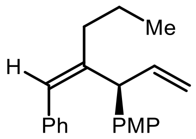

Prepared following **General Procedure A** from BPh_3. Purification by flash column chromatography (silica gel, 5-15% DCM in pentane) gave the title compound (45.0 mg, 77%, >20:1 *Z*/*E*, >99:1 e.r.) as a colorless oil.

R_f = 0.45 (30:1 pentane: EtOAc, UV)

^{1}H NMR (500 MHz, $CDCl_3$) δ 7.33 – 7.28 (m, 4H), 7.23 – 7.20 (m, 1H), 7.09 (d, *J* = 8.4 Hz, 2H), 6.83 (d, *J* = 8.4 Hz, 2H), 6.53 (s, 1H), 6.18 (ddd, *J* = 17.2, 10.4, 6.8 Hz, 1H), 5.27 (ddd, *J* = 10.4, 1.6 Hz, 1H), 5.16 (ddd, *J* = 17.2, 1.6 Hz, 1H), 4.78 (d, *J* = 6.8 Hz, 1H), 3.79 (s, 3H), 2.03 (dddd, *J* = 16.0, 10.4, 5.6, 1.6 Hz, 1H), 1.89 (dddd, *J* = 16.0, 10.4, 5.6, 1.6 Hz, 1H), 1.53 – 1.45 (m, 1H), 1.44 – 1.34 (m, 1H), 0.88 (t, *J* = 7.2 Hz, 3H).

^{13}C NMR (126 MHz, $CDCl_3$) δ 158.13, 143.31, 139.09, 138.48, 134.54, 129.21, 128.69, 128.29, 126.38, 126.26, 116.84, 113.70, 55.37, 49.48, 34.64, 21.95, 14.31.

HRMS (EI) m/z calculated for $C_{21}H_{24}O$ [M], 292.1827, found: 292.1818.

Specific rotation $[\alpha]_D^{24}$ = -33.4 (c = 1.15, CH_2Cl_2).

HPLC conditions: Chiral column AD-H, hexane: isopropanol = 99:1, flow rate = 1.0 mL/min, wavelength = 254 nm, t_R = 3.4 min for major isomer, t_R = 3.7 min for major isomer.

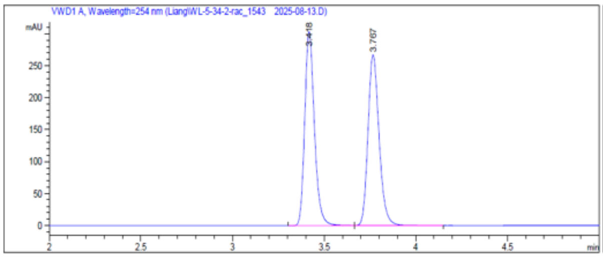

Peak #	RetTime [min]	Type	Width [min]	Area [mAU*s]	Height [mAU]	Area %
1	3.418	BB	0.0560	1115.81079	303.57504	49.8234
2	3.767	BB	0.0647	1123.72119	267.25357	50.1766

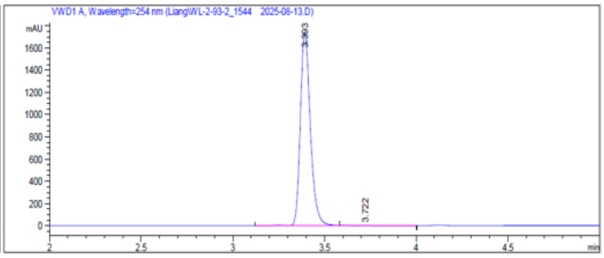

Peak #	RetTime [min]	Type	Width [min]	Area [mAU*s]	Height [mAU]	Area %
1	3.393	VV R	0.0582	6623.09521	1737.42371	99.8237
2	3.722	VB E	0.0895	11.69878	1.80323	0.1763

(*R*,*Z*)-1-methoxy-4-(4-propylnona-1,4-dien-3-yl)benzene (27)

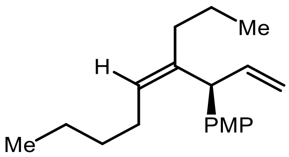

Prepared following **General Procedure A** from *n*-Bu_3B. Purification by flash column chromatography (silica gel, 5-13% DCM in pentane) gave the title compound (40.3 mg, 74%, >20:1 *Z*/*E*, 98:2 e.r.) as a colorless oil.

$\mathbf{R}_f$ = 0.60 (30:1 pentane: EtOAc, $KMnO_4$)

^{1}H NMR (500 MHz, $CDCl_3$) δ 7.12 (d, *J* = 8.4 Hz, 2H), 6.83 (d, *J* = 8.4 Hz, 2H), 6.12 (ddd, *J* = 17.2, 10.0, 7.6 Hz, 1H), 5.32 (tt, *J* = 7.2, 1.6 Hz, 1H), 5.16 (ddd, *J* = 10.0, 2.0, 1.2 Hz, 1H), 5.10 (ddd, *J* = 17.2, 2.0, 1.2 Hz, 1H), 4.49 (d, *J* = 7.2 Hz, 1H), 3.79 (s, 3H), 2.11 – 2.07 (m, 2H), 1.90 – 1.82 (m, 1H), 1.80 – 1.73 (m, 1H), 1.40 – 1.24 (m, 6H), 0.89 (t, *J* = 7.2 Hz, 3H), 0.81 (t, *J* = 7.2 Hz, 3H).

^{13}C NMR (101 MHz, $CDCl_3$) δ 157.98, 139.68, 139.25, 134.92, 129.01, 126.47, 115.89, 113.64, 55.37, 49.52, 35.76, 32.40, 27.71, 22.62, 21.89, 14.25, 14.19.

HRMS (EI) m/z calculated for $C_{19}H_{28}O$ [M], 272.2140, found: 272.2131.

Specific rotation $[\alpha]_D^{24}$ = -11.5 (c = 0.5, CH_2Cl_2).

HPLC conditions: Chiral column AD-H, hexane: isopropanol = 90:10, flow rate = 1.0 mL/min, wavelength = 220 nm, t_R = 9.0 min for major isomer, t_R = 6.5 min for major isomer.

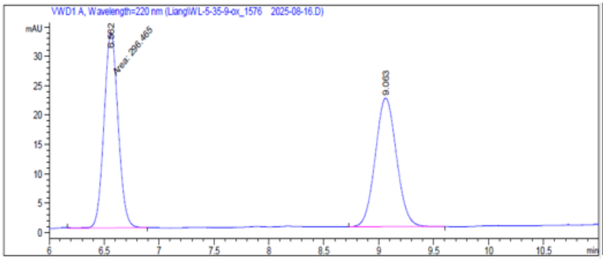

Peak #	RetTime [min]	Type	Width [min]	Area [mAU*s]	Height [mAU]	Area %
1	6.562	FM	0.1490	296.46463	33.16244	50.2428
2	9.063	BV R	0.2070	293.59872	21.84495	49.7572

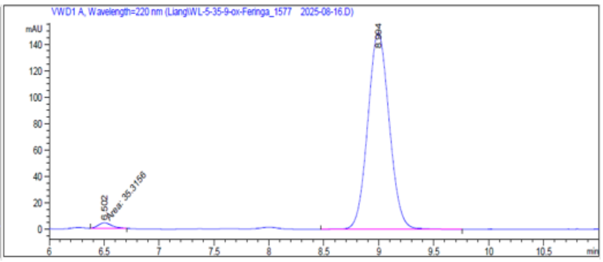

Peak #	RetTime [min]	Type	Width [min]	Area [mAU*s]	Height [mAU]	Area %
1	6.502	MM	0.1432	35.31556	4.11040	1.7189
2	8.994	BB	0.2111	2019.17432	148.44373	98.2811

(*S*,*Z*)-2-((*E*)-4-chlorostyryl)penta-1,4-diene-1,3-diyl)dibenzene (28)

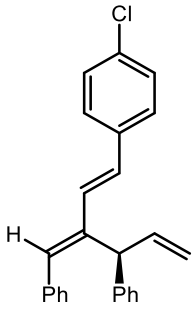

Prepared following **General Procedure A**. Purification by flash column chromatography (silica gel, 5-12% DCM in pentane) gave the title compound (54.2 mg, 76%, >20:1 *Z*/*E*, >99:1 e.r.) as a colorless oil.

R*f* = 0.65 (20:1 pentane: EtOAc, UV)

^{1}H NMR (400 MHz, $CDCl_3$) δ 7.40 – 7.23 (m, 14H), 7.10 (s, 1H), 6.70 (d, *J* = 16.4, 1H), 6.60 (d, *J* = 16.4 Hz, 1H), 6.40 (ddd, *J* = 17.2, 10.4, 6.8 Hz, 1H), 5.38 (ddd, *J* = 10.4, 1.6 Hz, 1H), 5.26 (ddd, *J* = 17.2, 1.6 Hz, 1H), 5.11 (d, *J* = 6.8 Hz, 1H).

^{13}C NMR (101 MHz, $CDCl_3$) δ 141.97, 140.35, 138.42, 137.40, 136.12, 133.06, 131.39, 130.36, 129.48, 128.94, 128.80, 128.56, 128.53, 127.95, 127.62, 127.36, 126.48, 118.07, 48.58.

HRMS (EI) m/z calculated for $C_{25}H_{21}Cl$ [M], 356.1322, found: 356.1313.

Specific rotation $[\alpha]_D^{24}$ = -12.3 (c = 0.30, CH_2Cl_2).

HPLC conditions: Hydroboration/oxidation was conducted following **General Procedure E** for ee determination. Chiral column IB, hexane: isopropanol = 90:10, flow rate = 0.7 mL/min, wavelength = 254 nm, t_R = 7.5 min for major isomer, t_R = 9.3 min for major isomer.

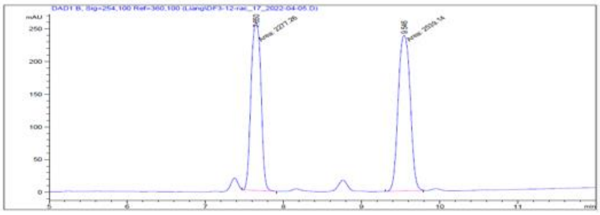

Peak #	RetTime [min]	Sig	Type	Area [mAU*s]	Height [mAU]	Area %
1	7.650	1	MM	2277.26343	256.56064	47.2814
2	9.546	1	MM	2539.13623	237.94763	52.7186

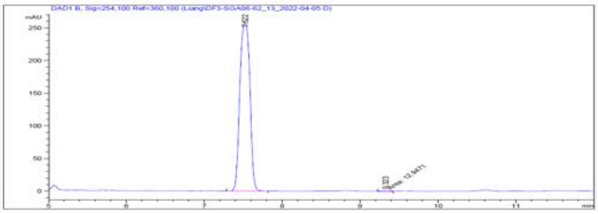

Peak #	RetTime [min]	Sig	Type	Area [mAU*s]	Height [mAU]	Area %
1	7.522	1	BB	2312.47632	258.15982	99.4432
2	9.323	1	MM	12.94712	1.36737	0.5568

(*S*,*Z*)-2-((*E*)-non-1-en-1-yl)penta-1,4-diene-1,3-diyl)dibenzene (29)

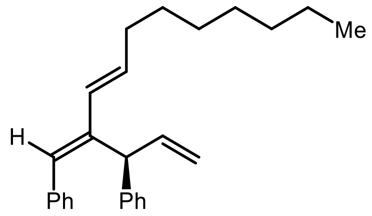

Prepared following **General Procedure A**. Purification by flash column chromatography (silica gel, 5-13% DCM in pentane) gave the title compound (47.5 mg, 69%, >20:1 *Z*/*E*, 98:2 e.r.) as a colorless oil.

R_f = 0.65 (20:1 pentane: EtOAc, UV)

^{1}H NMR (400 MHz, $CDCl_3$) δ 7.36 – 7.21 (m, 10H), 6.85 (s, 1H), 6.33 (ddd, *J* = 17.2, 10.4, 7.2 Hz, 1H), 5.95 (dd, *J* = 15.6, 1.2 Hz, 1H), 5.76 (dt, *J* = 15.6, 6.8 Hz, 1H), 5.33 (ddd, *J* = 10.4, 1.6 Hz, 1H), 5.21 (ddd, *J* = 17.2, 1.6 Hz, 1H), 4.98 (d, *J* = 7.2 Hz, 1H), 2.06 – 1.99 (m, 2H), 1.36 – 1.20 (m, 10H), 0.93 (t, *J* = 7.2 Hz, 3H).

^{13}C NMR (101 MHz, $CDCl_3$) δ 142.40, 140.85, 138.75, 137.94, 133.68, 130.28, 128.89, 128.60, 128.38, 128.32, 127.97, 126.84, 126.16, 117.62, 48.66, 33.30, 31.97, 29.44, 29.27, 29.11, 22.80, 14.25.

HRMS (EI) m/z calculated for $C_{26}H_{32}$ [M], 344.2504, found: 344.2496.

Specific rotation $[\alpha]_D^{24}$ = -10.0 (c = 0.20, CH_2Cl_2).

HPLC conditions: Chiral column IC, hexane: isopropanol = 98:2, flow rate = 1.0 mL/min, wavelength = 254 nm, t_R = 3.9 min for major isomer, t_R = 4.2 min for minor isomer.

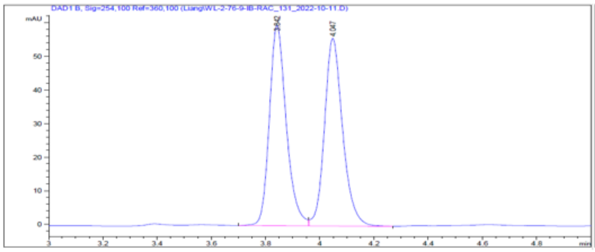

Peak #	RetTime [min]	Sig	Type	Area [mAU*s]	Height [mAU]	Area %
1	3.842	1	BV	258.86069	59.56872	49.8855
2	4.047	1	VB	260.04929	55.84125	50.1145

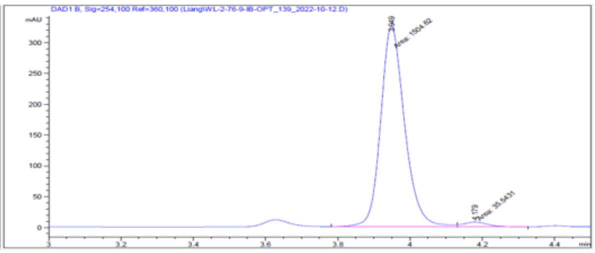

Peak #	RetTime [min]	Sig	Type	Area [mAU*s]	Height [mAU]	Area %
1	3.949	1	MF	1504.62256	325.84521	97.6923
2	4.179	1	FM	35.54311	7.71411	2.3077

((*R*,4*Z*,6*E*)-4-propyldeca-1,4,6-trien-3-yl)benzene (33)

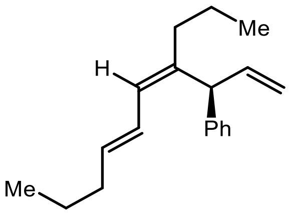

Prepared following **General Procedure B** from 1-pentyne. Purification by flash column chromatography (silica gel, 5-10% DCM in pentane) gave the title compound (47.3 mg, 93%, >20:1 *Z*/*E*, 97:3 e.r.) as a colorless oil.

R_f = 0.65 (30:1 pentane: EtOAc, UV)

^{1}H NMR (400 MHz, $CDCl_3$) δ 7.31 – 7.27 (m, 2H), 7.24 – 7.17 (m, 3H), 6.36 (dd, *J* = 14.4, 10.8 Hz, 1H), 6.18 (ddd, *J* = 17.2, 10.0, 7.2 Hz, 1H), 6.00 (d, *J* = 10.8 Hz, 1H), 5.67 (dt, *J* = 14.4, 7.2 Hz, 1H), 5.21 (ddd, *J* = 10.0, 1.6, 1.2 Hz, 1H), 5.14 (ddd, *J* = 17.2, 1.6 Hz, 1H), 4.68 (d, *J* = 7.2 Hz, 1H), 2.09 – 2.04 (m, 2H), 1.99 – 1.81 (m, 2H), 1.45 – 1.29 (m, 4H), 0.90 (t, *J* = 7.2 Hz, 3H), 0.82 (t, *J* = 7.2 Hz, 3H).

^{13}C NMR (101 MHz, $CDCl_3$) δ 142.63, 140.27, 138.64, 134.20, 128.32, 128.15, 126.40, 126.21, 116.54, 50.69, 36.27, 35.17, 22.75, 21.90, 14.25, 13.86.

HRMS (EI) m/z calculated for $C_{19}H_{26}$ [M], 254.2035, found: 254.2024.

Specific rotation $[\alpha]_D^{24}$ = -19.4 (c = 0.30, CH_2Cl_2).

HPLC conditions: Hydroboration/oxidation was conducted following **General Procedure E** for ee determination. Chiral column IB, hexane: isopropanol = 98:2, flow rate = 1.0 mL/min, wavelength = 254 nm, t_R = 4.1 min for major isomer, t_R = 4.0 min for major isomer.

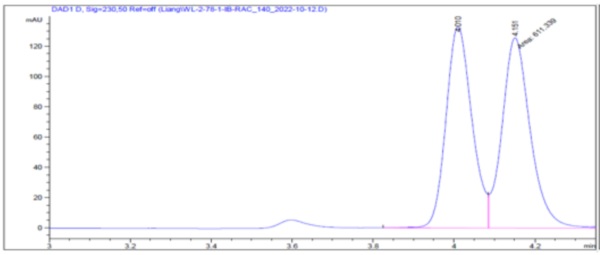

Peak #	RetTime [min]	Sig	Type	Area [mAU*s]	Height [mAU]	Area %
1	4.010	1	BV	593.37445	133.24126	49.2544
2	4.151	1	MF	611.33893	125.91512	50.7456

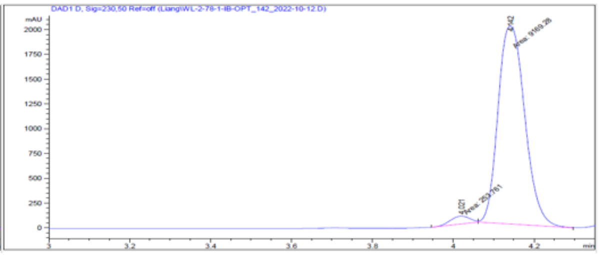

Peak #	RetTime [min]	Sig	Type	Area [mAU*s]	Height [mAU]	Area %
1	4.021	1	MM	253.76134	77.80778	2.6930
2	4.142	1	MM	9169.27637	1996.87329	97.3070

((R,4Z,6E)-4-cyclohexylundeca-1,4,6-trien-3-yl)benzene (31)

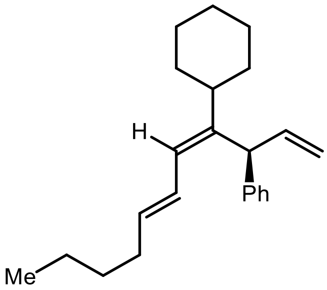

Prepared following **General Procedure B** from 1-hexyne. Purification by flash column chromatography (silica gel, 5-10% DCM in pentane) gave the title compound (53.7 mg, 87%, >20:1 *Z*/*E*, 99:1 e.r.) as a colorless oil.

R_f = 0.75 (30:1 pentane: EtOAc, UV)

^{1}H NMR (400 MHz, $CDCl_3$) δ 7.29 – 7.23 (m, 4H), 7.20 – 7.16 (m, 1H), 6.27 (dd, J = 14.4, 10.8 Hz, 1H), 6.17 (ddd, J = 17.2, 10.0, 7.6 Hz, 1H), 5.99 (d, J = 10.8 Hz, 1H), 5.62 (dt, J = 14.4, 7.2 Hz, 1H), 5.19 (ddd, J = 10.0, 1.6, 1.2 Hz, 1H), 5.13 (ddd, J = 17.2, 1.6 Hz, 1H), 4.59 (d, J = 7.6 Hz, 1H), 2.06 – 2.00 (m, 2H), 1.86 – 1.59 (m, 5H), 1.35 – 1.03 (m, 10H), 0.86 (t, J = 7.2 Hz, 3H).

^{13}C NMR (101 MHz, $CDCl_3$) δ 145.96, 142.79, 139.04, 134.23, 128.22, 128.15, 126.71, 126.14, 125.28, 116.55, 51.24, 43.27, 34.39, 33.99, 32.69, 31.65, 27.10, 27.03, 26.42, 22.35, 14.07.

HRMS (EI) m/z calculated for $C_{23}H_{32}$ [M], 308.2504, found: 308.2498.

Specific rotation $[\alpha]_D^{24}$ = -26.0 (c = 0.90, CH_2Cl_2).

HPLC conditions: Hydroboration/oxidation was conducted following **General Procedure E** for ee determination. Chiral column IB, hexane: isopropanol = 97:3, flow rate = 1.0 mL/min, wavelength = 254 nm, t_R = 7.2 min for major isomer, t_R = 6.6 min for minor isomer.

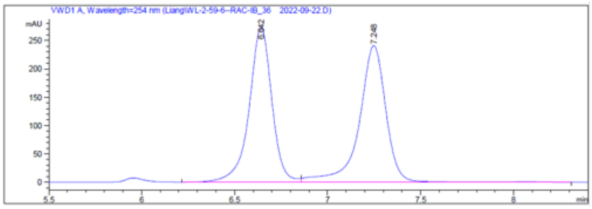

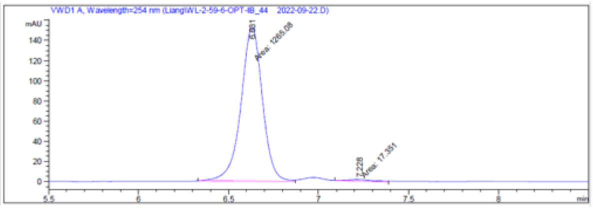

Peak #	RetTime [min]	Type	Width [min]	Area [mAU*s]	Height [mAU]	Area %
1	6.642	BV	0.1350	2321.61182	270.74573	49.0064
2	7.248	VV R	0.1580	2415.75684	239.81844	50.9936

Peak #	RetTime [min]	Type	Width [min]	Area [mAU*s]	Height [mAU]	Area %
1	6.631	MM	0.1375	1265.07800	153.39435	98.6470
2	7.228	MM	0.1813	17.35097	1.59544	1.3530

<u>**(*R*,*Z*)-1-(4-benzylidenehept-1-en-3-yl)-4-methylbenzene (32)**</u>

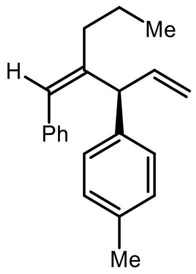

Prepared following **General Procedure C**. Purification by flash column chromatography (silica gel, 5-12% DCM in pentane) gave the title compound (47.0 mg, 85%, >20:1 *Z*/*E*, 99:1 e.r.) as a colorless oil.

R_f = 0.60 (30:1 pentane: EtOAc, UV)

^{1}H NMR (400 MHz, $CDCl_3$) δ 7.33 – 7.28 (m, 4H), 7.23 – 7.20 (m, 1H), 7.11 – 7.06 (m, 4H), 6.53 (s, 1H), 6.18 (ddd, *J* = 17.2, 10.4, 6.8 Hz, 1H), 5.27 (ddd, *J* = 10.4, 1.6 Hz, 1H), 5.15 (ddd, *J* = 17.2, 1.6 Hz, 1H), 4.79 (d, *J* = 6.8 Hz, 1H), 2.32 (s, 3H), 2.04 (dddd, *J* = 16.0, 10.4, 5.6, 1.6 Hz, 1H), 1.90 (dddd, *J* = 16.0, 10.4, 5.6, 1.6 Hz, 1H), 1.54 – 1.37 (m, 2H), 0.88 (t, *J* = 7.2 Hz, 3H).

^{13}C NMR (101 MHz, $CDCl_3$) δ 143.24, 139.44, 139.02, 138.52, 135.78, 129.03, 128.70, 128.29, 128.14, 126.37, 126.33, 116.89, 49.92, 34.65, 21.93, 21.14, 14.29.

HRMS (ESI) m/z calculated for $C_{21}H_{24}$ [M], 276.1878, found: 276.1868.

Specific rotation $[\alpha]_D^{24}$ = -20.0 (c = 0.65, CH_2Cl_2).

HPLC conditions: Chiral column IA, hexane: isopropanol = 98:2, flow rate = 1.0 mL/min, wavelength = 254 nm, t_R = 4.4 min for major isomer, t_R = 4.7 min for minor isomer.

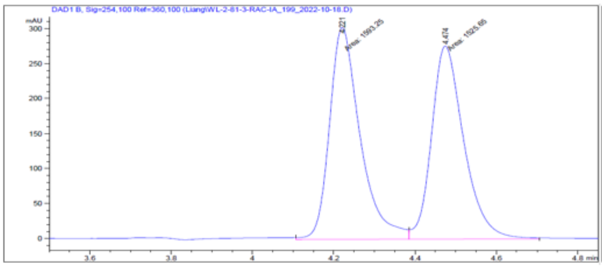

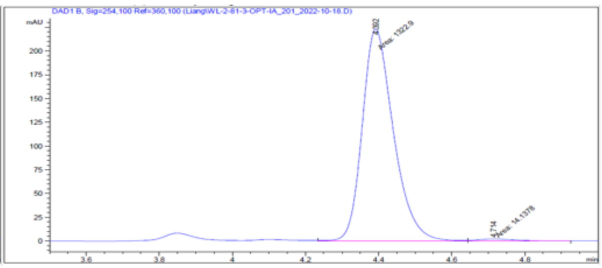

Peak #	RetTime [min]	Sig	Type	Area [mAU*s]	Height [mAU]	Area %
1	4.221	1	FM	1593.25146	302.67999	51.0837
2	4.474	1	MF	1525.65063	275.95703	48.9163

Peak #	RetTime [min]	Sig	Type	Area [mAU*s]	Height [mAU]	Area %
1	4.392	1	MF	1322.89526	223.22603	98.9426
2	4.714	1	FM	14.13780	2.06976	1.0574

(*R*,*Z*)-4-(4-benzylidenehept-1-en-3-yl)-1,1'-biphenyl (33)

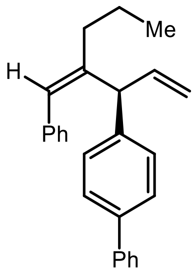

Prepared following **General Procedure C**. Purification by flash column chromatography (silica gel, 5-15% DCM in pentane) gave the title compound (48.0 mg, 71%, >20:1 *Z*/*E*, 99:1 e.r.) as a colorless oil.

R_f = 0.55 (20:1 pentane: EtOAc, UV)

^{1}H NMR (400 MHz, $CDCl_3$) δ 7.62 – 7.60 (m, 2H), 7.54 (d, *J* = 8.4 Hz, 2H), 7.46 – 7.42 (m, 2H), 7.37 – 7.30 (m, 5H), 7.28 – 7.23 (m, 3H), 6.59 (s, 1H), 6.25 (ddd, *J* = 17.2, 10.4, 6.8 Hz, 1H), 5.33 (ddd, *J* = 10.4, 1.6 Hz, 1H), 5.22 (ddd, *J* = 17.2, 1.6 Hz, 1H), 4.89 (d, *J* = 6.8 Hz, 1H), 2.10 (dddd, *J* = 16.0, 10.4, 5.6, 1.6 Hz, 1H), 1.96 (dddd, *J* = 16.0, 10.4, 5.6, 1.6 Hz, 1H), 1.61 – 1.43 (m, 2H), 0.91 (t, *J* = 7.2 Hz, 3H).

^{13}C NMR (101 MHz, $CDCl_3$) δ 142.97, 141.66, 141.07, 139.16, 138.69, 138.44, 128.86, 128.71, 128.66, 128.34, 127.23, 127.13, 127.02, 126.59, 126.46, 117.23, 50.02, 34.69, 21.93, 14.30.

HRMS (EI) m/z calculated for $C_{26}H_{26}$ [M], 338.2035, found: 338.2022.

Specific rotation $[\alpha]_D^{24}$ = -61.1 (c = 0.90, CH_2Cl_2).

HPLC conditions: Chiral column AD-H, hexane: isopropanol = 99:1, flow rate = 1.0 mL/min, wavelength = 254 nm, t_R = 4.7 min for major isomer, t_R = 4.2 min for minor isomer.

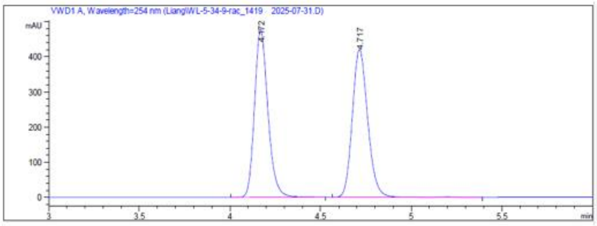

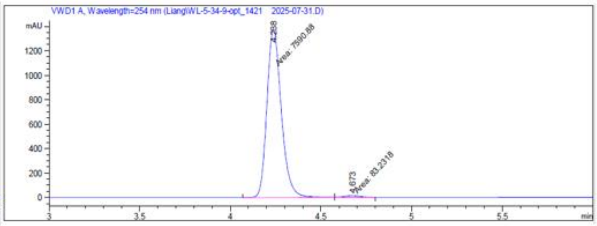

Peak #	RetTime [min]	Type	Width [min]	Area [mAU*s]	Height [mAU]	Area %
1	4.172	BB	0.0901	2452.02490	469.37085	49.7361
2	4.717	BV R	0.1046	2478.04858	413.66013	50.2639

Peak #	RetTime [min]	Type	Width [min]	Area [mAU*s]	Height [mAU]	Area %
1	4.238	MM	0.0913	7590.87646	1385.80432	98.9154
2	4.673	MM	0.0956	83.23177	14.51372	1.0846

(*R*,*Z*)-1-(4-benzylidenehept-1-en-3-yl)-4-bromobenzene (34)

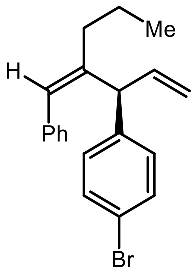

Prepared following **General Procedure C**. Purification by flash column chromatography (silica gel, 5-12% DCM in pentane) gave the title compound (61.4 mg, 90%, >20:1 *Z*/*E*, >99:1 e.r.) as a colorless oil.

R$_f$ = 0.60 (30:1 pentane: EtOAc, UV)

^{1}H NMR (400 MHz, $CDCl_3$) δ 7.39 (d, *J* = 8.4 Hz, 2H), 7.34 – 7.30 (m, 2H), 7.25 – 7.20 (m, 3H), 7.04 (d, *J* = 8.4 Hz, 2H), 6.55 (s, 1H), 6.14 (ddd, *J* = 17.2, 10.4, 6.8 Hz, 1H), 5.30 (ddd, *J* = 10.4, 1.6 Hz, 1H), 5.17 (ddd, *J* = 17.2, 1.6 Hz, 1H), 4.75 (d, *J* = 6.8 Hz, 1H), 2.01 (dddd, *J* = 16.0, 10.4, 5.6, 1.6 Hz, 1H), 1.85 (dddd, *J* = 16.0, 10.4, 5.6, 1.6 Hz, 1H), 1.52 – 1.34 (m, 2H), 0.88 (t, *J* = 7.2 Hz, 3H).

^{13}C NMR (101 MHz, $CDCl_3$) δ 142.42, 141.60, 138.25, 138.15, 131.38, 130.05, 128.64, 128.38, 126.93, 126.58, 120.23, 117.61, 49.78, 34.60, 21.90, 14.26.

HRMS (EI) m/z calculated for $C_{20}H_{21}Br$ [M], 340.0827, found: 340.0818.

Specific rotation $[\alpha]_D^{24}$ = -25.0 (c = 0.50, CH_2Cl_2).

HPLC conditions: Chiral column IA, hexane: isopropanol = 98:2, flow rate = 1.0 mL/min, wavelength = 254 nm, t_R = 4.7 min for major isomer, t_R = 5.0 min for minor isomer.

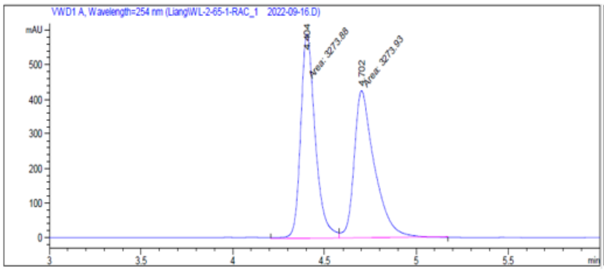

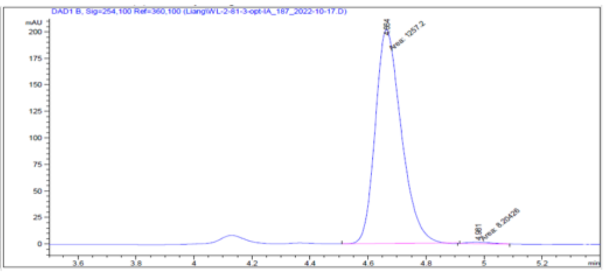

Peak #	RetTime [min]	Type	Width [min]	Area [mAU*s]	Height [mAU]	Area %
1	4.404	MF	0.0910	3273.87744	599.30286	49.9996
2	4.702	FM	0.1270	3273.92847	429.52527	50.0004

Peak #	RetTime [min]	Sig	Type	Area [mAU*s]	Height [mAU]	Area %
1	4.664	1	MM	1257.19958	201.45433	99.3516
2	4.981	1	MM	8.20426	1.43135	0.6484

<u>(*R*,*Z*)-4-(4-benzylidenehept-1-en-3-yl)-1,2-dichlorobenzene (35)</u>

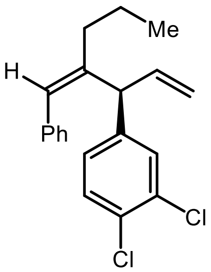

Prepared following **General Procedure C**. Purification by flash column chromatography (silica gel, 5-12% DCM in pentane) gave the title compound (54.8 mg, 83%, >20:1 *Z*/*E*, >99:1 e.r.) as a colorless oil.

R*f* = 0.60 (30:1 pentane: EtOAc, UV)

^{1}H NMR (400 MHz, $CDCl_3$) δ 7.34 – 7.31 (m, 3H), 7.25 – 7.22 (m, 4H), 7.00 (dd, *J* = 8.4, 2.0 Hz, 1H), 6.57 (s, 1H), 6.11 (ddd, *J* = 17.2, 10.4, 7.2 Hz, 1H), 5.33 (ddd, *J* = 10.4, 1.6 Hz, 1H), 5.18 (ddd, *J* = 17.2, 1.6 Hz, 1H), 4.74 (d, *J* = 7.2 Hz, 1H), 2.00 (dddd, *J* = 16.0, 10.4, 5.6, 1.6 Hz, 1H), 1.84 (dddd, *J* = 16.0, 10.0, 5.6, 1.6 Hz, 1H), 1.54 – 1.35 (m, 3H), 0.89 (t, *J* = 7.2 Hz, 3H).

^{13}C NMR (101 MHz, $CDCl_3$) δ 142.95, 141.81, 138.03, 137.48, 132.37, 130.30, 130.20, 130.17, 128.60, 128.44, 127.72, 127.36, 126.71, 118.18, 49.55, 34.55, 21.85, 14.24.

HRMS (EI) m/z calculated for $C_{20}H_{20}Cl_2$ [M], 330.0942, found: 330.0931.

Specific rotation $[\alpha]_D^{24}$ = -30.8 (c = 0.55, CH_2Cl_2).

HPLC conditions: Chiral column AD-H, hexane: isopropanol = 99.5:0.5, flow rate = 1.0 mL/min, wavelength = 254 nm, t_R = 3.5 min for major isomer, t_R = 3.8 min for major isomer.

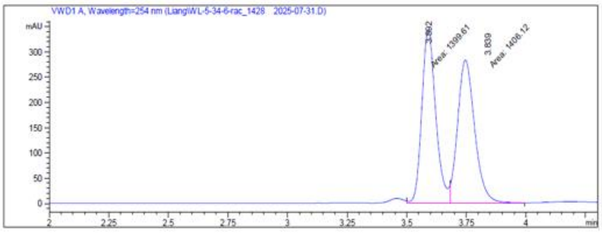

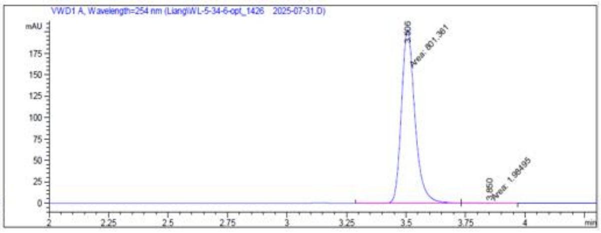

Peak #	RetTime [min]	Type	Width [min]	Area [mAU*s]	Height [mAU]	Area %
1	3.592	MF	0.0686	1399.60852	340.28085	49.8839
2	3.839	MF	0.0815	1406.12463	287.45078	50.1161

Peak #	RetTime [min]	Type	Width [min]	Area [mAU*s]	Height [mAU]	Area %
1	3.506	FM	0.0644	801.36115	207.33234	99.7529
2	3.850	MF	0.1007	1.98495	2.62938e-1	0.2471

(*R*,*Z*)-1-(4-benzylidenehept-1-en-3-yl)-2-methylbenzene (36)

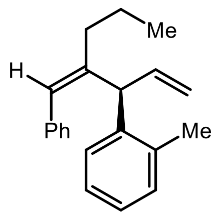

Prepared following **General Procedure C**. Purification by flash column chromatography (silica gel, 5-10% DCM in pentane) gave the title compound (33.2 mg, 60%, 7:1 *Z*/*E*, 97:3) as a colorless oil.

R_f = 0.60 (30:1 pentane: EtOAc, UV)

^{1}H NMR (400 MHz, $CDCl_3$) δ 7.34 – 7.28 (m, 4H), 7.24 – 7.09 (m, 4H), 7.05 – 7.03 (m, 1H), 6.56 (s, 1H), 6.22 (ddd, *J* = 17.2, 10.4, 4.4 Hz, 1H), 5.37 (ddd, *J* = 10.4, 2.0 Hz, 1H), 5.24 (ddd, *J* = 17.4, 2.0 Hz, 1H), 4.82 – 4.80 (m, 1H), 1.97 (dddd, *J* = 15.6, 10.4, 5.2, 1.6 Hz, 1H), 1.85 (s, 3H), 1.71 (dddd, *J* = 15.6, 10.0, 5.2, 1.6 Hz, 1H), 1.36 – 1.26 (m, 1H), 1.19 – 1.07 (m, 1H), 0.78 (t, *J* = 7.2 Hz, 3H).

^{13}C NMR (101 MHz, $CDCl_3$) δ 140.63, 140.48, 140.16, 138.32, 137.34, 130.22, 128.66, 128.36, 128.31, 128.19, 127.55, 126.46, 125.68, 116.44, 47.40, 35.47, 21.99, 19.28, 14.25.

HRMS (EI) m/z calculated for $C_{21}H_{24}$ [M], 276.1878, found: 276.1866.

Specific rotation $[\alpha]_D^{24}$ = -16.7 (c = 0.50, CH_2Cl_2).

HPLC conditions: Chiral column AD-H, hexane: isopropanol = 99:1, flow rate = 1.0 mL/min, wavelength = 254 nm, t_R = 3.3 min for major isomer, t_R = 3.5 min for minor isomer.

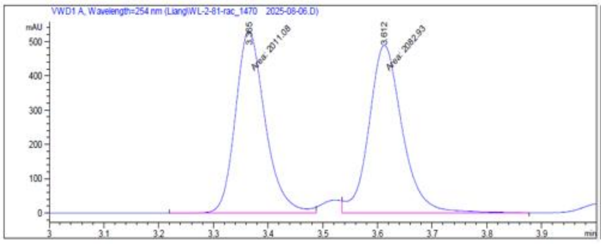

Peak #	RetTime [min]	Type	Width [min]	Area [mAU*s]	Height [mAU]	Area %
1	3.365	FM	0.0635	2011.08179	528.01489	49.1226
2	3.612	FM	0.0699	2082.92603	496.49310	50.8774

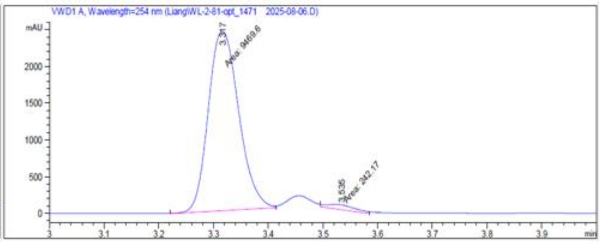

Peak #	RetTime [min]	Type	Width [min]	Area [mAU*s]	Height [mAU]	Area %
1	3.317	MM	0.0635	9469.60156	2485.29468	97.5064
2	3.535	MM	0.0612	242.16972	65.93845	2.4936

(*R*,*Z*)-2-(4-benzylidenehept-1-en-3-yl)naphthalene (37)

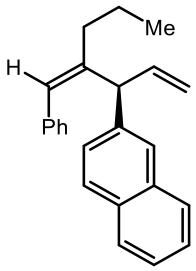

Prepared following **General Procedure C**. Purification by flash column chromatography (silica gel, 5-13% DCM in pentane) gave the title compound (51.2 mg, 82%, >20:1 *Z*/*E*, 99:1 e.r.) as a colorless oil.

$\mathbf{R_f}$ = 0.58 (20:1 pentane: EtOAc, UV)

^{1}H NMR (400 MHz, $CDCl_3$) δ 7.83 – 7.80 (m, 2H), 7.76 (d, *J* = 8.4 Hz, 1H), 7.65 (s, 1H), 7.49 – 7.42 (m, 2H), 7.35 – 7.34 (m, 4H), 7.30 (dd, *J* = 8.4, 1.6 Hz, 1H), 7.27 – 7.23 (m, 1H), 6.63 (s, 1H), 6.33 (ddd, *J* = 17.2, 10.4, 6.8 Hz, 1H), 5.38 (ddd, *J* = 10.4, 1.6 Hz, 1H), 5.26 (ddd, *J* = 17.2, 1.6 Hz, 1H), 5.00 (d, *J* = 6.8 Hz, 1H), 2.09 (dddd, *J* = 16.0, 10.4, 5.6, 1.6 Hz, 1H), 1.92 (dddd, *J* = 16.0, 10.4, 5.6, 1.6 Hz, 1H), 1.57 – 1.39 (m, 2H), 0.86 (t, *J* = 7.2 Hz, 3H).

^{13}C NMR (101 MHz, $CDCl_3$) δ 142.85, 140.15, 138.68, 138.45, 133.56, 132.28, 128.73, 128.36, 127.94, 127.83, 127.67, 127.18, 126.76, 126.49, 126.37, 126.02, 125.56, 117.36, 50.41, 34.75, 21.86, 14.27.

HRMS (EI) m/z calculated for $C_{24}H_{24}$ [M], 312.1878, found: 312.1864

Specific rotation $[\alpha]_D^{24}$ = -47.3 (c = 1.30, CH_2Cl_2).

HPLC conditions: Chiral column AD-H, hexane: isopropanol = 98:2, flow rate = 1.0 mL/min, wavelength = 254 nm, t_R = 5.1 min for major isomer, t_R = 6.5 min for minor isomer.

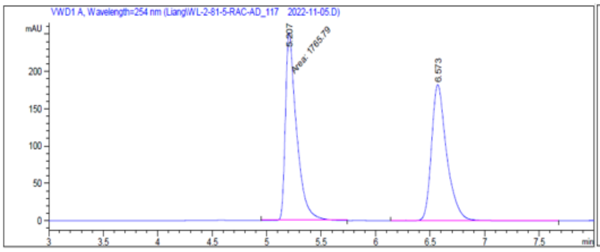

Peak #	RetTime [min]	Type	Width [min]	Area [mAU*s]	Height [mAU]	Area %
1	5.207	MM	0.1153	1765.78833	255.25128	49.9255
2	6.573	BB	0.1546	1771.05762	181.58685	50.0745

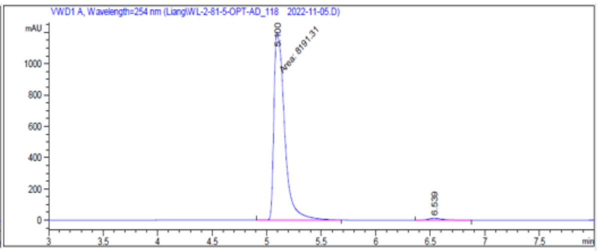

Peak #	RetTime [min]	Type	Width [min]	Area [mAU*s]	Height [mAU]	Area %
1	5.100	MF	0.1142	8191.31299	1195.12158	98.6267
2	6.539	BB	0.1388	114.05585	12.80716	1.3733

(*R*,*Z*)-2-(4-benzylidenehept-1-en-3-yl)thiophene (38)

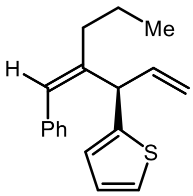

Prepared following **General Procedure C**. Purification by flash column chromatography (silica gel, 5-12% DCM in pentane) gave the title compound (36.0 mg, 67%, >20:1 *Z*/*E*, 96:4 e.r.) as a colorless oil.

R$_f$ = 0.60 (30:1 pentane: EtOAc, UV)

^{1}H NMR (400 MHz, CDCl3) δ 7.34 – 7.26 (m, 4H), 7.24 – 7.20 (m, 1H), 7.17 (d, *J* = 5.2 Hz, 1H), 6.95 (dd, *J* = 5.2, 3.6 Hz, 1H), 6.81 – 6.80 (m, 1H), 6.52 (s, 1H), 6.19 (ddd, *J* = 17.2, 10.0, 6.8 Hz, 1H), 5.23 (ddd, *J* = 10.0, 1.6 Hz, 1H), 5.16 (ddd, *J* = 17.2, 1.6 Hz, 1H), 4.94 (d, *J* = 6.8 Hz, 1H), 2.16 – 2.02 (m, 2H), 1.63 – 1.44 (m, 2H), 0.93 (t, *J* = 7.2 Hz, 3H).

^{13}C NMR (101 MHz, $CDCl_3$) δ 146.92, 142.90, 138.19, 138.13, 128.71, 128.37, 126.88, 126.57, 126.44, 124.63, 124.04, 117.13, 46.63, 34.46, 22.05, 14.30.

HRMS (EI) m/z calculated for $C_{18}H_{20}S$ [M], 268.1286, found: 268.1278.

Specific rotation $[\alpha]_D^{24}$ = -10.0 (c = 0.80, CH_2Cl_2).

HPLC conditions: Chiral column AD-H, hexane: isopropanol = 99:1, flow rate = 1.0 mL/min, wavelength = 254 nm, t_R = 4.3 min for major isomer, t_R = 3.9 min for minor isomer.

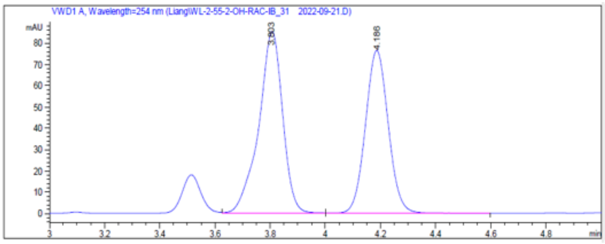

Peak #	RetTime [min]	Type	Width [min]	Area [mAU*s]	Height [mAU]	Area %
1	3.803	VV	0.1091	515.17853	81.04638	53.9188
2	4.186	VB	0.0970	440.29306	75.62768	46.0812

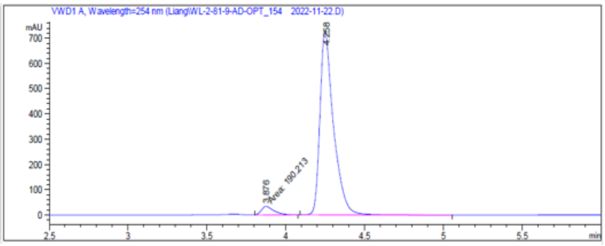

Peak #	RetTime [min]	Type	Width [min]	Area [mAU*s]	Height [mAU]	Area %
1	3.876	FM	0.0910	190.21324	34.82990	4.1841
2	4.258	BB	0.1037	4355.93408	681.50812	95.8159

((S,1Z,4E)-2-propyl-3-vinylpenta-1,4-diene-1,5-diyl)dibenzene (39)

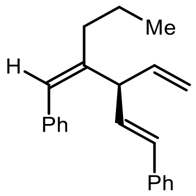

Prepared following **General Procedure D**. Purification by flash column chromatography (silica gel, 5-12% DCM in pentane) gave the title compound (36.9 mg, 64%, 11:1 *rr*, >20:1 *Z*/*E*, 97:3 e.r.) as a colorless oil.

R_f = 0.60 (30:1 pentane: EtOAc, UV)

^{1}H NMR (400 MHz, $CDCl_3$) δ 7.39 – 7.21 (m, 10H), 6.48 (s, 1H), 6.40 – 6.29 (m, 2H), 6.03 (ddd, J = 17.2, 10.4, 5.6 Hz, 1H), 5.18 (ddd, J = 10.4, 1.6 Hz, 1H), 5.13 (ddd, J = 17.2, 1.6 Hz, 1H), 4.35 (dd, J = 5.6 Hz, 1H), 2.20 – 2.16 (m, 2H), 1.68 – 1.58 (m, 2H), 1.00 (t, J = 7.2 Hz, 3H).

^{13}C NMR (101 MHz, $CDCl_3$) δ 142.93, 139.30, 138.37, 137.69, 131.02, 130.88, 128.85, 128.65, 128.27, 127.30, 126.39, 126.30, 126.14, 116.01, 48.00, 35.03, 22.21, 14.33.

HRMS (EI) m/z calculated for $C_{22}H_{24}$ [M], 288.1878, found: 288.1867.

Specific rotation $[\alpha]_D^{24}$ = -55.3 (c = 0.75, CH_2Cl_2).

HPLC conditions: Chiral column AD-H, hexane: isopropanol = 99:1, flow rate = 1.0 mL/min, wavelength = 254 nm, t_R = 3.9 min for major isomer, t_R = 3.5 min for minor isomer.

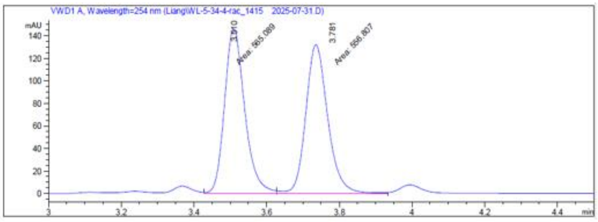

Peak #	RetTime [min]	Type	Width [min]	Area [mAU*s]	Height [mAU]	Area %
1	3.510	FM	0.0639	565.08868	147.49667	50.3691
2	3.781	MF	0.0706	556.80719	131.36194	49.6309

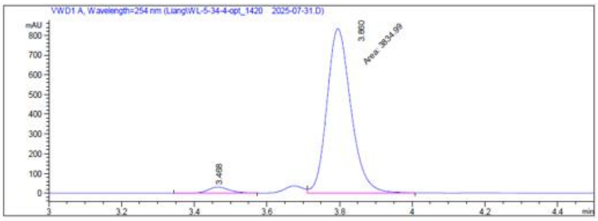

Peak #	RetTime [min]	Type	Width [min]	Area [mAU*s]	Height [mAU]	Area %
1	3.468	BV	0.0777	124.61632	29.95774	3.1472
2	3.860	MF	0.0755	3834.99341	846.58490	96.8528

(*R*,*Z*)-(2-propyl-3-vinylpent-1-en-4-yne-1,5-diyl)dibenzene (40)

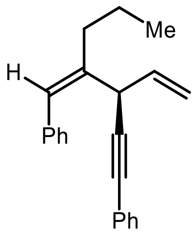

Prepared following **General Procedure D**. Purification by flash column chromatography (silica gel, 5-12% DCM in pentane) gave the title compound (41.8 mg, 73%, >20:1 *Z*/*E*, 99:1 e.r.) as a colorless oil.

R*f* = 0.60 (30:1 pentane: EtOAc, UV)

^{1}H NMR (400 MHz, $CDCl_3$) δ 7.46 – 7.44 (m, 2H), 7.37 – 7.29 (m, 7H), 7.26 – 7.22 (m, 1H), 6.46 (s, 1H), 5.83 (ddd, *J* = 16.8, 10.0, 4.4 Hz, 1H), 5.51 (ddd, *J* = 16.8, 1.6 Hz, 1H), 5.23 (ddd, *J* = 10.0, 1.6 Hz, 1H), 4.69 – 4.67 (m, 1H), 2.35 (dddd, *J* = 15.6, 9.6, 6.0, 1.6 Hz, 1H), 2.24 – 2.16 (m, 1H), 1.74 – 1.62 (m, 2H), 1.01 (t, *J* = 7.2 Hz, 3H).

^{13}C NMR (101 MHz, $CDCl_3$) δ 140.31, 137.87, 136.44, 131.76, 128.87, 128.45, 128.37, 127.96, 126.69, 126.43, 123.87, 116.27, 88.81, 84.56, 37.90, 35.22, 22.00, 14.20.

HRMS (EI) m/z calculated for $C_{22}H_{22}$ [M], 286.1722, found: 286.1714.

Specific rotation $[\alpha]_D^{24}$ = -28.0 (c = 0.40, CH_2Cl_2).

HPLC conditions: Chiral column AD-H, hexane: isopropanol = 98:2, flow rate = 1.0 mL/min, wavelength = 254 nm, t_R = 3.8 min for major isomer, t_R = 4.5 min for minor isomer.

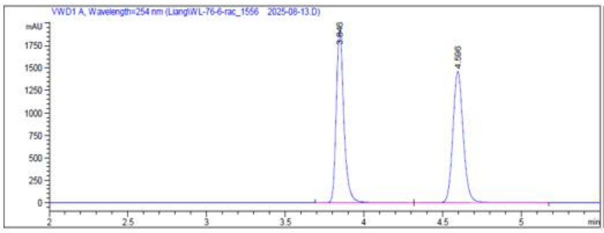

Peak #	RetTime [min]	Type	Width [min]	Area [mAU*s]	Height [mAU]	Area %
1	3.846	BV R	0.0515	6518.03906	1920.05994	49.5341
2	4.596	VB R	0.0702	6640.65820	1460.57678	50.4659

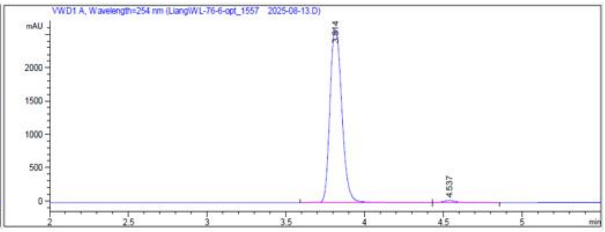

Peak #	RetTime [min]	Type	Width [min]	Area [mAU*s]	Height [mAU]	Area %
1	3.814	BV R	0.0829	1.36747e4	2596.90039	98.8104
2	4.537	VV R	0.0779	164.63666	32.38233	1.1896

(*R*,*Z*)-(2-propyl-3-vinylhept-1-en-4-yne-1,7-diyl)dibenzene (41)

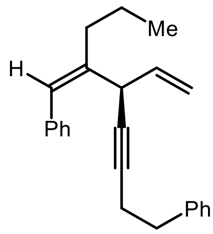

Prepared following **General Procedure D**. Purification by flash column chromatography (silica gel, 5-12% DCM in pentane) gave the title compound (42.1 mg, 67%, >20:1 *Z*/*E*, 98:2 e.r.) as a colorless oil.

R*f* = 0.60 (30:1 pentane: EtOAc, UV)

^{1}H NMR (400 MHz, $CDCl_3$) δ 7.34 – 7.19 (m, 10H), 6.39 (s, 1H), 5.71 (ddd, *J* = 17.2, 10.0, 4.8 Hz, 1H), 5.32 (ddd, *J* = 17.2, 1.6 Hz, 1H), 5.12 (ddd, *J* = 10.0, 1.6 Hz, 1H), 4.48 – 4.35 (m, 1H), 2.85 (t, *J* = 7.6 Hz, 2H), 2.54 (td, *J* = 7.6, 2.0 Hz, 2H), 2.20 (dddd, *J* = 15.6, 9.6, 6.4, 1.6 Hz, 1H), 2.07 (dddd, *J* = 15.6, 8.8, 6.4, 1.6 Hz, 1H), 1.64 – 1.54 (m, 2H), 0.96 (t, *J* = 7.2 Hz, 3H).

^{13}C NMR (101 MHz, $CDCl_3$) δ 141.01, 140.93, 137.97, 136.97, 128.85, 128.65, 128.46, 128.36, 126.52, 126.32, 125.70, 115.83, 83.74, 79.69, 37.37, 35.54, 34.86, 21.85, 21.20, 14.20.

HRMS (EI) m/z calculated for $C_{24}H_{26}$ [M], 314.2035, found: 314.2025.

Specific rotation $[\alpha]_D^{24}$ = -33.9 (c = 0.28, CH_2Cl_2).

HPLC conditions: Hydroboration/oxidation was conducted following **General Procedure E** for ee determination. Chiral column IA, hexane: isopropanol = 97:3, flow rate = 1.0 mL/min, wavelength = 254 nm, t_R = 4.6 min for major isomer, t_R = 5.6 min for minor isomer.

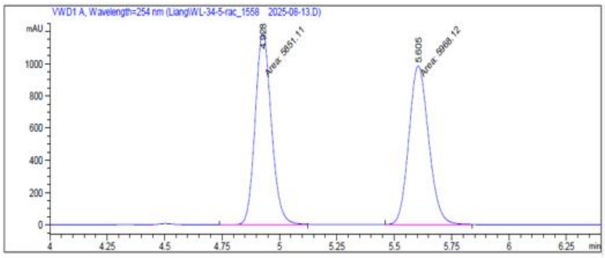

Peak #	RetTime [min]	Type	Width [min]	Area [mAU*s]	Height [mAU]	Area %
1	4.928	MF	0.0819	5851.10840	1190.64526	49.5050
2	5.605	MM	0.1007	5968.11719	987.69647	50.4950

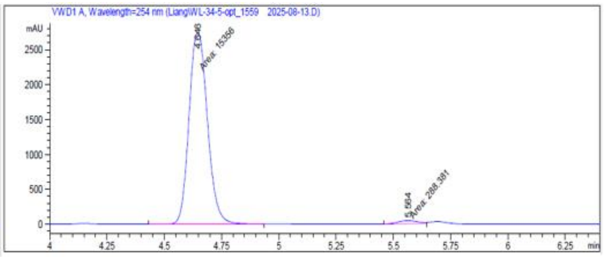

Peak #	RetTime [min]	Type	Width [min]	Area [mAU*s]	Height [mAU]	Area %
1	4.646	MF	0.0933	1.53560e4	2743.14917	98.1566
2	5.564	MM	0.1057	288.38065	45.49248	1.8434

(*R*,*Z*)-(3-methyl-2-propylpenta-1,4-dien-1-yl)benzene (42)

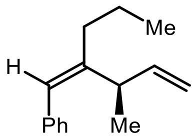

Prepared following **General Procedure C**. Purification by flash column chromatography (silica gel, 2-8% DCM in pentane) gave the title compound (31.2 mg, 78%, 15:1 *rr*, >20:1 *Z*/*E*, 95:5 e.r.) as a colorless oil.

R_f = 0.70 (30:1 pentane: EtOAc, UV)

^{1}H NMR (400 MHz, $CDCl_3$) δ 7.33 – 7.29 (m, 2H), 7.22 – 7.18 (m, 3H), 6.33 (s, 1H), 5.89 (ddd, *J* = 17.2, 10.4, 5.2 Hz, 1H), 5.05 – 4.98 (m, 2H), 3.66 – 3.58 (m, 1H), 2.07 – 2.02 (m, 2H), 1.63 – 1.52 (m, 2H), 1.13 (d, *J* = 7.2 Hz, 3H), 0.98 (t, *J* = 7.2 Hz, 3H).

^{13}C NMR (101 MHz, $CDCl_3$) δ 145.42, 142.28, 138.75, 128.82, 128.20, 126.11, 124.79, 113.66, 38.32, 33.64, 22.27, 17.93, 14.35.

HRMS (EI) m/z calculated for $C_{15}H_{20}$ [M], 200.1565, found: 200.1555.

Specific rotation $[\alpha]_D^{24}$ = +33.3 (c = 0.15, CH_2Cl_2).

HPLC conditions: Hydroboration/oxidation was conducted following **General Procedure E** for ee determination. Chiral column AD-H, hexane: isopropanol = 98:2, flow rate = 1.0 mL/min, wavelength = 254 nm, t_R = 14.1 min for major isomer, t_R = 14.9 min for minor isomer.

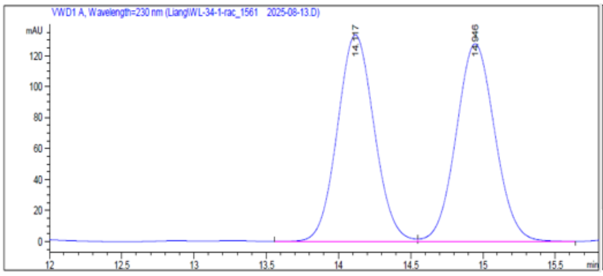

Peak #	RetTime [min]	Type	Width [min]	Area [mAU*s]	Height [mAU]	Area %
1	14.117	BV	0.2736	2359.28271	133.23712	49.8636
2	14.946	VB	0.2884	2372.18896	127.48154	50.1364

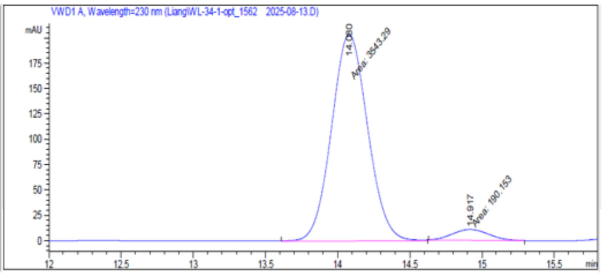

Peak #	RetTime [min]	Type	Width [min]	Area [mAU*s]	Height [mAU]	Area %
1	14.080	MM	0.2898	3543.29492	203.79951	94.9068
2	14.917	MM	0.2930	190.15276	10.81741	5.0932

(*R*,*Z*)-(2-propyl-3-vinylhex-1-en-1-yl)benzene (43)

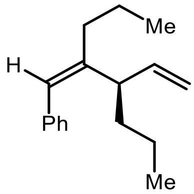

Prepared following **General Procedure C**. Purification by flash column chromatography (silica gel, 2-10% DCM in pentane) gave the title compound (29.6 mg, 65%, >20:1 *Z*/*E*, 99:1 e.r.) as a colorless oil.

R*f* = 0.70 (30:1 pentane: EtOAc, UV)

^{1}H NMR (400 MHz, $CDCl_3$) δ 7.32 – 7.28 (m, 2H), 7.21 – 7.17 (m, 3H), 6.38 (s, 1H), 5.86 (ddd, *J* = 17.2, 10.4, 6.4 Hz, 1H), 5.15 – 4.97 (m, 2H), 3.46 – 3.39 (m, 1H), 2.07 – 2.02 (m, 2H), 1.63 – 1.55 (m, 2H), 1.48 – 1.38 (m, 2H), 1.26 – 1.12 (m, 2H), 0.99 (t, *J* = 7.2 Hz, 3H), 0.79 (t, *J* = 7.2 Hz, 3H).

^{13}C NMR (101 MHz, $CDCl_3$) δ 144.27, 141.36, 138.95, 128.91, 128.12, 126.03, 125.56, 114.36, 44.42, 34.63, 33.40, 21.99, 20.62, 14.41, 14.21.

HRMS (EI) m/z calculated for $C_{17}H_{24}$ [M], 228.1878, found: 228.1869.

Specific rotation $[\alpha]_D^{24}$ = 41.3 (c = 0.4, CH_2Cl_2).

HPLC conditions: Chiral column IA, hexane: isopropanol = 99:1, flow rate = 1.0 mL/min, wavelength = 254 nm, t_R = 4.0 min for major isomer, t_R = 4.2 min for minor isomer.

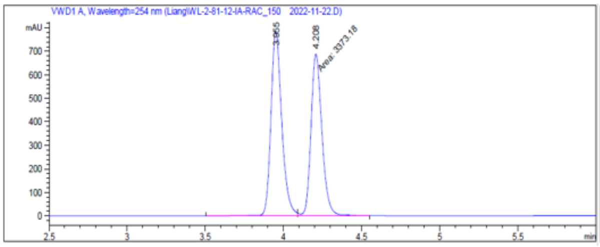

Peak #	RetTime [min]	Type	Width [min]	Area [mAU*s]	Height [mAU]	Area %
1	3.955	BV	0.0862	3783.47046	773.00653	52.8665
2	4.208	MF	0.0804	3373.18481	698.99384	47.1335

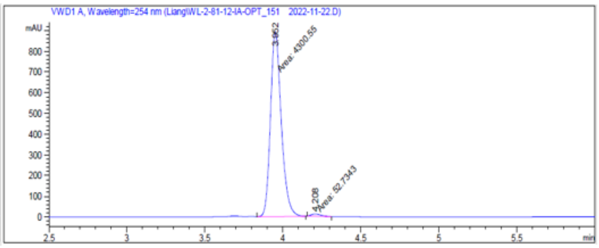

Peak #	RetTime [min]	Type	Width [min]	Area [mAU*s]	Height [mAU]	Area %
1	3.952	MM	0.0790	4300.55371	907.10669	98.7886
2	4.208	MM	0.0758	52.73425	11.59971	1.2114

<u>((3*R*,5*S*,*Z*)-5,9-dimethyl-2-propyl-3-vinyldeca-1,8-dien-1-yl)benzene (44)</u>

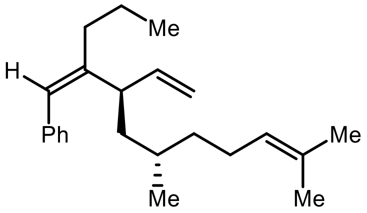

Prepared following **General Procedure C**. Purification by flash column chromatography (silica gel, 2-10% DCM in pentane) gave the title compound (50.2 mg, 81%, >20:1 d.r.) as a colorless oil.).

R*f* = 0.70 (20:1 pentane: EtOAc, UV)

^{1}H NMR (400 MHz, $CDCl_3$) δ 7.31 – 7.27 (m, 2H), 7.20 – 7.16 (m, 3H), 6.40 (s, 1H), 5.89 (ddd, *J* = 16.8, 10.4, 6.4 Hz, 1H), 5.07 – 5.00 (m, 3H), 3.57 – 3.51 (m, 1H), 2.08 – 1.97 (m, 2H), 1.93 – 1.87 (m, 2H), 1.67 (d, *J* = 1.2 Hz, 3H), 1.63 – 1.54 (m, 3H), 1.57 (s, 3H), 1.33 – 1.23 (m, 1H), 1.22 – 1.05 (m, 3H), 0.98 (t, *J* = 7.2 Hz, 3H), 0.54 (d, *J* = 6.4 Hz, 3H).

^{13}C NMR (101 MHz, $CDCl_3$) δ 143.80, 141.94, 138.93, 131.16, 128.85, 128.10, 126.09, 125.94, 125.11, 114.13, 42.05, 39.55, 37.76, 33.15, 29.98, 25.84, 25.54, 21.89, 19.52, 17.78, 14.42.

HRMS (EI) m/z calculated for $C_{23}H_{34}$ [M], 310.2661, found: 310.2650.

Specific rotation $[\alpha]_D^{24}$ = +36.2 (c = 0.65, CH_2Cl_2).

10-((*S*,*Z*)-3-(4-methoxyphenyl)-1-phenyl-2-propylpenta-1,4-dien-1-yl)-9-oxa-10-borabicyclo[3.3.2]decane (45a)

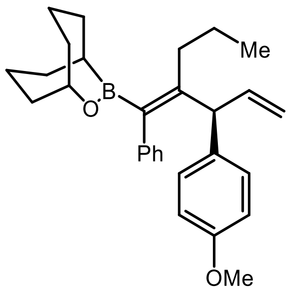

Prepared following **Procedure 2.4.1**. Purification by flash column chromatography (aluminum oxide neutral, 10-20% DCM in pentane) gave the title compound (82.7 mg, 88%, >20:1 *Z*/*E*).

R*f* = 0.40 (20:1 pentane: EtOAc, UV)

^{1}H NMR (400 MHz, $CDCl_3$) δ 7.29 – 7.26 (m, 2H), 7.18 – 7.13 (m, 3H), 7.07 (d, *J* = 8.4 Hz, 2H), 6.79 (d, *J* = 8.4 Hz, 2H), 6.08 (ddd, *J* = 17.2, 10.0, 7.2 Hz, 1H), 5.14 (ddd, *J* = 10.0, 2.0, 1.2 Hz, 1H), 5.02 (ddd, *J* = 17.2, 2.0, 1.2 Hz, 1H), 4.63 (tt, *J* = 5.6, 3.2 Hz, 1H), 4.28 (d, *J* = 7.2 Hz, 1H), 3.77 (s, 3H), 2.18 – 2.02 (m, 2H), 1.87 – 1.71 (m, 5H), 1.68 – 1.60 (m, 2H), 1.50 – 1.30 (m, 7H), 1.08 – 0.96 (m, 1H), 0.77 (t, *J* = 7.2 Hz, 3H).

^{13}C NMR (101 MHz, $CDCl_3$) δ 157.94, 145.99, 143.20, 139.63, 135.26, 129.22, 128.76, 128.11, 125.55, 116.10, 113.54, 73.85, 55.37, 51.47, 35.26, 31.90, 31.56, 27.07, 26.98, 26.48, 22.39, 22.23, 14.99.

HRMS (ESI) m/z calculated for $C_{29}H_{38}BO_2$ $[M+H]^+$, 429.2959, found: 429.2947.

Specific rotation $[\alpha]_D^{24}$ = -17.0 (c = 0.70, CH_2Cl_2).

(*S,E*)-5-(3-(4-methoxyphenyl)-1-phenyl-2-propylpenta-1,4-dien-1-yl)-2-methylpyridine (46)

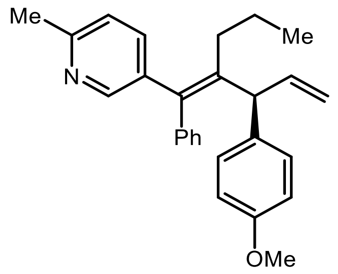

Prepared following **Procedure 2.4.2**. Purification by flash column chromatography (silica gel, 3-15% EtOAc in pentane) gave the title compound (57.0 mg, 74%, >20:1 *E*/*Z*, 99:1 e.r.).

R*f* = 0.30 (10:1 pentane: EtOAc, UV)

^{1}H NMR (400 MHz, $CDCl_3$) δ 8.43 (s, *J* = 1.2 Hz, 1H), 7.40 (dd, *J* = 8.0, 2.4 Hz, 1H), 7.31 – 7.18 (m, 5H), 7.14 (d, *J* = 8.4 Hz, 2H), 7.07 (d, *J* = 8.4 Hz, 1H), 6.84 (d, *J* = 8.4 Hz, 2H), 6.15 (ddd, *J* = 17.2, 10.4, 7.2 Hz, 1H), 5.26 (ddd, *J* = 10.4, 1.6 Hz, 1H), 5.13 (ddd, *J* = 17.2, 1.6 Hz, 1H), 4.52 (d, *J* = 7.2 Hz, 1H), 3.79 (s, 3H), 2.52 (s, 3H), 2.05 (ddd, *J* = 13.6, 11.6, 5.2 Hz, 1H), 1.89 (ddd, *J* = 13.6, 11.6, 5.2 Hz, 1H), 1.22 – 1.09 (m, 1H), 0.89 – 0.80 (m, 1H), 0.54 (t, *J* = 7.2 Hz, 3H).

^{13}C NMR (101 MHz, $CDCl_3$) δ 158.22, 156.30, 149.04, 142.73, 142.18, 138.94, 136.99, 136.93, 135.92, 134.62, 129.09, 129.04, 128.52, 126.83, 122.91, 116.72, 113.77, 55.40, 51.59, 32.52, 24.28, 23.60, 14.77.

HRMS (ESI) m/z calculated for $C_{27}H_{30}NO$ $[M+H]^+$, 384.2322, found: 384.2313.

Specific rotation $[\alpha]_D^{24}$ = -9.0 (c = 1.50, CH_2Cl_2).

HPLC conditions: Chiral column IA, hexane: isopropanol = 98:2, flow rate = 1.0 mL/min, wavelength = 254 nm, t_R = 5.3 min for major isomer, t_R = 5.0 min for minor isomer.

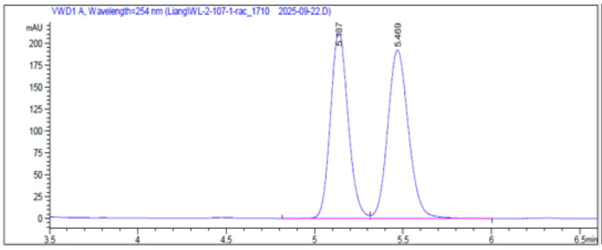

Peak #	RetTime [min]	Type	Width [min]	Area [mAU*s]	Height [mAU]	Area %
1	5.137	BV	0.1074	1477.62256	213.20860	48.5375
2	5.469	VB	0.1260	1566.66809	192.17694	51.4625

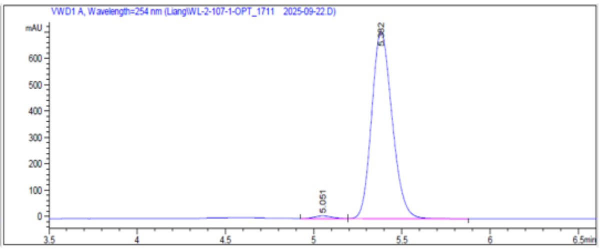

Peak #	RetTime [min]	Type	Width [min]	Area [mAU*s]	Height [mAU]	Area %
1	5.051	BV	0.1031	67.08279	10.03579	1.1592
2	5.382	VB	0.1251	5719.91895	708.68225	98.8408

(*S*,*E*)-4-(3-(4-methoxyphenyl)-1-phenyl-2-propylpenta-1,4-dien-1-yl)-1-methyl-1H-indole (47)

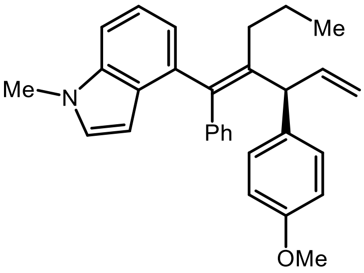

Prepared following **Procedure 2.4.2**. Purification by flash column chromatography (silica gel, 5-10% EtOAc in pentane) gave the title compound (68.1 mg, 81%, >20:1 *E*/*Z*).

R*f* = 0.40 (10:1 pentane: EtOAc, UV)

^{1}H NMR (400 MHz, $CDCl_3$) δ 7.40 (d, *J* = 6.8 Hz, 2H), 7.25 – 7.22 (m, 3H), 7.18 – 7.16 (m, 2H), 7.14 – 7.12 (m, 1H), 7.02 – 7.00 (m, 2H), 6.87 (d, *J* = 8.8 Hz, 2H), 6.46 (d, *J* = 3.2 Hz, 1H), 6.26 (ddd, *J* = 17.2, 10.4, 7.6 Hz, 1H), 5.31 (ddd, *J* = 10.4, 2.0, 1.2 Hz, 1H), 5.25 (ddd, *J* = 17.2, 2.0, 1.2 Hz, 1H), 4.70 (d, *J* = 7.6 Hz, 1H), 3.81 (s, 3H), 3.76 (s, 3H), 2.00 (ddd, *J* = 13.2, 11.6, 5.2 Hz, 1H), 1.81 (ddd, *J* = 13.2, 11.6, 4.8 Hz, 1H), 1.19 – 1.08 (m, 1H), 0.88 – 0.78 (m, 1H), 0.41 (t, *J* = 7.2 Hz, 3H).

^{13}C NMR (101 MHz, $CDCl_3$) δ 158.03, 143.40, 140.70, 139.56, 138.98, 136.99, 136.01, 135.40, 129.13, 128.94, 128.65, 128.11, 127.82, 127.28, 126.30, 120.00, 116.42, 114.10, 107.65, 100.69, 55.38, 51.51, 33.10, 33.06, 23.73, 14.86.

HRMS (ESI) m/z calculated for $C_{30}H_{32}NO$ $[M+H]^+$, 422.2478, found: 422.2470.

Specific rotation $[\alpha]_D^{24}$ = +7.1 (c = 1.05, CH_2Cl_2).

(*S*,*Z*)-1-methoxy-4-(5-(3-phenylpropyl)-4-propylnona-1,4-dien-3-yl)benzene (48)

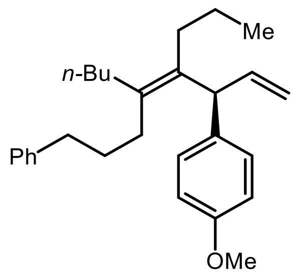

Prepared following **Procedure 2.4.2**. Purification by flash column chromatography (silica gel, 0-12% DCM in pentane) gave the title compound (63.2 mg, 81%, >20:1 *Z*/*E*).

R*f* = 0.75 (20:1 pentane: EtOAc, $KMnO_4$)

^{1}H NMR (400 MHz, $CDCl_3$) δ 7.29 – 7.25 (m, 2H), 7.21 – 7.11 (m, 3H), 7.09 (d, *J* = 8.4 Hz, 2H), 6.82 (d, *J* = 8.4 Hz, 2H), 6.07 (ddd, *J* = 17.2, 10.0, 7.2 Hz, 1H), 5.13 (ddd, *J* = 10.0, 2.0, 1.2 Hz, 1H), 5.02 (ddd, *J* = 17.2, 2.0, 1.2 Hz, 1H), 4.39 (d, *J* = 7.2 Hz, 1H), 3.80 (s, 3H), 2.62 – 2.53 (m, 2H), 2.16 – 1.96 (m, 4H), 1.95 – 1.75 (m, 2H), 1.75 – 1.64 (m, 2H), 1.44 – 1.30 (m, 4H), 1.26 – 1.14 (m, 1H), 0.96 – 0.86 (m, 1H), 0.92 (t, *J* = 7.2 Hz, 3H), 0.73 (t, *J* = 7.2 Hz, 3H).

^{13}C NMR (101 MHz, $CDCl_3$) δ 157.94, 142.73, 139.85, 136.43, 135.52, 134.16, 129.16, 128.52, 128.40, 125.79, 115.69, 113.55, 55.37, 50.53, 36.50, 32.88, 32.20, 31.42, 31.39, 31.33, 23.90, 23.34, 14.90, 14.25.

HRMS (EI) m/z calculated for $C_{28}H_{38}O$ [M], 390.2923, found: 390.2914.

Specific rotation $[\alpha]_D^{24}$ = -14.8 (c = 0.65, CH_2Cl_2).

(*S*,*E*)-(5-(4-methoxyphenyl)-3-phenyl-4-propylhepta-3,6-dien-1-yn-1-yl)trimethylsilane (49)

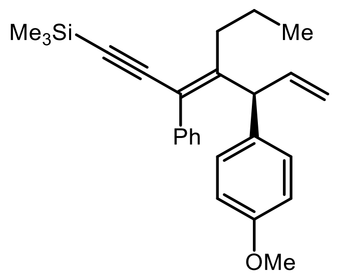

Prepared following **Procedure 2.4.3**. Purification by flash column chromatography (silica gel, 5-12% DCM in pentane) gave the title compound (61.6 mg, 79%, >20:1 *E*/*Z*, 99:1 e.r.).

R$_f$ = 0.65 (20:1 pentane: EtOAc, UV)

^{1}H NMR (400 MHz, $CDCl_3$) δ 7.38 – 7.32 (m, 4H), 7.30 – 7.25 (m, 1H), 7.02 (d, *J* = 8.4 Hz, 2H), 6.81 (d, *J* = 8.4 Hz, 2H), 6.09 (ddd, *J* = 17.2, 10.4, 6.8 Hz, 1H), 5.25 (ddd, *J* = 10.4, 1.6 Hz, 1H), 5.07 (ddd, *J* = 17.2, 1.6 Hz, 1H), 4.51 (d, *J* = 6.8 Hz, 1H), 3.78 (s, 3H), 2.28 – 2.24 (m, 2H), 1.53 – 1.44 (m, 1H), 1.13 – 1.00 (m, 1H), 0.82 (t, *J* = 7.2 Hz, 3H), 0.18 (s, 9H).

^{13}C NMR (101 MHz, $CDCl_3$) δ 158.33, 153.04, 139.01, 138.37, 133.90, 129.24, 129.03, 128.37, 127.27, 121.82, 117.15, 113.74, 105.62, 98.73, 55.37, 50.88, 35.22, 22.98, 15.05, 0.21.

HRMS (ESI) m/z calculated for $C_{26}H_{33}OSi$ $[M+H]^+$, 389.2295, found: 389.2291.

Specific rotation $[\alpha]_D^{24}$ = -12.0 (c = 0.80, CH_2Cl_2).

HPLC conditions: Chiral column AD-H, hexane: isopropanol = 99:1, flow rate = 1.0 mL/min, wavelength = 254 nm, t_R = 6.2 min for major isomer, t_R = 5.8 min for minor isomer.

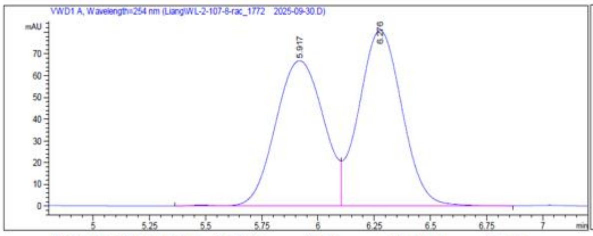

Peak #	RetTime [min]	Type	Width [min]	Area [mAU*s]	Height [mAU]	Area %
1	5.917	VV R	0.2350	989.28967	66.82849	47.8827
2	6.276	VB	0.2076	1076.77856	80.75970	52.1173

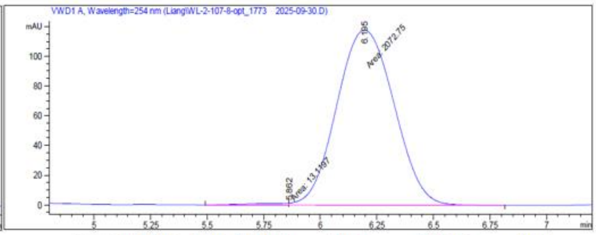

Peak #	RetTime [min]	Type	Width [min]	Area [mAU*s]	Height [mAU]	Area %
1	5.862	MF	0.1683	13.11970	1.29954	0.6290
2	6.195	FM	0.2933	2072.74756	117.79943	99.3710

<u>(*R*,*Z*)-1-methoxy-4-(4-(phenylmethylene-*d*)hept-1-en-3-yl)benzene (50)</u>

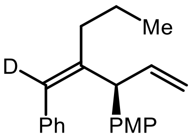

Prepared following **General Procedure C** except to quench the reaction with acetic acid-*d4* instead of AcOH. Purification by flash column chromatography (silica gel, 5-15% DCM in pentane) gave the title compound (51.1 mg, 87%, >20:1 *Z*/*E*, 98:2 e.r.) as a colorless oil.

R$_f$ = 0.45 (30:1 pentane: EtOAc, UV)

^{1}H NMR (400 MHz, $CDCl_3$) δ 7.36 – 7.24 (m, 4H), 7.26 – 7.16 (m, 1H), 7.09 (d, *J* = 8.4 Hz, 2H), 6.83 (d, *J* = 8.4 Hz, 2H), 6.18 (ddd, *J* = 17.2, 10.4, 6.8 Hz, 1H), 5.27 (ddd, *J* = 10.4, 1.6 Hz, 1H), 5.16 (ddd, *J* = 17.2, 1.6 Hz, 1H), 4.78 (d, *J* = 6.8 Hz, 1H), 3.79 (s, 3H), 2.03 (ddd, *J*

= 16.0, 10.4, 5.6 Hz, 1H), 1.88 (ddd, J = 16.0, 10.4, 5.6 Hz, 1H), 1.55 – 1.35 (m, 2H), 0.88 (t, J = 7.2 Hz, 3H).

^{13}C NMR (101 MHz, $CDCl_3$) δ 158.12, 143.20, 139.09, 138.39, 134.54, 129.21, 128.69, 128.29, 126.38, 116.84, 113.69, 55.37, 49.45, 34.60, 21.92, 14.31.

HRMS (EI) m/z calculated for $C_{21}H_{23}DO$ [M], 293.1890, found: 293.1880.

Specific rotation $[\alpha]_D^{24}$ = -17.6 (c = 1.0, CH_2Cl_2).

HPLC conditions: Chiral column AD-H, hexane: isopropanol = 99:1, flow rate = 1.0 mL/min, wavelength = 254 nm, t_R = 4.3 min for major isomer, t_R = 5.2 min for minor isomer.

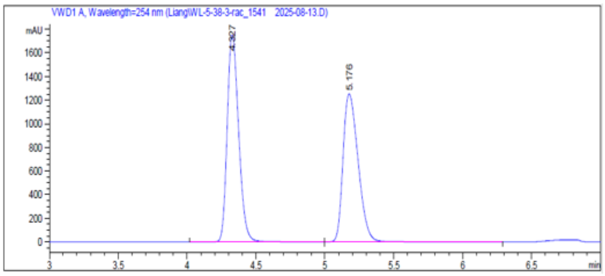

Peak #	RetTime [min]	Type	Width [min]	Area [mAU*s]	Height [mAU]	Area %
1	4.327	VB R	0.0898	1.00967e4	1750.94995	51.4538
2	5.176	BV R	0.1201	9526.14941	1251.71899	48.5462

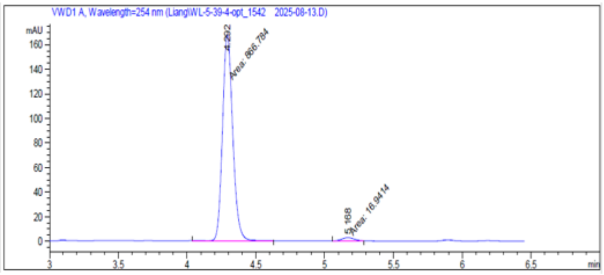

Peak #	RetTime [min]	Type	Width [min]	Area [mAU*s]	Height [mAU]	Area %
1	4.292	FM	0.0861	866.78369	167.73402	98.0830
2	5.168	MM	0.1024	16.94143	2.75768	1.9170

(*S*,*Z*)-1-methoxy-4-(4-(1-phenylethylidene)hept-1-en-3-yl)benzene (51)

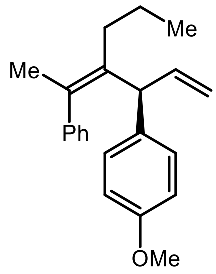

Prepared following **Procedure 2.4.4**. Purification by flash column chromatography (silica gel, 0-12% DCM in pentane) gave the title compound (33.2 mg, 54%, >20:1 *Z*/*E*, 97:3 e.r.).

R_f = 0.65 (20:1 pentane: EtOAc, UV)

^{1}H NMR (400 MHz, $CDCl_3$) δ 7.37 – 7.28 (m, 2H), 7.26 – 7.17 (m, 3H), 7.04 (d, J = 8.8 Hz, 2H), 6.80 (d, J = 8.8 Hz, 2H), 6.07 (ddd, J = 17.2, 10.4, 6.8 Hz, 1H), 5.17 (ddd, J = 10.4, 2.0, 1.2 Hz, 1H), 5.01 (ddd, J = 17.2, 1.6 Hz, 1H), 4.28 (d, J = 6.8 Hz, 1H), 3.78 (s, 3H), 2.07 – 1.94 (m, 2H), 2.01 (s, 3H), 1.38 – 1.24 (m, 1H), 0.98 – 0.86 (m, 1H), 0.78 (t, J = 7.2 Hz, 3H).

^{13}C NMR (101 MHz, $CDCl_3$) δ 157.97, 145.21, 139.59, 136.53, 135.27, 133.85, 129.13, 128.31, 128.16, 126.26, 116.01, 113.51, 55.35, 51.59, 32.34, 23.23, 21.55, 14.91.

HRMS (ES) m/z calculated for $C_{22}H_{26}O$ [M], 306.1984, found: 306.1975.

Specific rotation $[\alpha]_D^{24}$ = -45.2 (c = 0.62, CH_2Cl_2).

HPLC conditions: Chiral column IA, hexane: isopropanol = 98:2, flow rate = 1.0 mL/min, wavelength = 254 nm, t_R = 4.3 min for major isomer, t_R = 4.9 min for major isomer.

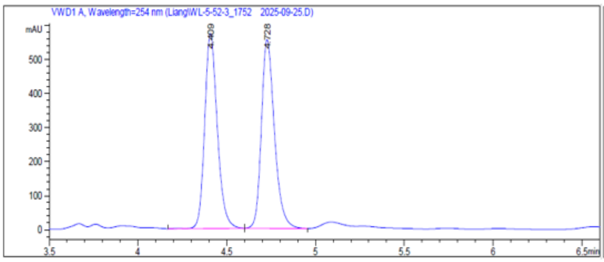

Peak #	RetTime [min]	Type	Width [min]	Area [mAU*s]	Height [mAU]	Area %
1	4.409	VB R	0.0757	2820.23755	572.22101	49.9005
2	4.728	BB	0.0786	2831.48193	553.75671	50.0995

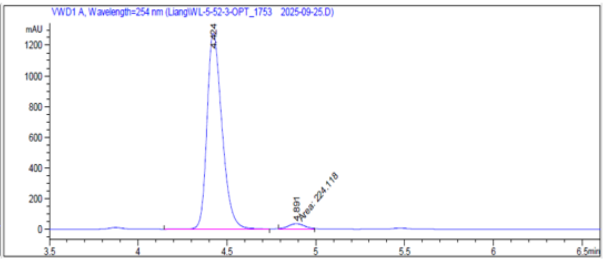

Peak #	RetTime [min]	Type	Width [min]	Area [mAU*s]	Height [mAU]	Area %
1	4.424	VV R	0.0903	7491.79590	1276.41125	97.0954
2	4.891	MM	0.1074	224.11844	34.76826	2.9046

(*S*,*Z*)-4,4'-(4-propylocta-1,4,7-triene-3,5-diyl)bis(methoxybenzene) (52)

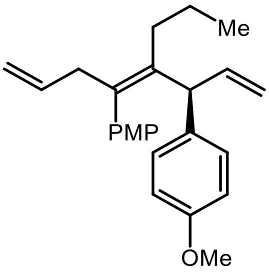

Prepared following **Procedure 2.4.4**. Purification by flash column chromatography (silica gel, 5-20% DCM in pentane) gave the title compound (42.8 mg, 59%, >20:1 *Z*/*E*, 97:3 e.r.).

R_f = 0.55 (20:1 pentane: EtOAc, UV)

^{1}H NMR (500 MHz, $CDCl_3$) δ 7.08 (d, J = 8.8 Hz, 2H), 7.04 (d, J = 8.4 Hz, 2H), 6.85 (d, J = 8.4 Hz, 2H), 6.80 (d, J = 8.8 Hz, 2H), 6.06 (ddd, J = 17.2, 10.4, 6.4 Hz, 1H), 5.72 (ddt, J = 16.8, 10.4, 6.4 Hz, 1H), 5.17 (ddd, J = 10.4, 1.6 Hz, 1H), 5.06 – 4.88 (m, 3H), 4.27 (d, J = 6.8 Hz, 1H), 3.80 (s, 3H), 3.78 (s, 3H), 3.21 – 3.06 (m, 2H), 2.01 (ddd, J = 9.2, 6.0, 2.0 Hz, 2H), 1.37 – 1.23 (m, 1H), 0.94 – 0.83 (m, 1H), 0.77 (t, J = 7.2 Hz, 3H).

^{13}C NMR (126 MHz, $CDCl_3$) δ 158.11, 158.00, 139.62, 137.98, 136.30, 135.75, 135.39, 135.20, 129.97, 129.13, 116.06, 115.54, 113.54, 113.46, 55.35, 55.28, 51.64, 39.47, 32.10, 24.04, 15.00.

HRMS (ESI) m/z calculated for $C_{25}H_{30}O_2$ [M], 362.2246, found: 362.2236.

Specific rotation $[\alpha]_D^{24}$ = -52.6 (c = 0.80, CH_2Cl_2).

HPLC conditions: Chiral column IA, hexane: isopropanol = 98:2, flow rate = 1.0 mL/min, wavelength = 254 nm, t_R = 4.6 min for major isomer, t_R = 5.2 min for major isomer.

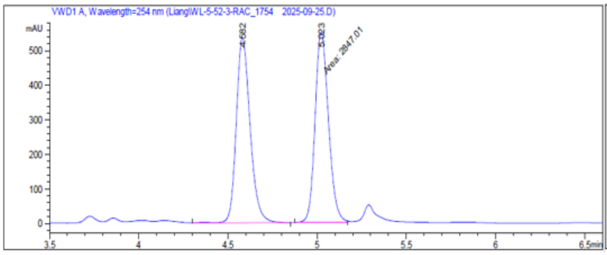

Peak #	RetTime [min]	Type	Width [min]	Area [mAU*s]	Height [mAU]	Area %
1	4.582	VB R	0.0824	2892.37866	536.19415	50.3953
2	5.023	MM	0.0865	2847.00708	548.69958	49.6047

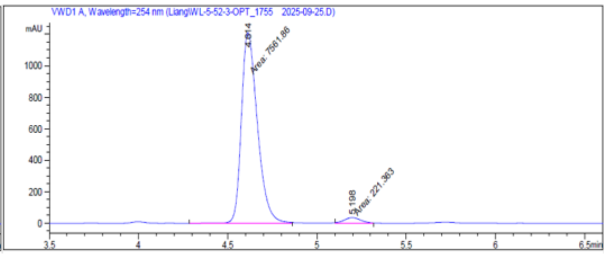

Peak #	RetTime [min]	Type	Width [min]	Area [mAU*s]	Height [mAU]	Area %
1	4.614	MF	0.1040	7561.85693	1211.94788	97.1559
2	5.198	MM	0.1060	221.36336	34.81966	2.8441

3. REFERENCES

1. Weix, D. J.; Marković, D.; Ueda, M.; Hartwig, J. F. Direct, Intermolecular, Enantioselective, Iridium-Catalyzed Allylation of Carbamates to Form Carbamate-Protected, Branched Allylic Amines. *Org. Lett.* **2009**, *11*, 2944-2947.

2. Davis, C. R.; Luvaga, I. K.; Ready, J. M. Enantioselective Allylation of Alkenyl Boronates Promotes a 1,2-Metalate Rearrangement with 1,3-Diastereocontrol. *J. Am. Chem. Soc.* **2021**, *143*, 4921-4927.

3. Fang, G. Y.; Wallner, O. A.; Blasio, N. D.; Ginesta, X.; Harvey, J. N.; Aggarwal, V. K. Asymmetric Sulfur Ylide Reactions with Boranes: Scope and Limitations, Mechanism and Understanding. *J. Am. Chem. Soc.* **2007**, *129*, 14632-14639.

4. Raskatov, J. A.; Spiess, S.; Gnamm, C.; Brödner, K.; Rominger, F.; Helmchen, G. Ir-Catalysed Asymmetric Allylic Substitutions with Cyclometalated (Phosphoramidite)Ir Complexes—Resting States, Catalytically Active (π-Allyl)Ir Complexes and Computational Exploration. *Chem. Eur. J.* **2010**, *16*, 6601-6615.

5. Ishida, N.; Shimamoto, Y.; Murakami, M. Stereoselective Synthesis of (E)-(Trisubstituted alkenyl)borinic Esters: Stereochemistry Reversed by Ligand in the Palladium-Catalyzed Reaction of Alkynylborates with Aryl Halides. *Org. Lett.* **2009**, *11*, 5434-5437.

6. Nishihara, Y.; Okada, Y.; Jiao, J.; Suetsugu, M.; Lan, M.-T.; Kinoshita, M.; Iwasaki, M.; Takagi, K. Highly Regio- and Stereoselective Synthesis of Multialkylated Olefins through Carbozirconation of Alkynylboronates and Sequential Negishi and Suzuki–Miyaura Coupling Reactions. *Angew. Chem. Int. Ed.* **2011**, *50*, 8660-8664.

7. Canterbury, D. P.; Micalizio, G. C. Polyketide Assembly by Alkene−Alkyne Reductive Cross-Coupling: Spiroketals through the Union of Homoallylic Alcohols. *J. Am. Chem. Soc.* **2010**, *132*, 7602-7604.

8. Corey, E. J.; Seibel, W. L. A simple stereoselective synthesis of Z-γ-bisabolene. *Tetrahedron Lett.* **1986**, *27*, 909-910.

4. NMR SPECTRA

^{1}H NMR (400 MHz, $CDCl_3$) of **5**

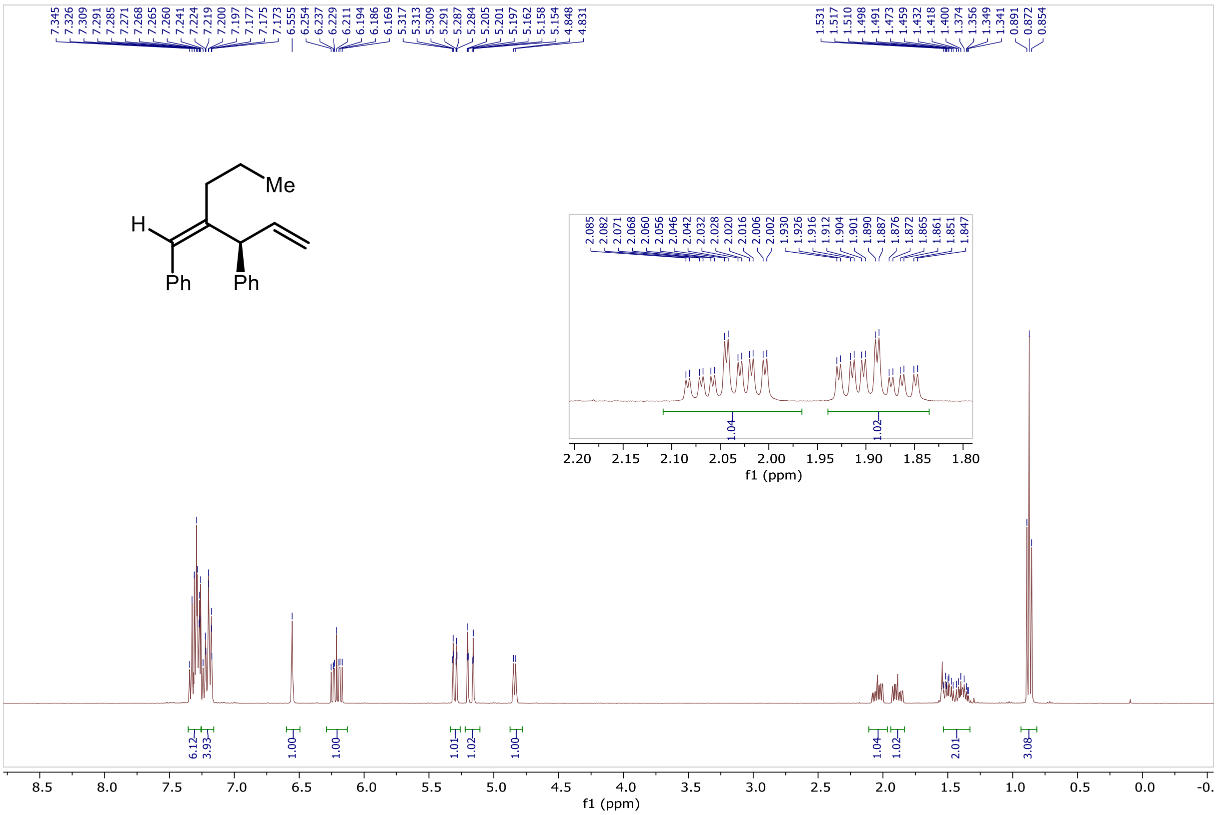

^{13}C NMR (400 MHz, $CDCl_3$) of **5**

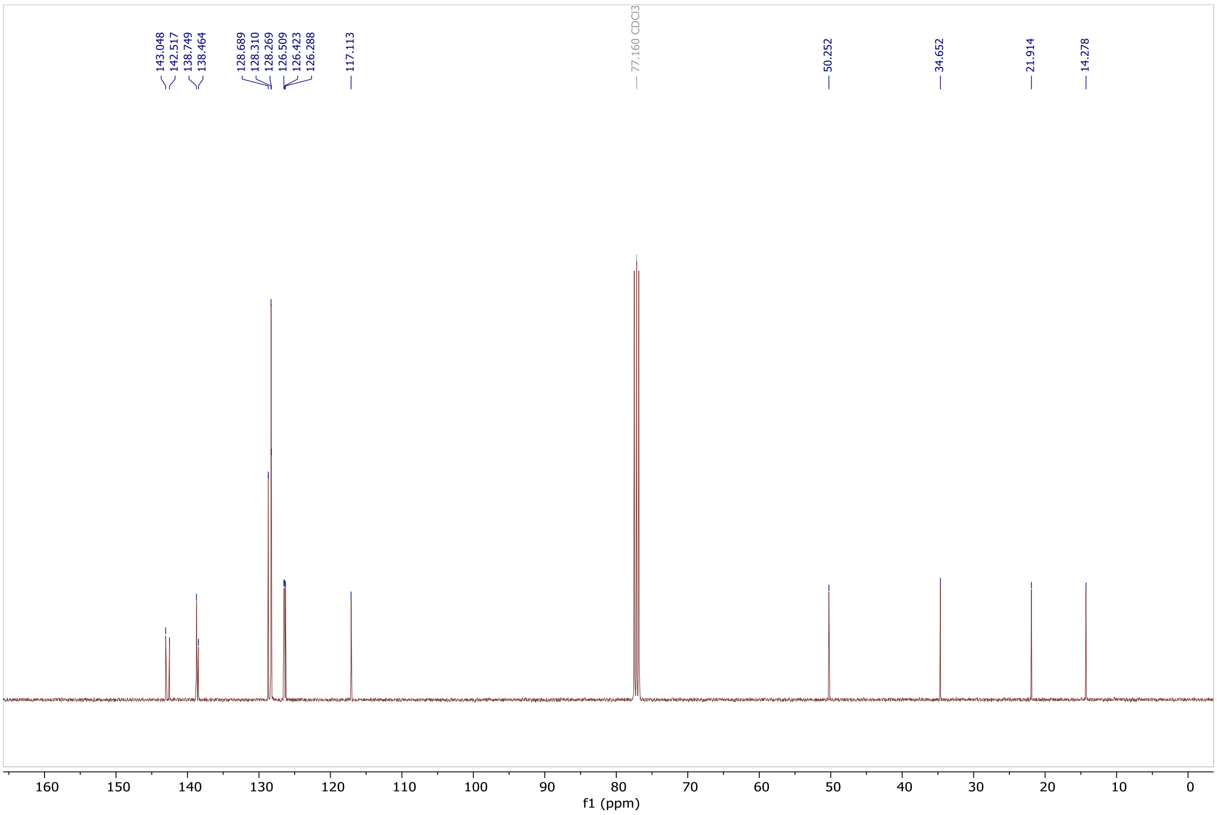

^{1}H NMR (400 MHz, $CDCl_3$) of **6**

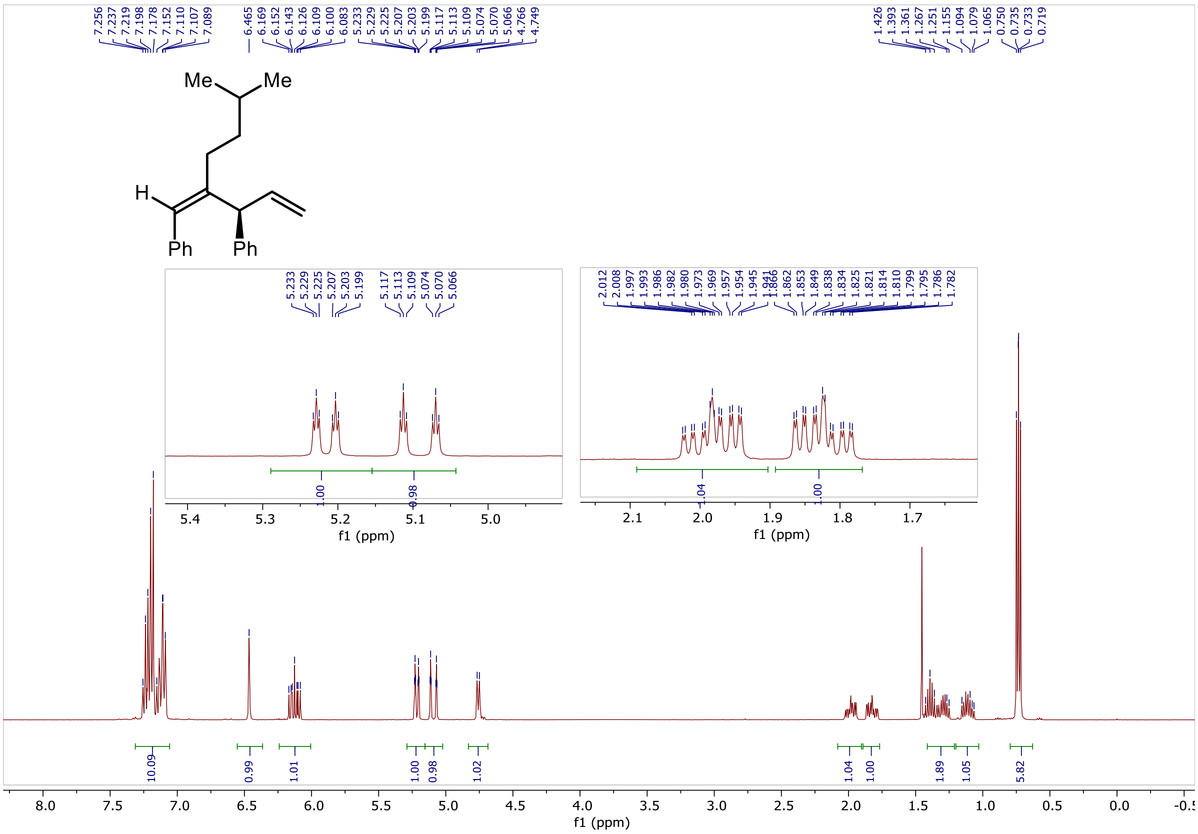

^{13}C NMR (400 MHz, $CDCl_3$) of **6**

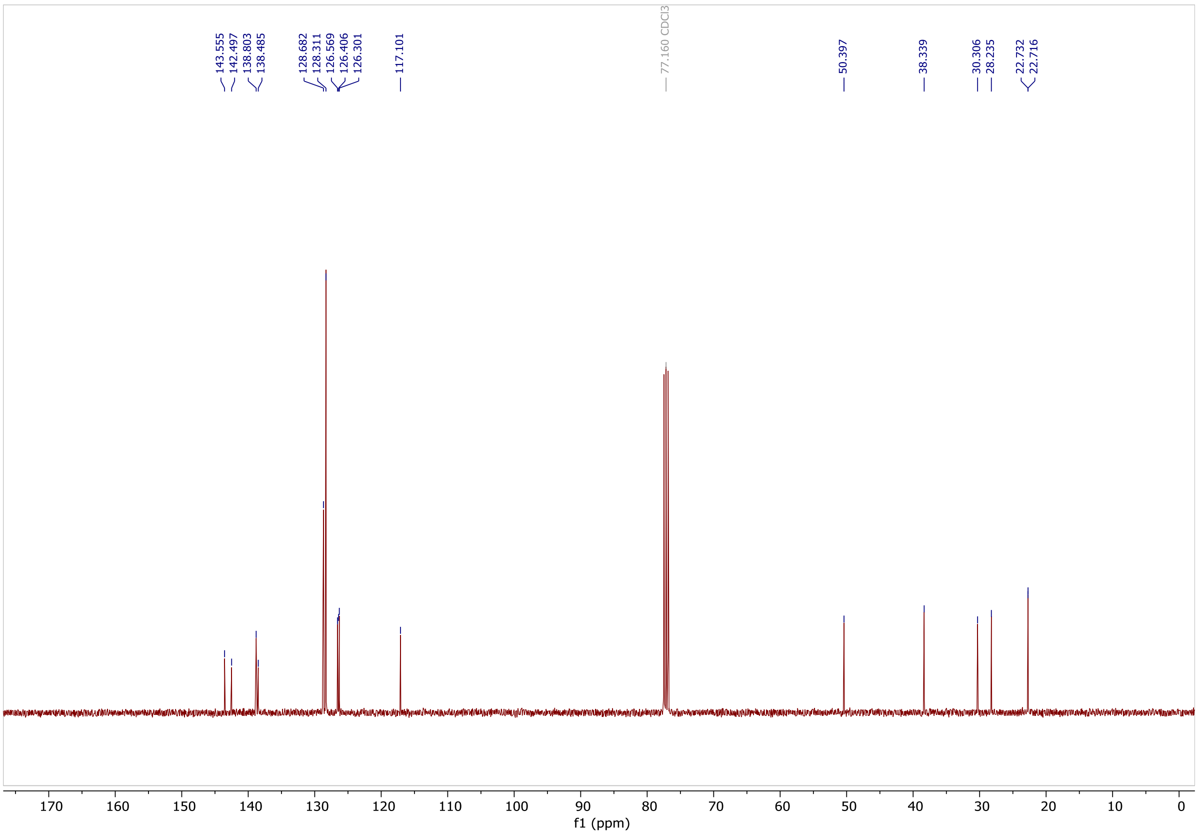

^{1}H NMR (400 MHz, $CDCl_3$) of **7**

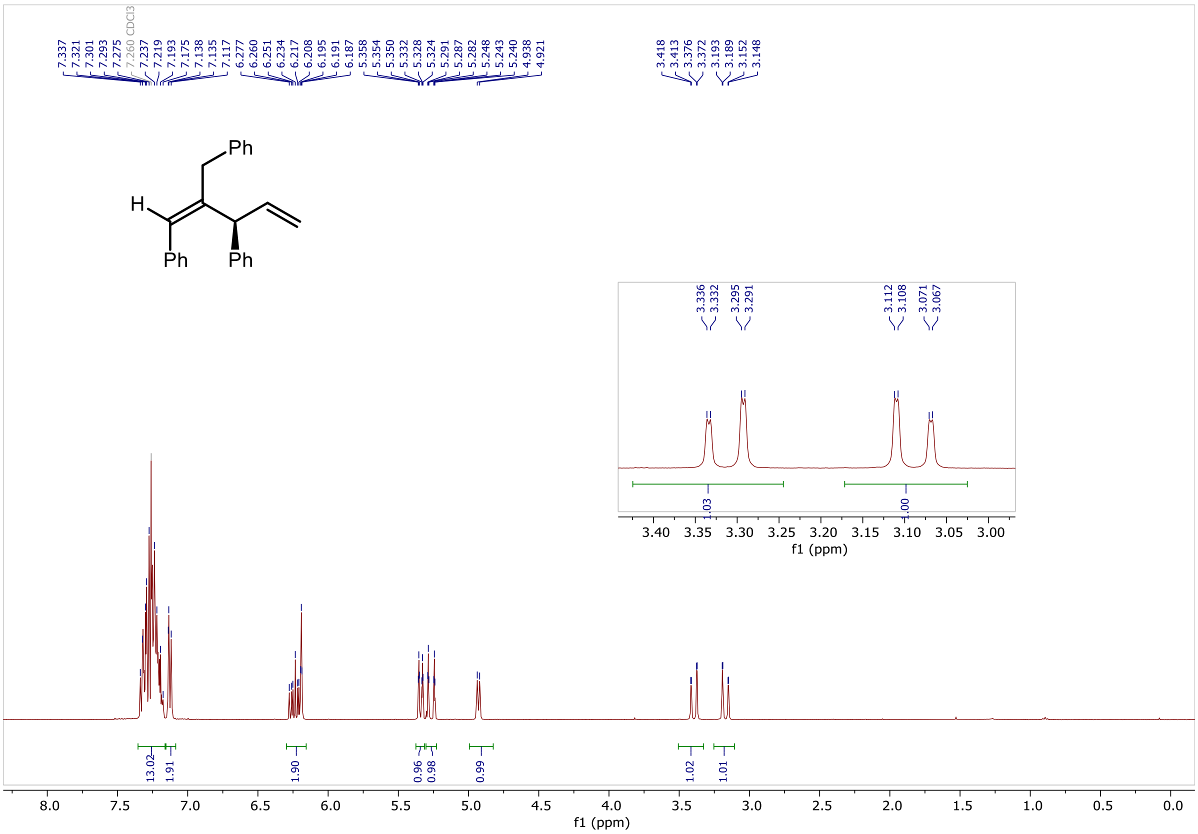

^{13}C NMR (400 MHz, $CDCl_3$) of **7**

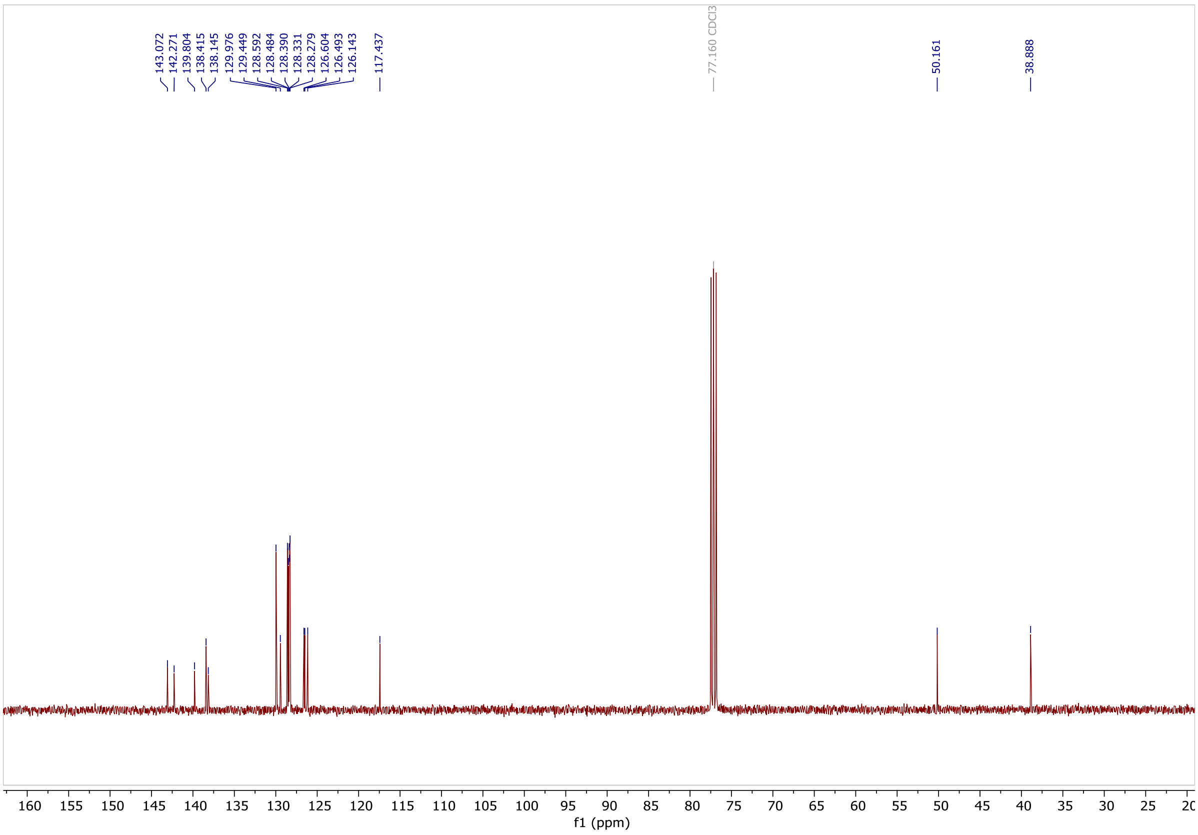

^{1}H NMR (400 MHz, $CDCl_3$) of **8**

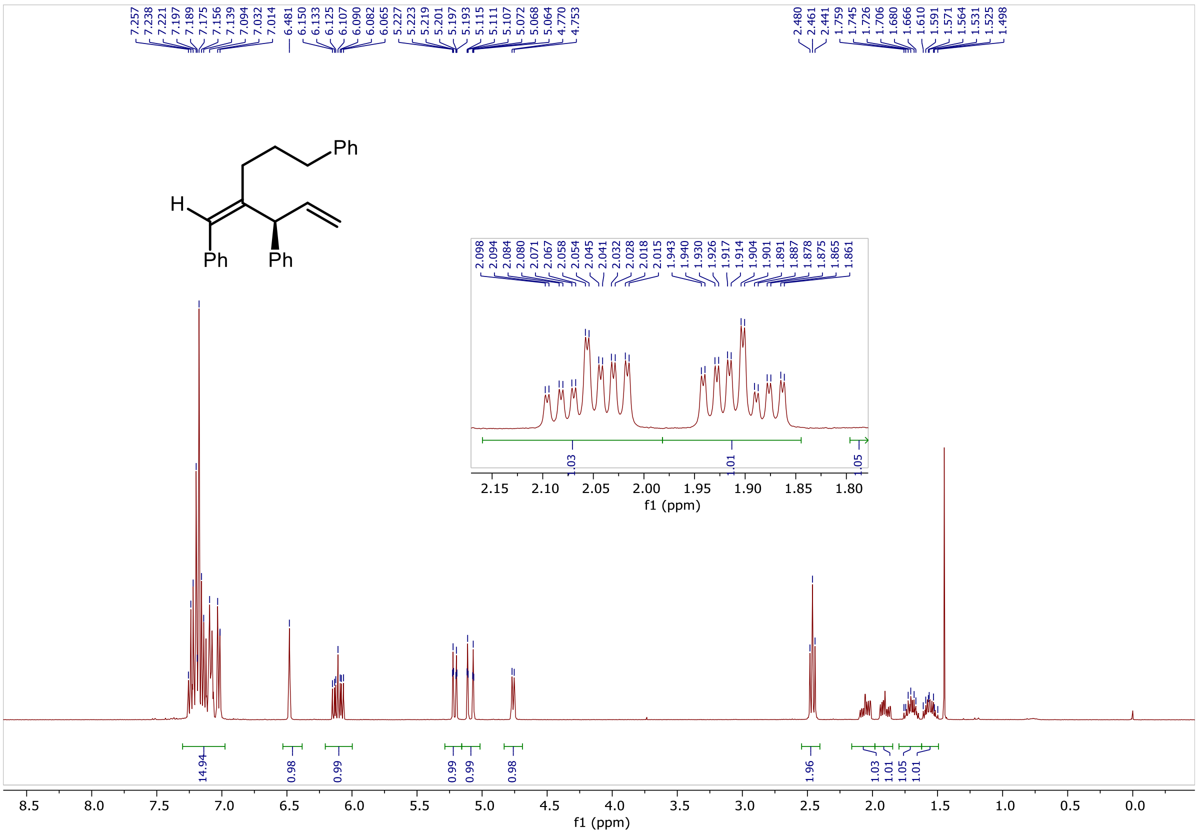

^{13}C NMR (400 MHz, $CDCl_3$) of **8**

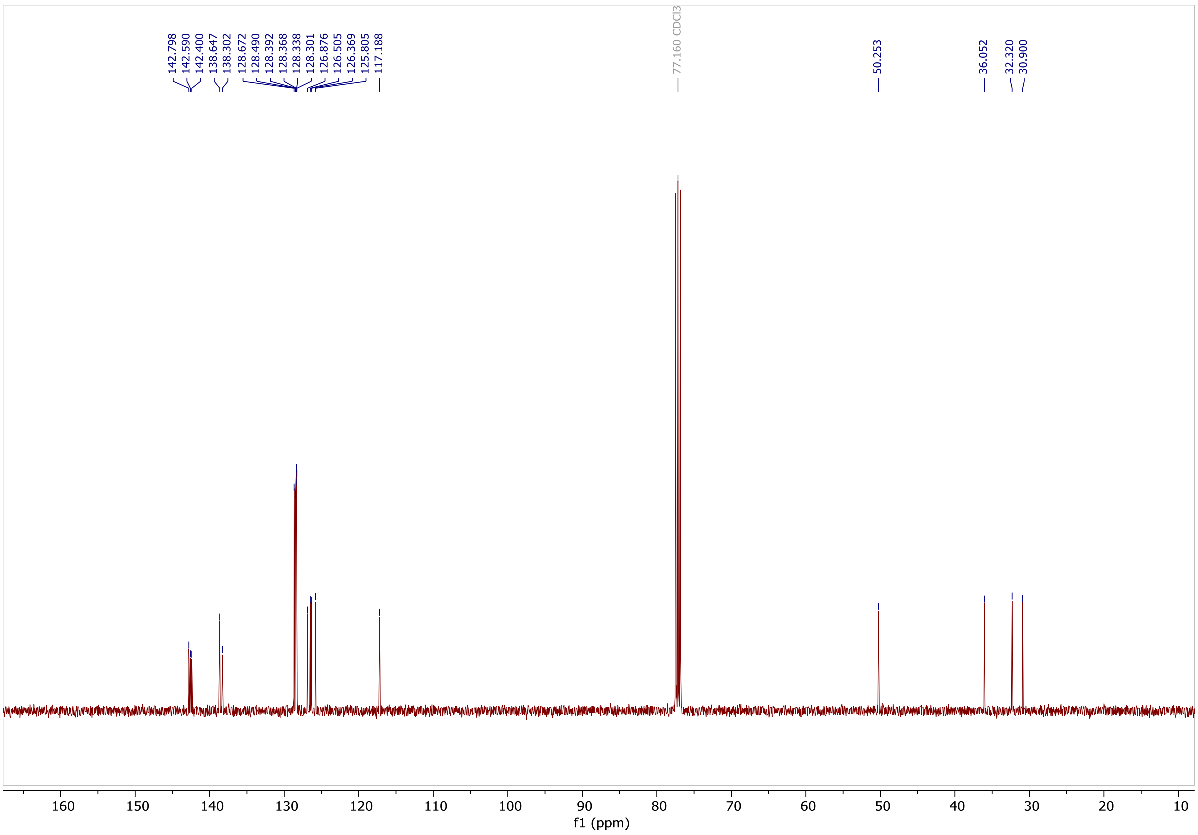

^{1}H NMR (400 MHz, $CDCl_3$) of **9**

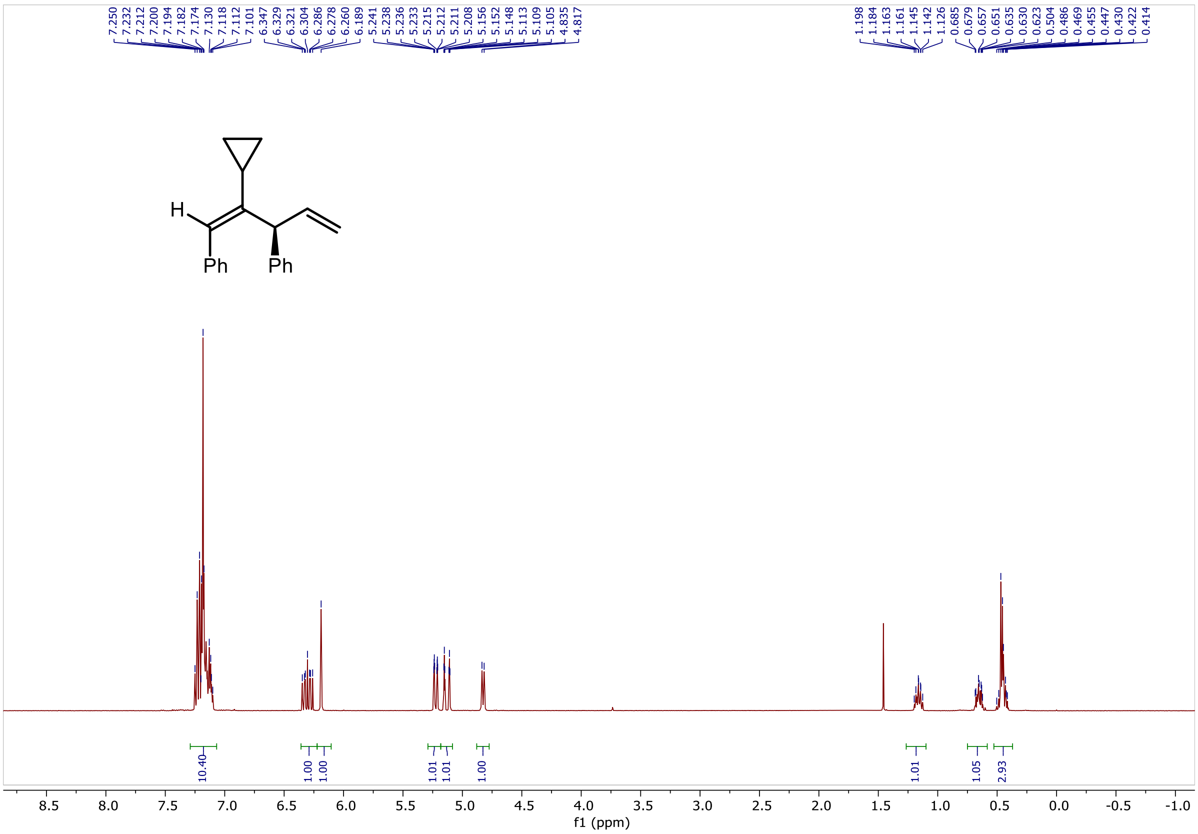

^{13}C NMR (400 MHz, $CDCl_3$) of **9**

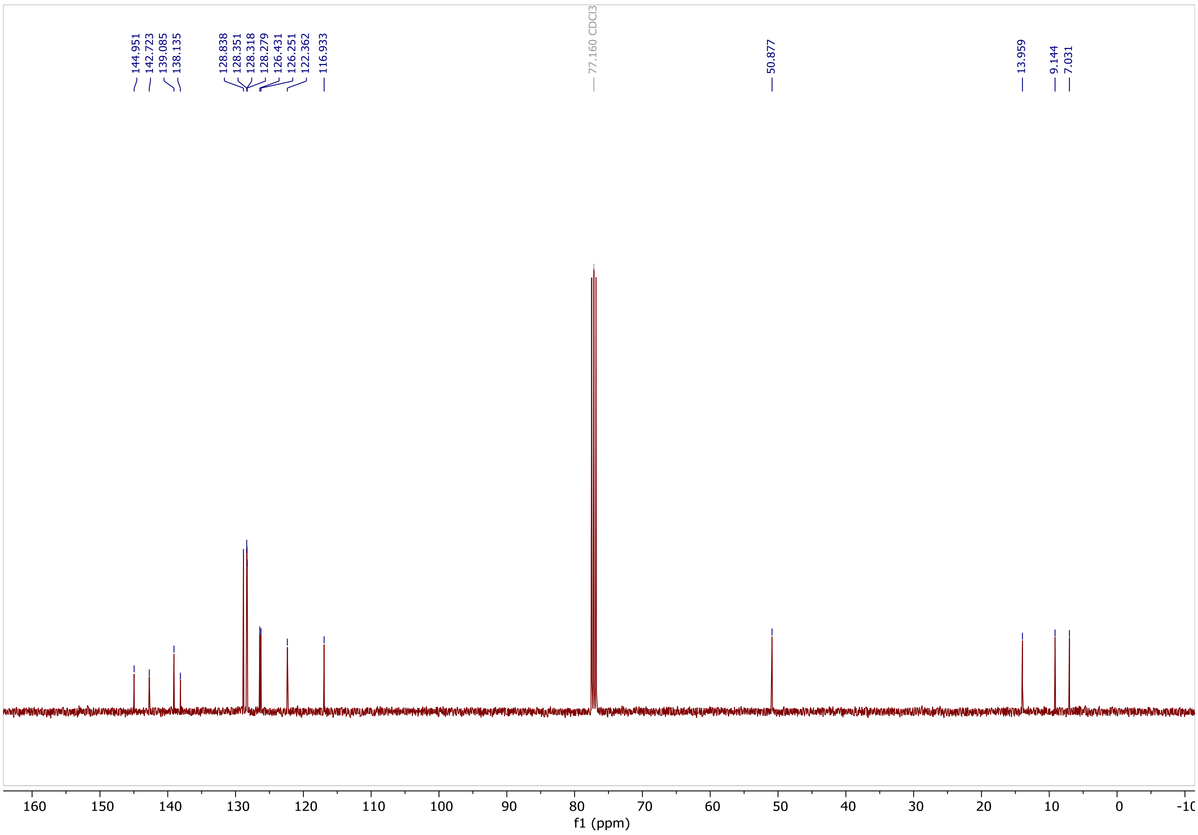

^{1}H NMR (400 MHz, $CDCl_3$) of **10**

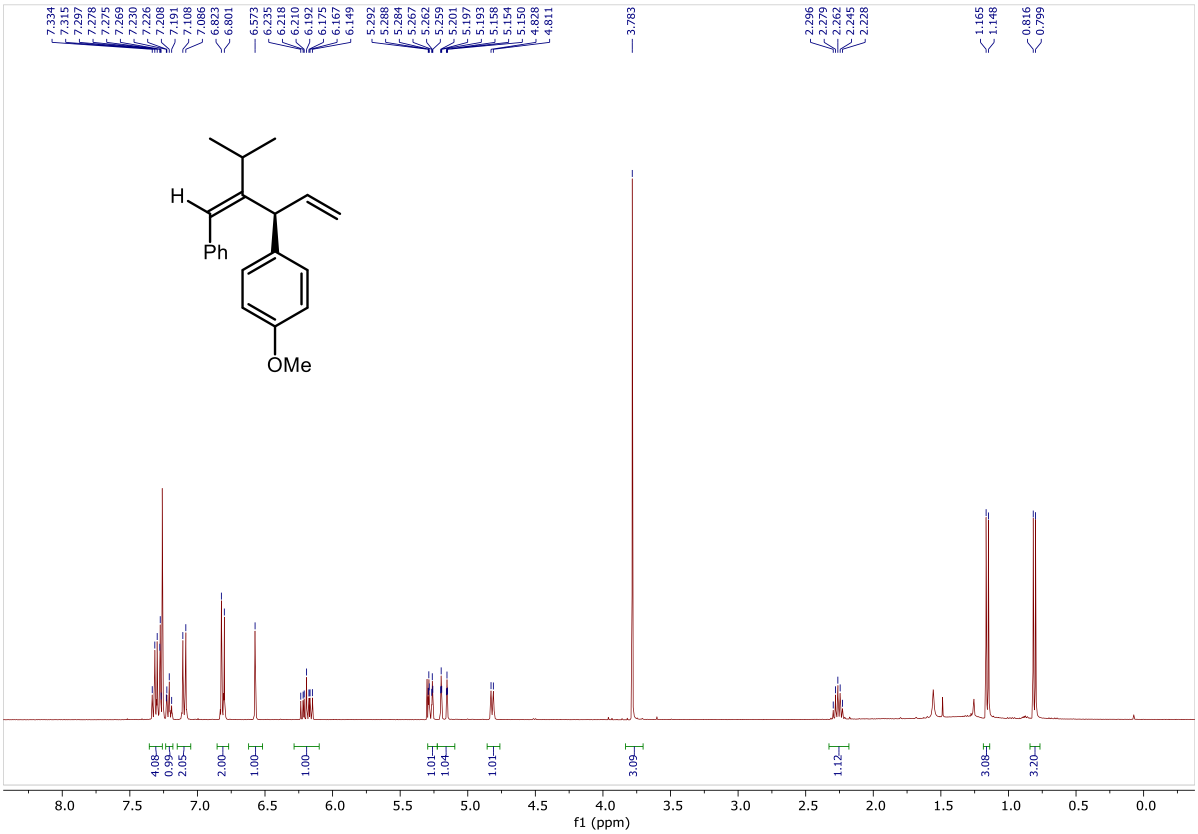

^{13}C NMR (400 MHz, $CDCl_3$) of **10**

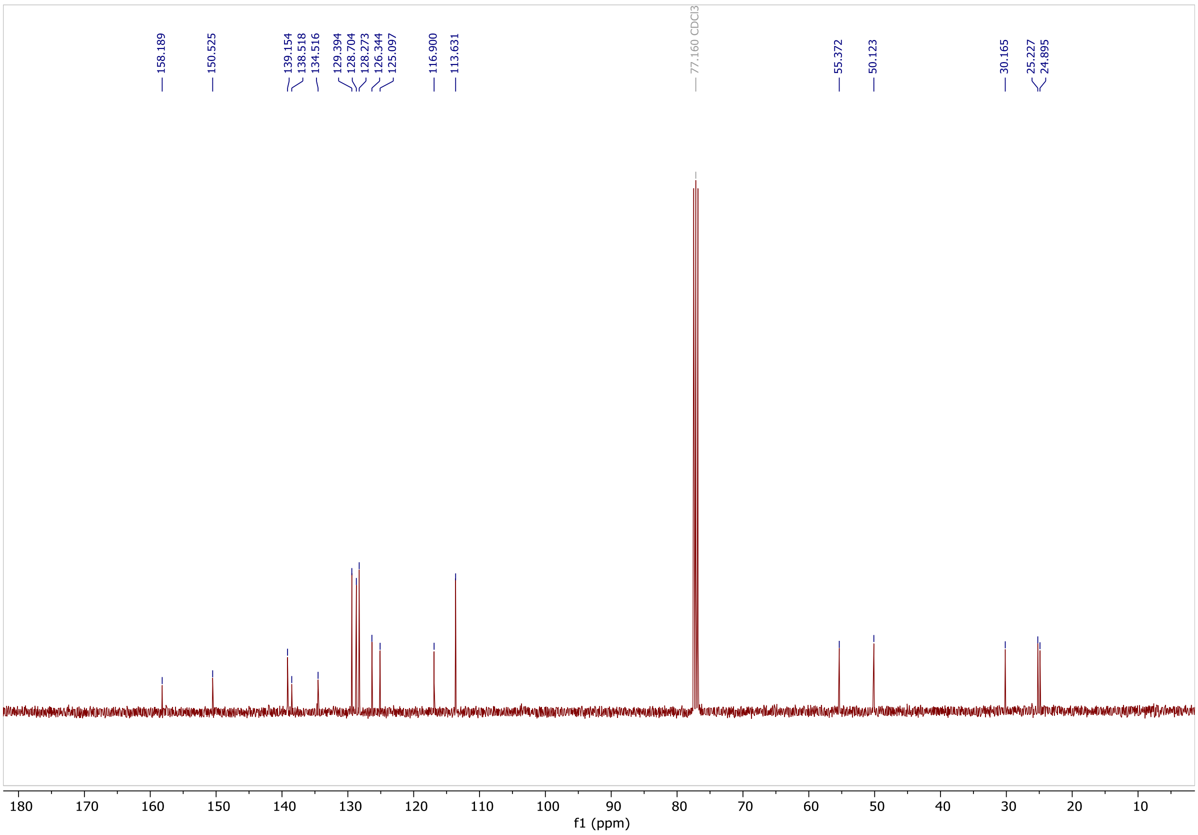

^{1}H NMR (400 MHz, $CDCl_3$) of **11**

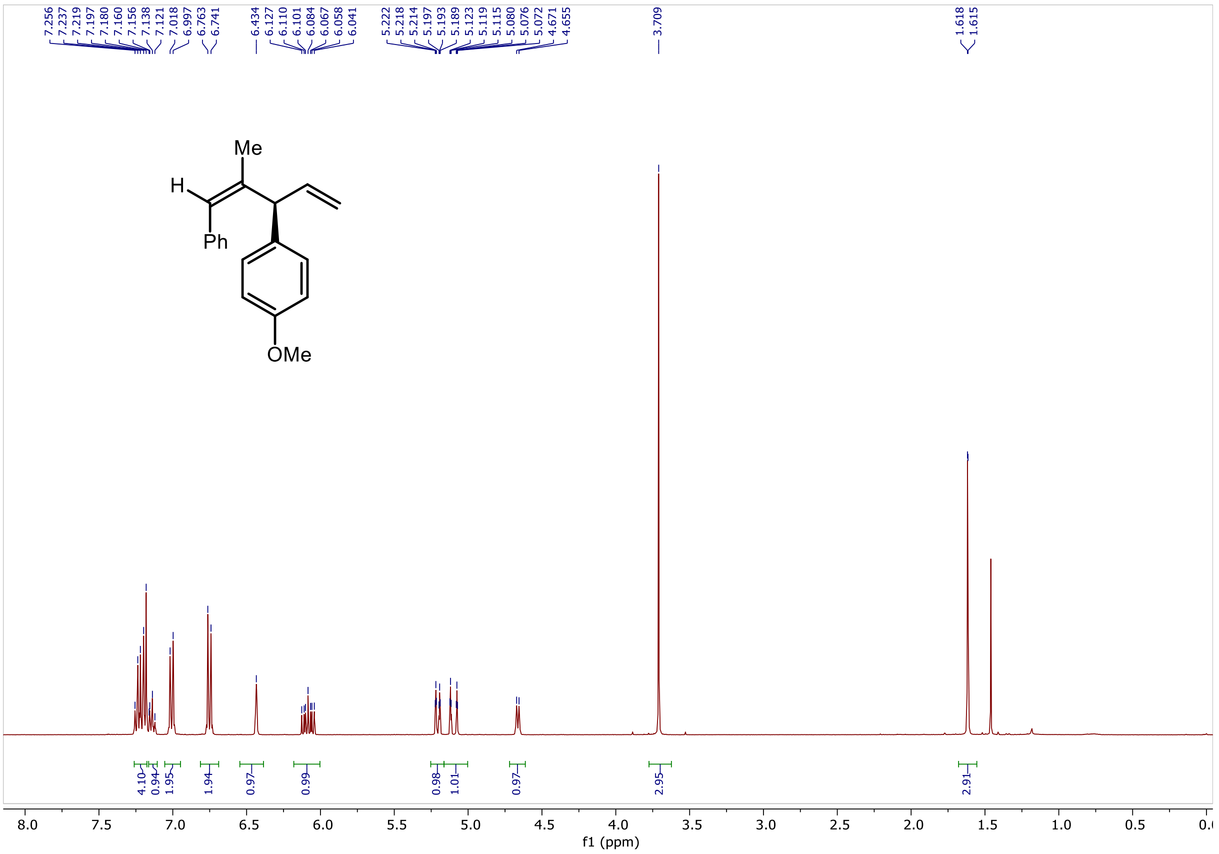

^{13}C NMR (400 MHz, $CDCl_3$) of **11**

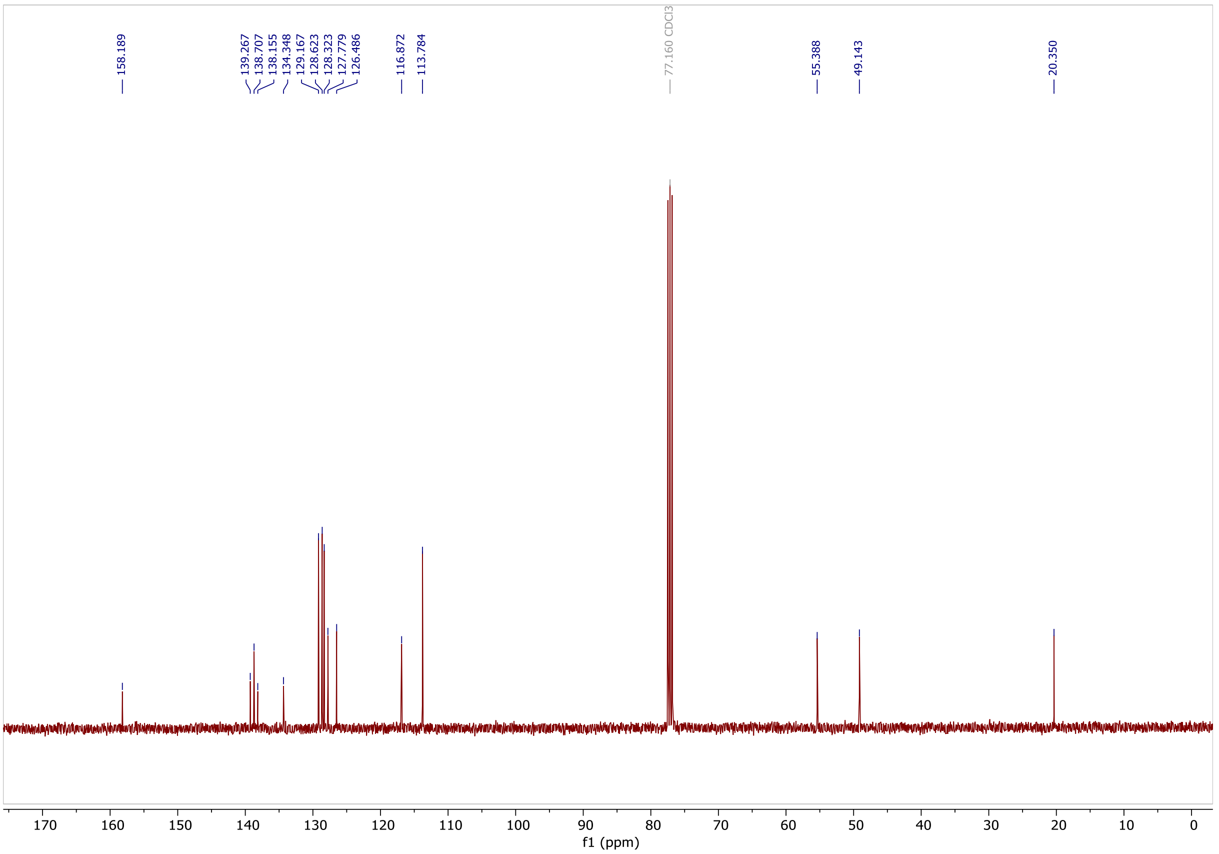

^{1}H NMR (500 MHz, $CDCl_3$) of **12**

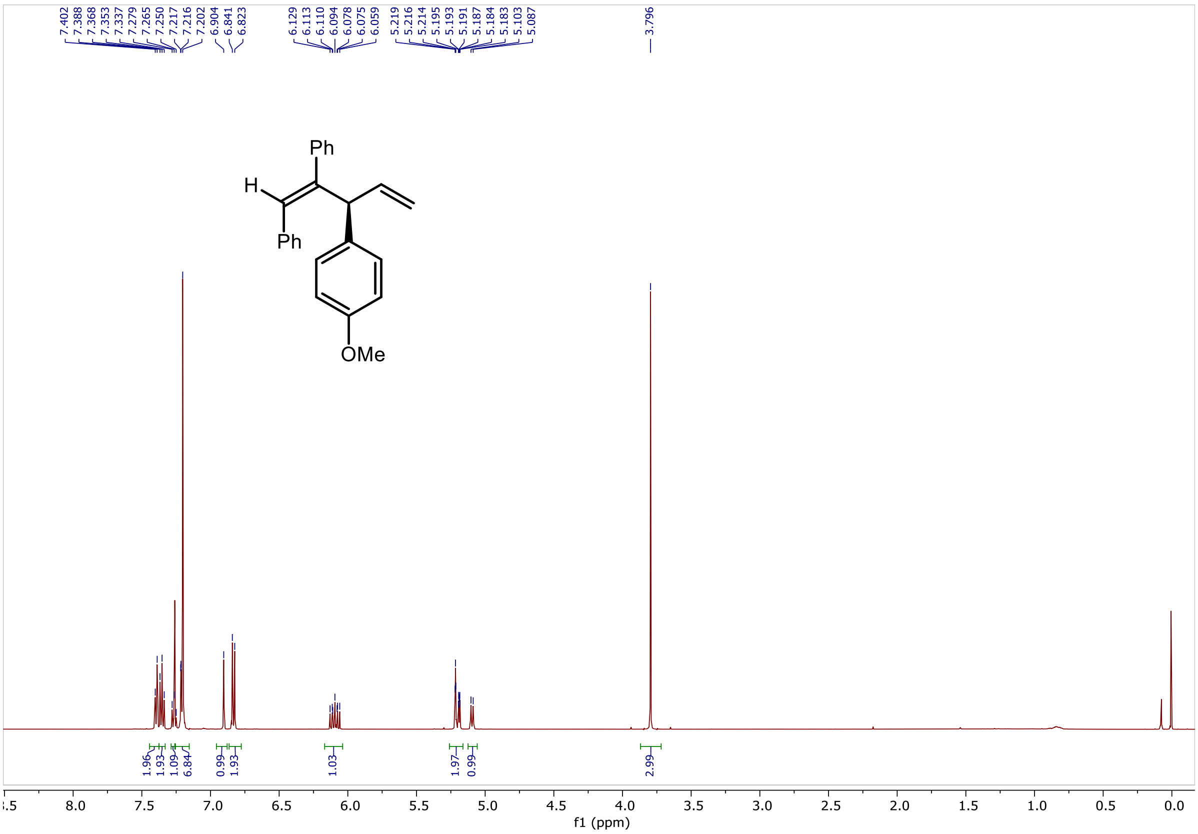

^{13}C NMR (500 MHz, $CDCl_3$) of **12**

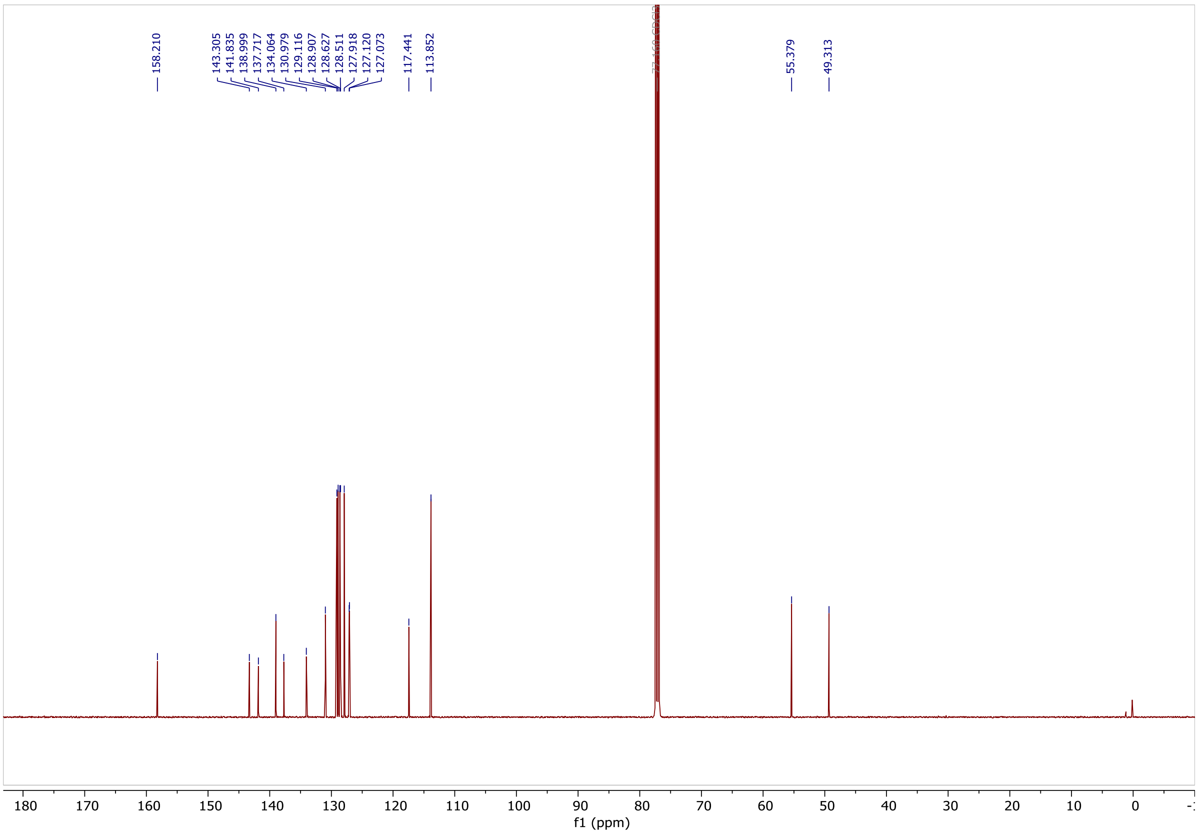

^{1}H NMR (400 MHz, $CDCl_3$) of **13**

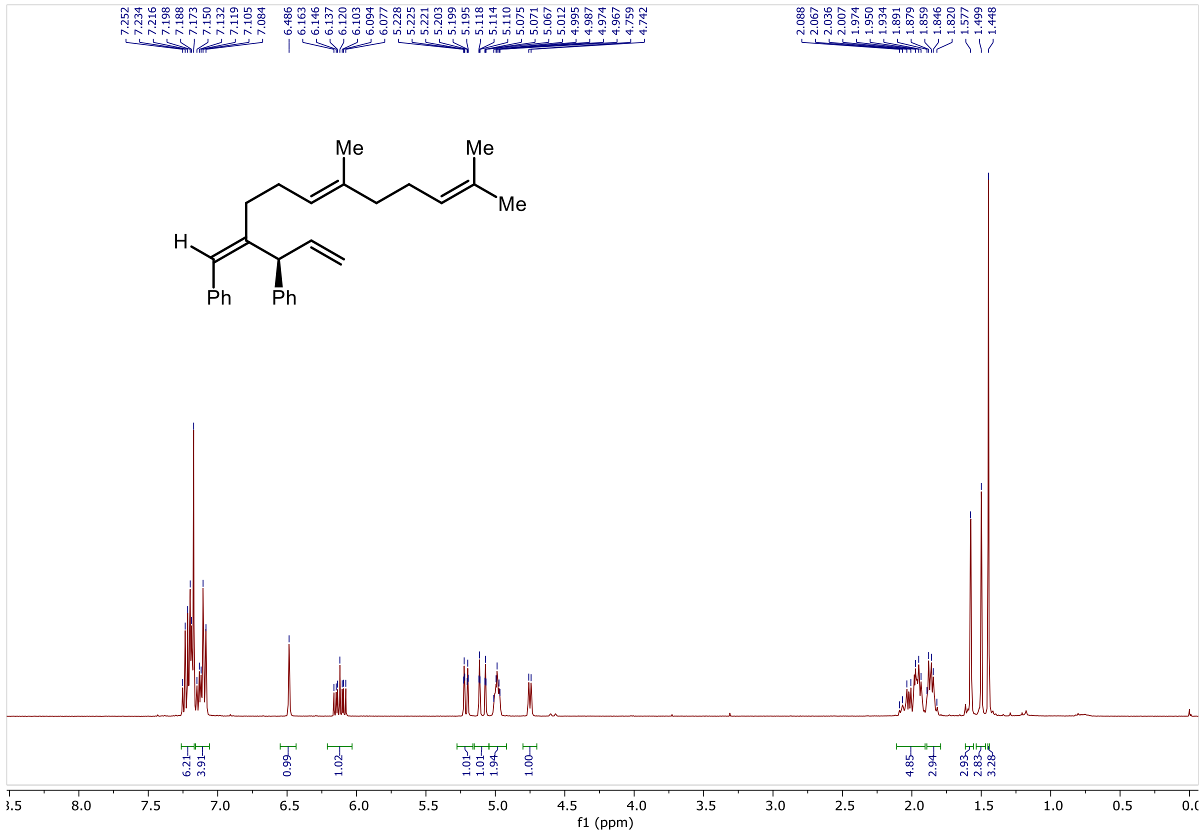

^{13}C NMR (400 MHz, $CDCl_3$) of **13**

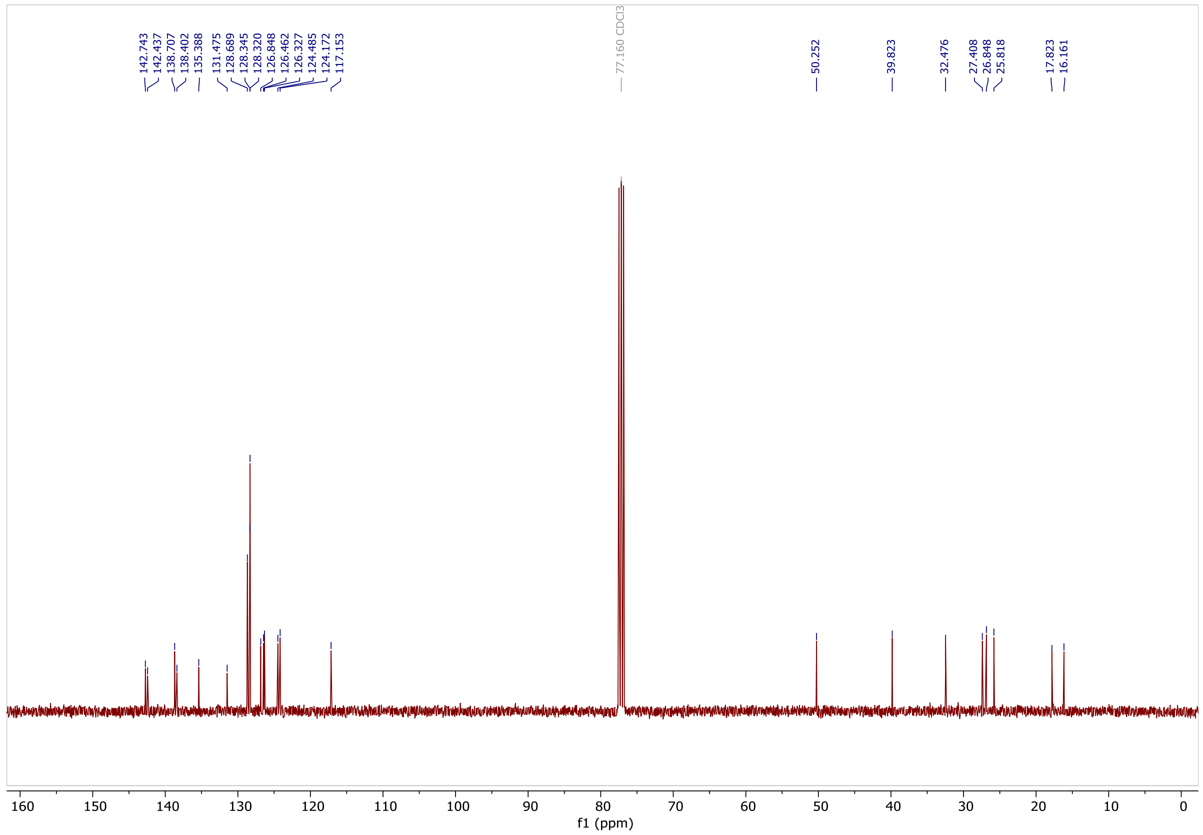

^{1}H NMR (400 MHz, $CDCl_3$) of **14**

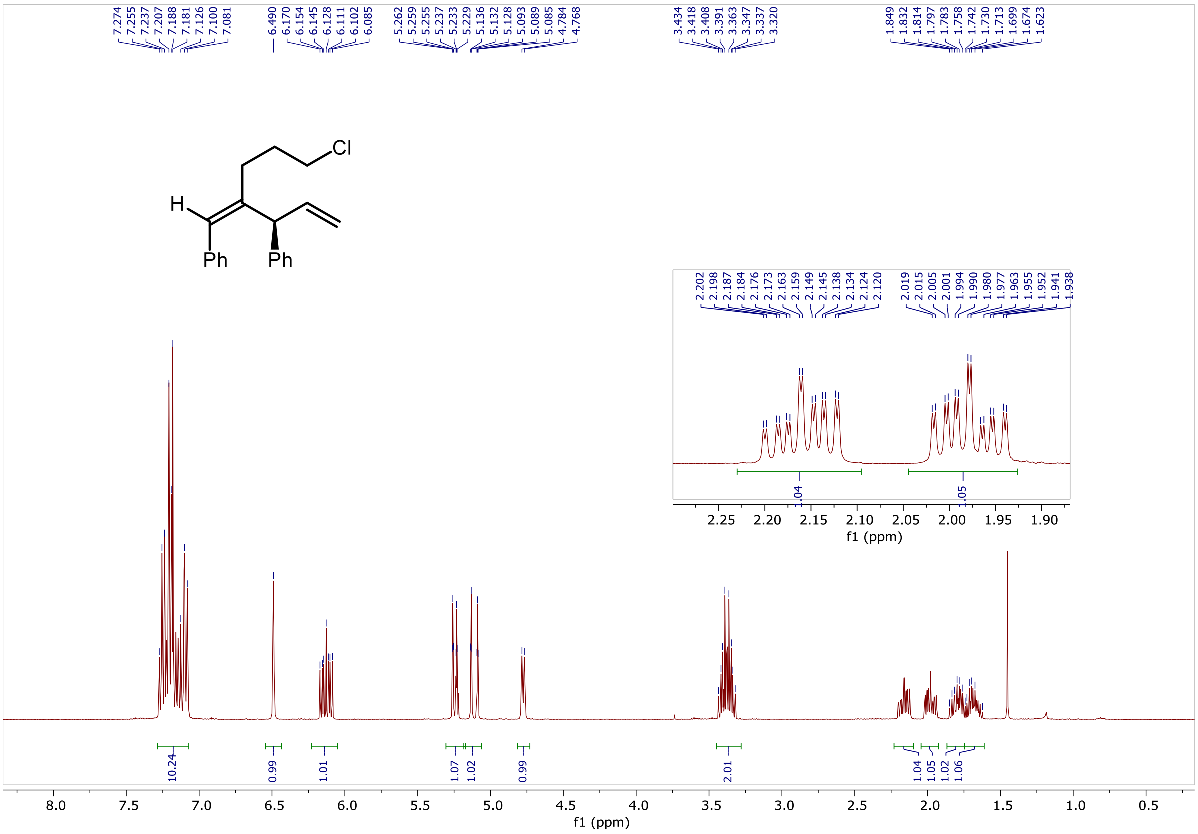

^{13}C NMR (400 MHz, $CDCl_3$) of **14**

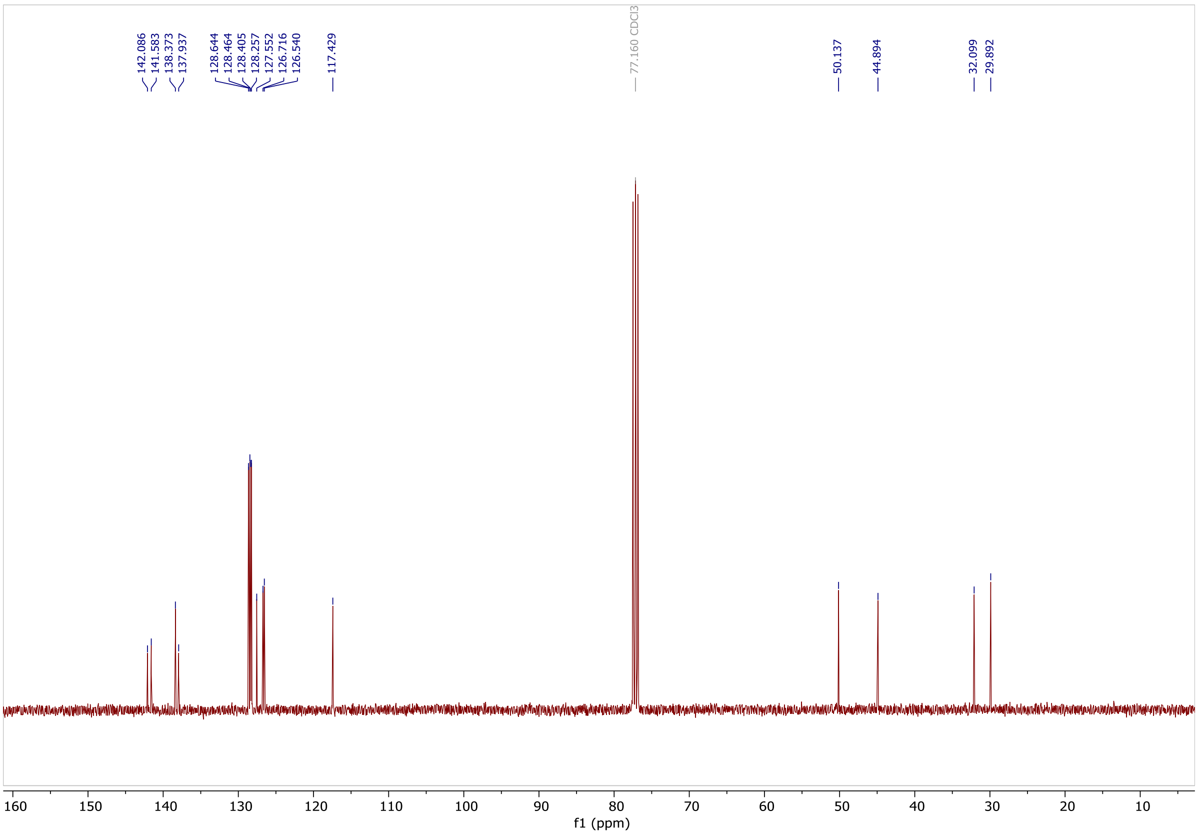

^{1}H NMR (400 MHz, $CDCl_3$) of **15**

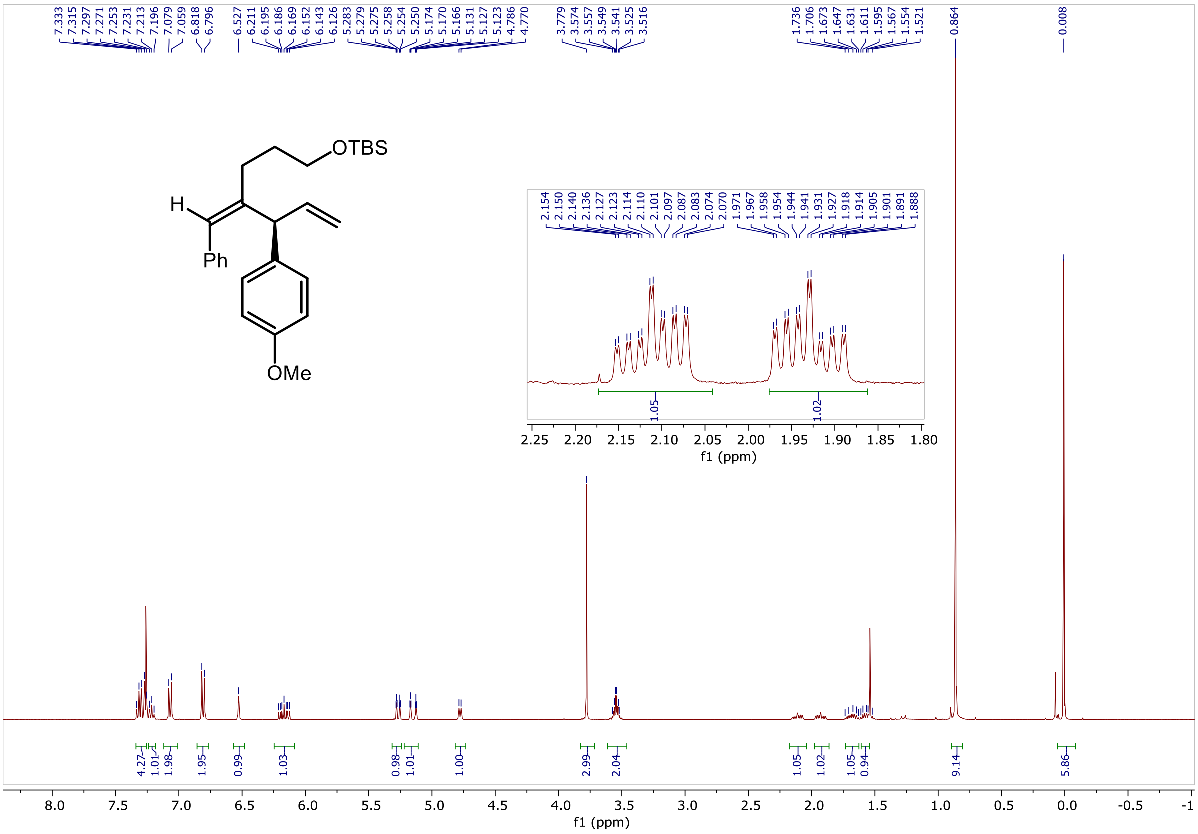

^{13}C NMR (400 MHz, $CDCl_3$) of **15**

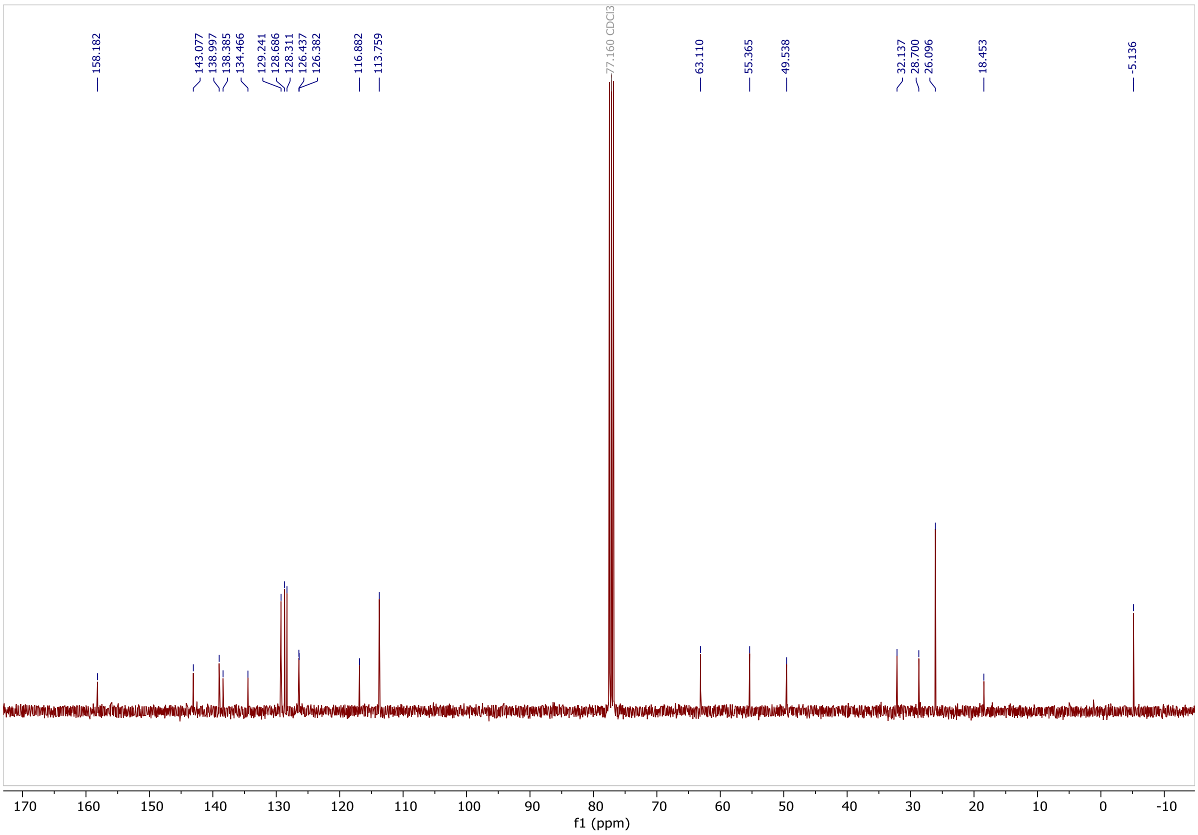

^{1}H NMR (400 MHz, $CDCl_3$) of **16**

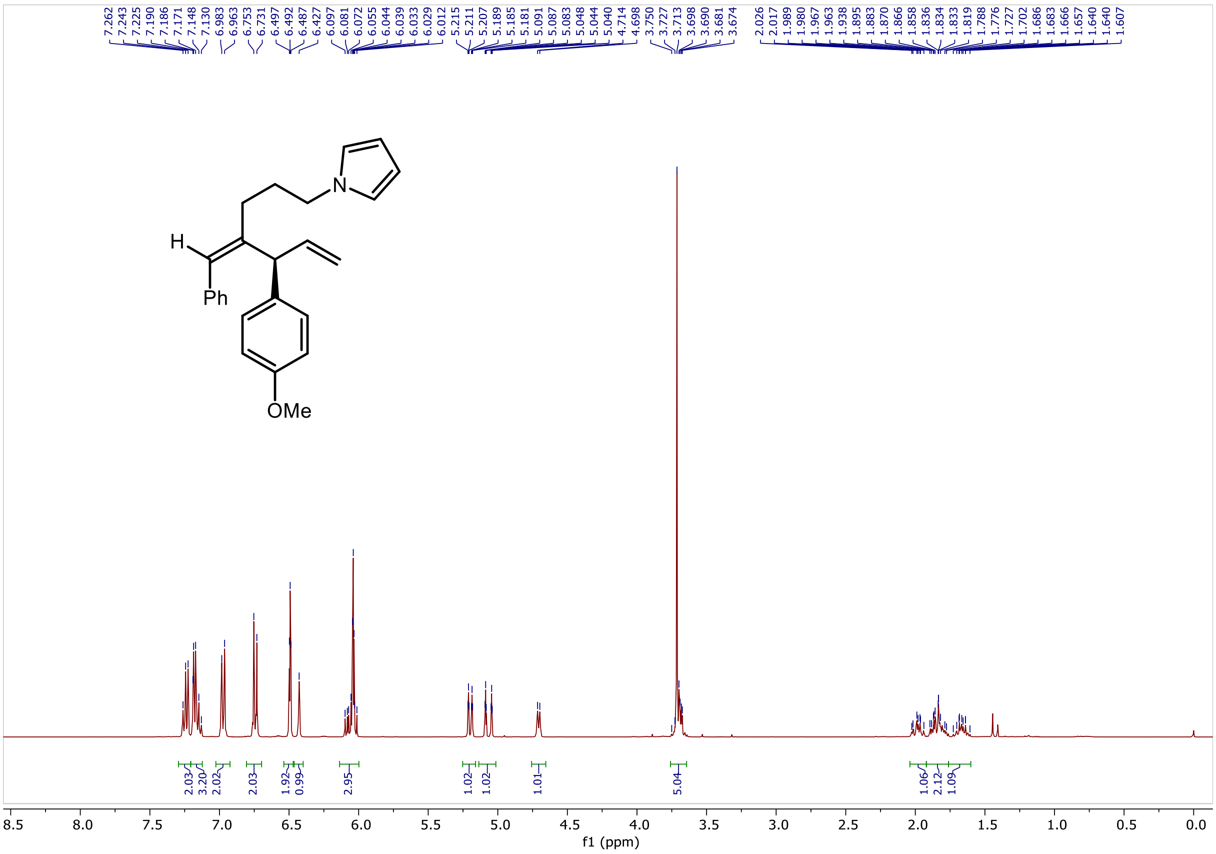

^{13}C NMR (400 MHz, $CDCl_3$) of **16**

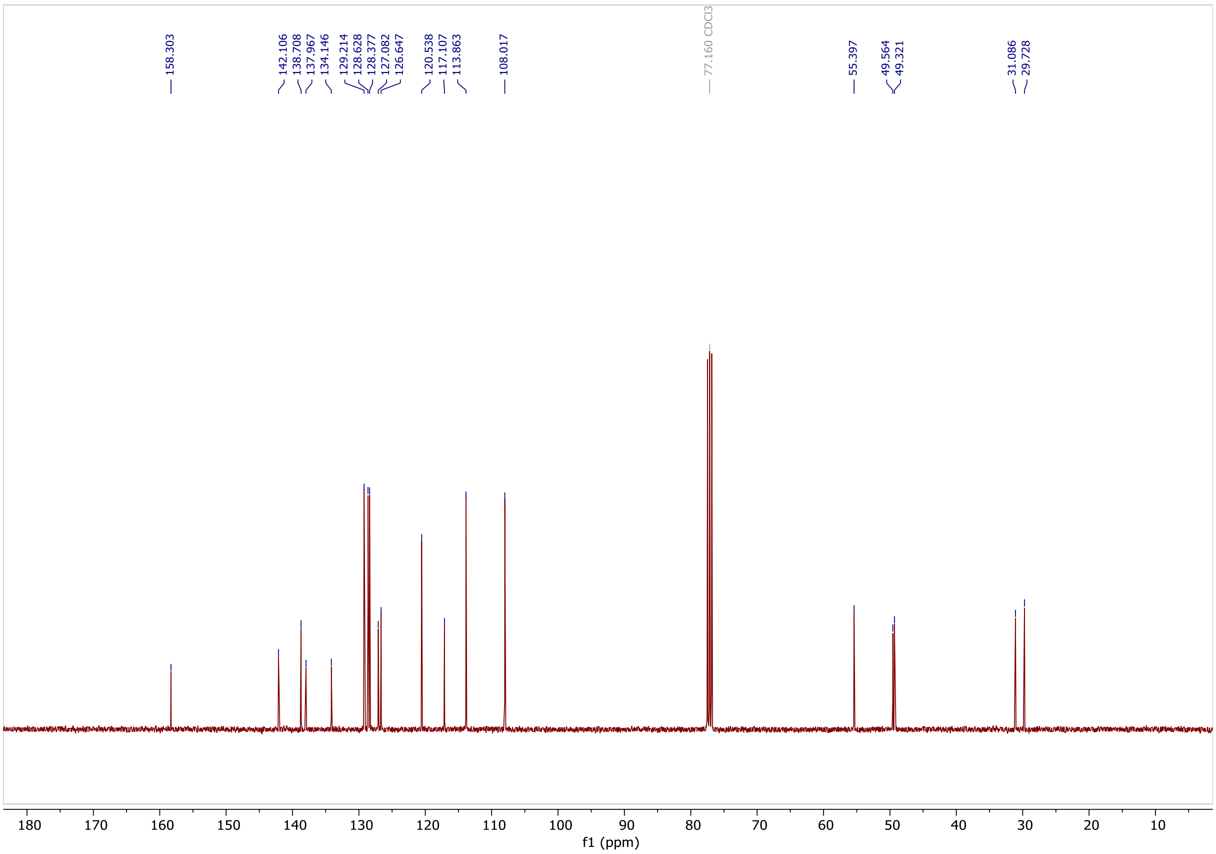

^{1}H NMR (500 MHz, $CDCl_3$) of **17**

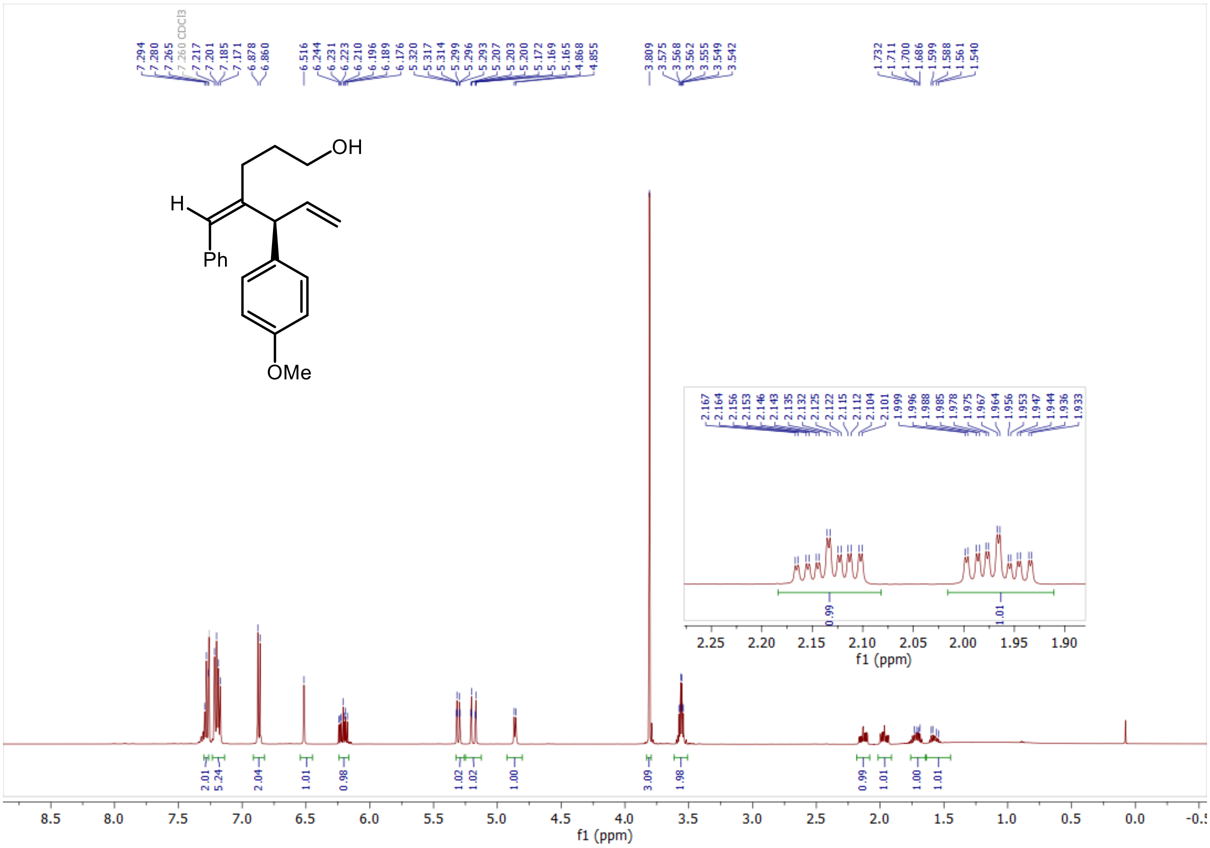

^{13}C NMR (126 MHz, $CDCl_3$) of **17**

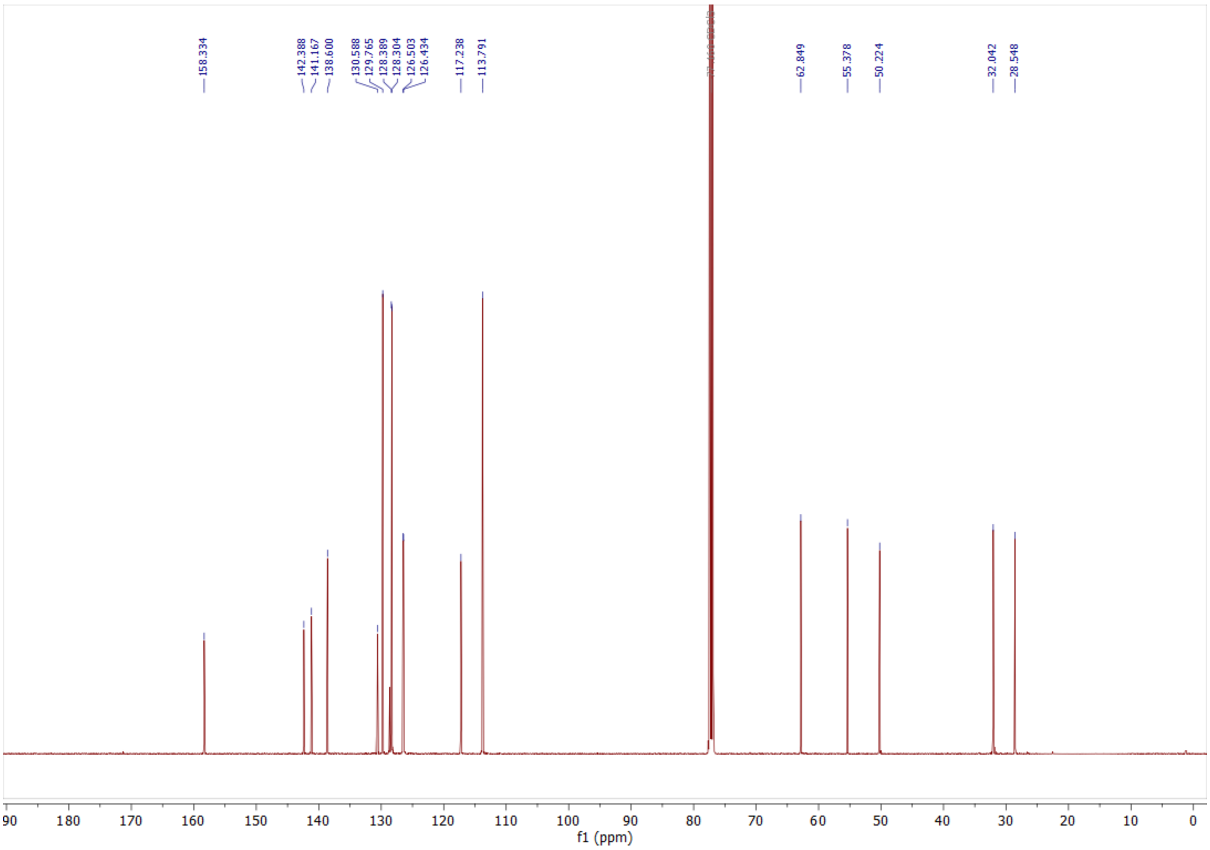

^{1}H NMR (500 MHz, $CDCl_3$) of **18**

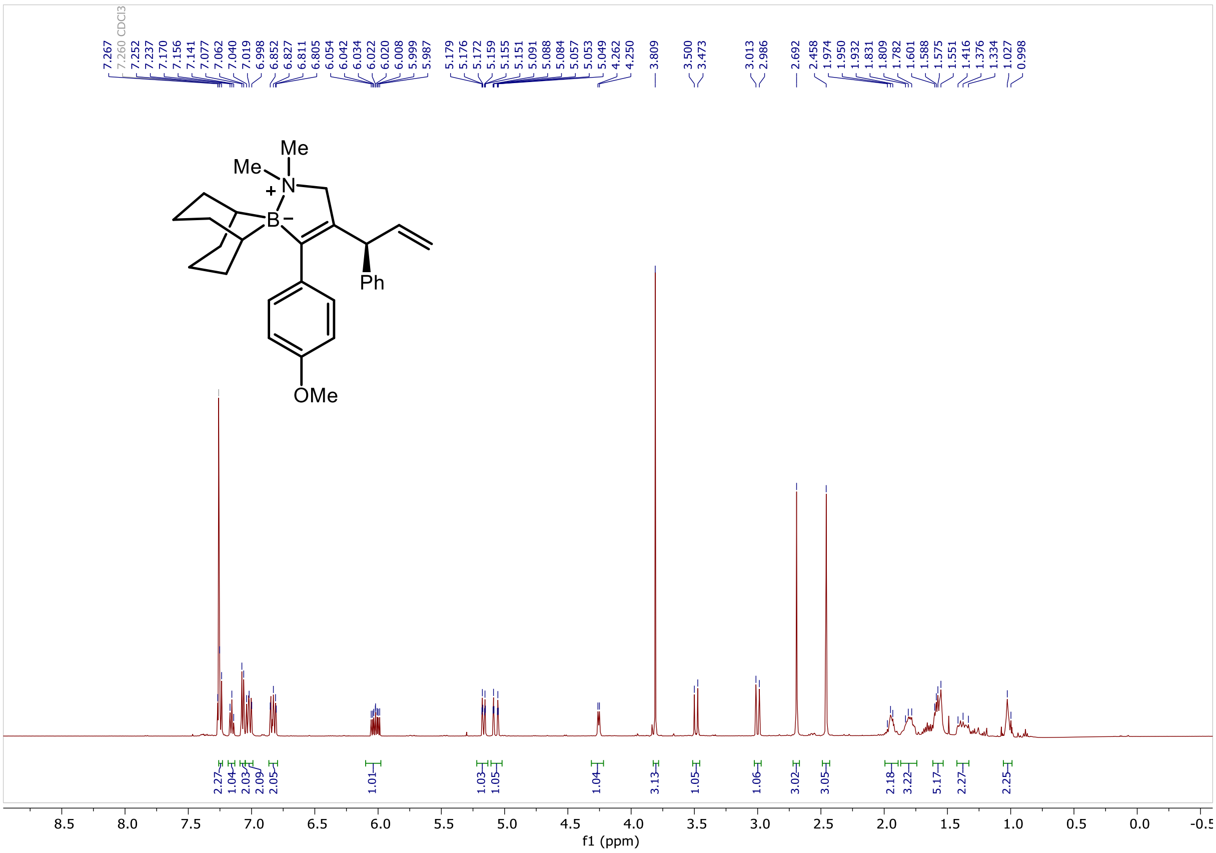

^{13}C NMR (126 MHz, $CDCl_3$) of **18**

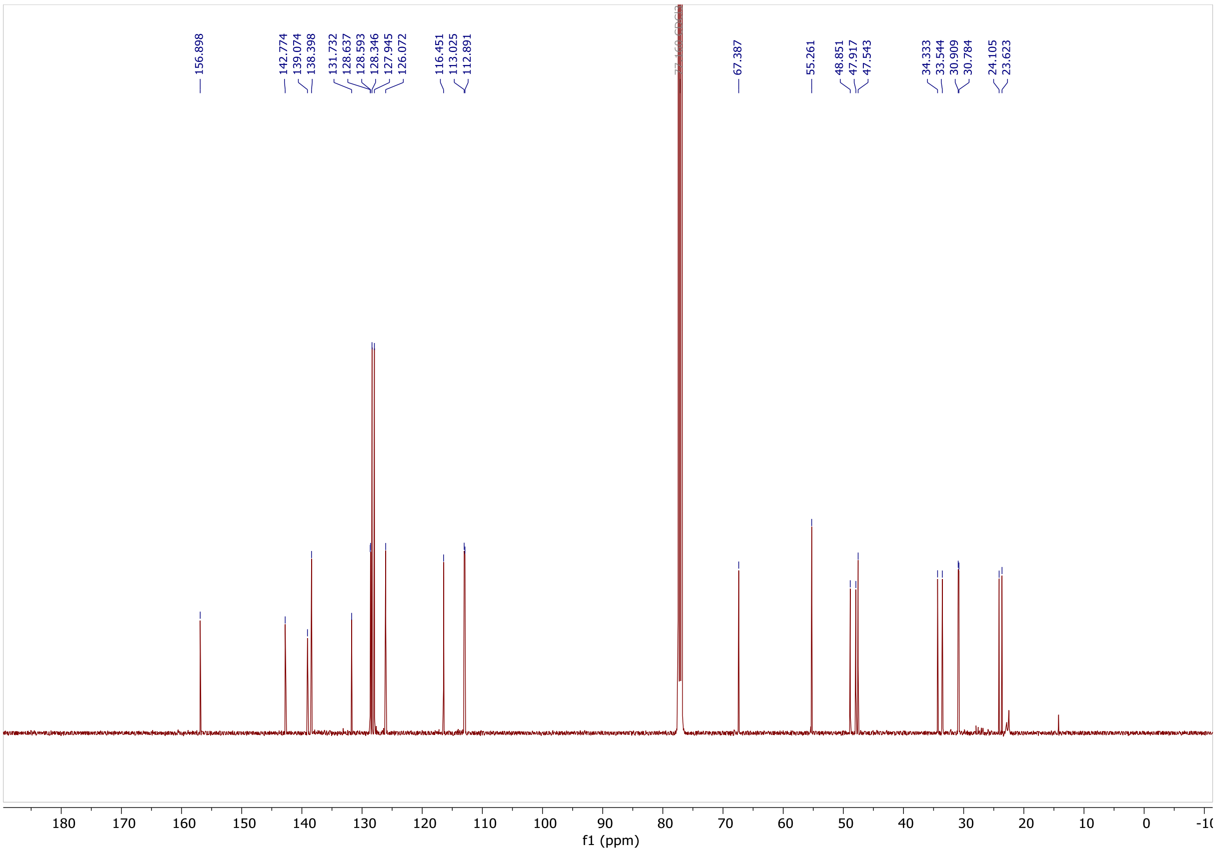

^{1}H NMR (400 MHz, $CDCl_3$) of **19**

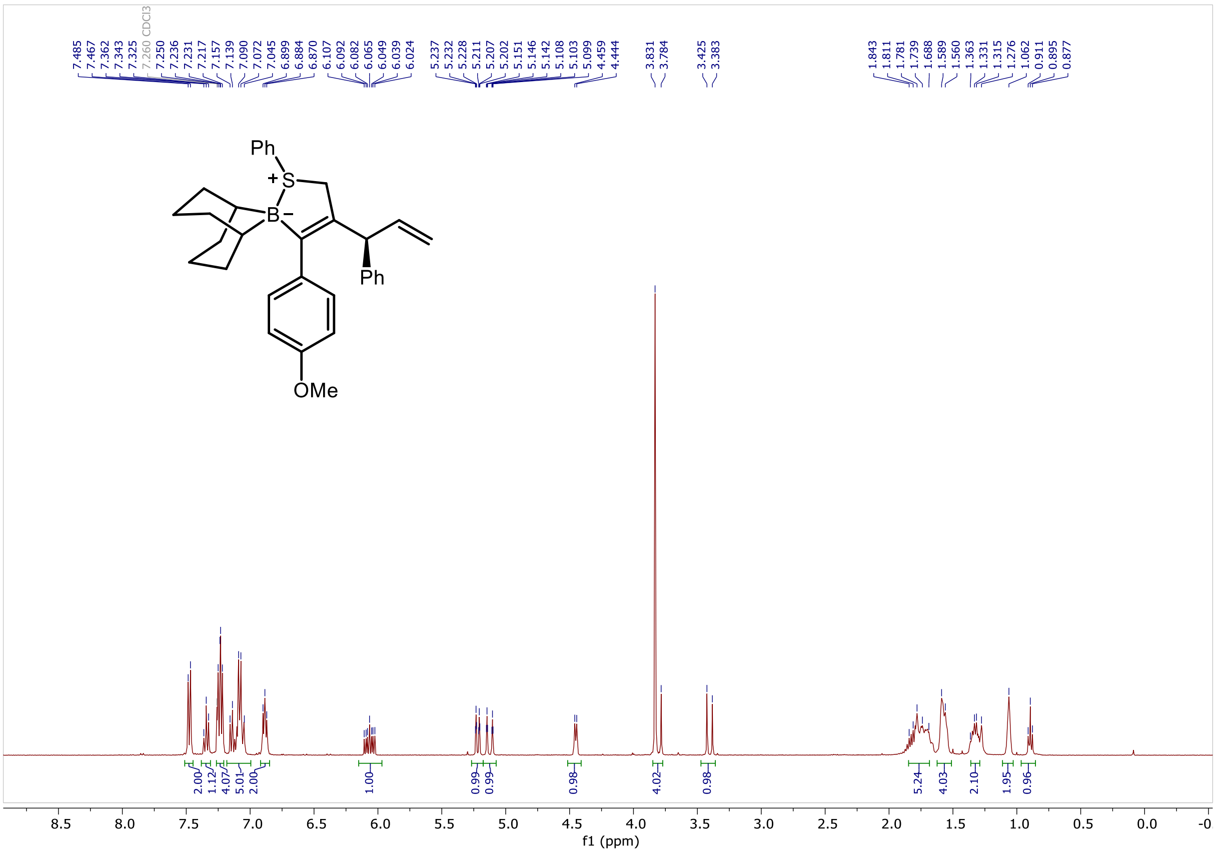

^{13}C NMR (400 MHz, $CDCl_3$) of **19**

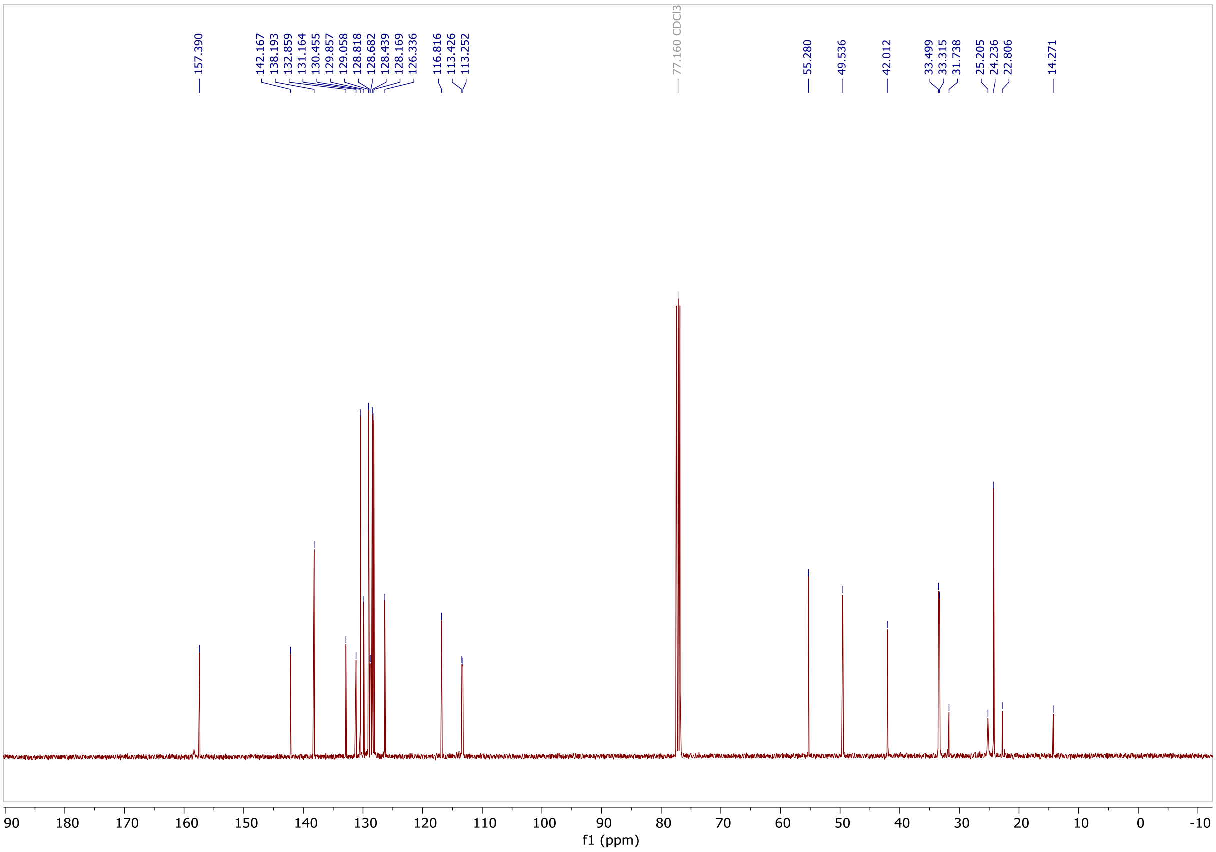

^{1}H NMR (400 MHz, $CDCl_3$) of **20**

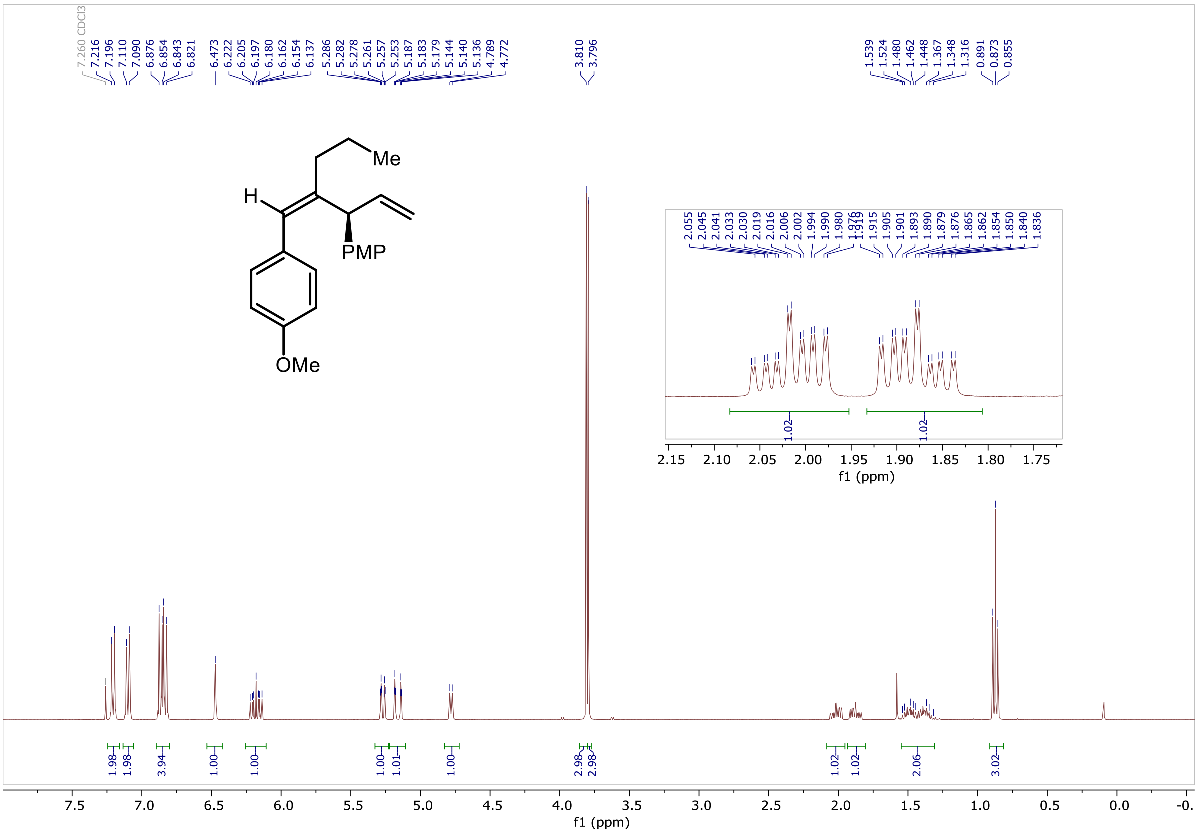

^{13}C NMR (400 MHz, $CDCl_3$) of **20**

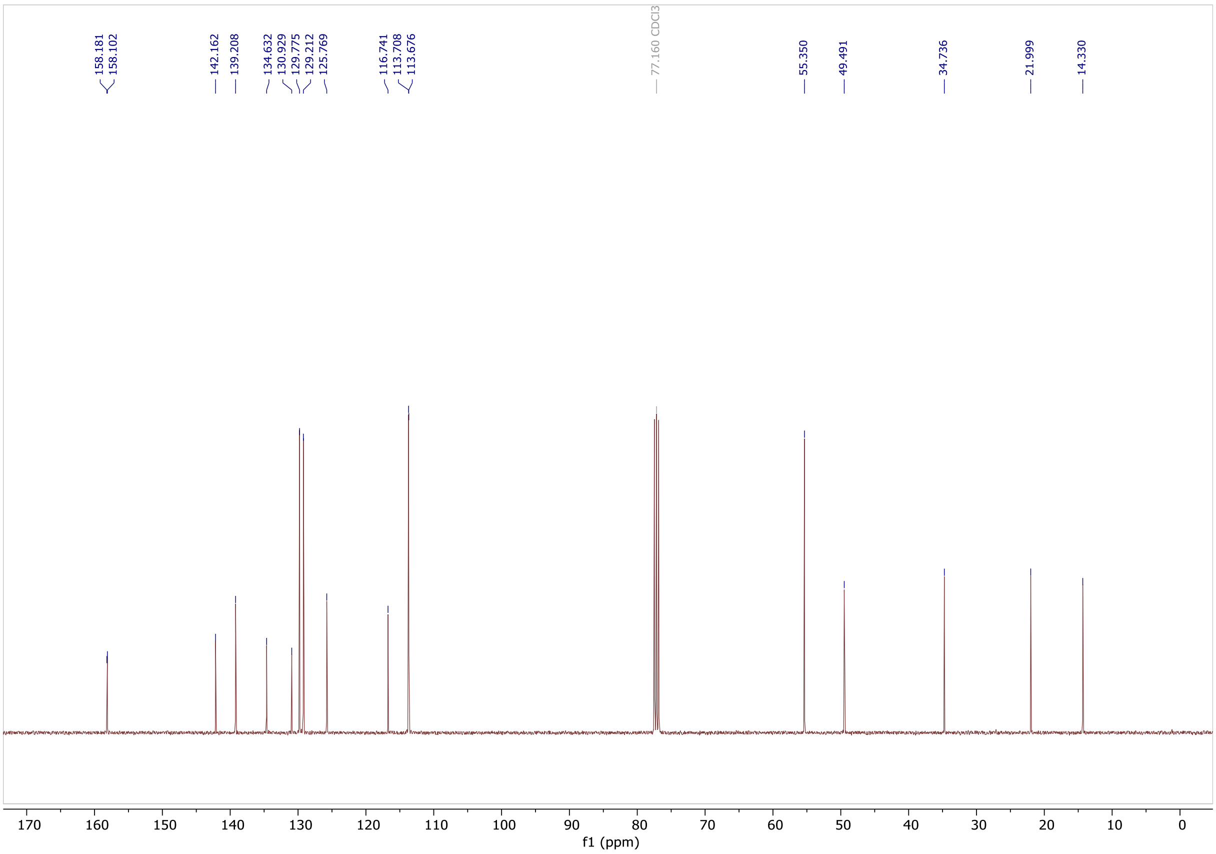

^{1}H NMR (400 MHz, $CDCl_3$) of **21**

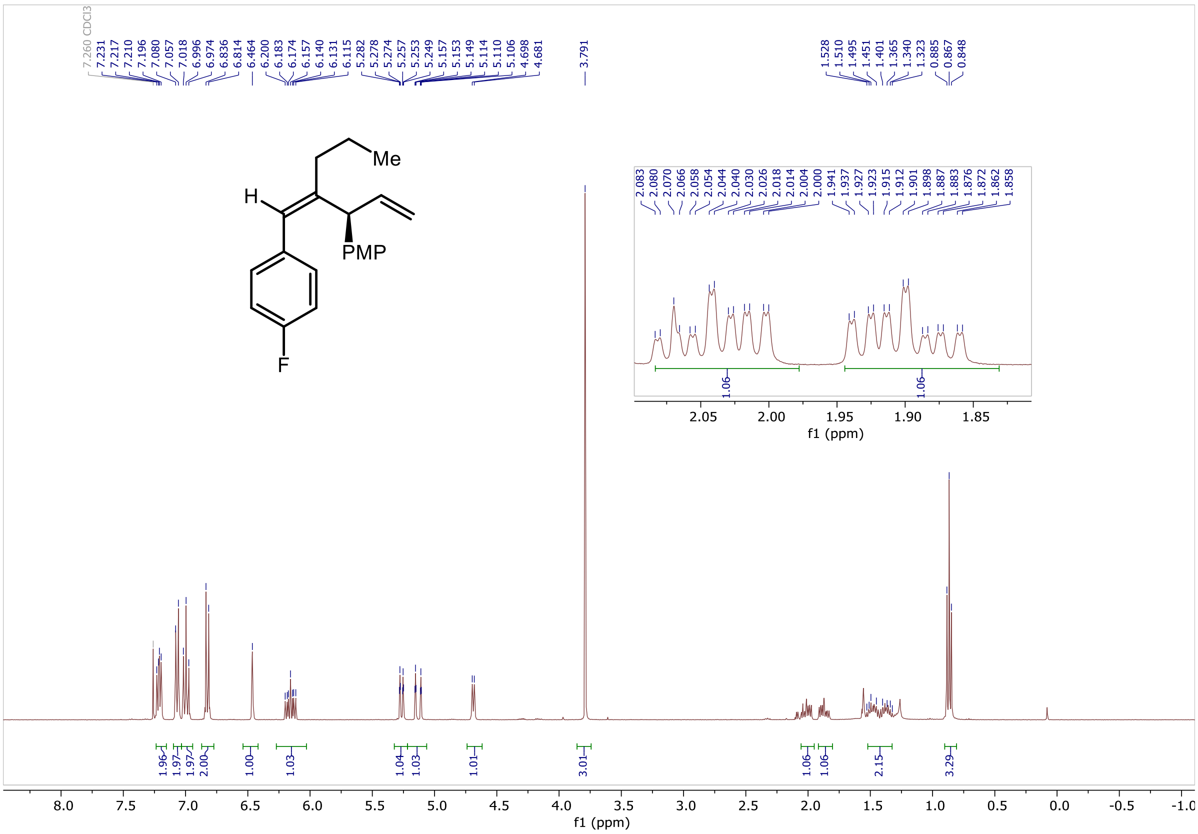

^{13}C NMR (400 MHz, $CDCl_3$) of **21**

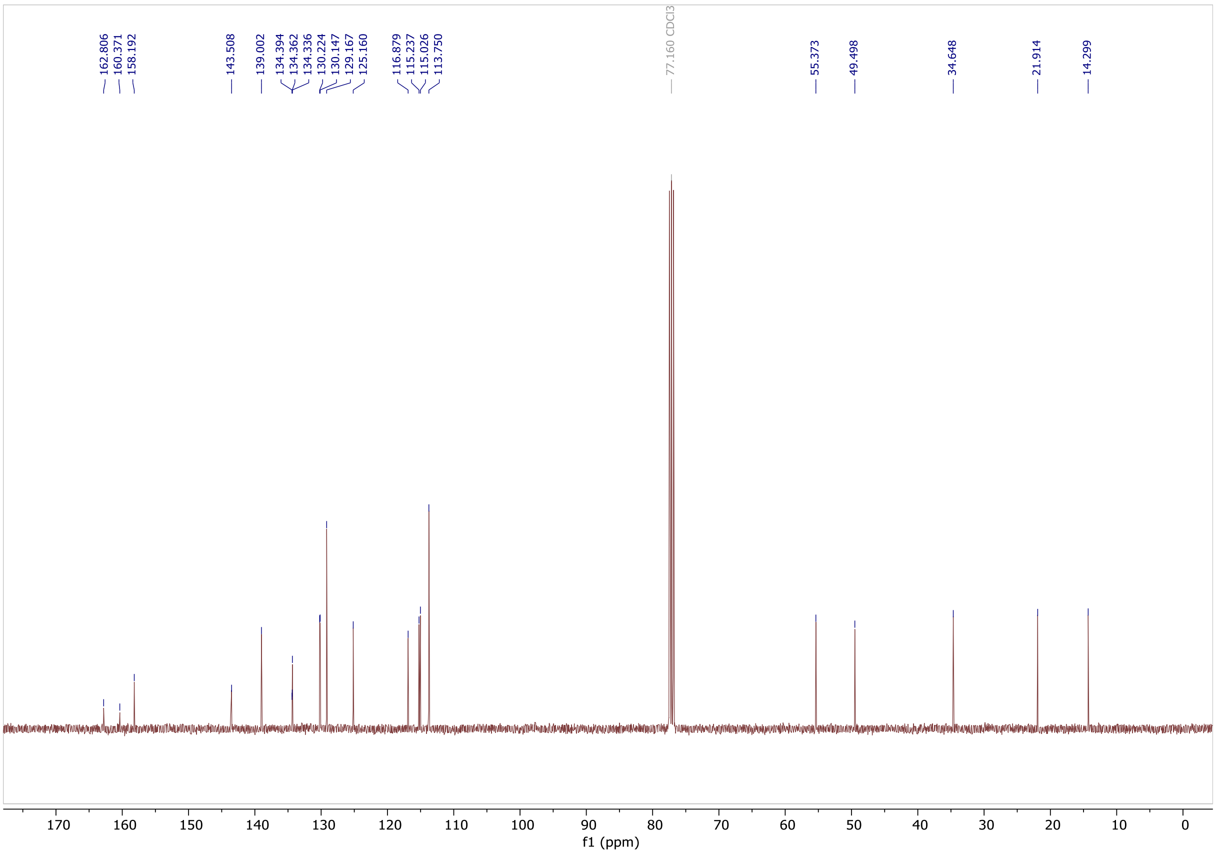

^{19}F NMR (377 MHz, $CDCl_3$) of **21**

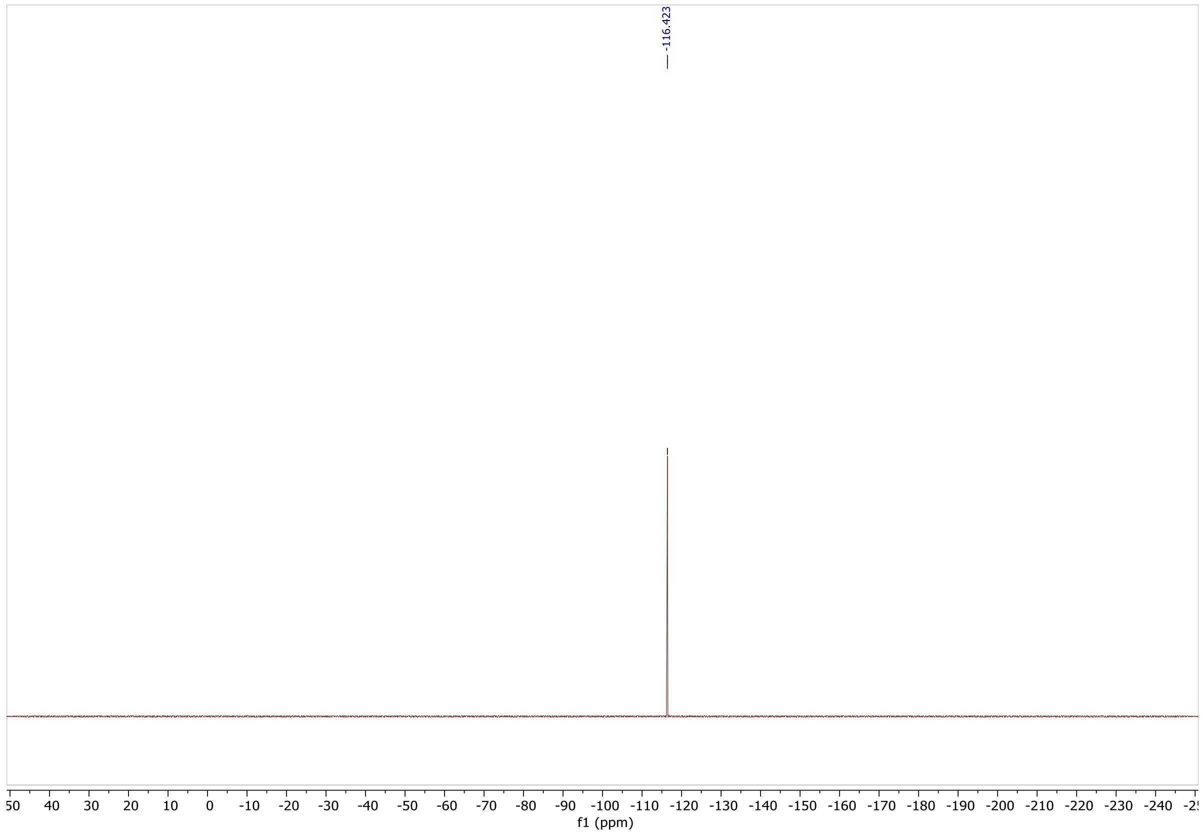

^{1}H NMR (400 MHz, $CDCl_3$) of **22**

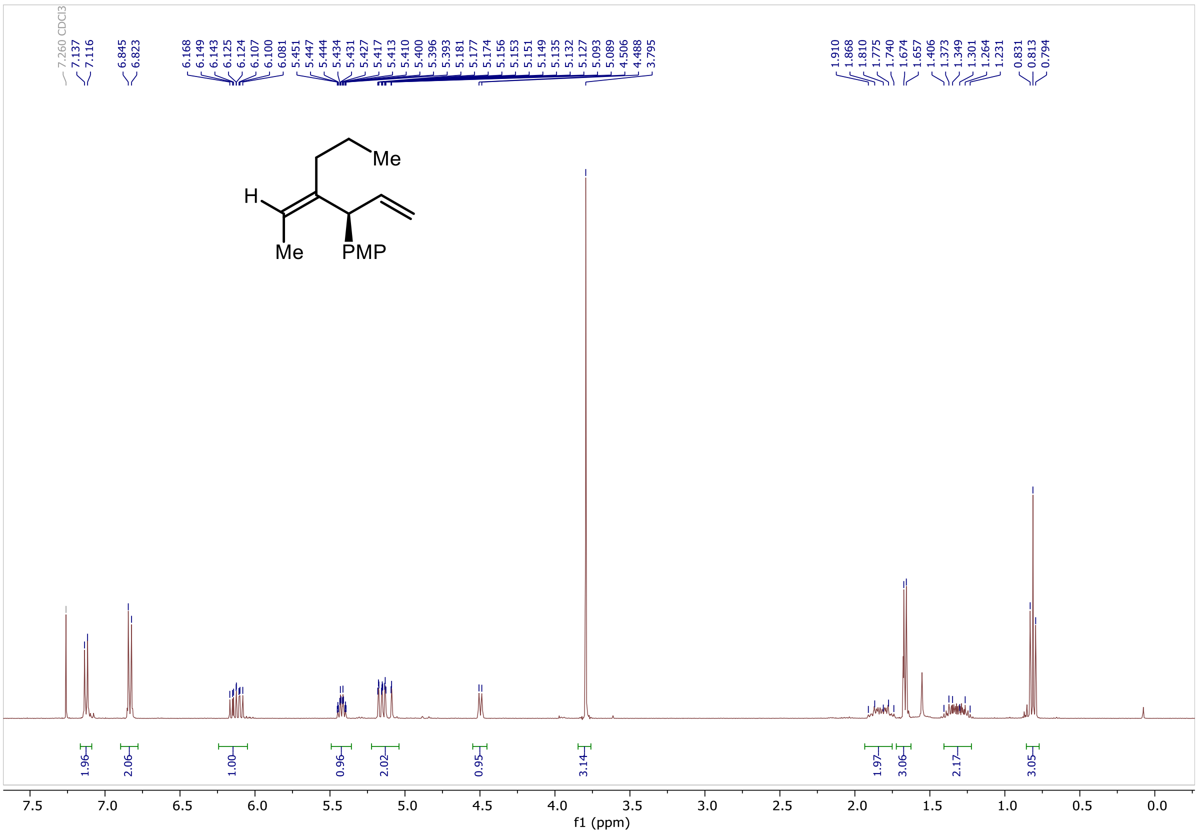

^{13}C NMR (400 MHz, $CDCl_3$) of **22**

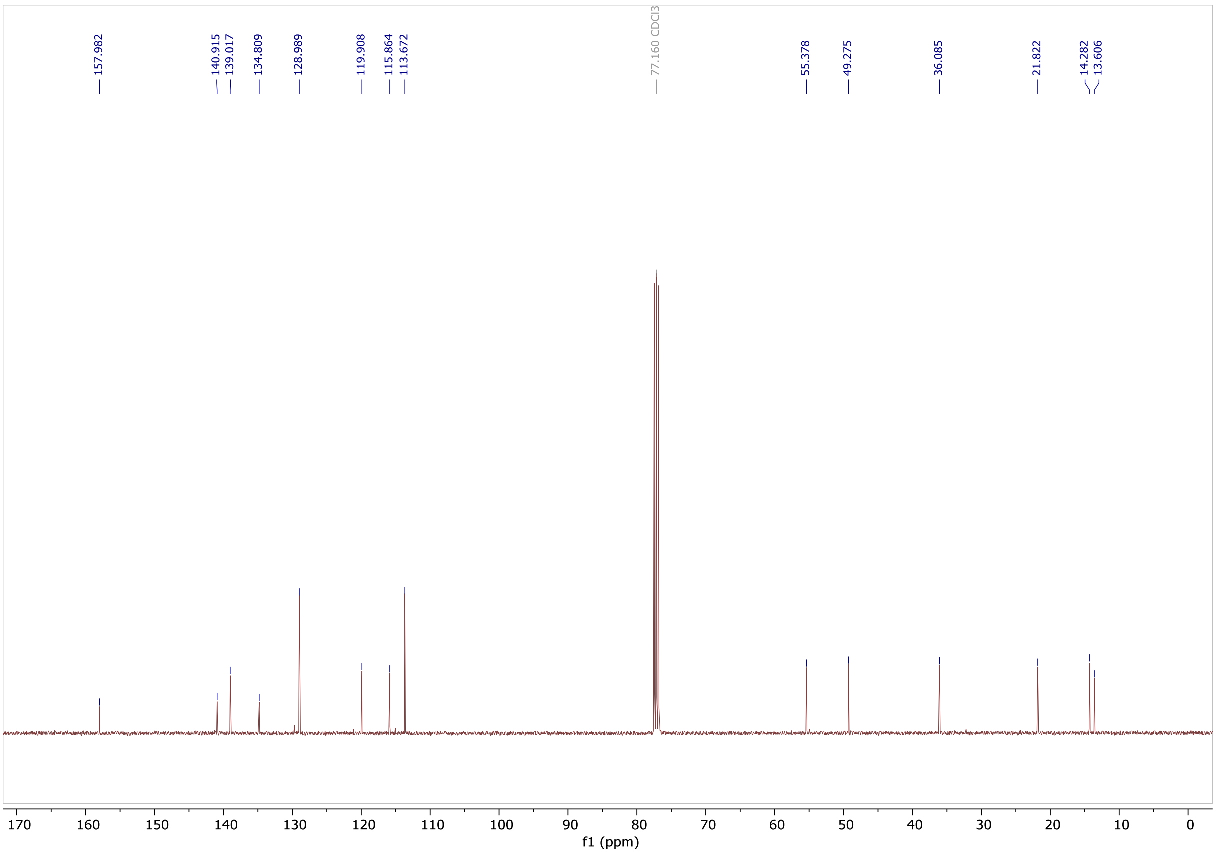

^{1}H NMR (400 MHz, $CDCl_3$) of **23**

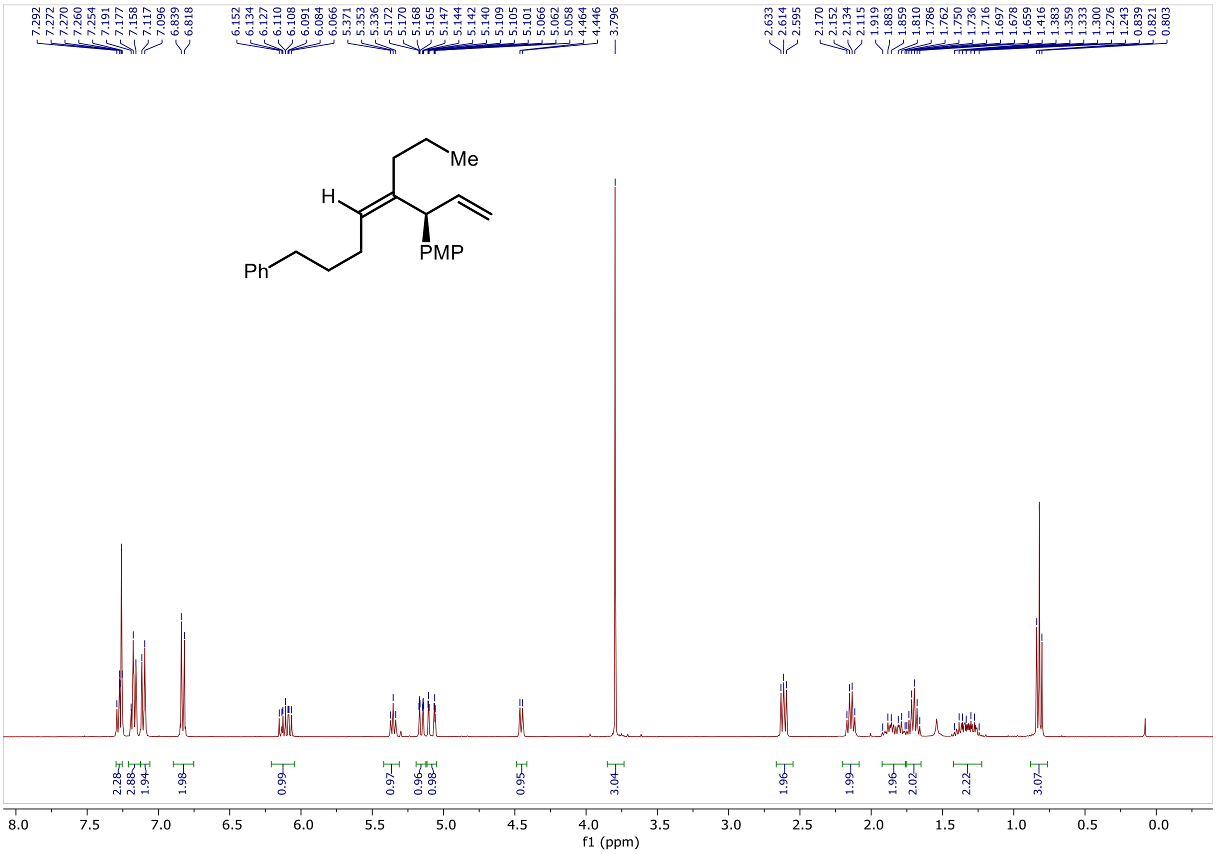

^{13}C NMR (400 MHz, $CDCl_3$) of **23**

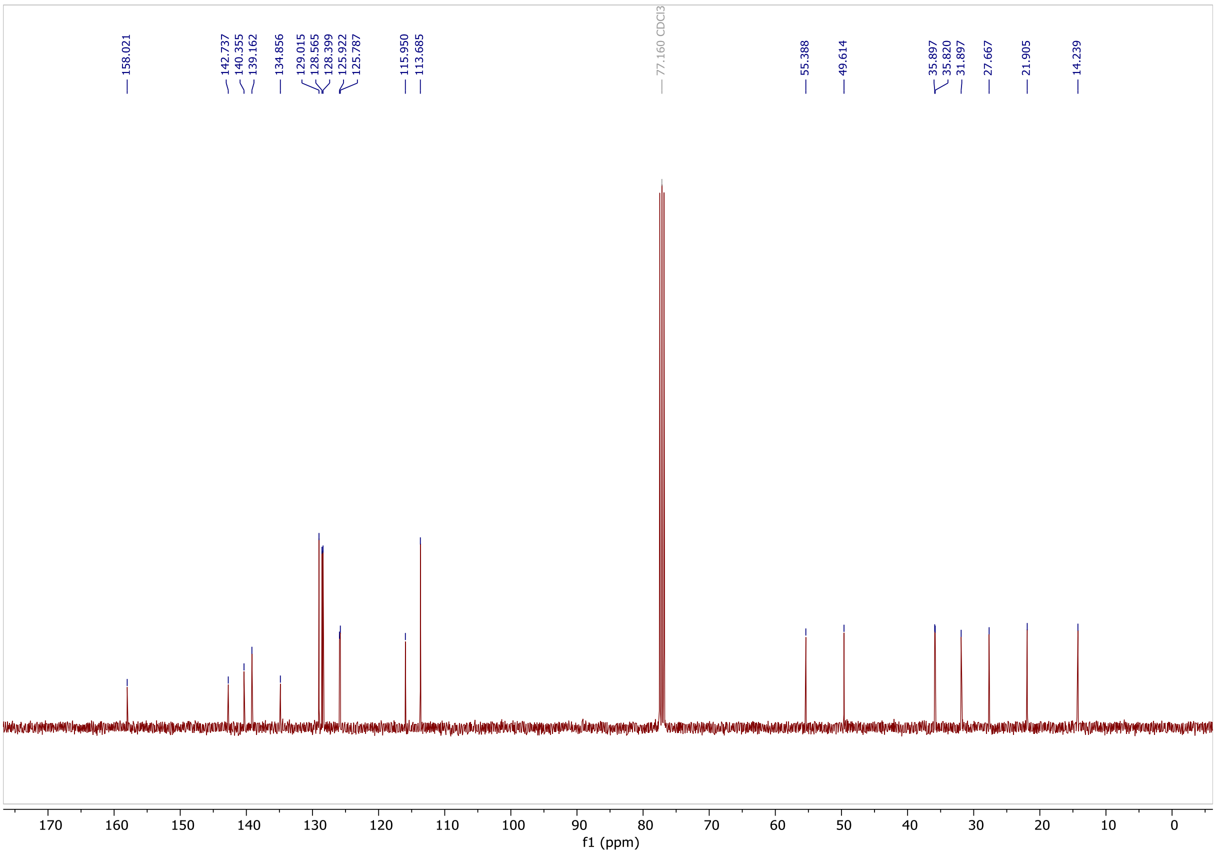

^{1}H NMR (400 MHz, $CDCl_3$) of **24**

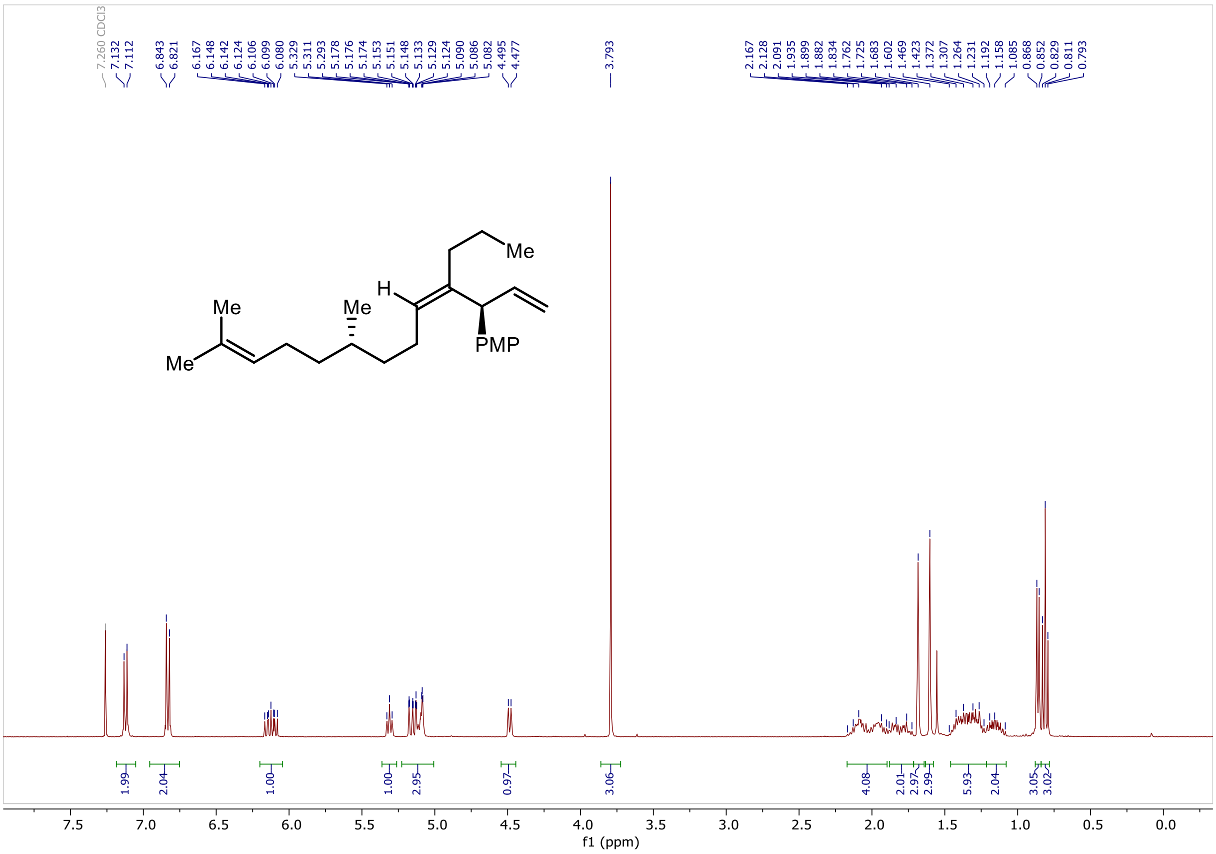

^{13}C NMR (101 MHz, $CDCl_3$) of **24**

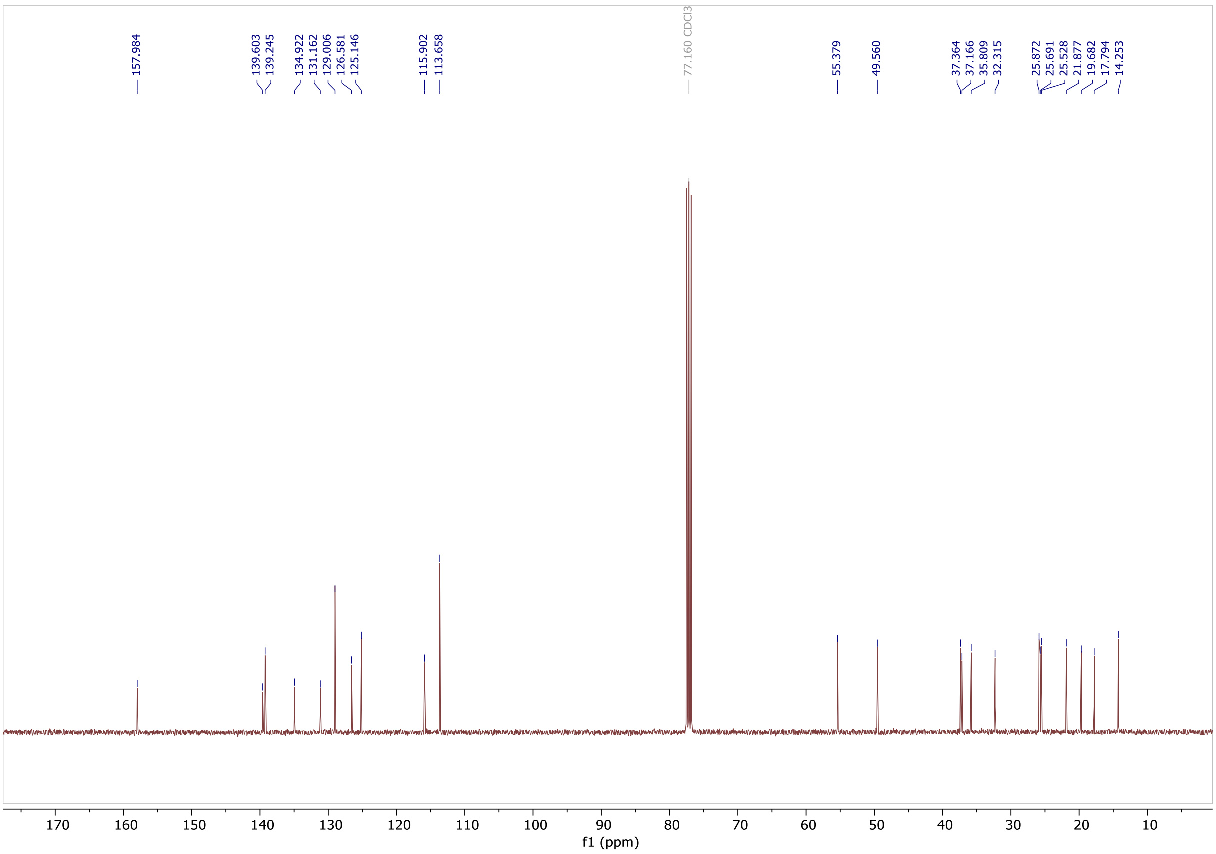

^{1}H NMR (500 MHz, CDCl$_3$) of **25**

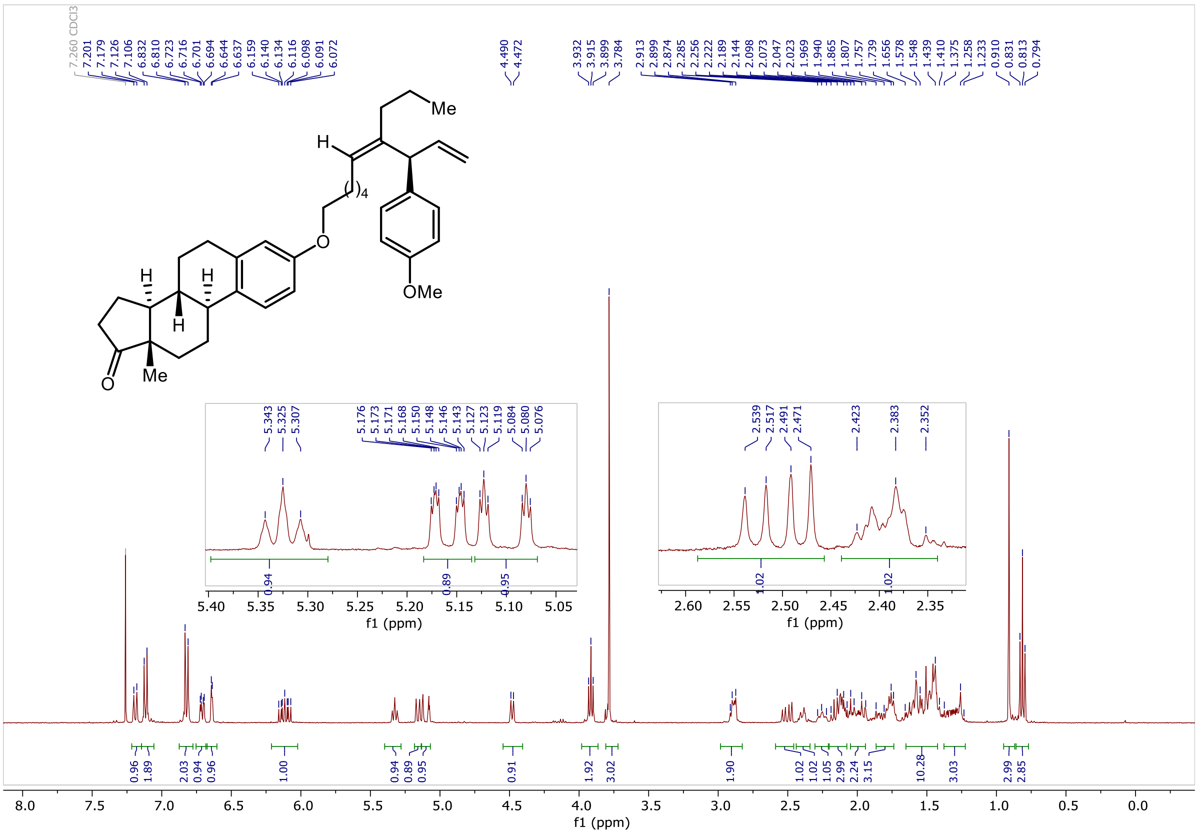

^{13}C NMR (126 MHz, CDCl$_3$) of **25**

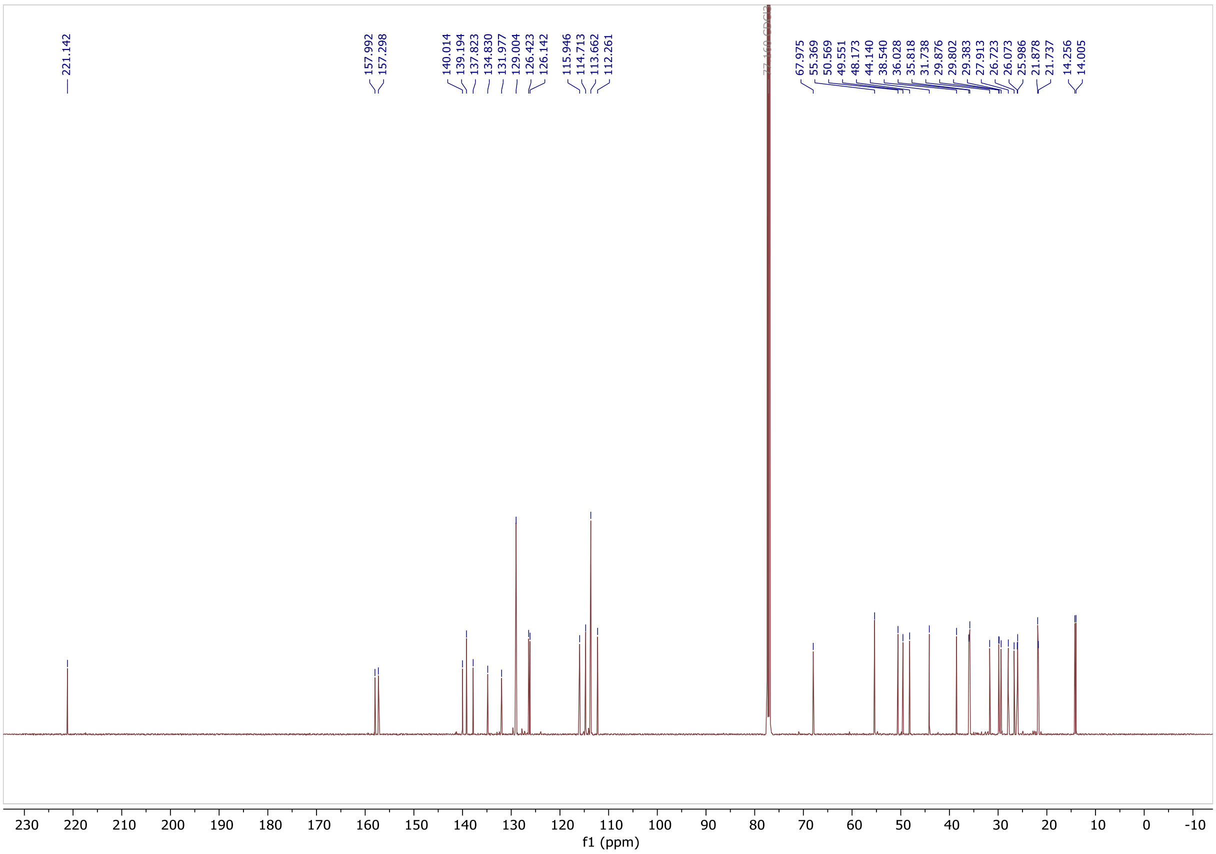

^{1}H NMR (500 MHz, $CDCl_3$) of **26**

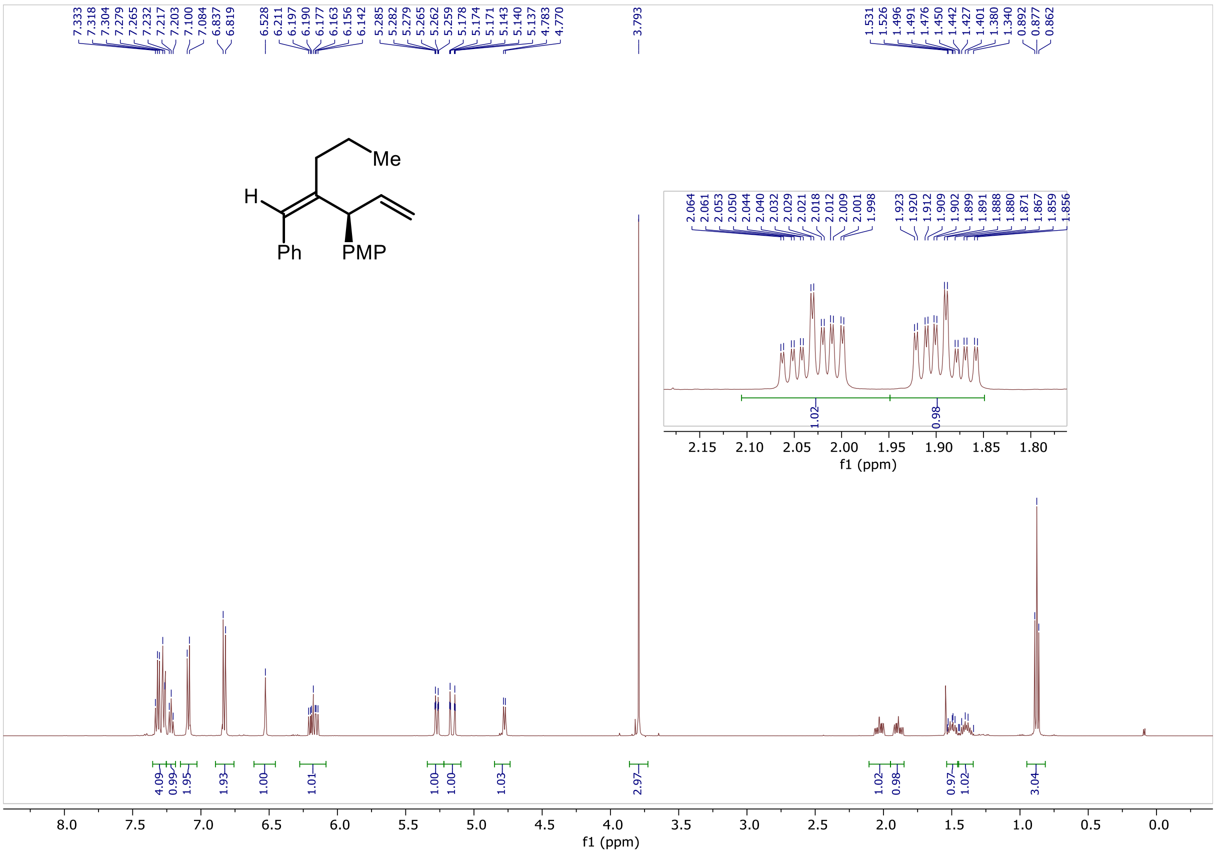

^{13}C NMR (126 MHz, $CDCl_3$) of **26**

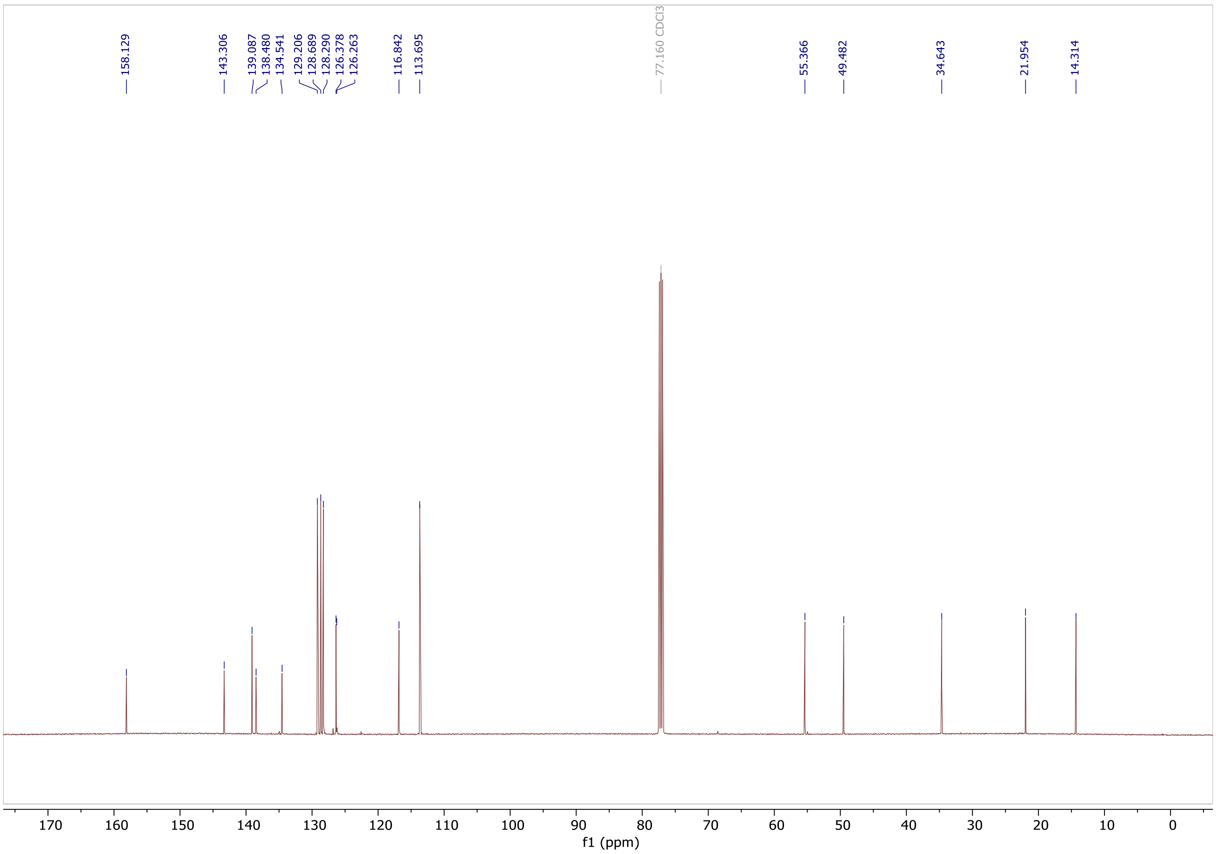

^{1}H NMR (500 MHz, $CDCl_3$) of **27**

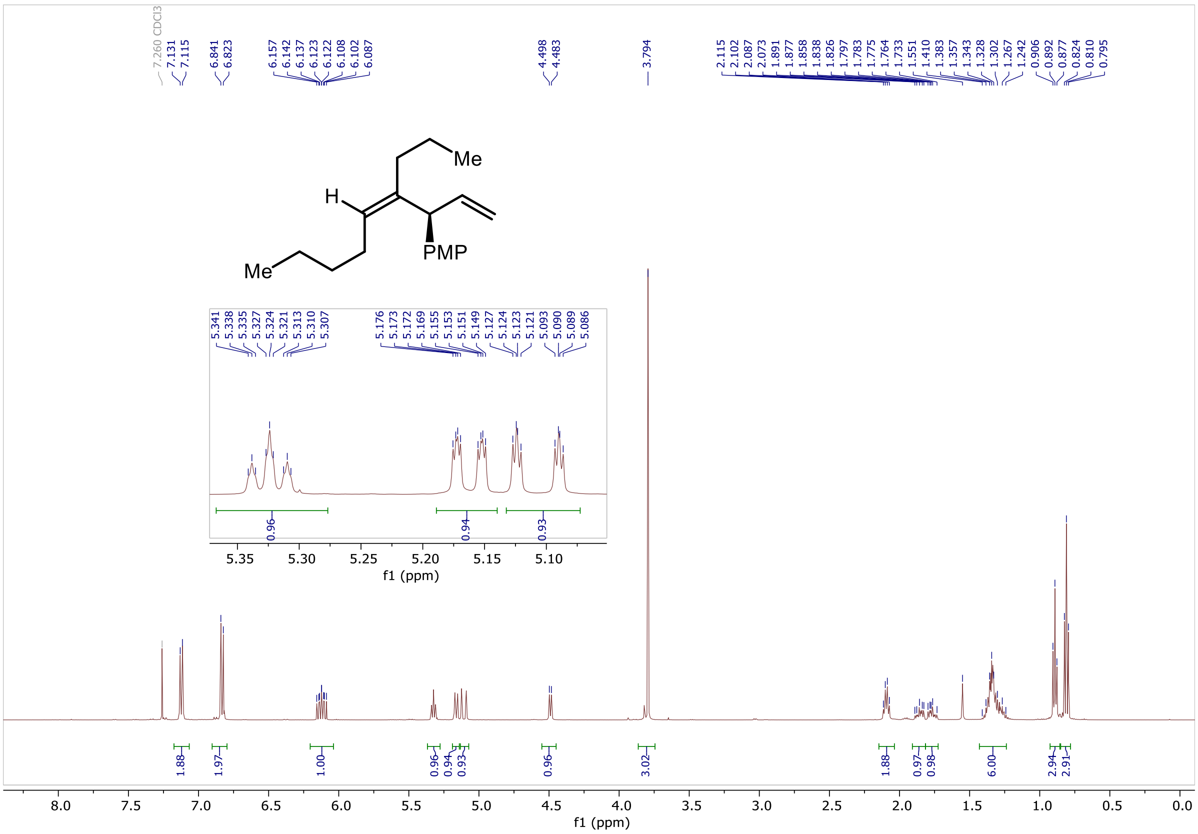

^{13}C NMR (101 MHz, $CDCl_3$) of **27**

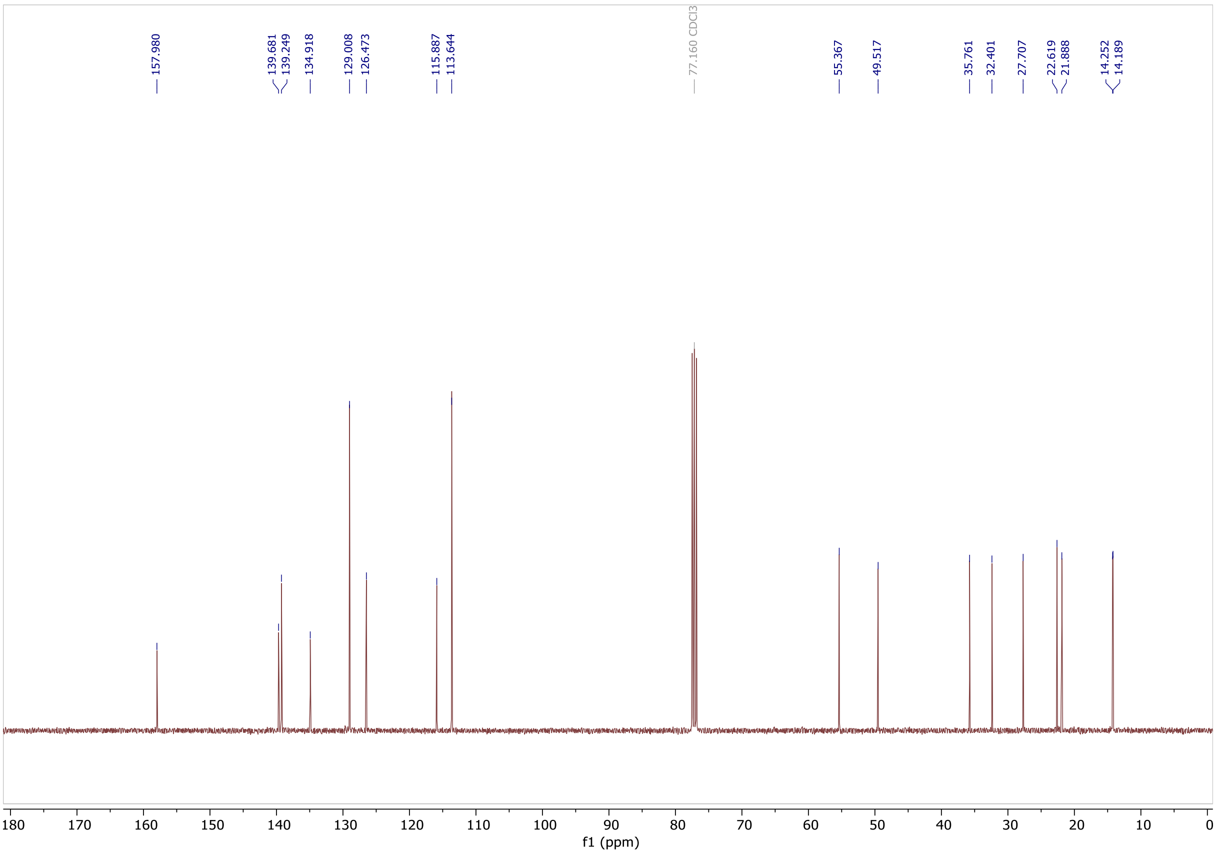

^{1}H NMR (400 MHz, $CDCl_3$) of **28**

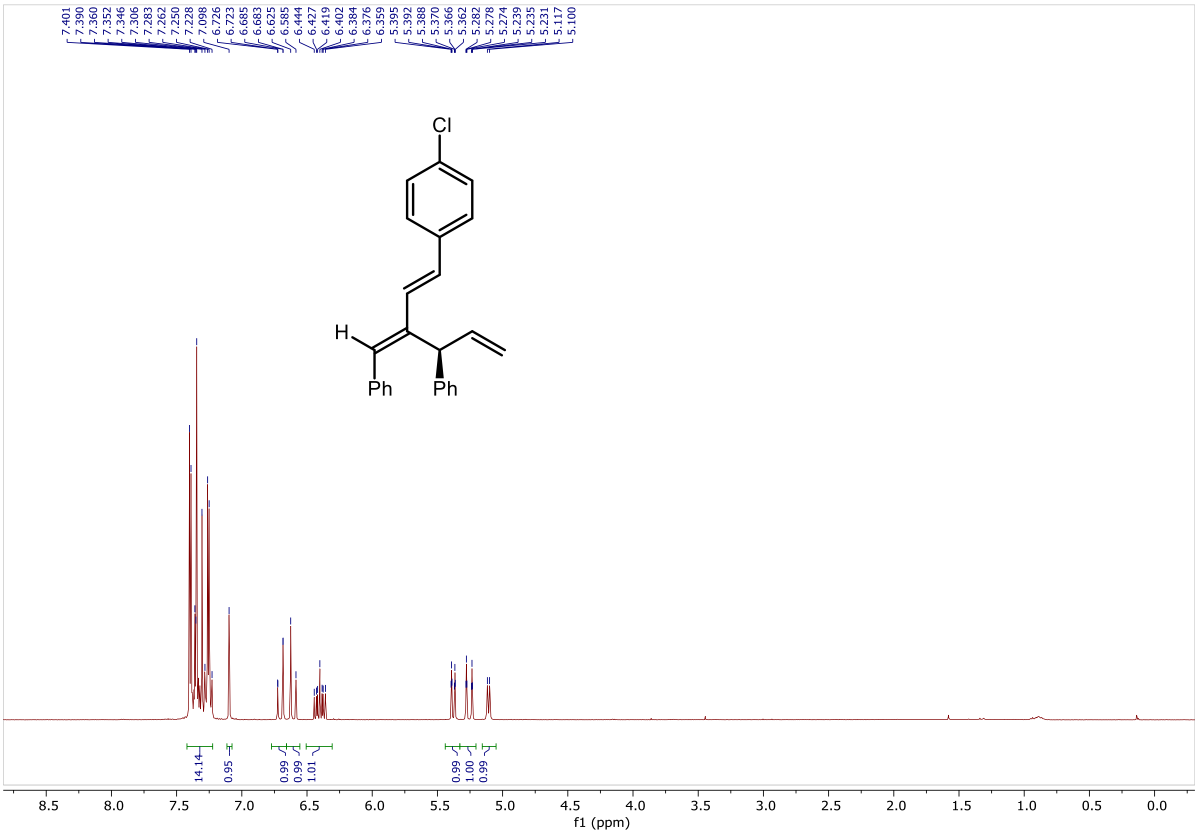

^{13}C NMR (101 MHz, $CDCl_3$) of **28**

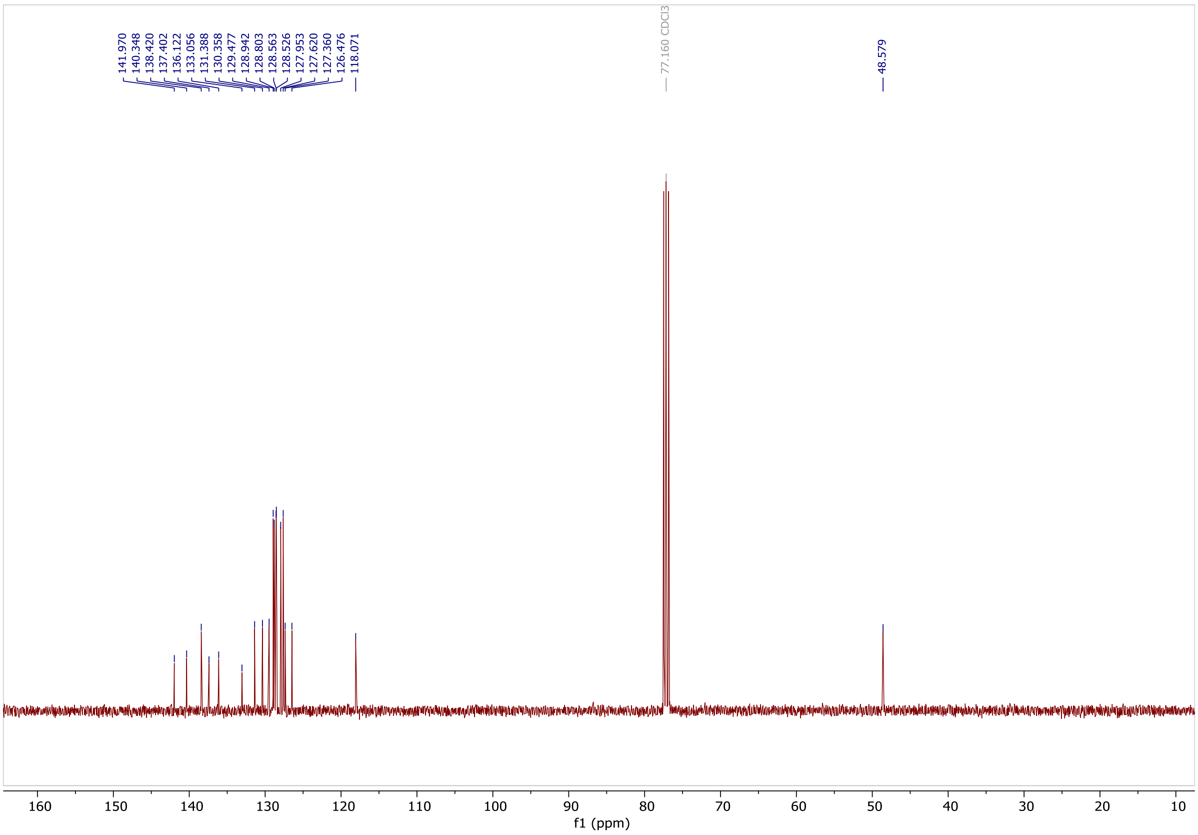

^{1}H NMR (400 MHz, $CDCl_3$) of **29**

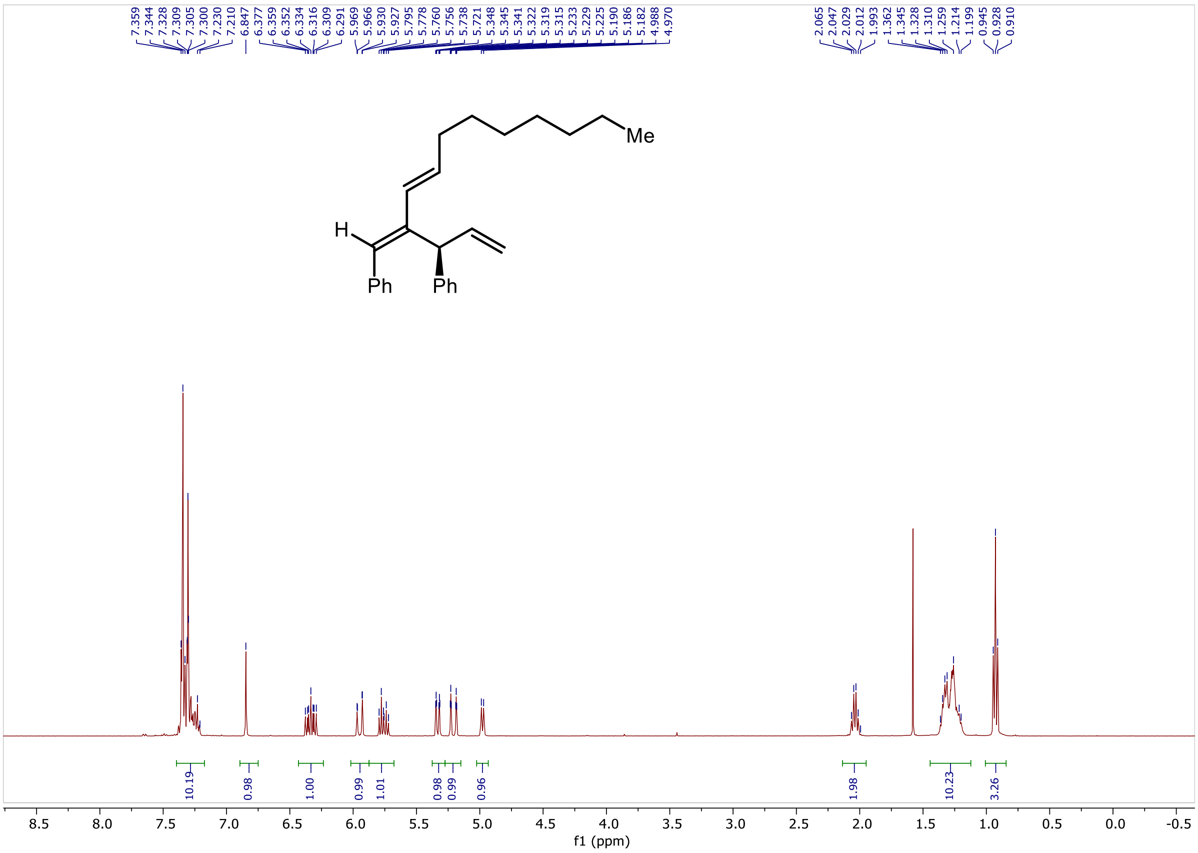

^{13}C NMR (101 MHz, $CDCl_3$) of **29**

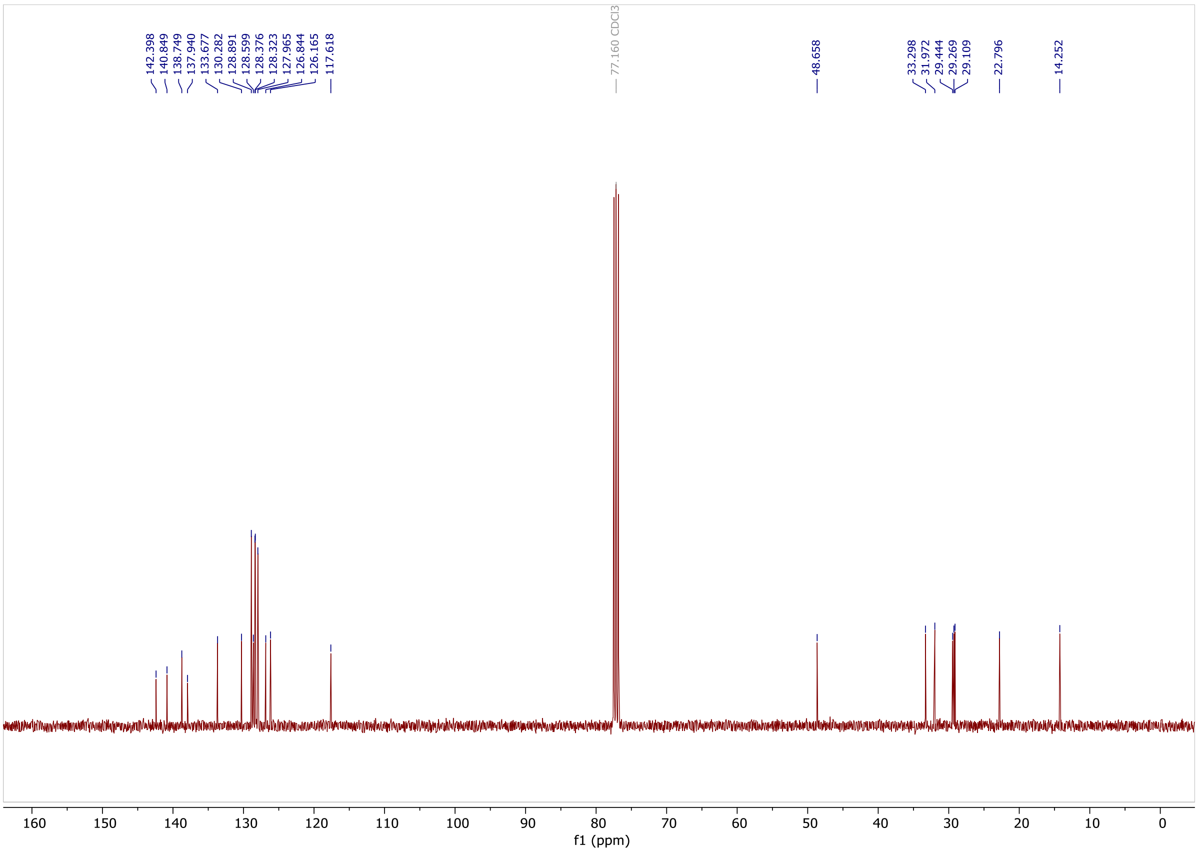

^{1}H NMR (400 MHz, $CDCl_3$) of **30**

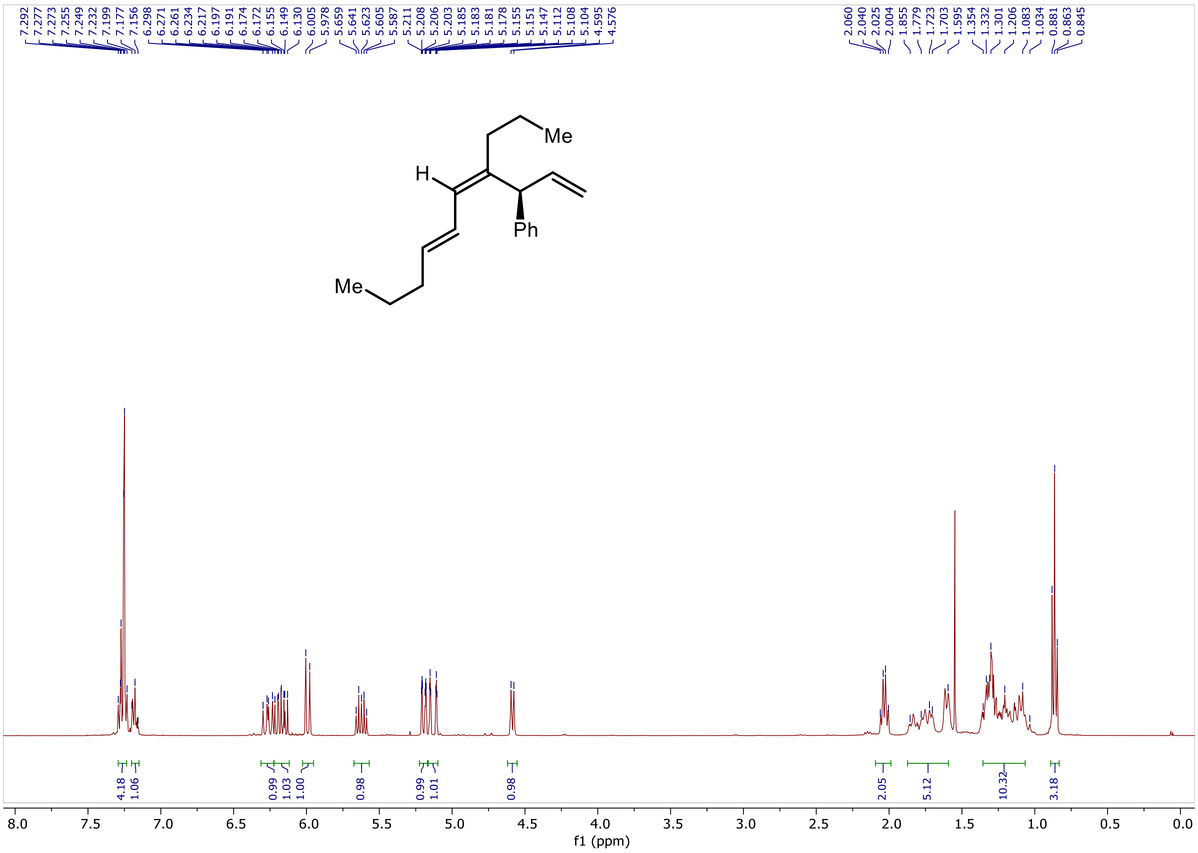

^{13}C NMR (101 MHz, $CDCl_3$) of **30**

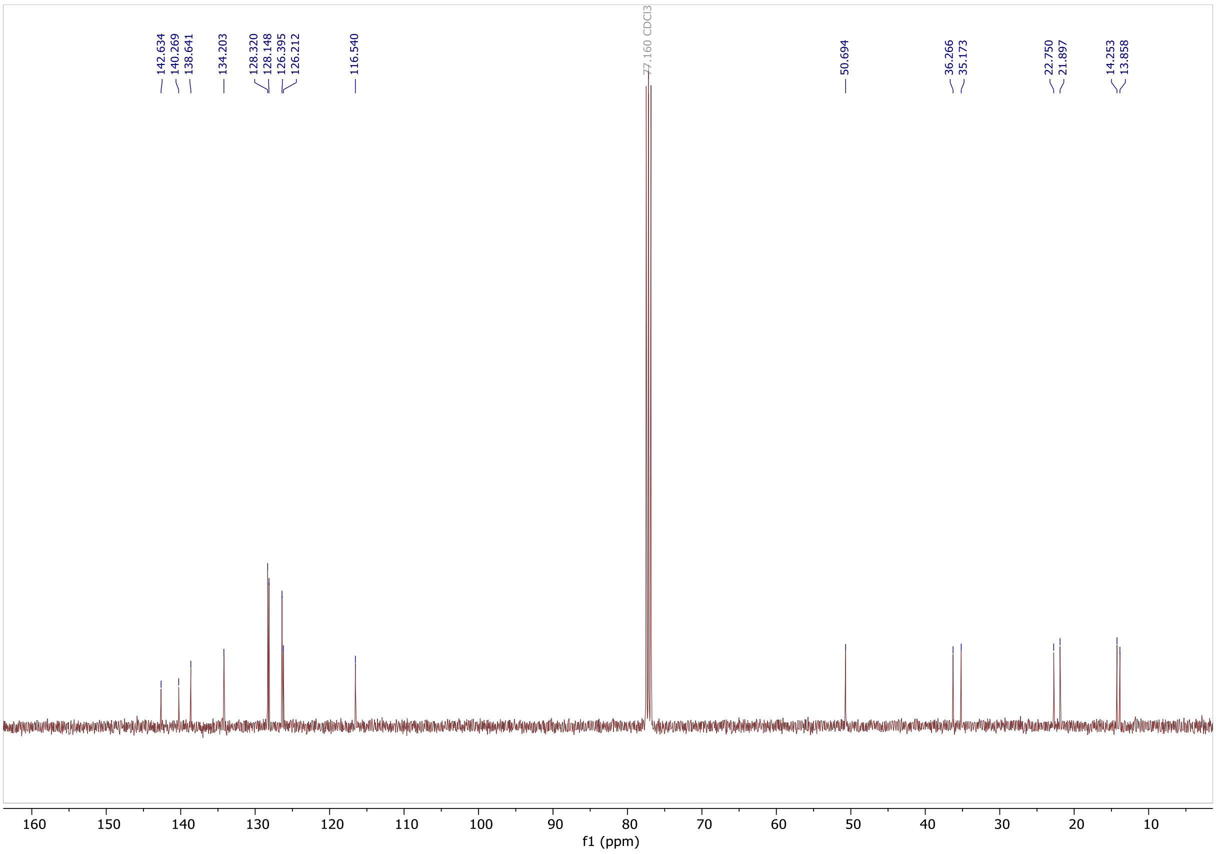

^{1}H NMR (400 MHz, $CDCl_3$) of **31**

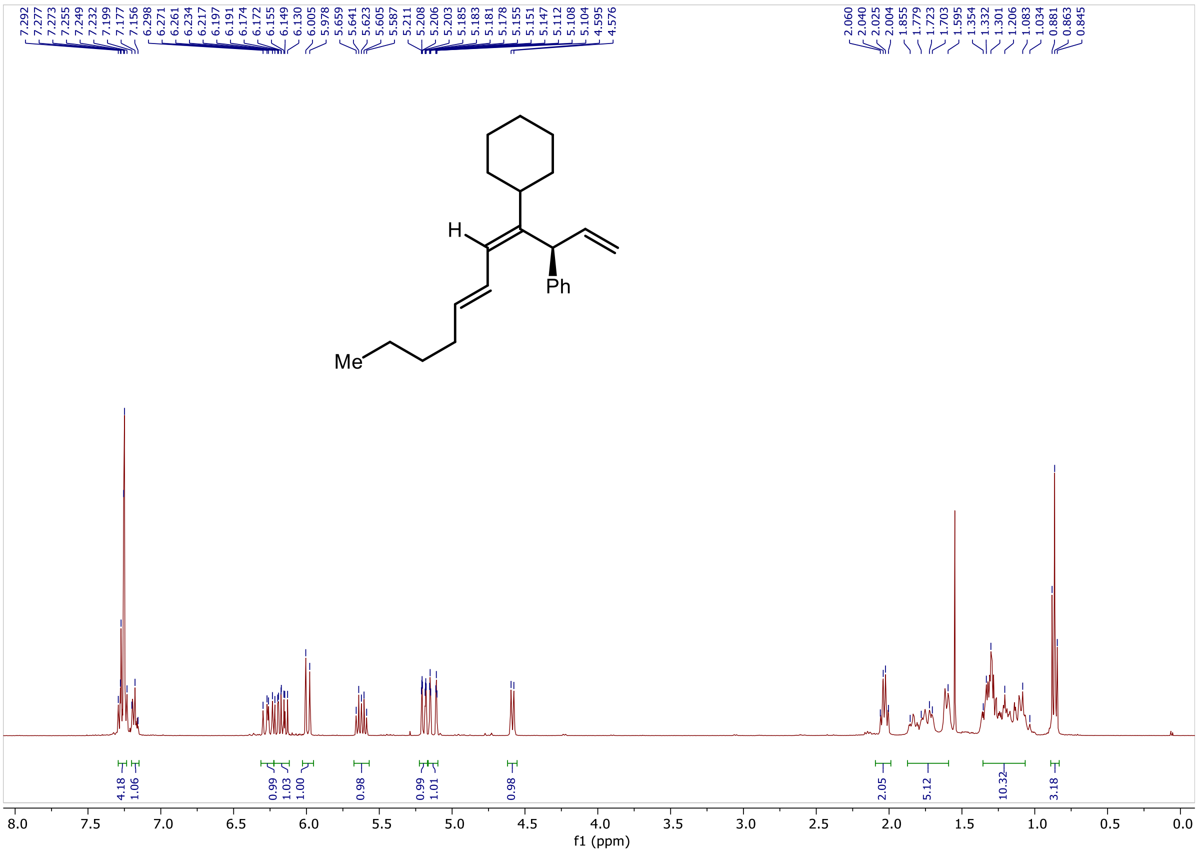

^{13}C NMR (101 MHz, $CDCl_3$) of **31**

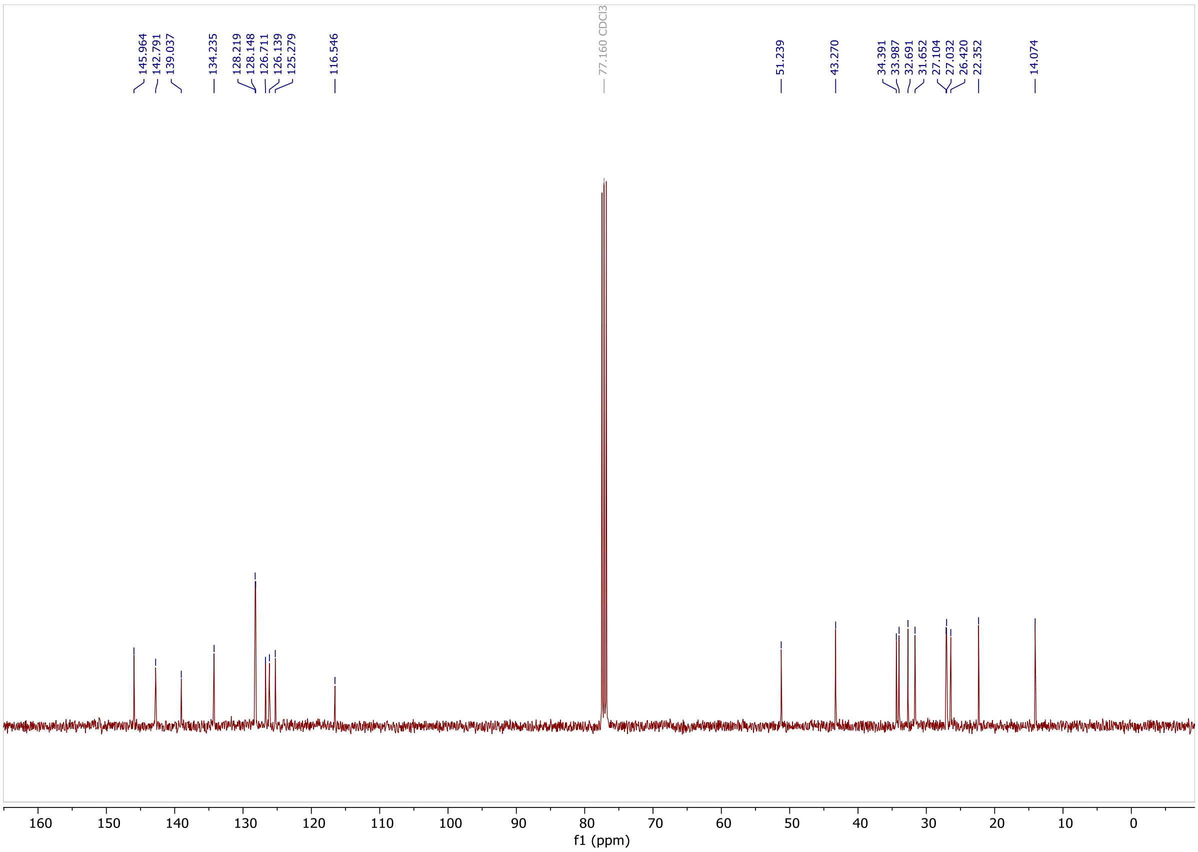

^{1}H NMR (400 MHz, $CDCl_3$) of **32**

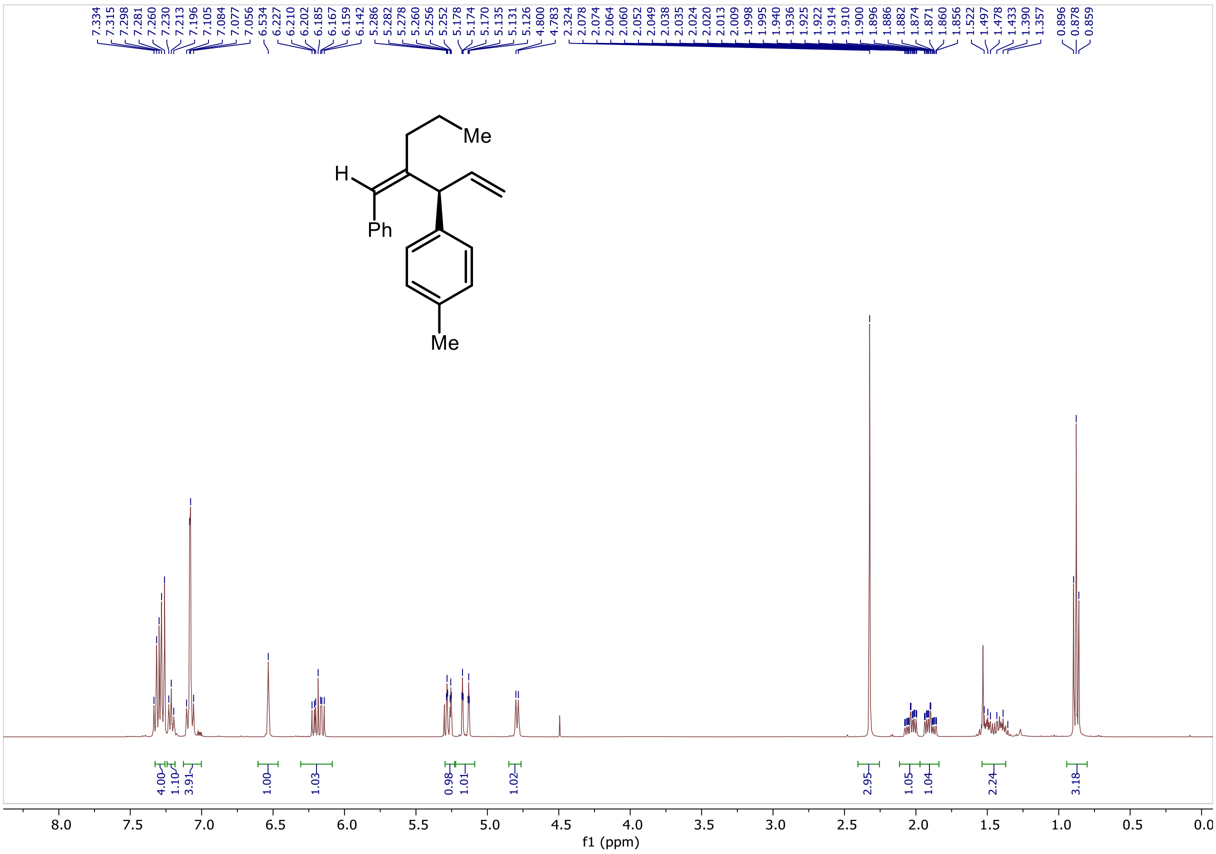

^{13}C NMR (101 MHz, $CDCl_3$) of **32**

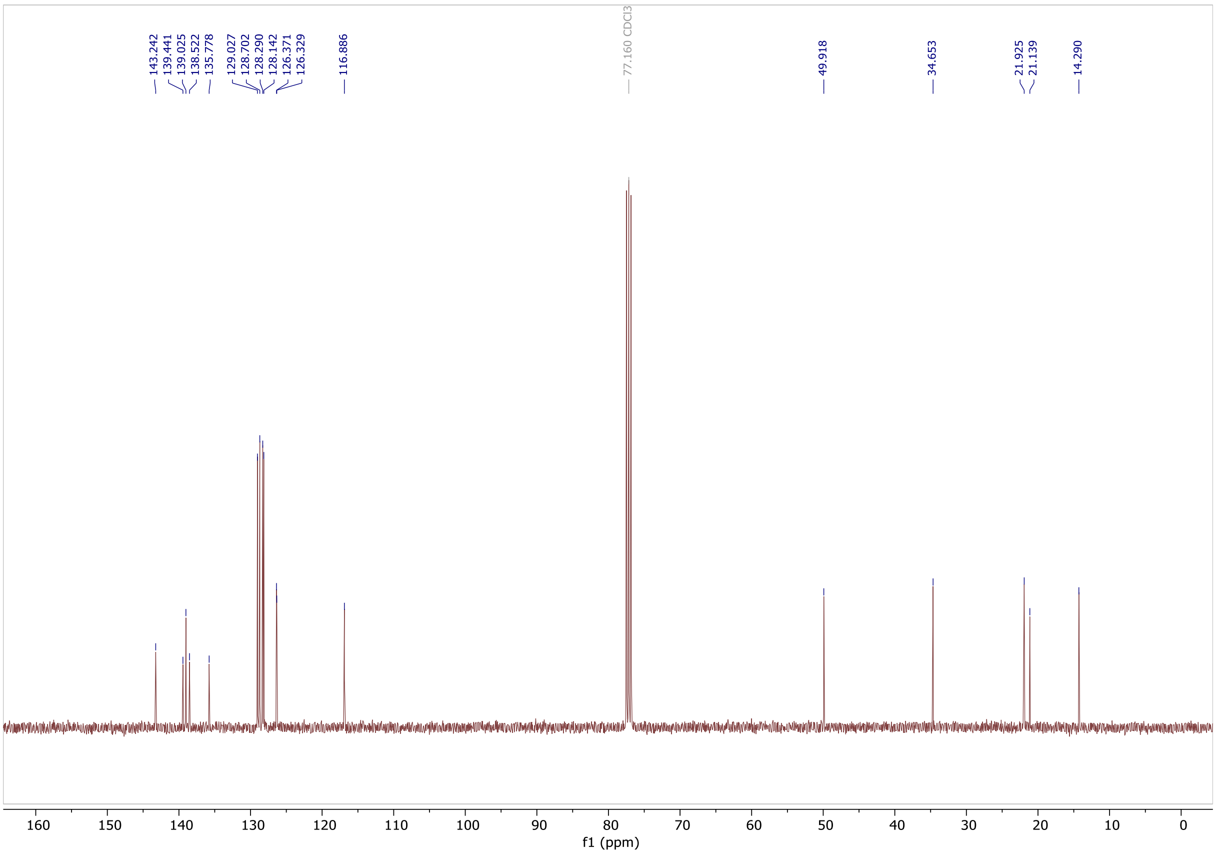

^{1}H NMR (400 MHz, $CDCl_3$) of **33**

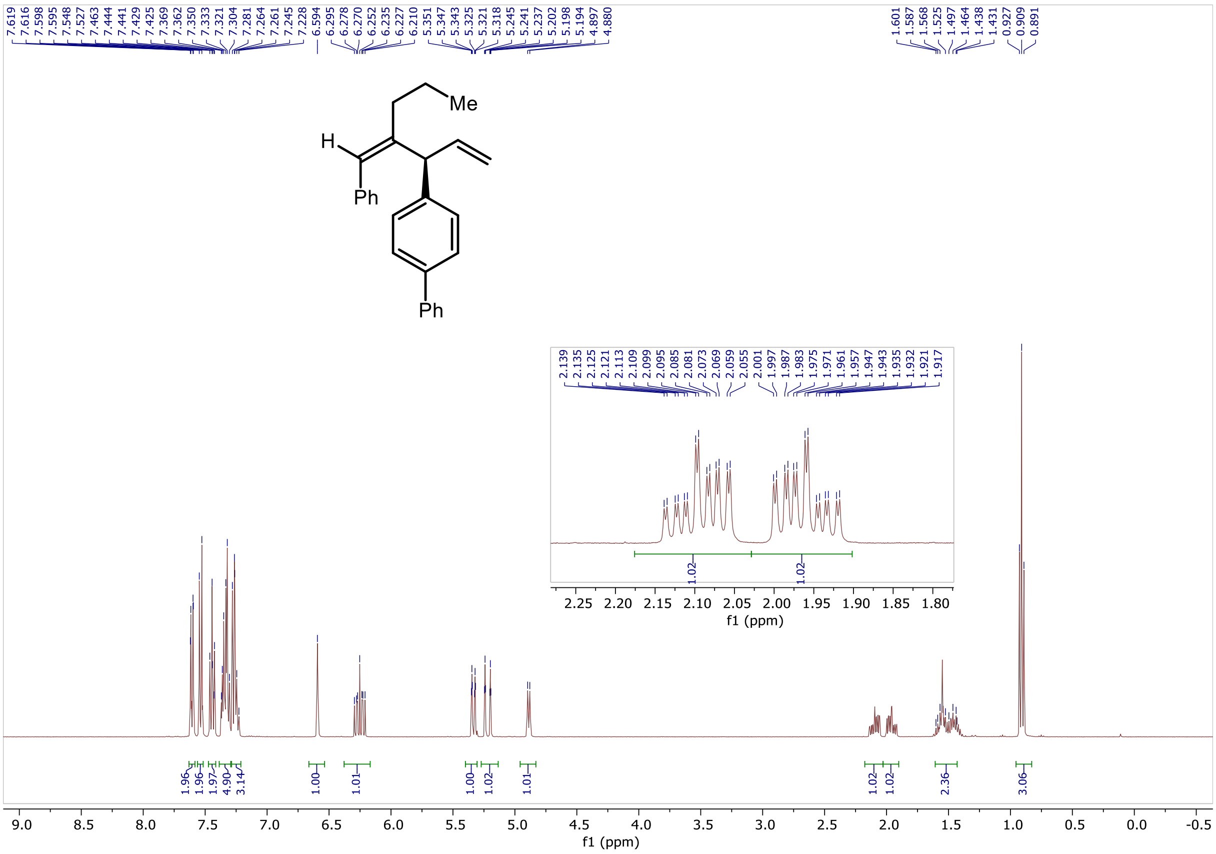

^{13}C NMR (101 MHz, $CDCl_3$) of **33**

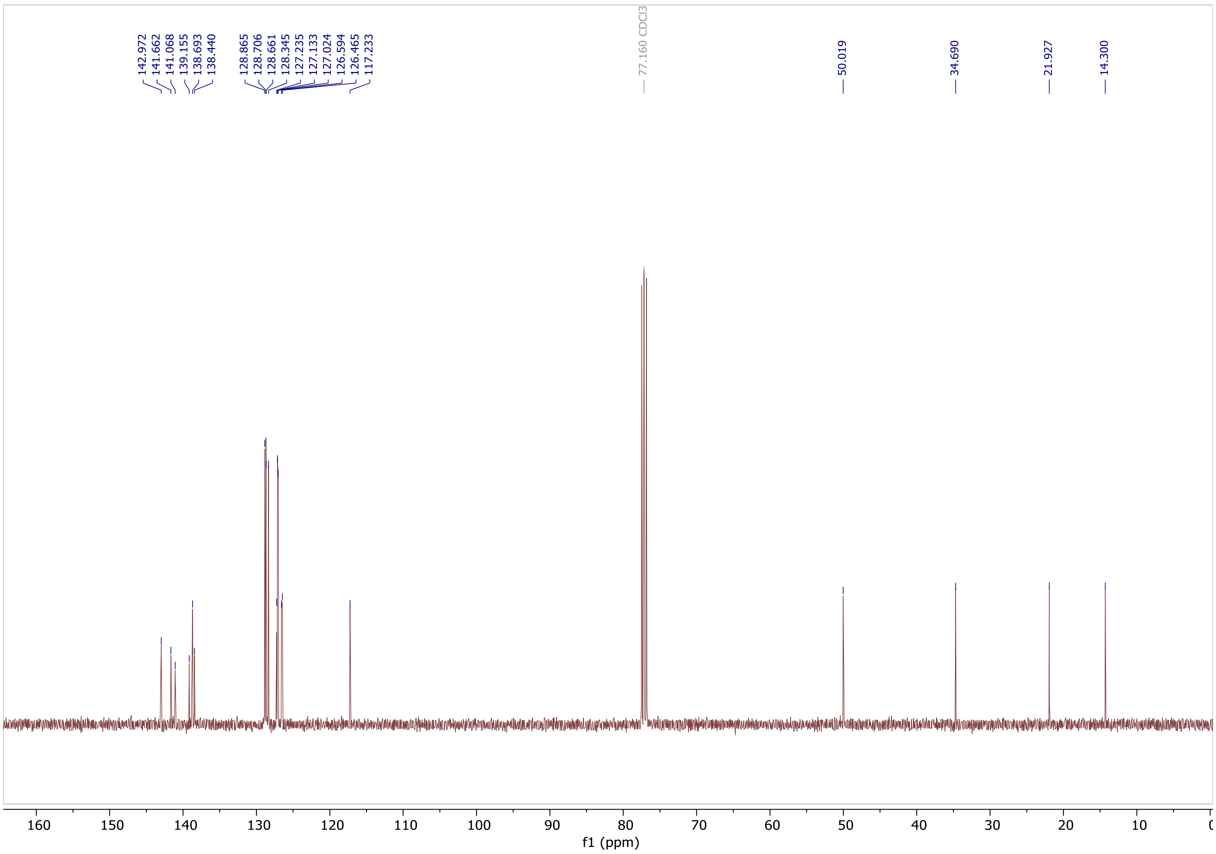

^{1}H NMR (400 MHz, $CDCl_3$) of **34**

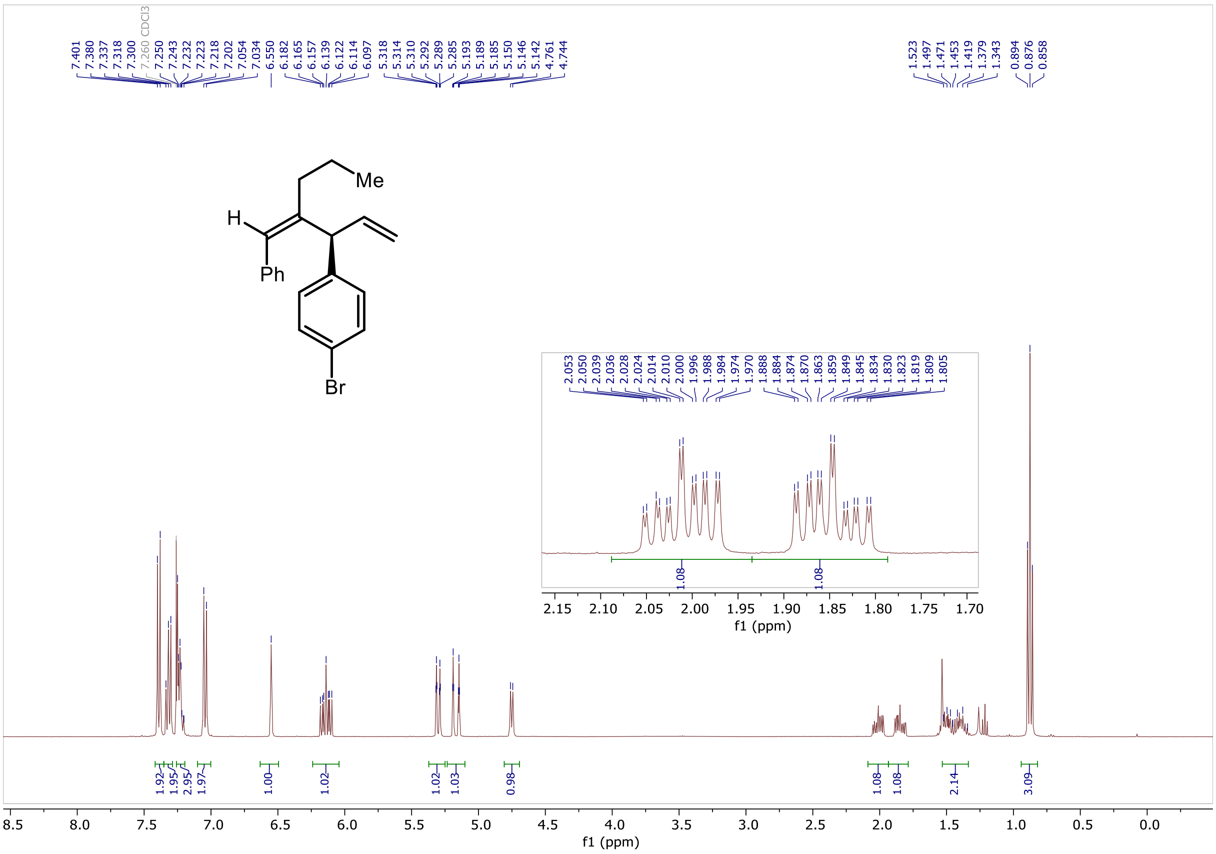

^{13}C NMR (101 MHz, $CDCl_3$) of **34**

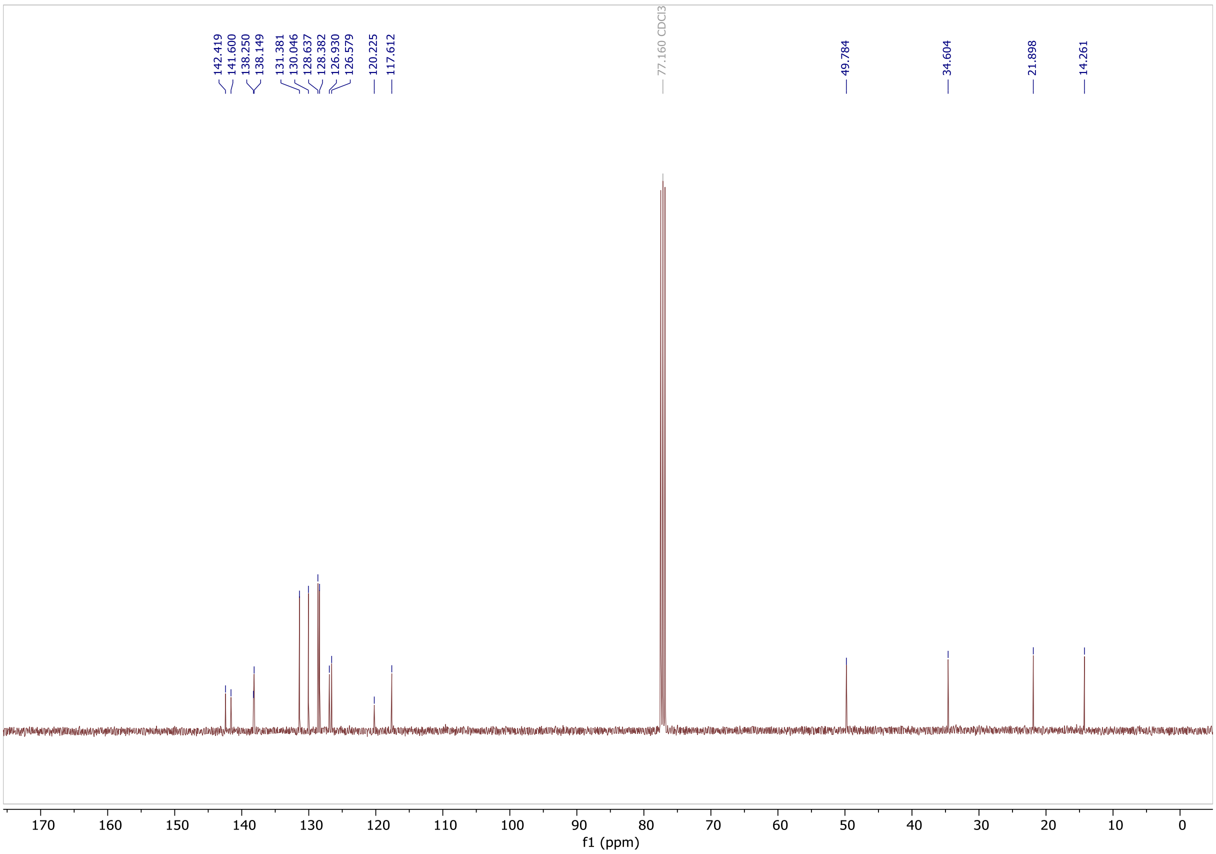

^{1}H NMR (400 MHz, $CDCl_3$) of **35**

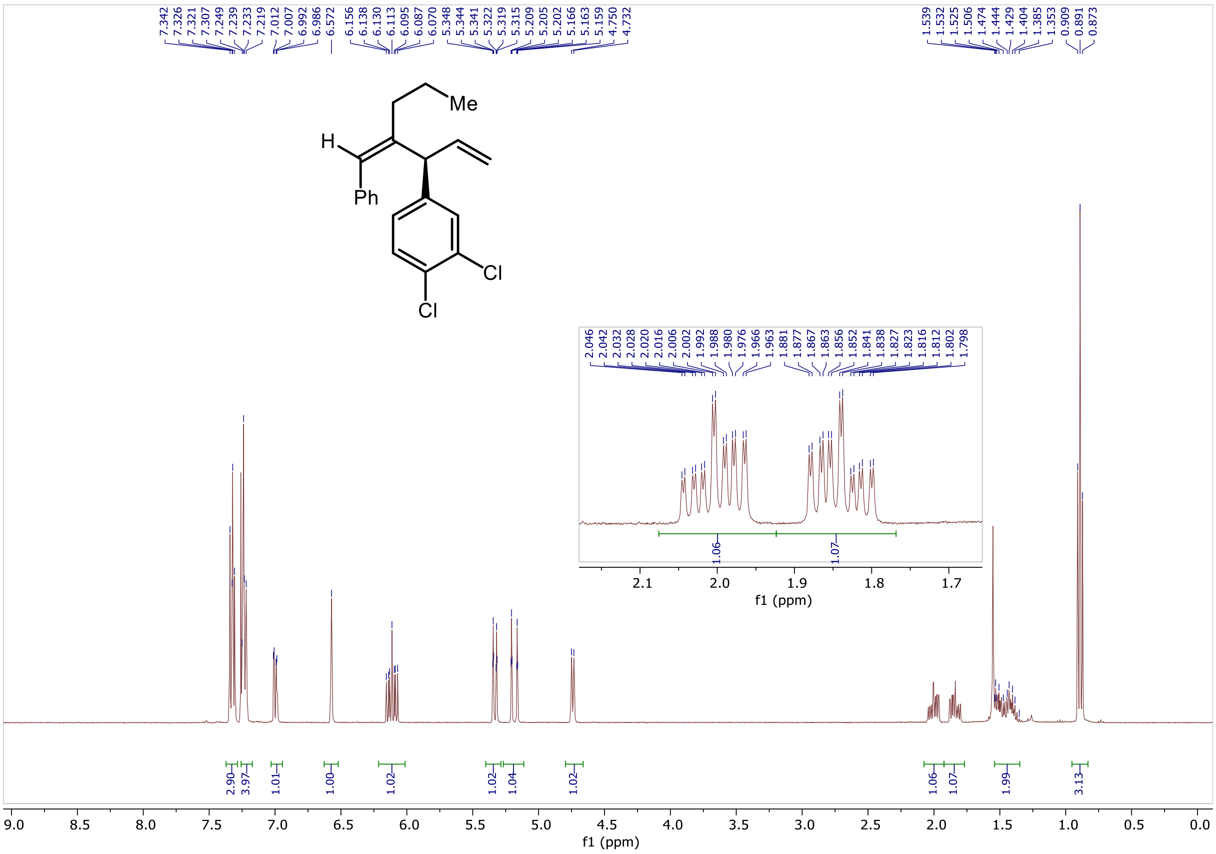

^{13}C NMR (101 MHz, $CDCl_3$) of **35**

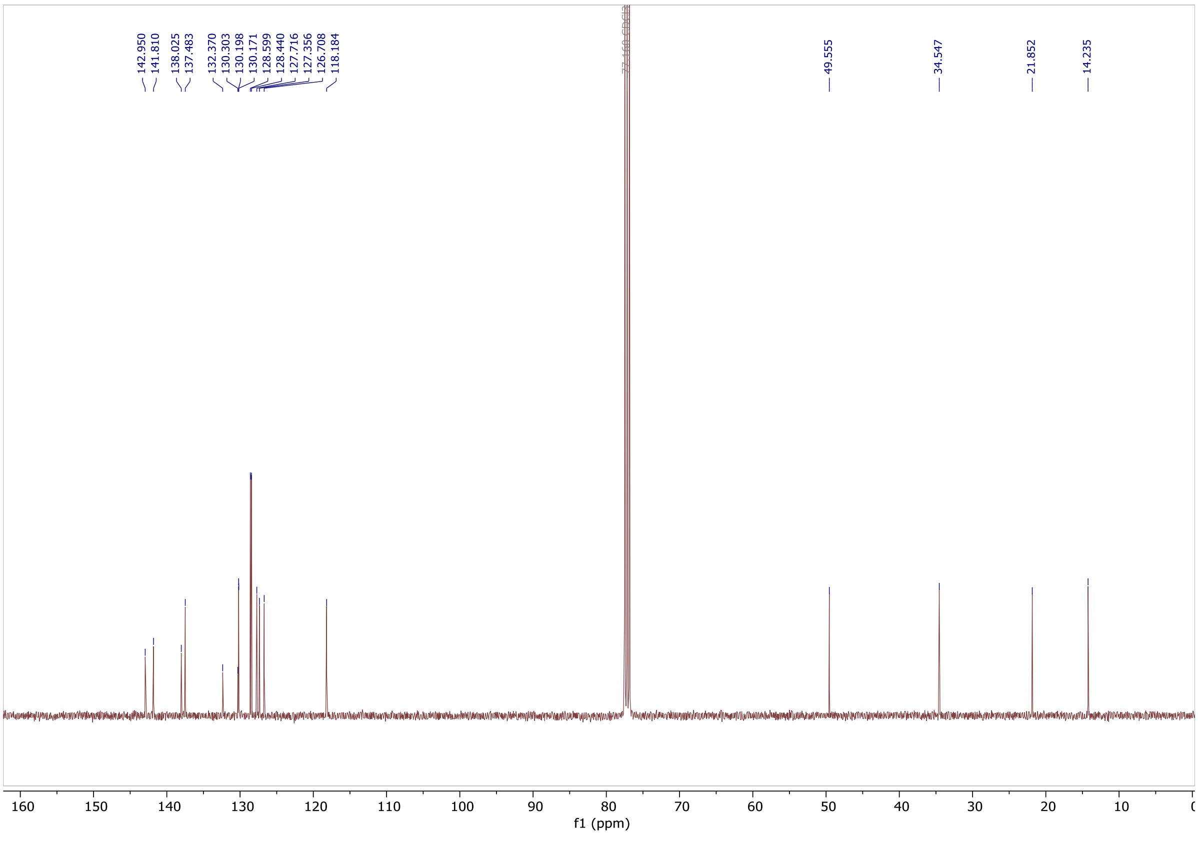

^{1}H NMR (400 MHz, $CDCl_3$) of **36**

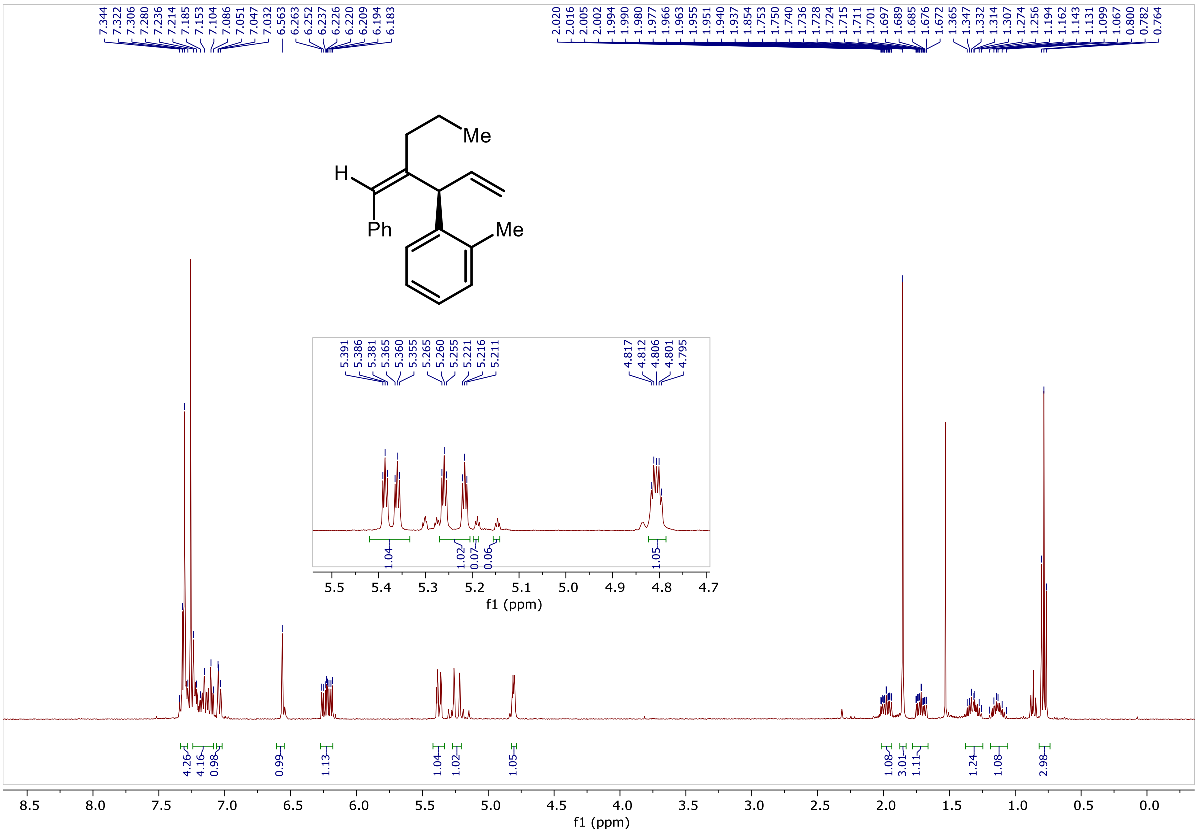

^{13}C NMR (101 MHz, $CDCl_3$) of **36**

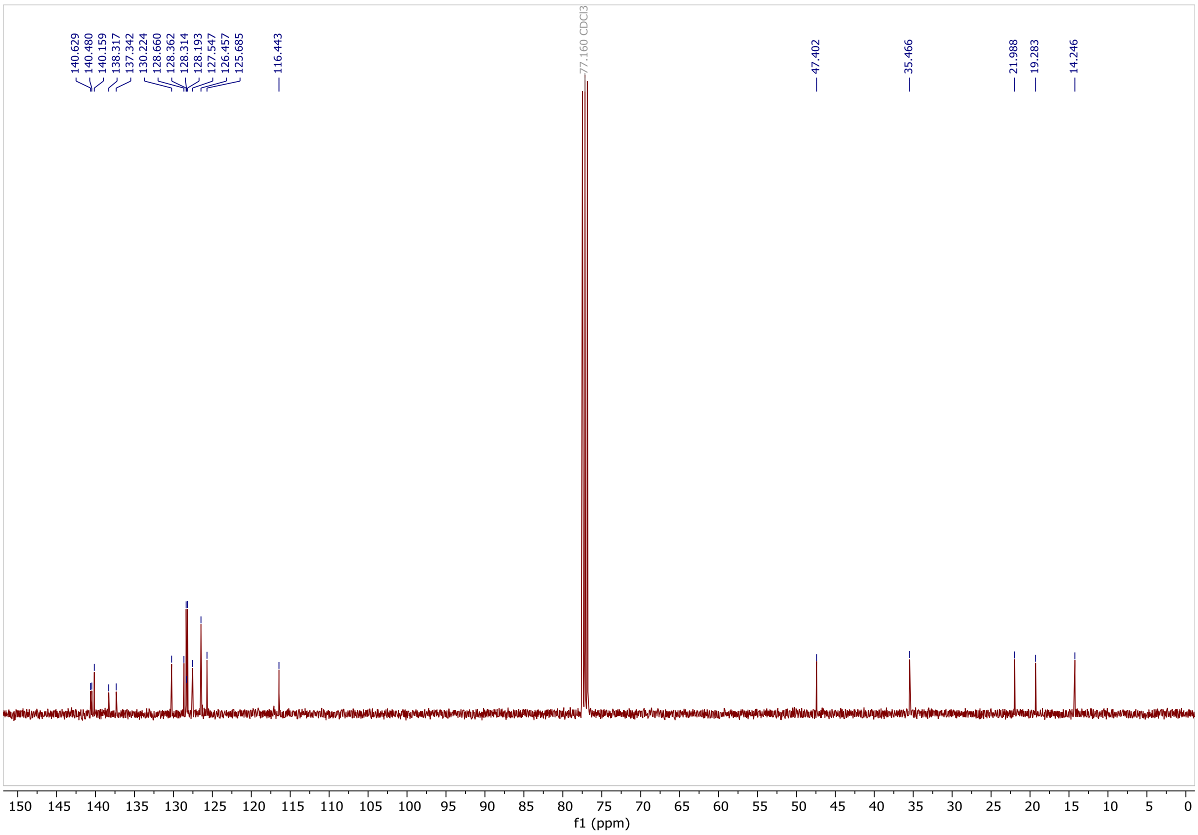

^{1}H NMR (400 MHz, $CDCl_3$) of **37**

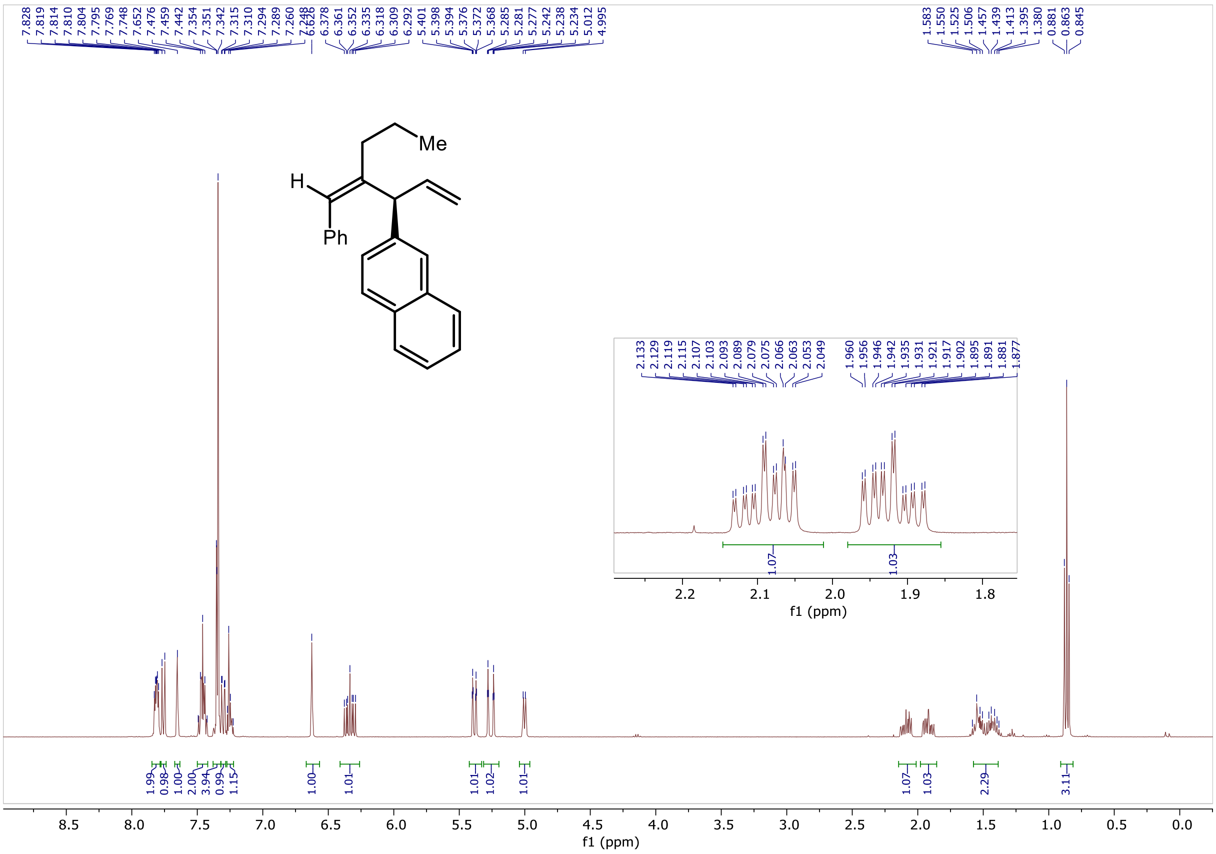

^{13}C NMR (101 MHz, $CDCl_3$) of **37**

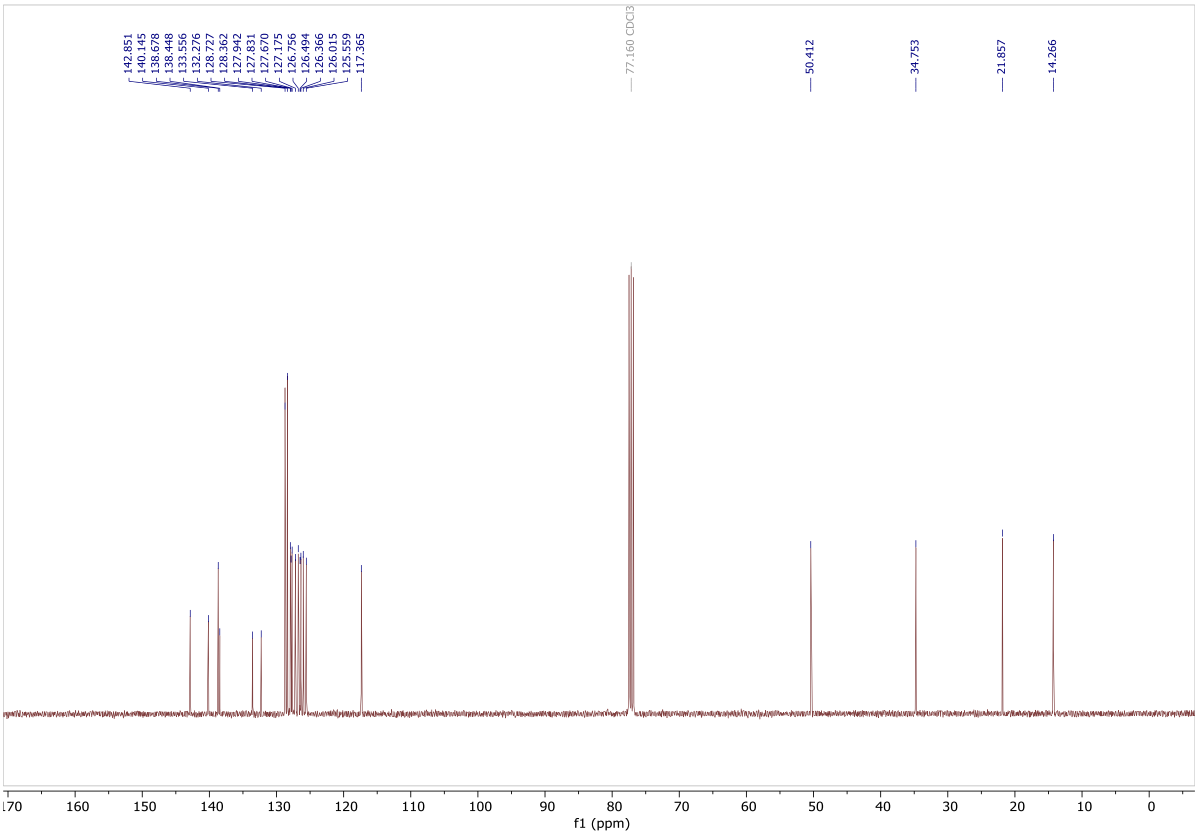

^{1}H NMR (400 MHz, $CDCl_3$) of **38**

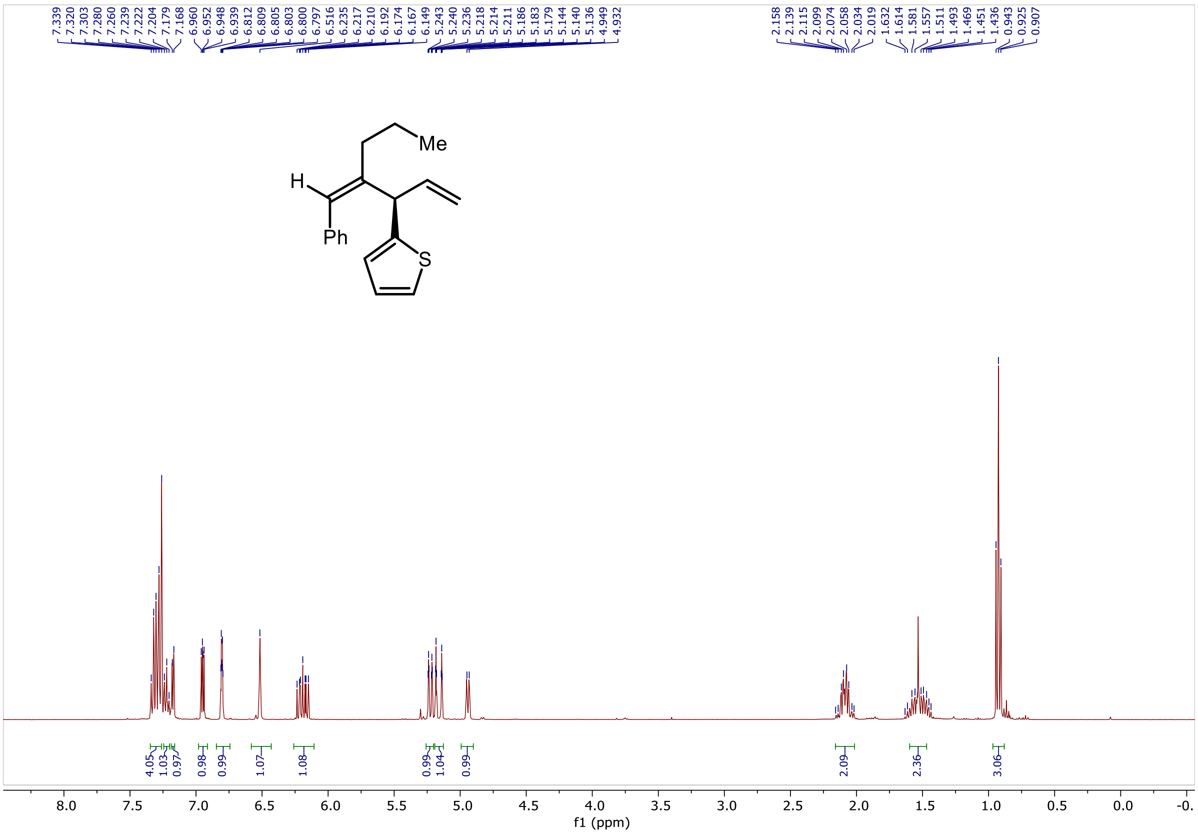

^{13}C NMR (101 MHz, $CDCl_3$) of **38**

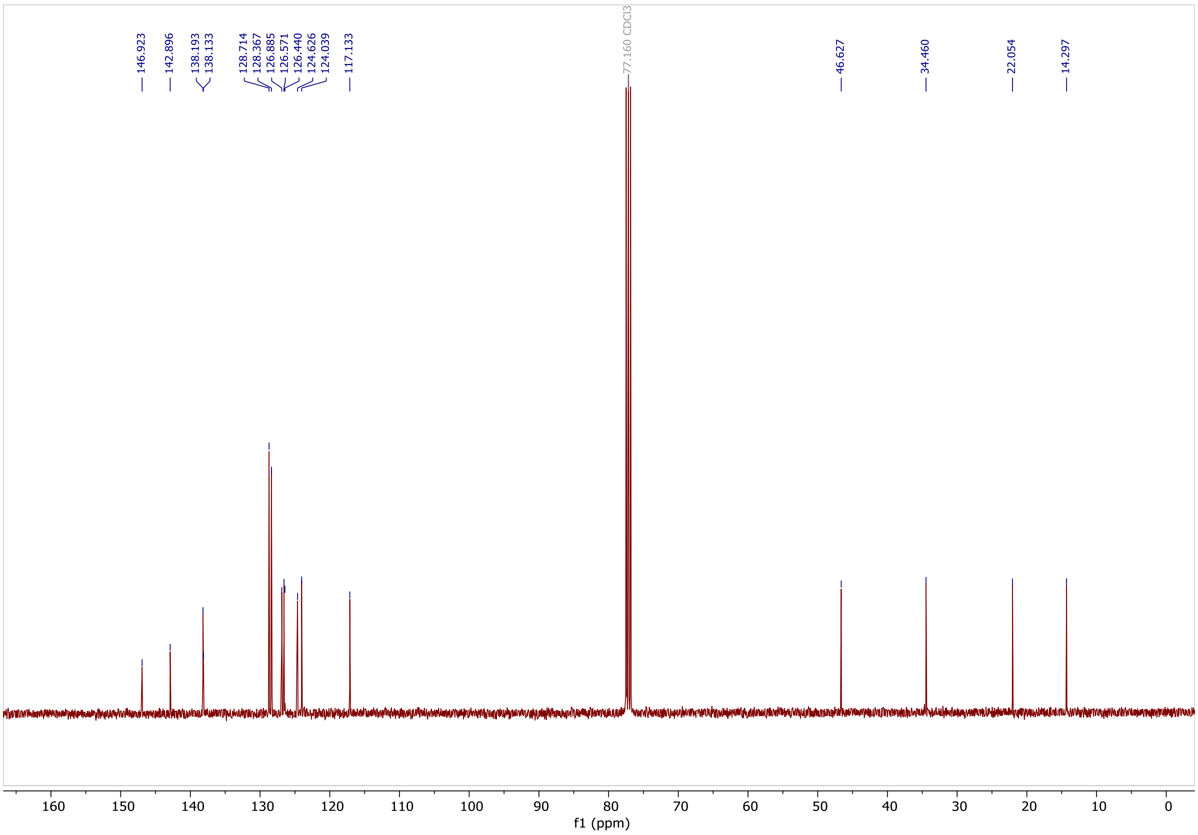

^{1}H NMR (400 MHz, $CDCl_3$) of **39**

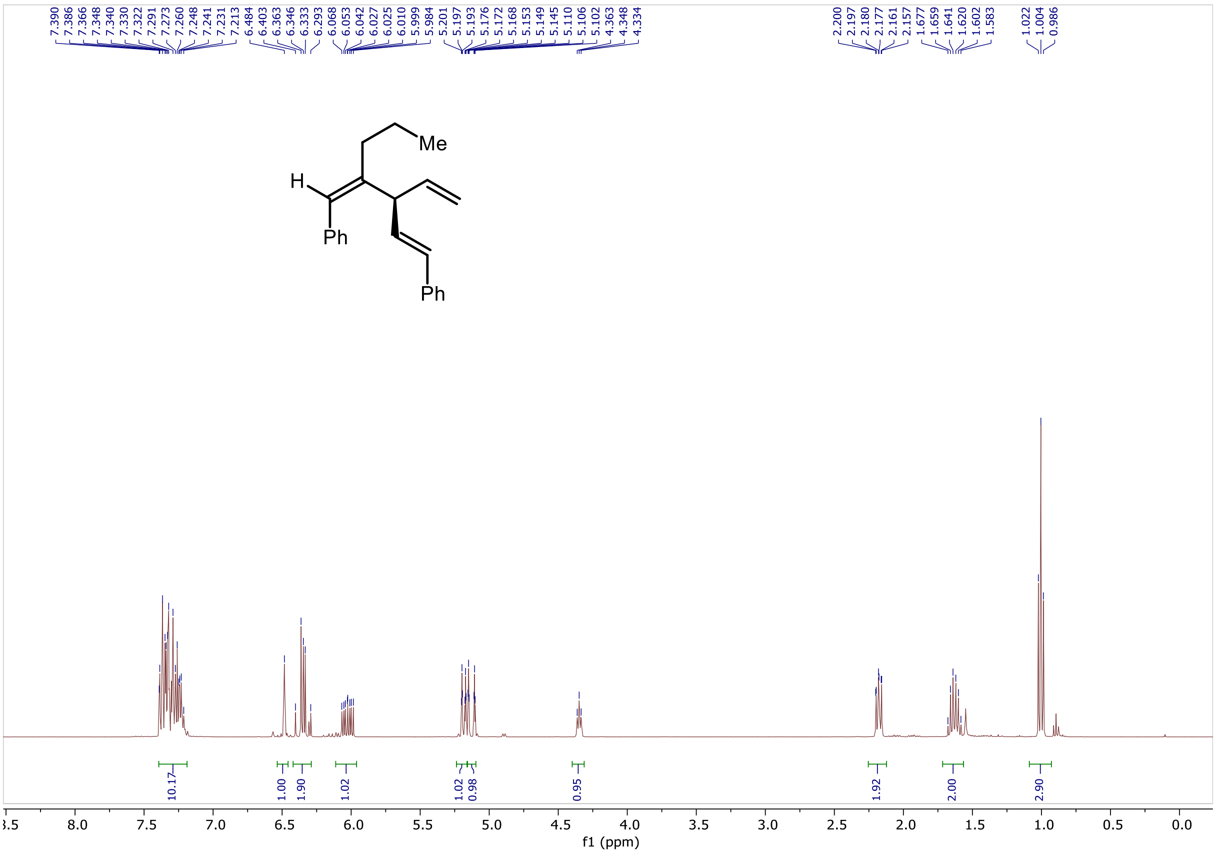

^{13}C NMR (101 MHz, $CDCl_3$) of **39**

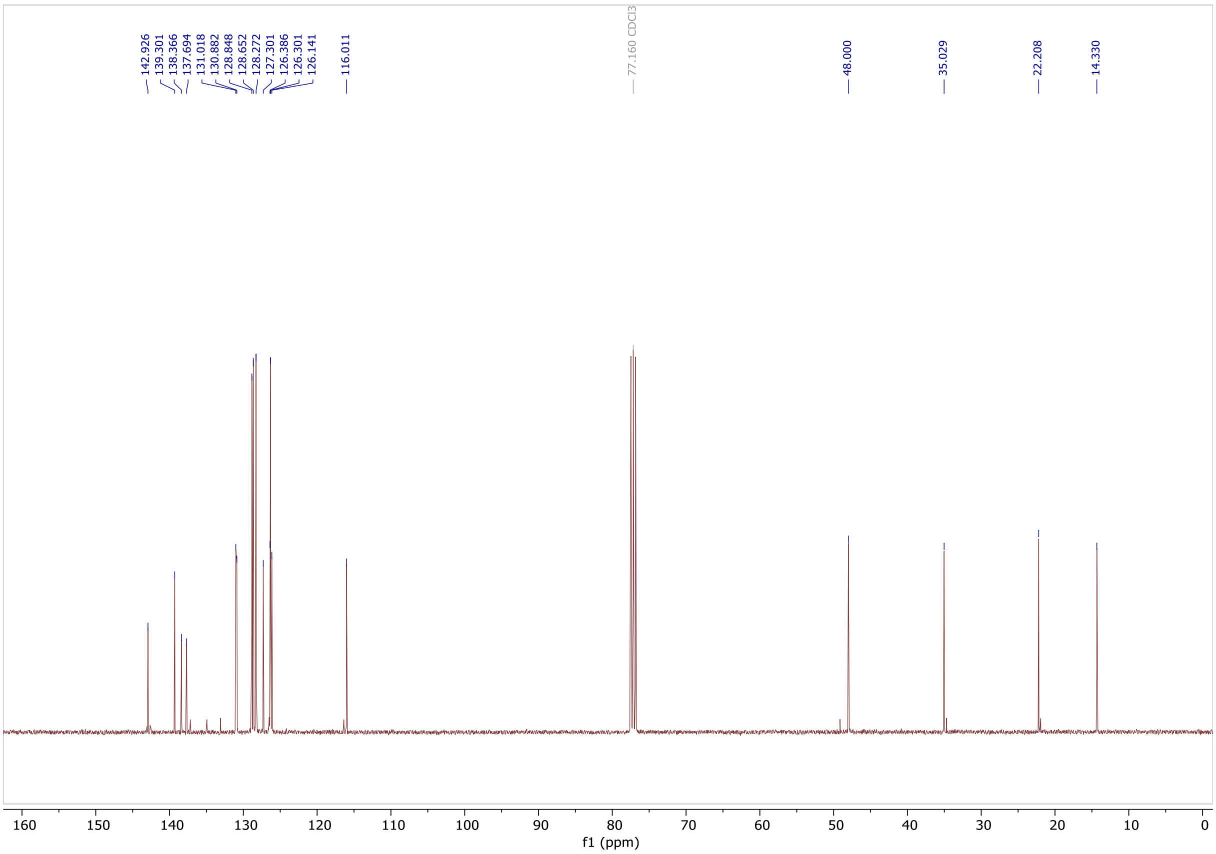

^{1}H NMR (400 MHz, $CDCl_3$) of **40**

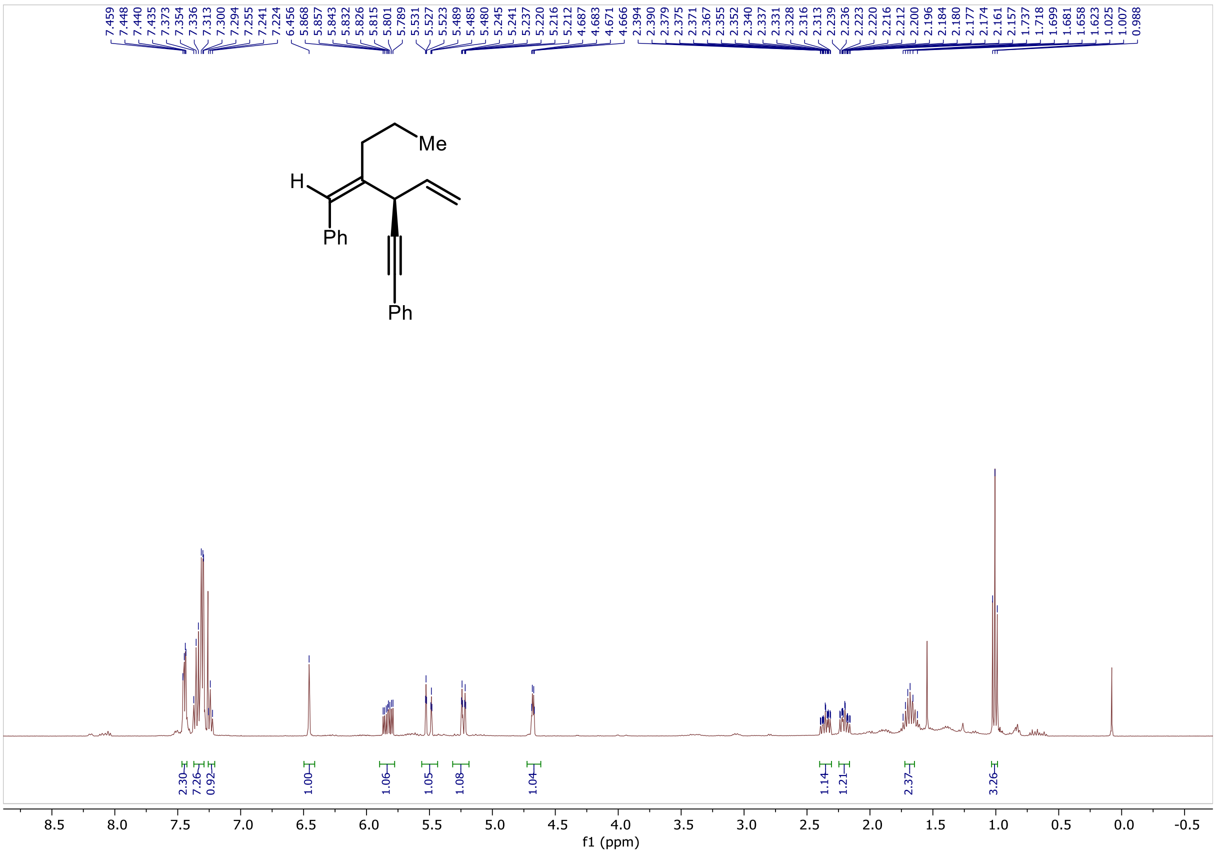

^{13}C NMR (101 MHz, $CDCl_3$) of **40**

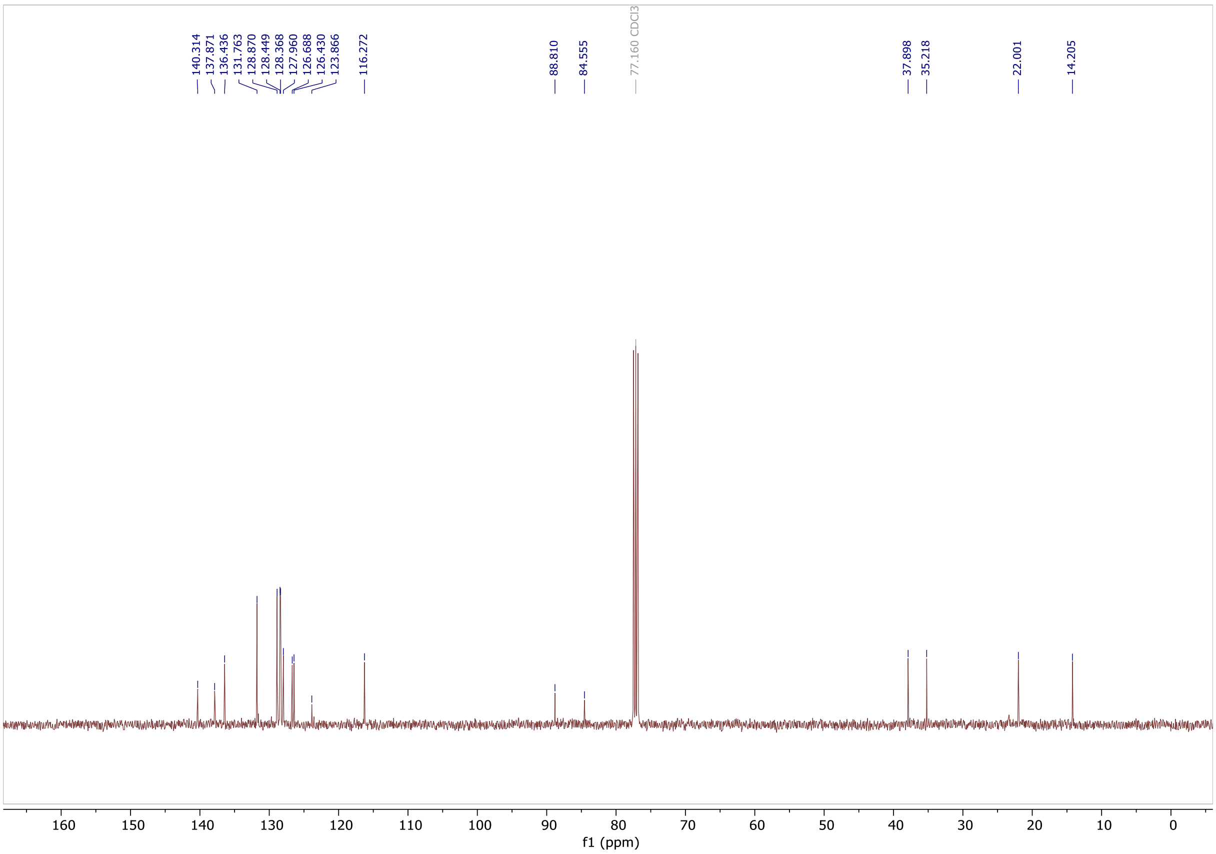

^{1}H NMR (400 MHz, $CDCl_3$) of **41**

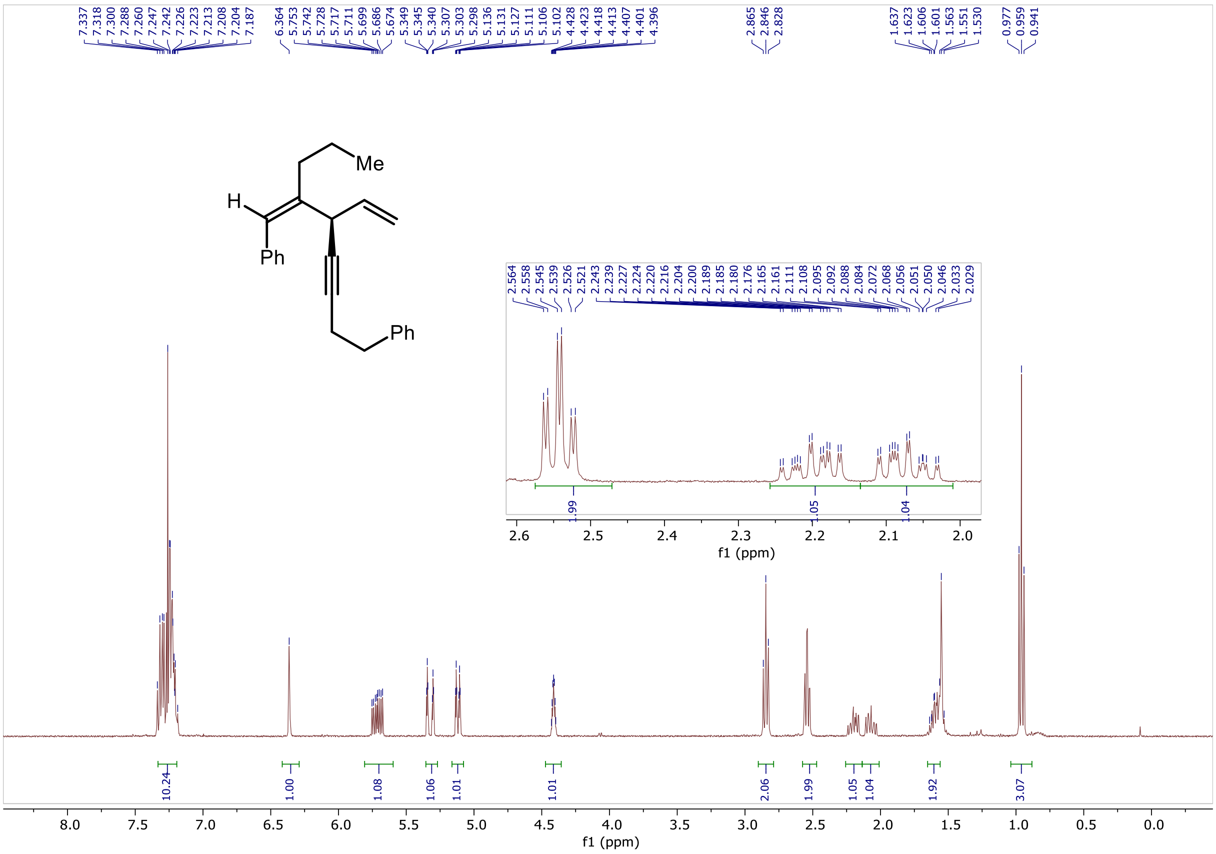

^{13}C NMR (101 MHz, $CDCl_3$) of **41**

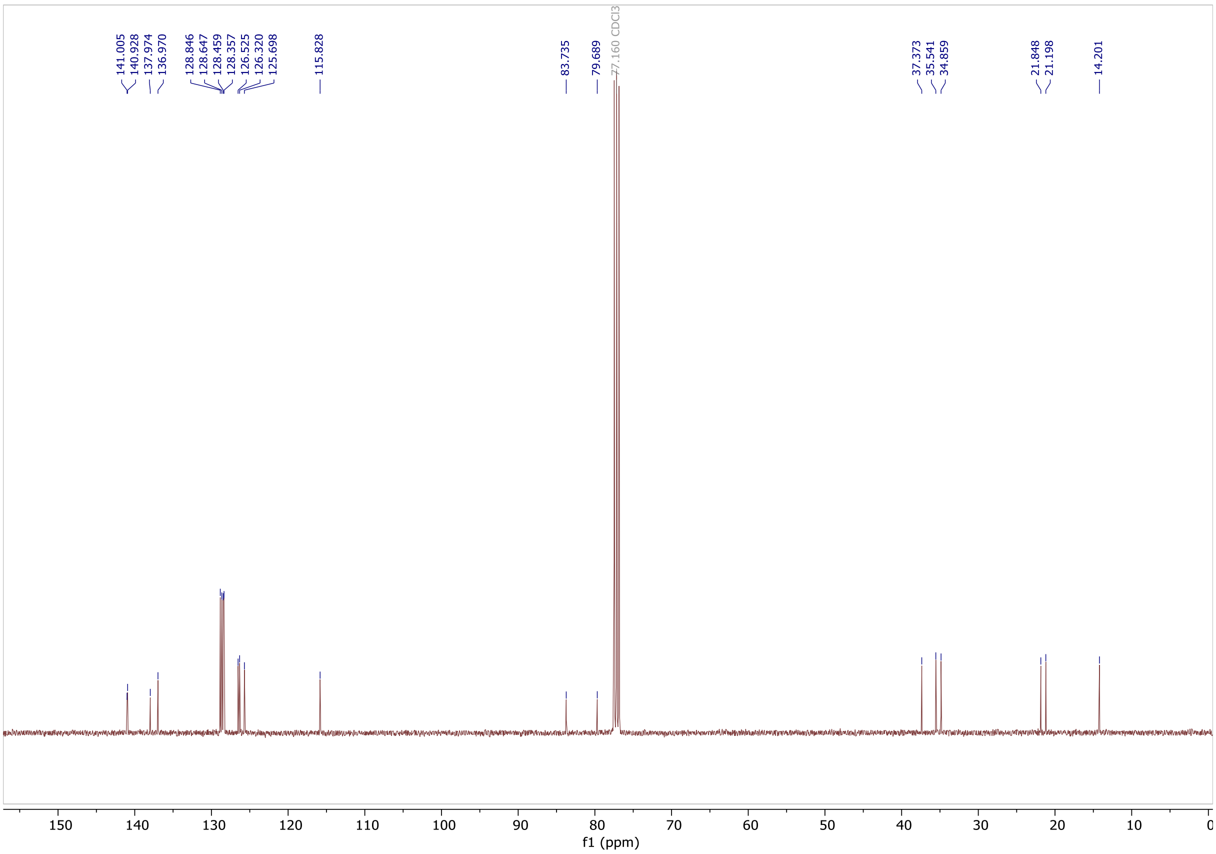

^{1}H NMR (400 MHz, $CDCl_3$) of **42**

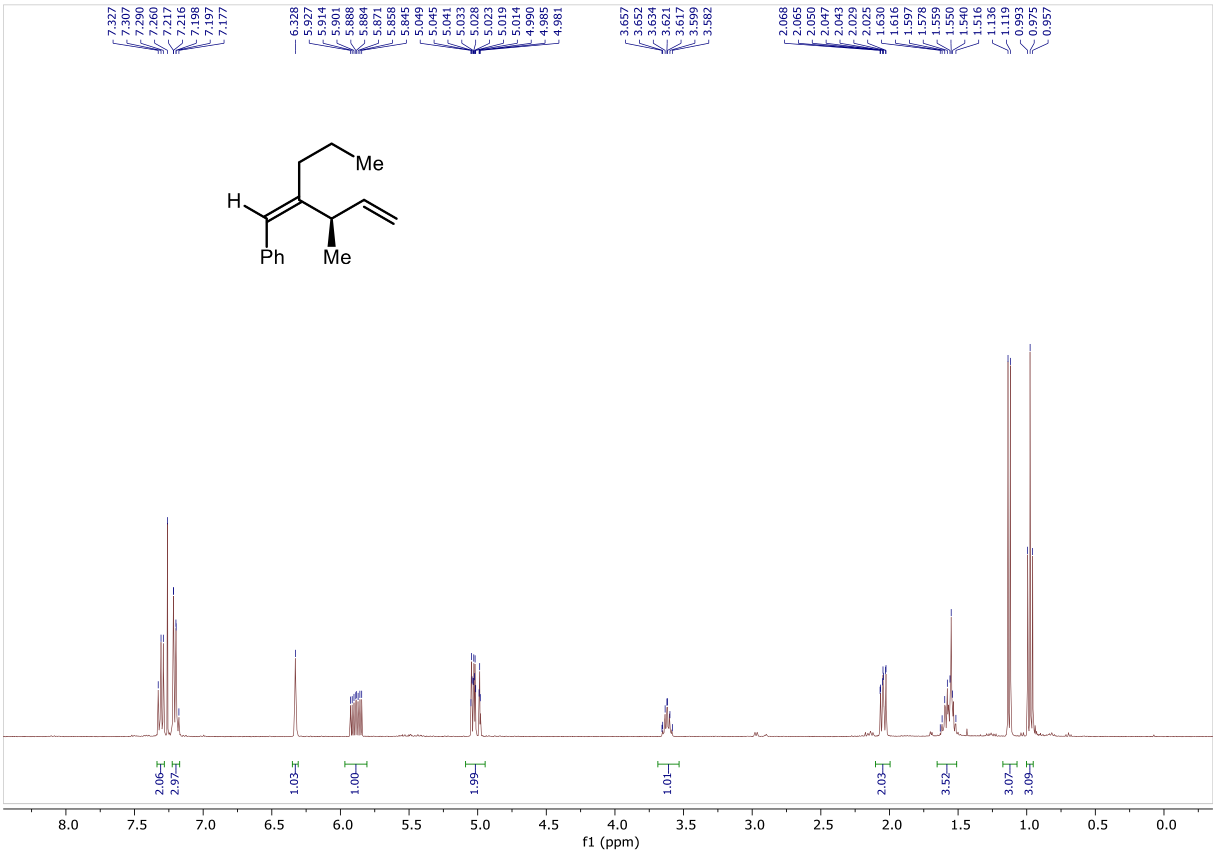

^{13}C NMR (101 MHz, $CDCl_3$) of **42**

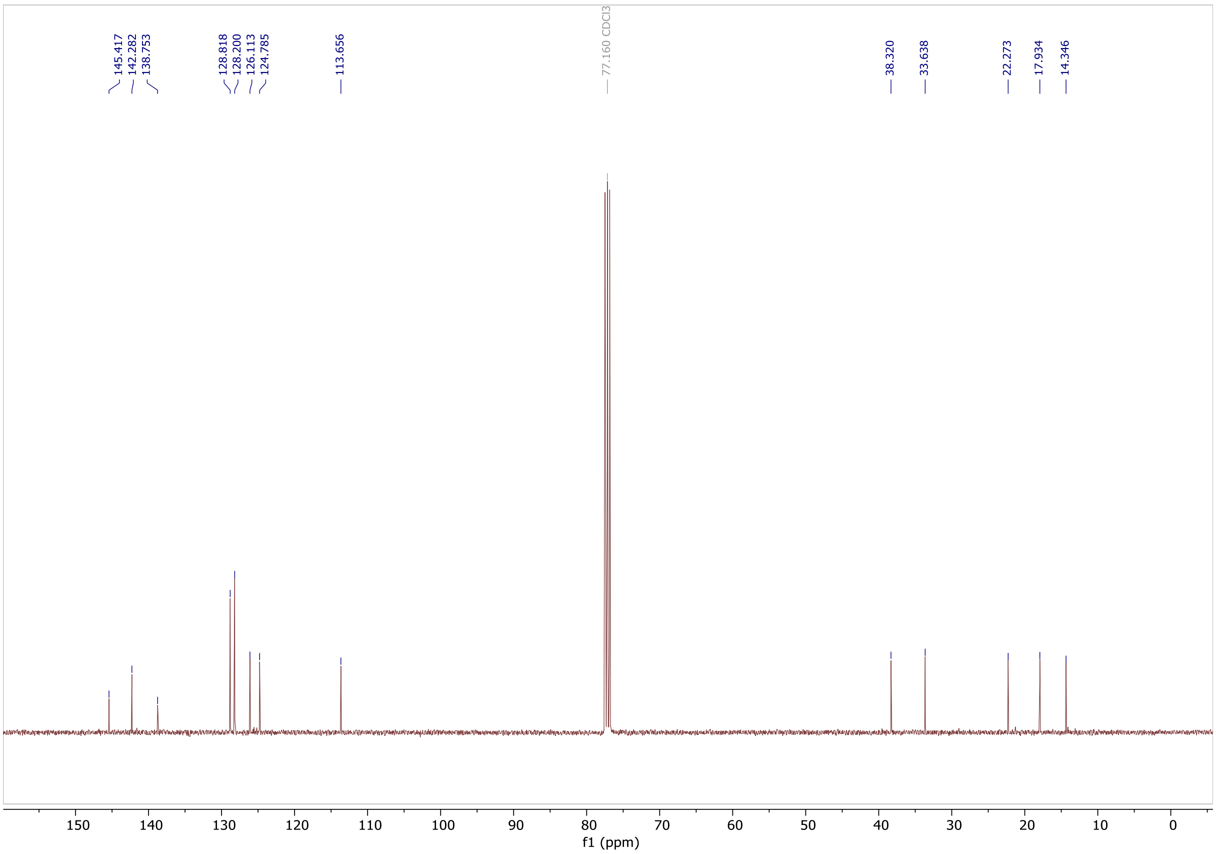

^{1}H NMR (400 MHz, $CDCl_3$) of **43**

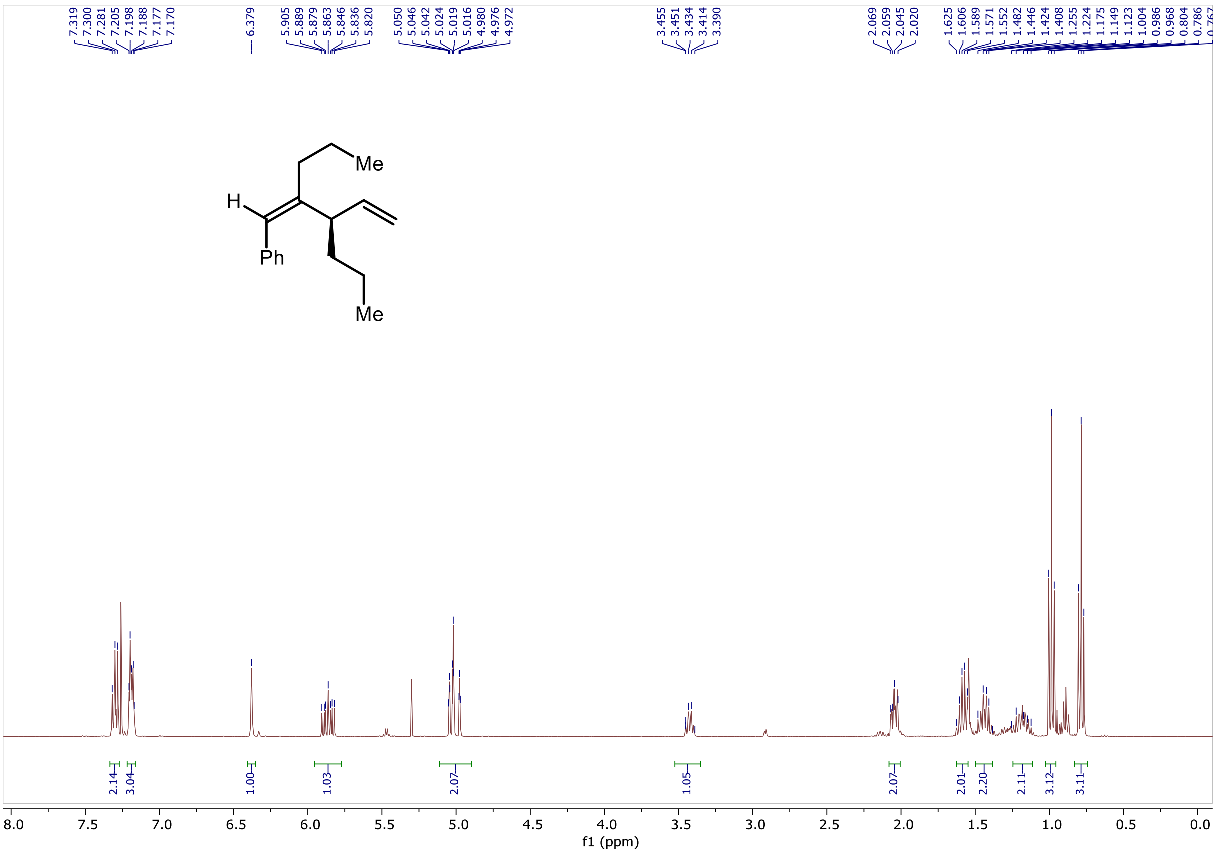

^{13}C NMR (101 MHz, $CDCl_3$) of **43**

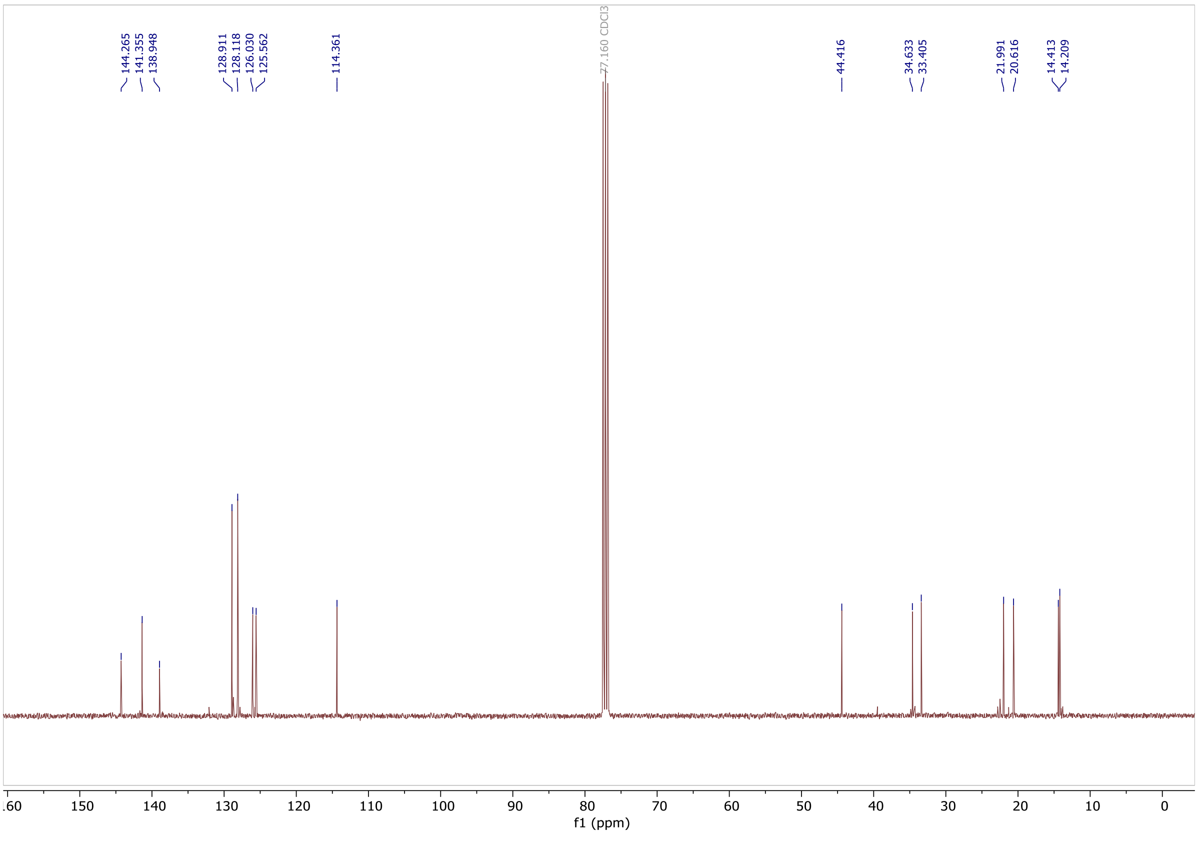

^{1}H NMR (400 MHz, $CDCl_3$) of **44**

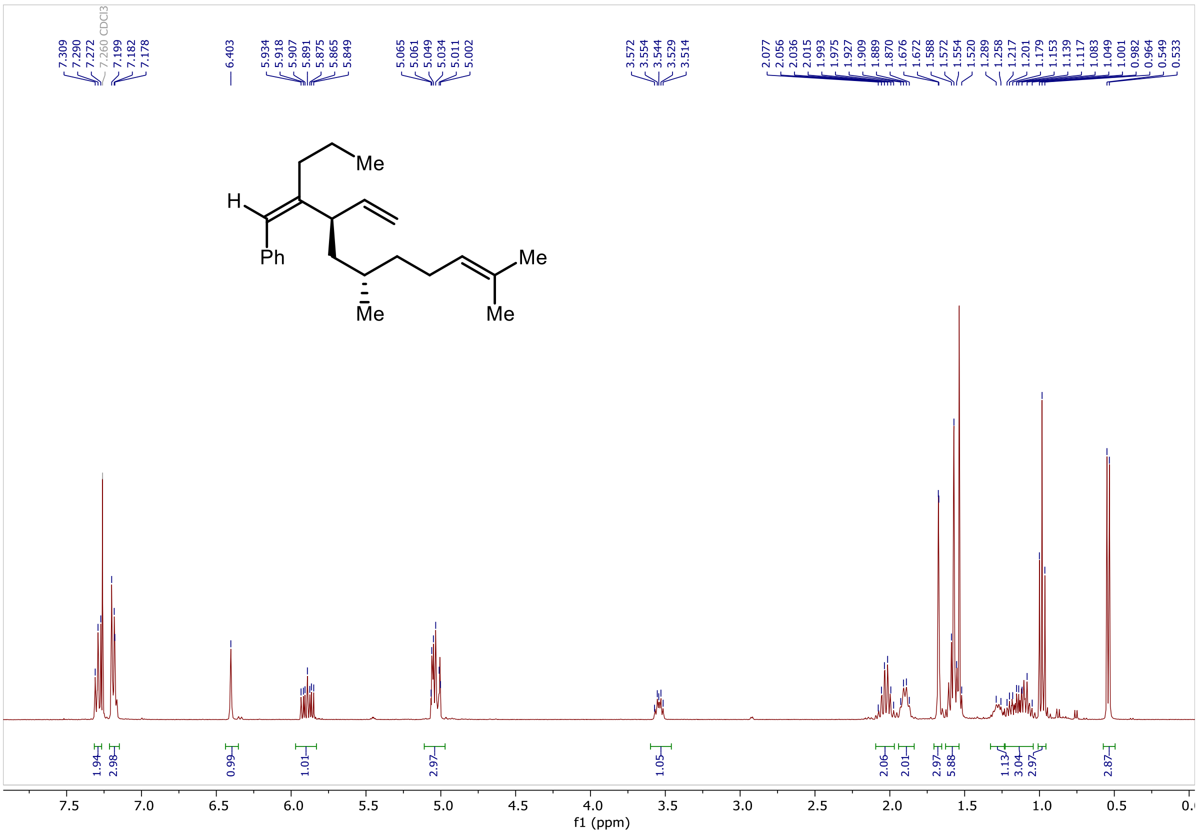

^{13}C NMR (101 MHz, $CDCl_3$) of **44**

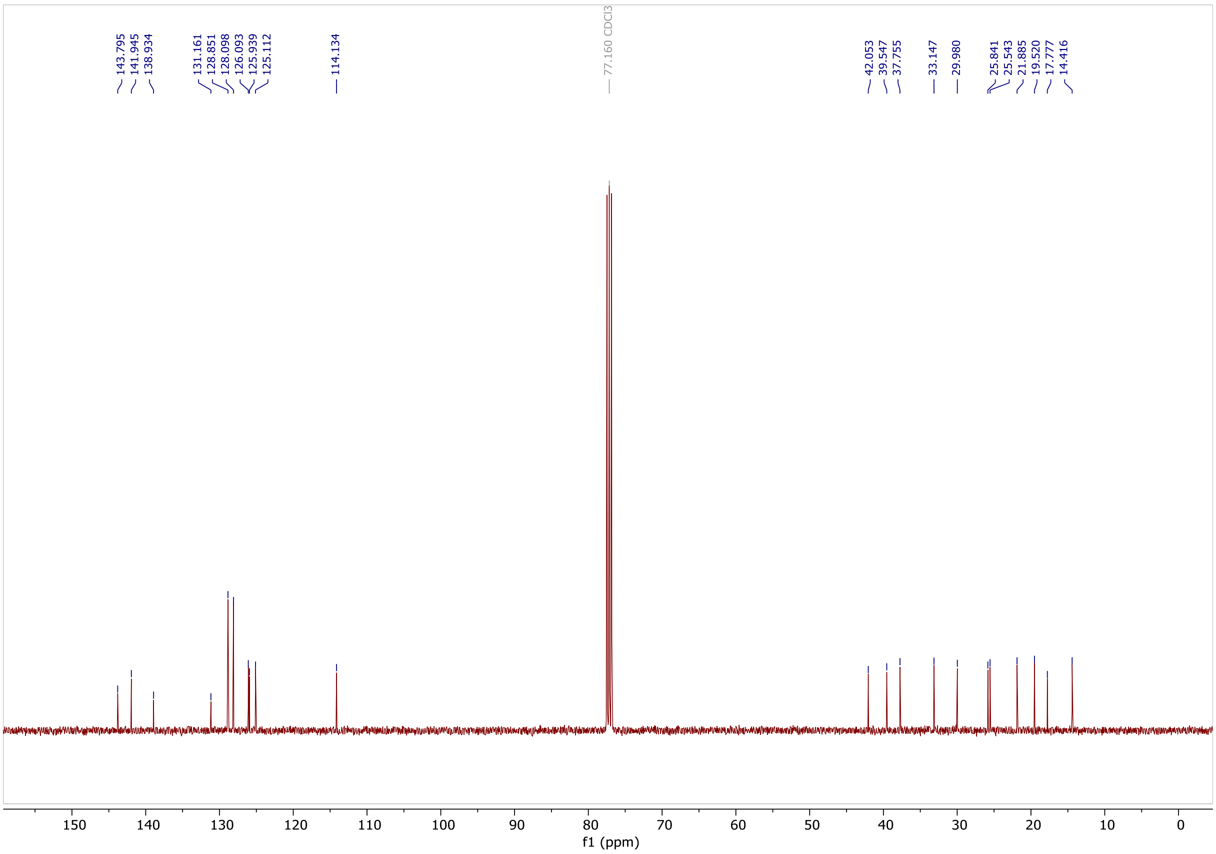

^{1}H NMR (400 MHz, $CDCl_3$) of **45**

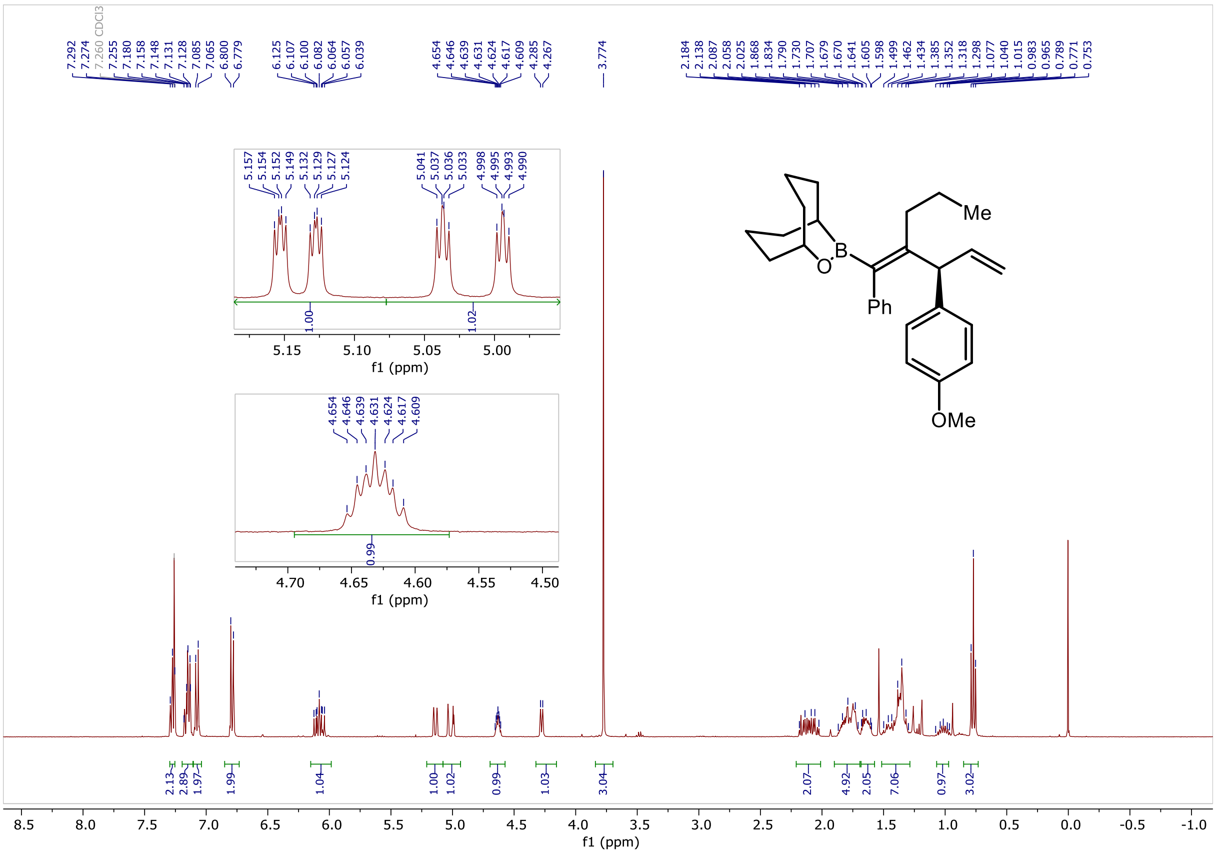

^{13}C NMR (101 MHz, $CDCl_3$) of **45**

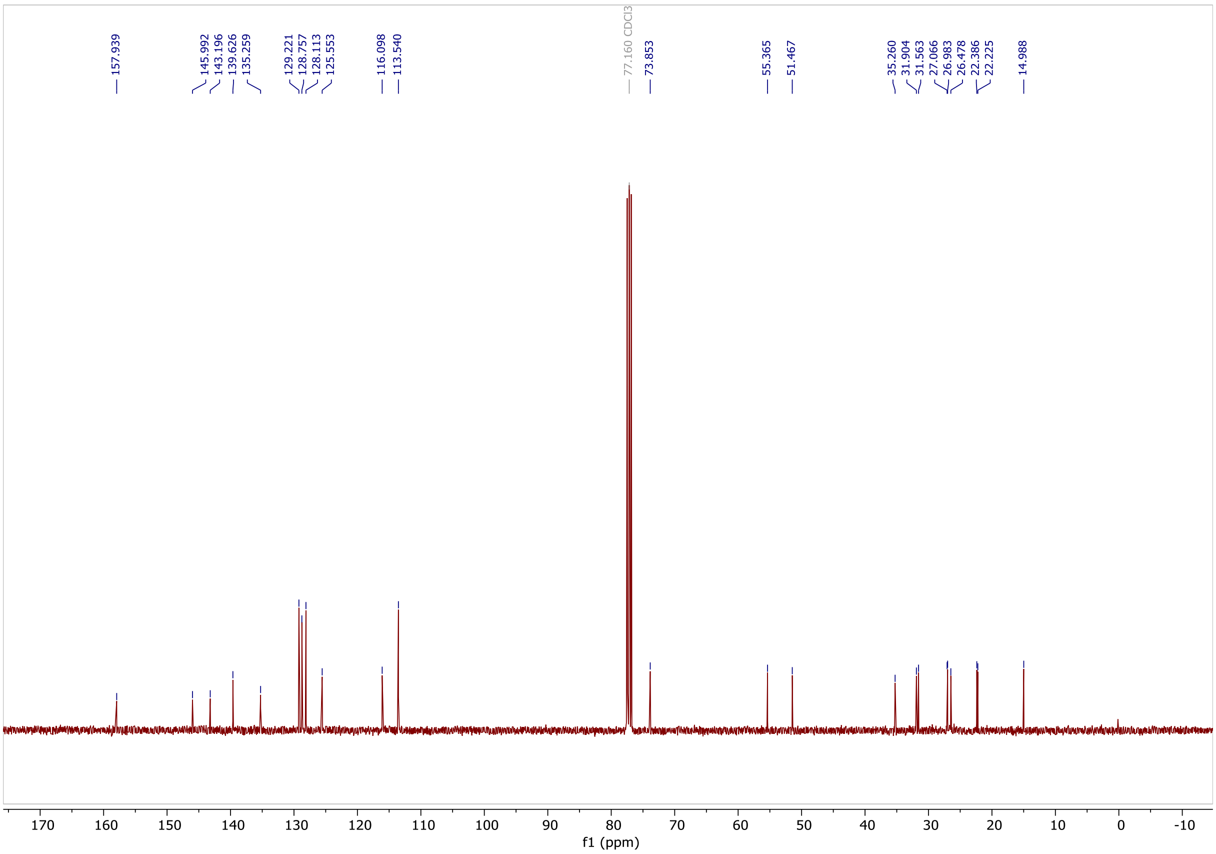

^{1}H NMR (400 MHz, $CDCl_3$) of **46**

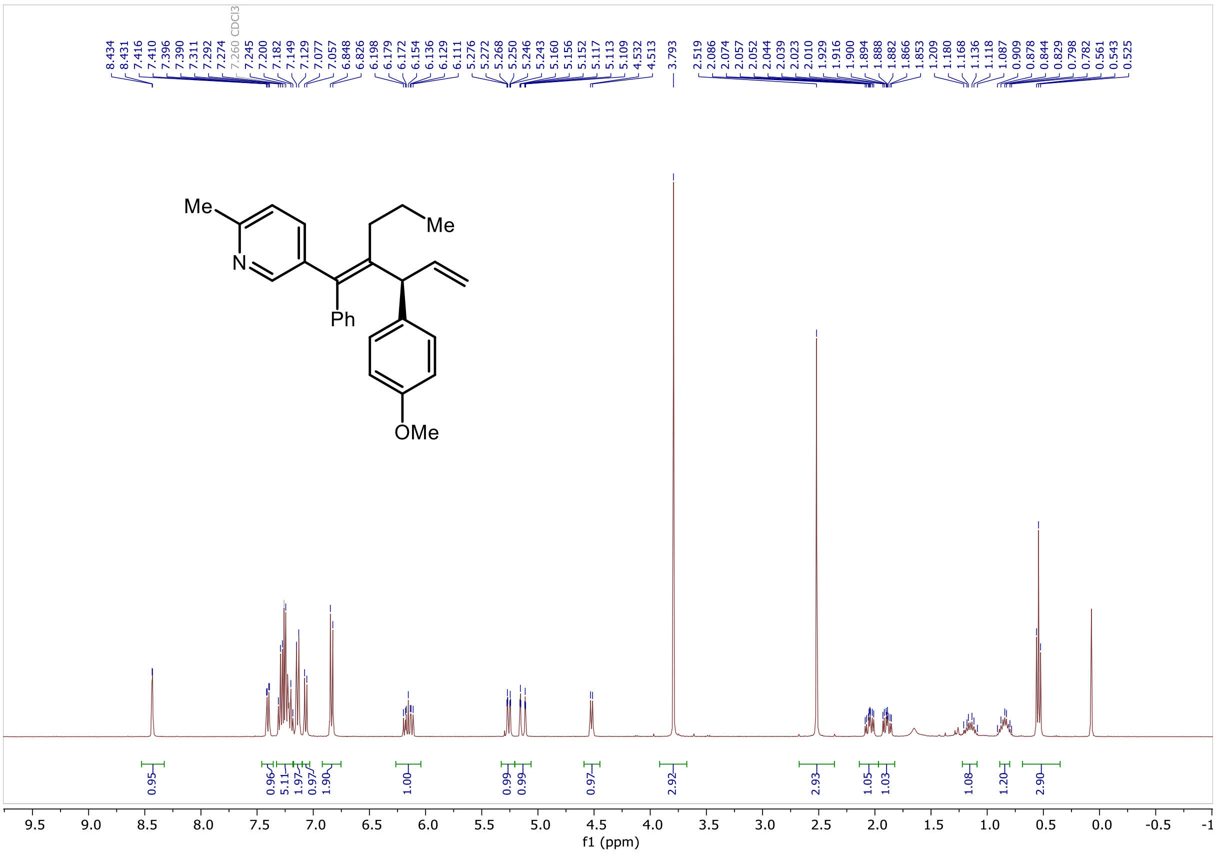

^{13}C NMR (101 MHz, $CDCl_3$) of **46**

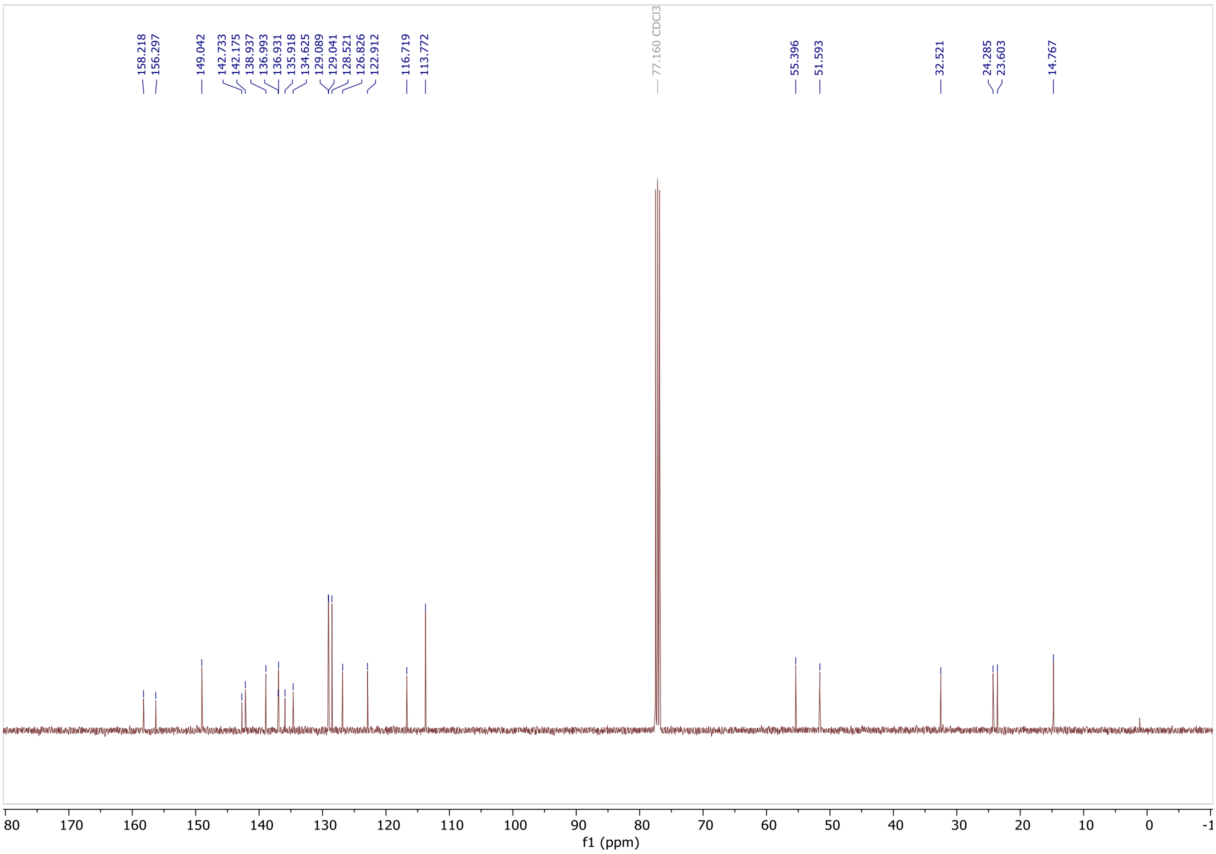

^{1}H NMR (400 MHz, $CDCl_3$) of **47**

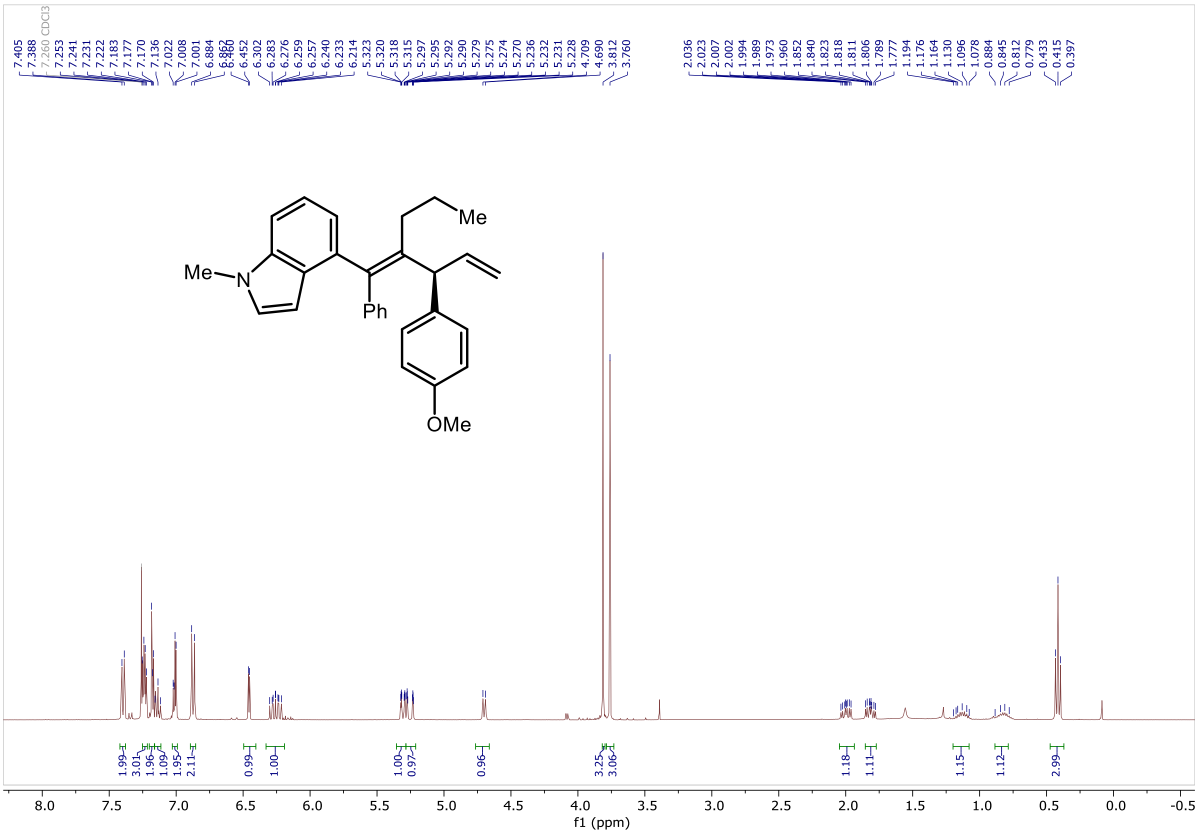

^{13}C NMR (101 MHz, $CDCl_3$) of **47**

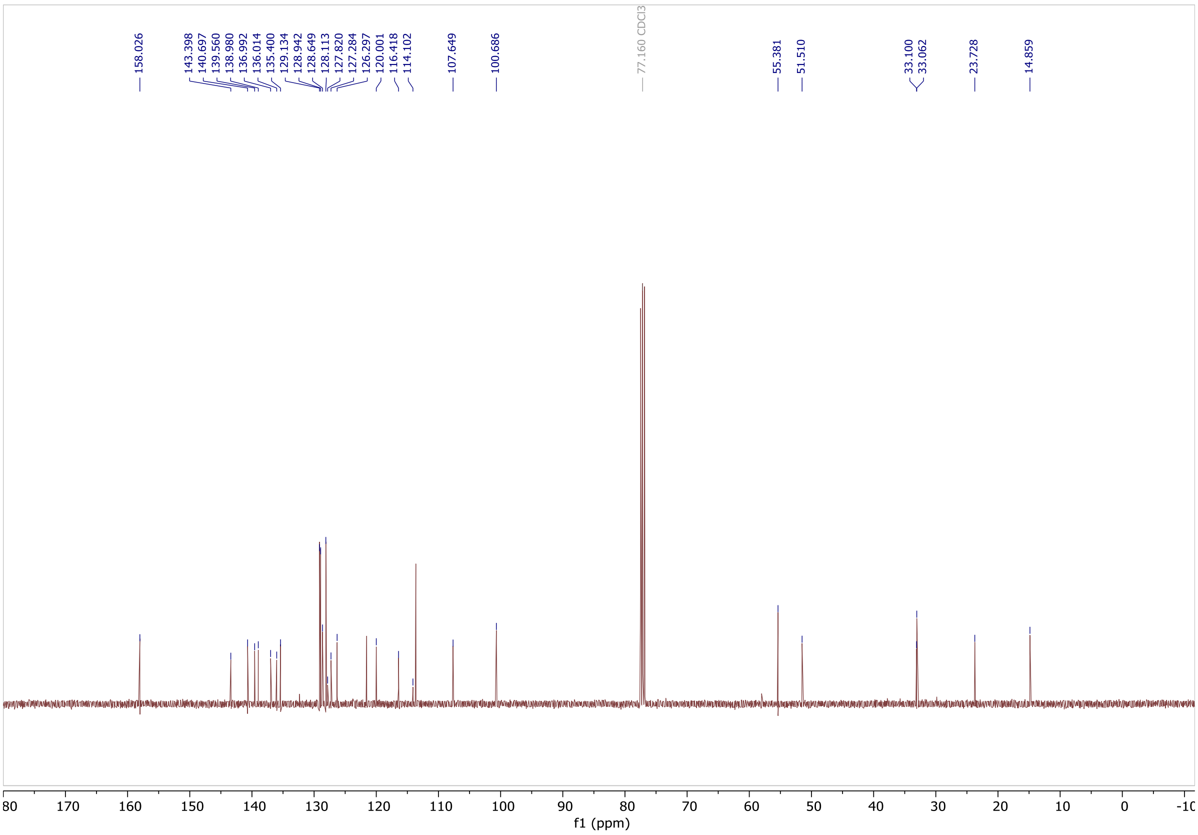

^{1}H NMR (400 MHz, $CDCl_3$) of **48**

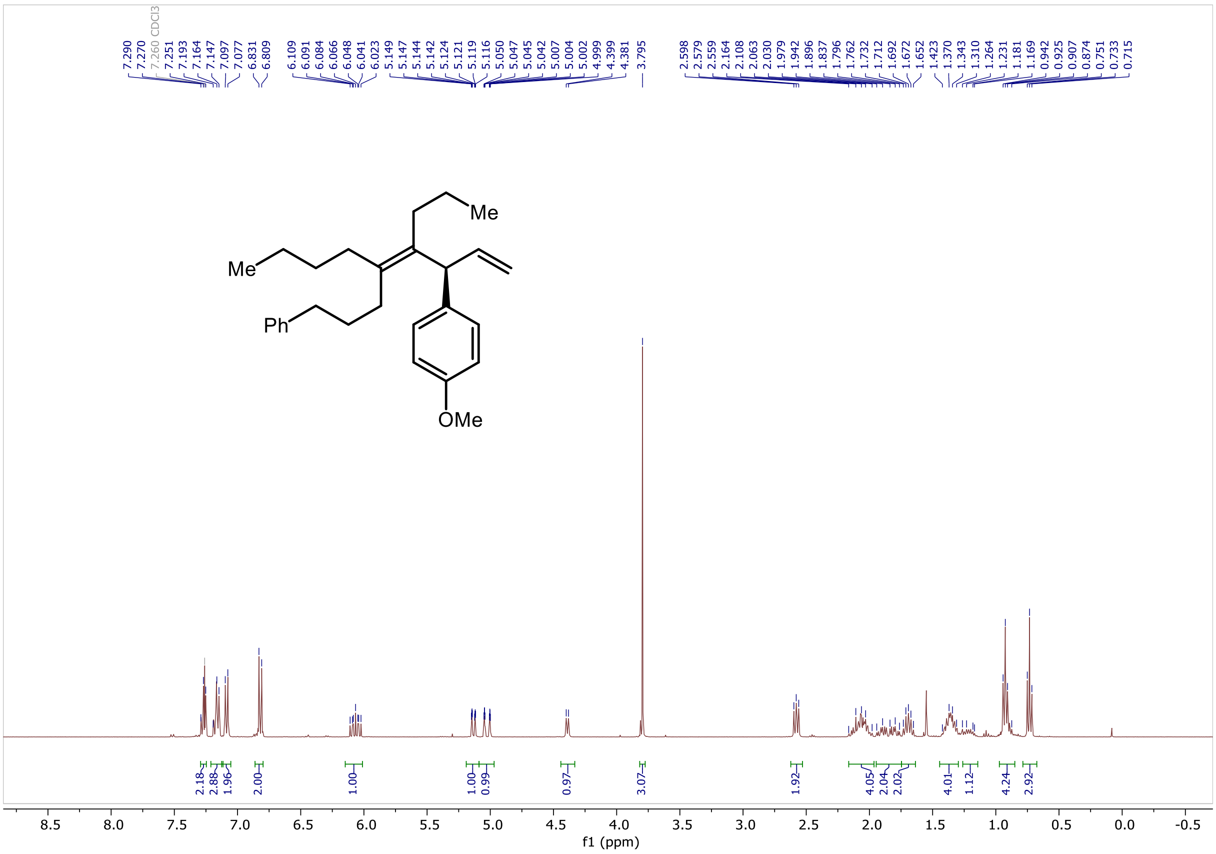

^{13}C NMR (101 MHz, $CDCl_3$) of **48**

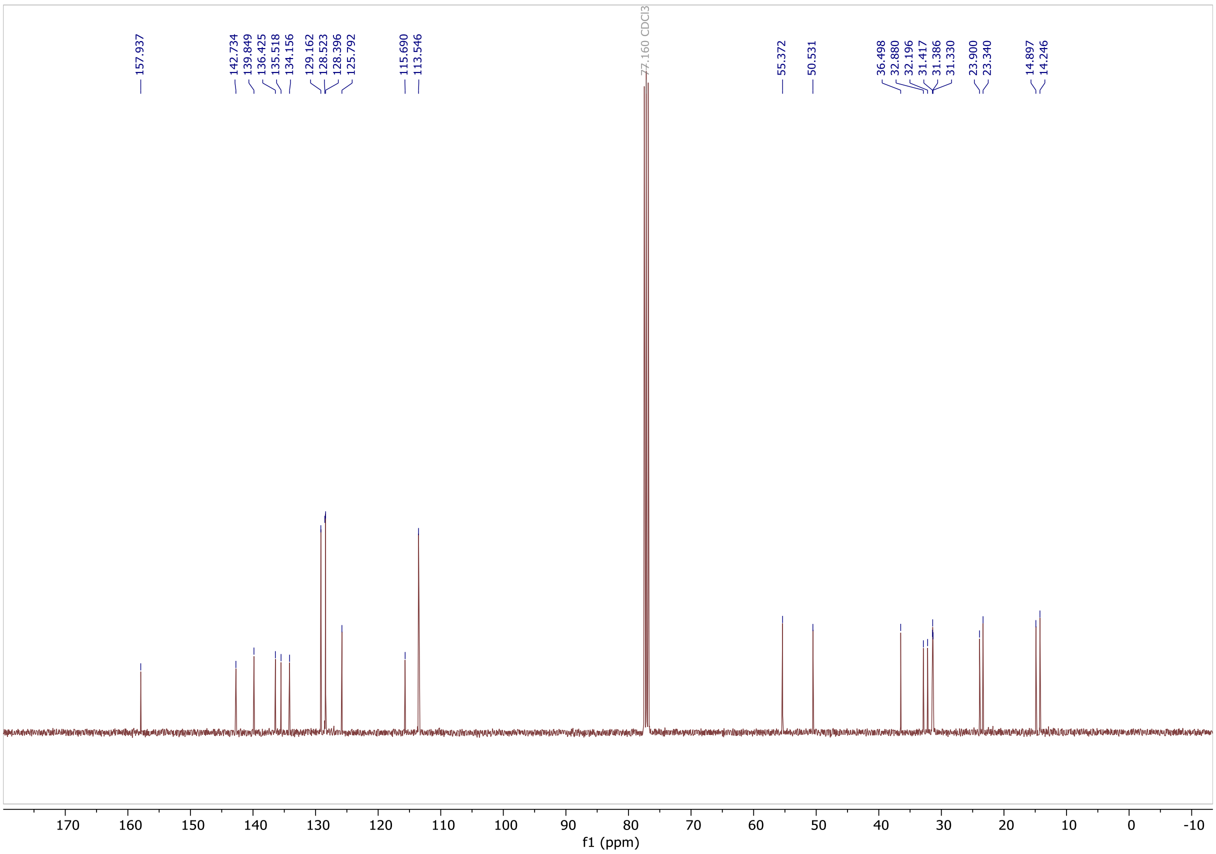

^{1}H NMR (400 MHz, $CDCl_3$) of **49**

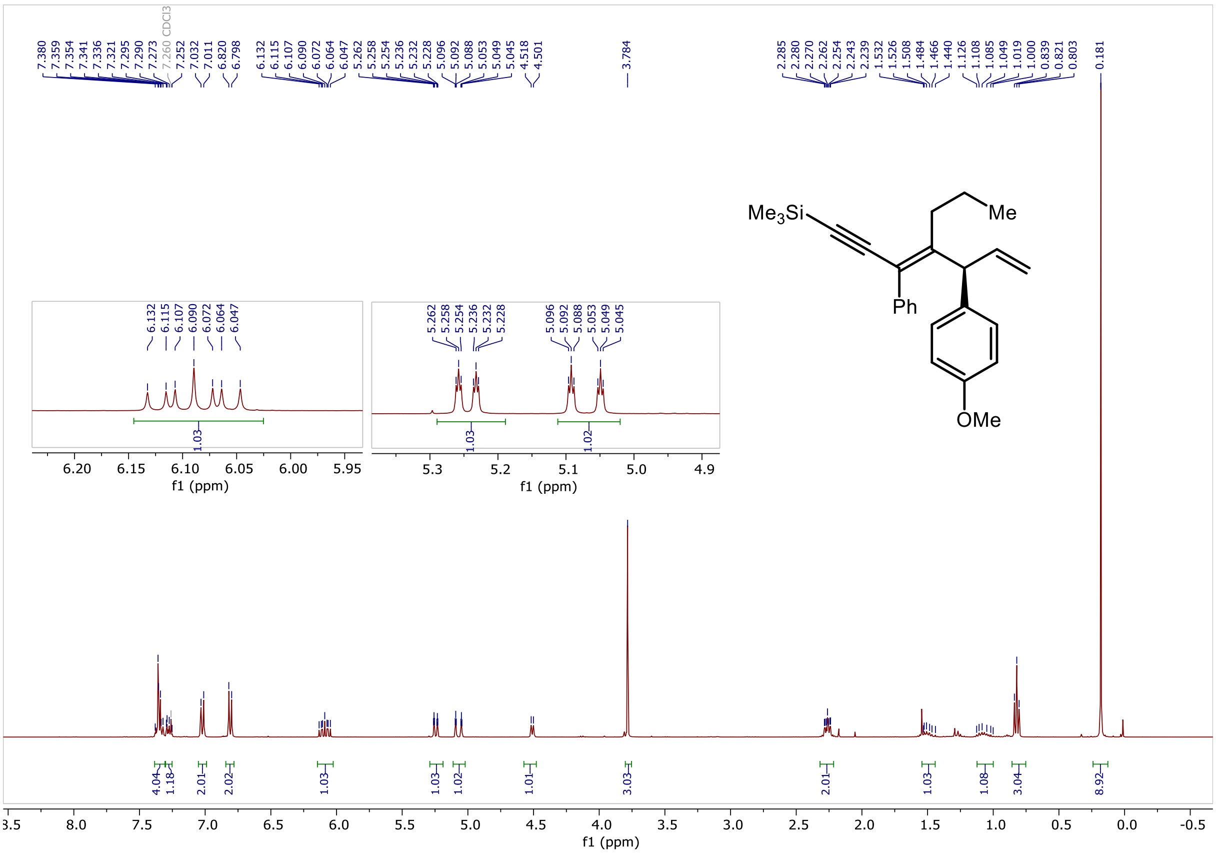

^{13}C NMR (101 MHz, $CDCl_3$) of **49**

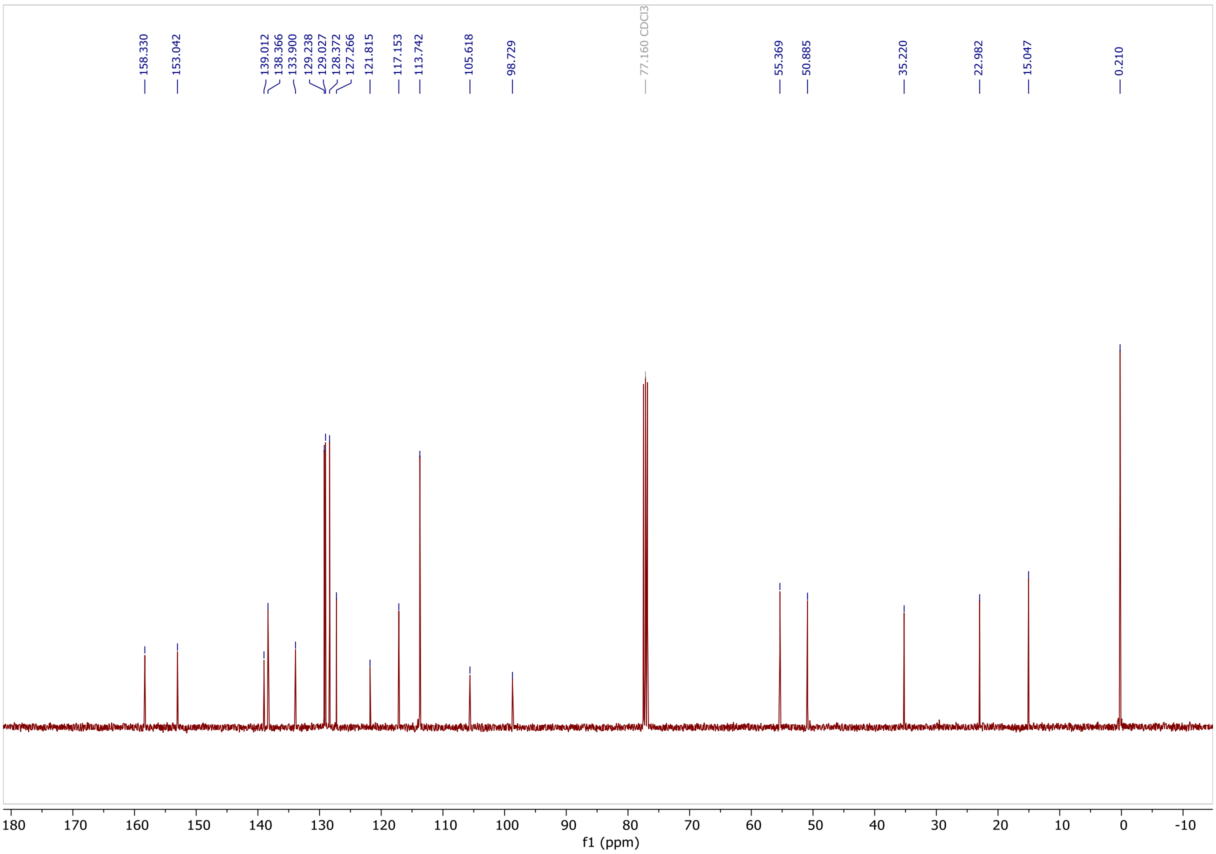

^{1}H NMR (400 MHz, $CDCl_3$) of **50**

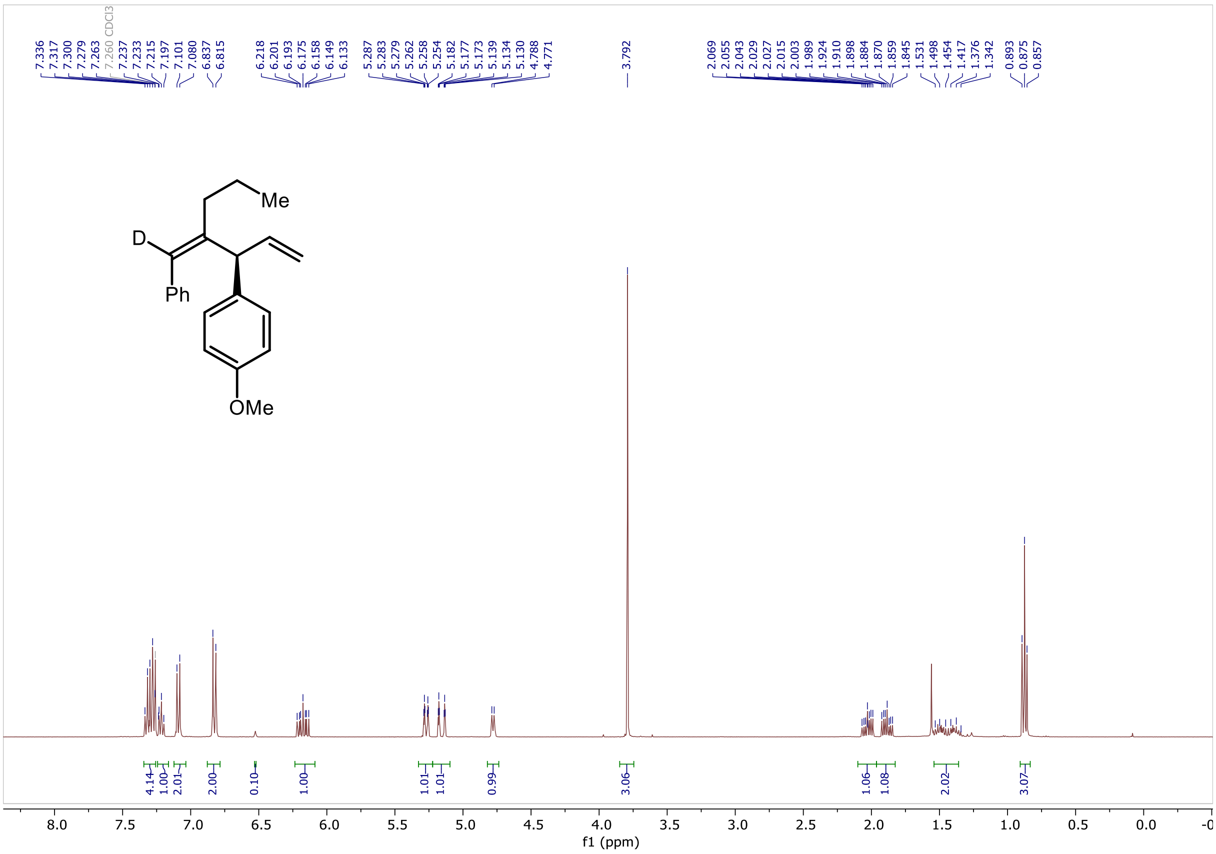

^{13}C NMR (101 MHz, $CDCl_3$) of **50**

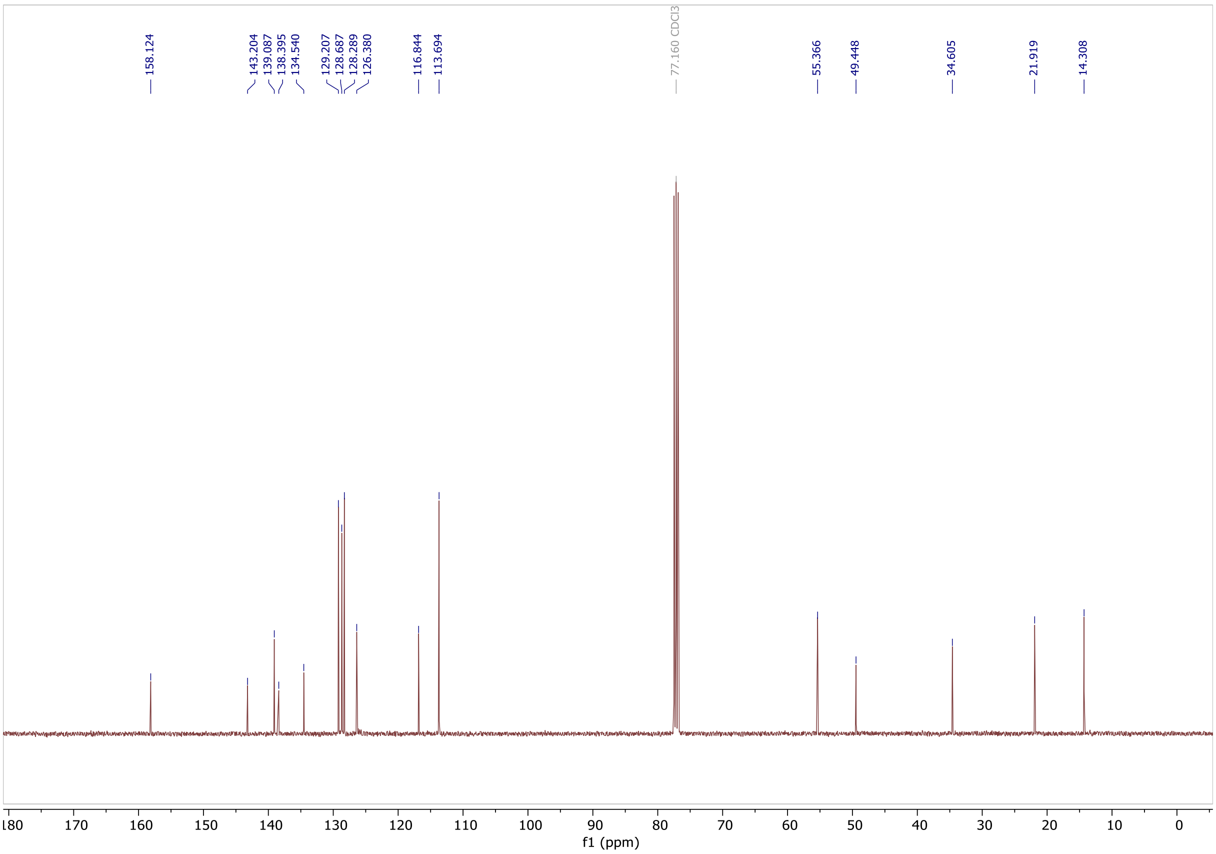

^{1}H NMR (500 MHz, $CDCl_3$) of **51**

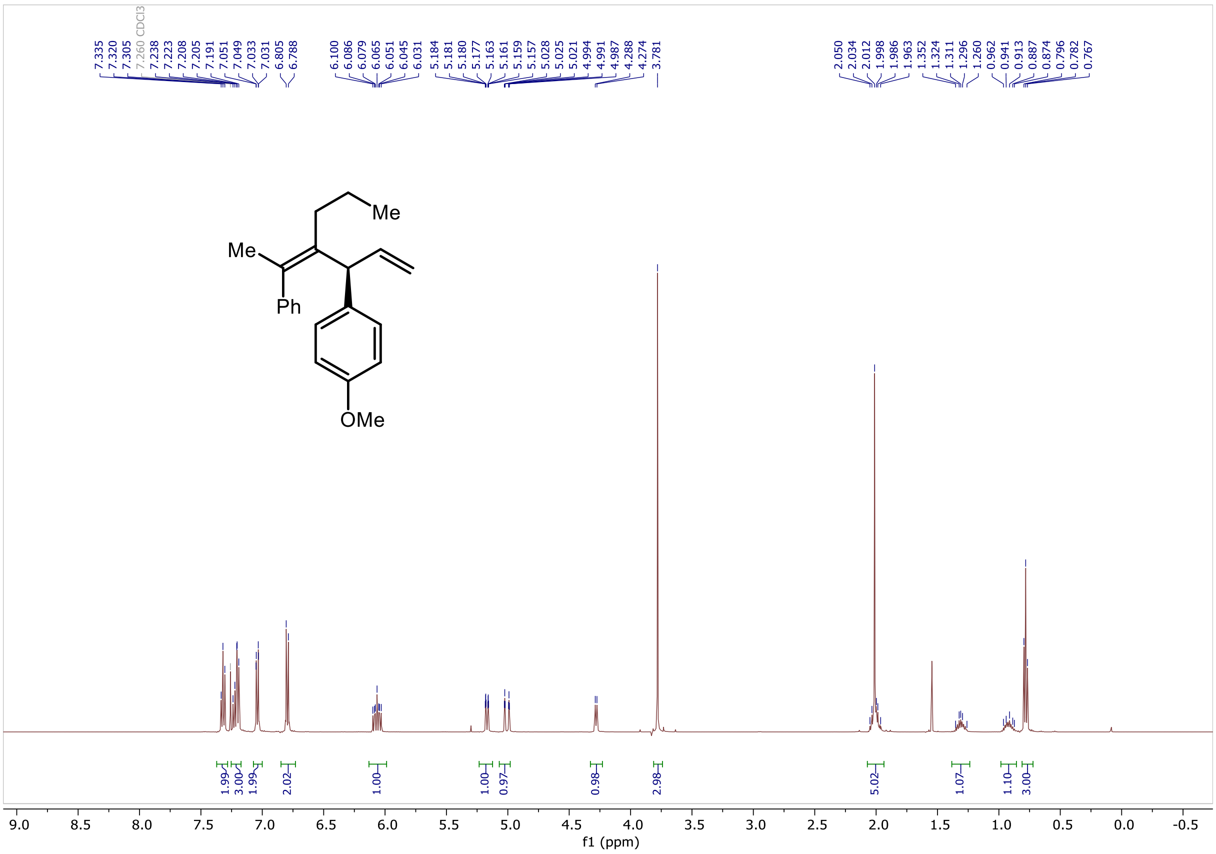

^{13}C NMR (126 MHz, $CDCl_3$) of **51**

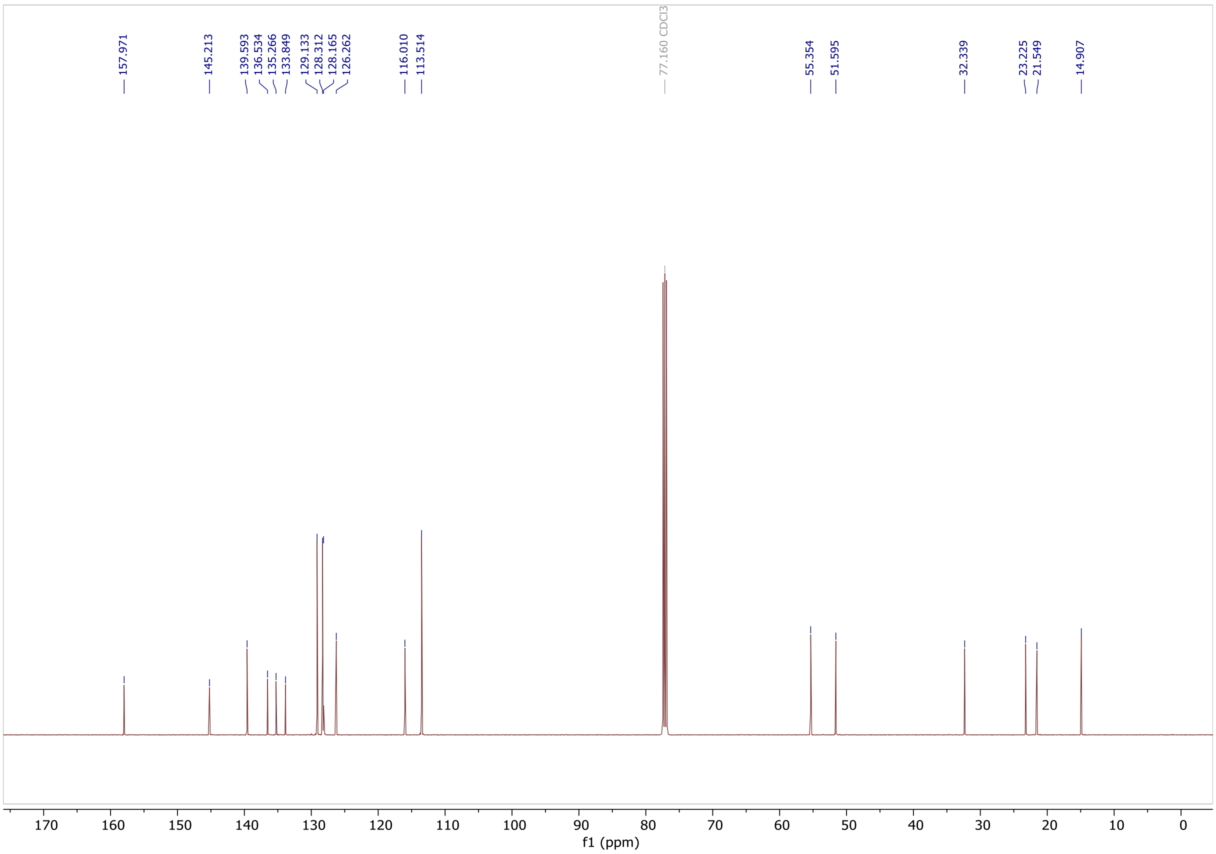

^{1}H NMR (500 MHz, $CDCl_3$) of **52**

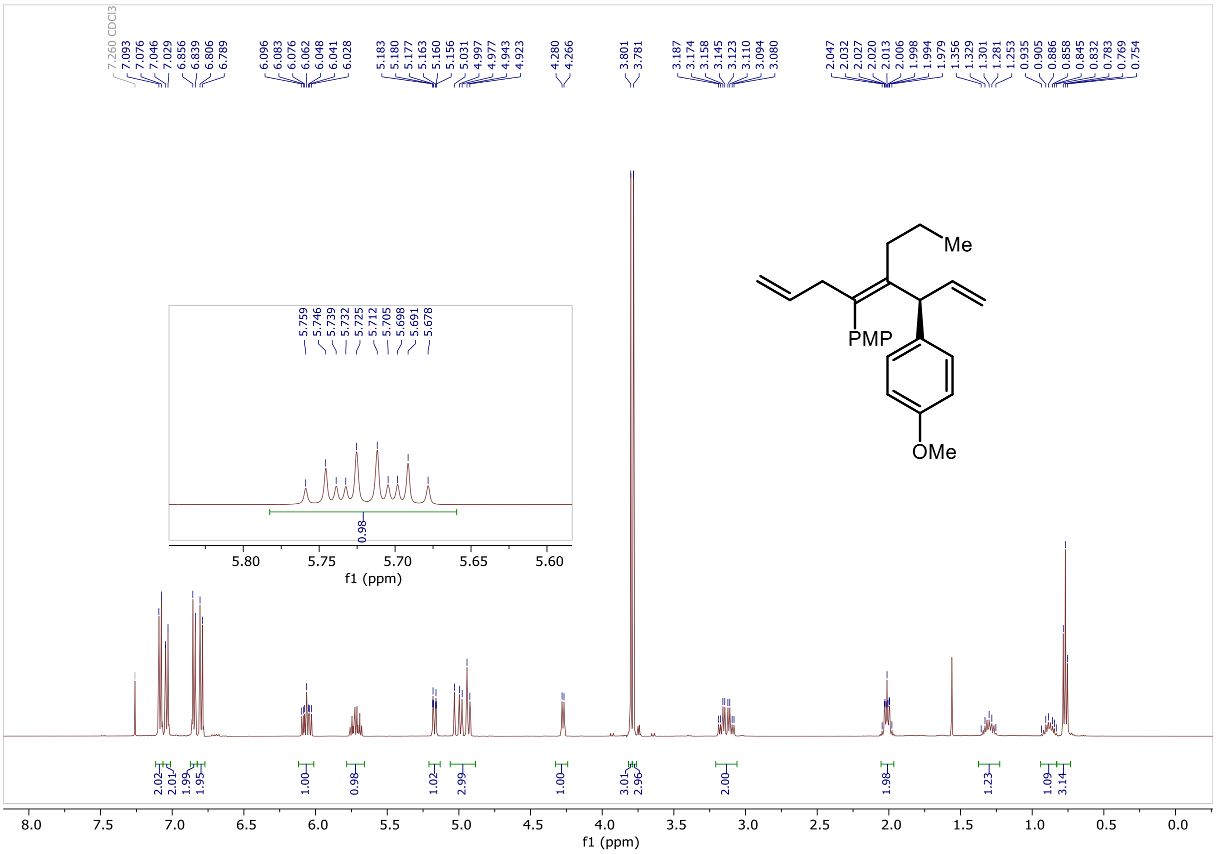

^{13}C NMR (126 MHz, $CDCl_3$) of **52**

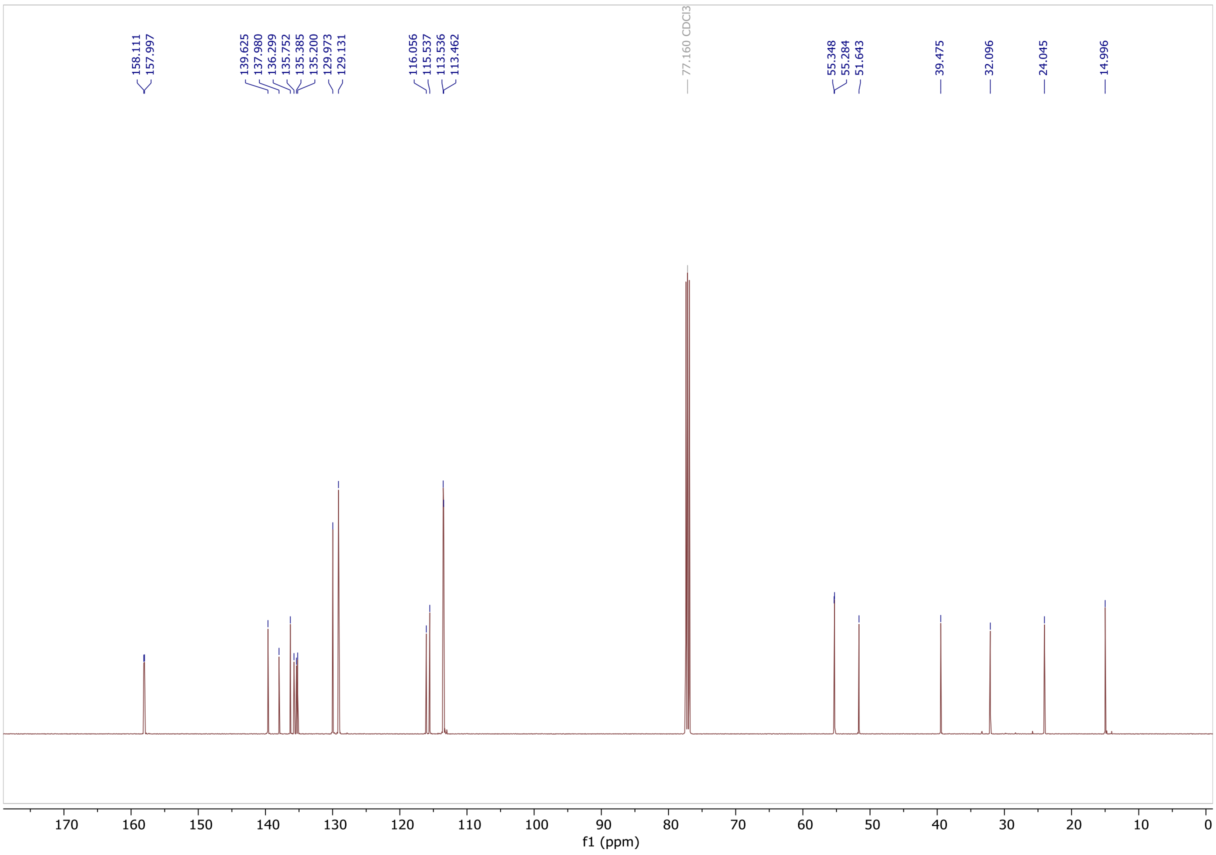

Supplement: Supplementary file 1 [file ja5c19143_si_001.pdf]
